# Periscope

**Published:** 1833-04-01

**Authors:** 


### Periscope;

OR,

### CIRCUMSPECTIVE REVIEW.

"Ore trahit quodcunque potest, atque addit acervo."

### I.

### GLASGOW ROYAL INFIRMARY.

The following cases are selected and abridged from Dr. Macfarlane's Clinical Report. They formed the concluding portion of an article in our last Number, but want of space compelled us to defer their insertion till the present opportunity.

### I. Wounds of the Bladder.

These are now known not to be necessarily fatal. If the wound is inflicted through the rectum, there is a double risk, first of urinary extravasation, secondly of recto-vesical fistula. The following case is interesting and brief.

Case 1. *Recto-vesical laceration—Fistula—cure.*

"W. J. æt. 14, when attempting to leap over a wall, on the 24th May, 1826, fell, with a good deal of force, upon the sharp iron-pikes which covered it. One of these, without producing an external wound, entered the anus, tore open the anterior part of the rectum, and penetrated the bladder behind the prostate. The injury was so extensive, that, besides the free escape of urine into the rectum, the fæces, when soft or liquid, passed readily into the bladder. For the first eight days, the febrile symptoms ran high. He had abdominal pain and vomiting, but without any swelling about the anus or perinæum, or other external evidence of urinary extravasation. He was bled, and felt soothed and relieved by the daily use of the warm hip-bath. When the local irritation and general excitement had diminished, the elastic catheter was introduced along the penis into the bladder, removed every second day, and worn for three months. At this time, there still remained a large fistulous opening, through which the greater part of the urine escaped into the rectum. He became impatient of the restraint and confinement under which he was placed, and refused to submit to the necessary treatment for the cure of the fistula. In about two years from the receipt of the injury, he again placed himself under my care. By retaining a catheter constantly in the bladder, dilating the anus with Weiss' instrument, and then applying the actual cautery to the fistulous opening, a cure was accomplished in about two months."

The next case is still more remarkable for its favourable issue. A boy, æt. 12, was gored by a bull, the horn of the animal penetrating between the coccyx and anus, passing through both sides of the rectum, and freely laying open the neck of the bladder, and lacerating the prostate and membranous part of the urethra to the bulb. In spite of all this mischief the boy was dismissed in six weeks cured.

### II. Contusions of the Urethra.

Dr. M. refrains from relating two cases of ruptured urethra which were under his care. We regret this, as such cases, fatal or not fatal, are interesting and instructive. Three cases are detailed. In two stricture of the urethra succeeded a contusion on the perinæum. We will glance at one of these.

*Case* 1. J. L. received a severe blow on the perinæum, which was followed by difficult micturition, and hæmorrhage from the urethra which lasted for three or four days, and amounted to three pints. Symptoms of stricture succeeded, and, when admitted, one was found six or seven inches from the orifice of the urethra, so small as to prevent the passage of the smallest bougie. A hard irregular tumour was also felt externally in this situation. At first it was found impossible to pass any instrument into the bladder, but at length a very small sound was got in, and at the end of six weeks a full-sized one could be introduced. In the other case the occurrence of stricture was prevented by timely measures.

*Case* 2. W. B. æt. 16, struck his perinæum in crossing a wooden fence. In an hour afterwards, profuse hæmorrhage from the penis took place when attempting to make water, and in three days it recurred, about four pints being lost in all. Eight days after the accident he was admitted, having then some swelling and tenderness in the perinæum. *Acidulated drinks—leeches to the perinæum—cold applications—recumbent position.* In ten days the perinæal swelling was reduced to a defined tumour, about the size of a filbert, encroaching on the urethra, diminishing the stream of urine, and occasioning pain in its expulsion. These symptoms were removed by leeches, fomentations, iodine frictions, and the daily introduction and retention for an hour or more, of a large metallic sound.

"The history of this case, the existence of a tumour in the perinæum, where the injury was inflicted, and the profuse hemorrhage that was produced, showed that the urethra was partially lacerated; and the fact of the blood escaping, unmixed with the urine, was conclusive as to its not having proceeded from the bladder or kidneys. We may infer from the amount of the hemorrhage, that the injury to the urethra was considerable; yet the external swelling was trifling, and there was no threatening of urinary extravasation, an occurrence which almost uniformly attends the bursting of the urethra. Its absence can only be accounted for on the supposition, that the laceration did not extend through the entire substance of the urethra, or that, from some accidental circumstance, the aperture was closed against the stream of urine. When the laceration of the urethra is not so extensive as immediately to give rise to extravasation, I have succeeded in preventing it in two cases, by introducing a large elastic catheter into the bladder, and retaining it for several days, until the danger was warded off by the sides of the lacerated opening becoming consolidated."

### III. URINARY ABSCESS.

Practical surgeons know what a formidable affection extravasation of urine into the cellular tissue is, and they also know that early scarifications will frequently arrest completely the progress of the mischief. But those scarifications must be very early and equally bold, or the cellular tissue will be destroyed so widely that persons of enfeebled constitutions will be unable to sustain the effects of its sloughing. We will not quote a case related by Dr. Macfarlane, though both important and instructive. Diffuse cellular inflammation of the scrotum and contiguous parts is occasionally mistaken for extravasation of urine. The mistake is of no great consequence, saving that the treatment is rendered unnecessarily severe. But sometimes a worse error is committed, extravasation of urine being looked upon as erysipelas. In such a case bad consequences might ensue from the neglect of free incisions. Such a case is mentioned by Dr. M.

*Case.* "I was requested by a surgeon in town to visit an old soldier, who had long laboured under stricture of the urethra. Eight days previously, a small painful tumour began to form in the perinæum, which gradually increased; and was followed, in four days by a sudden swelling of the scrotum and penis, which extended rapidly to the groins, upper part of the thighs and abdomen, and was speedily productive of redness of the integuments; which, in a few hours, assumed a violet colour. The urine continuing to dribble from the penis, it was supposed that the disease was erysipelas, and that the external swelling had no connexion with the urethra. I made several free incisions into the affected parts, which were gangrenous, and gave exit to a large quantity of fetid urine, mixed with pus. Through one of these openings, in the situation of the original tumour in the perinæum, I succeeded in passing an elastic catheter into the bladder, and drawing off two pounds of urine. From the extent of the extravasation, the dissipated habits of the patient, who was a dram-drinker, and had been long in a warm climate, and the great typhoid depression which existed, but little benefit followed this treatment. He did not survive more than twelve hours.

On dissection, besides the great destruction of the cellular texture and integuments externally, it was found that the urine was extensively extravasated into the cellular substance of the pelvis, —having passed along the crura of the penis, and under the symphisis pubis, so as to form a communication with the sloughy cavity on the surface of the abdominal muscles."

In erysipelas attacking the scrotum the progress of the case is not so rapid, the general symptoms are not so typhoid, and the history is different. We need hardly say, however, that if the swelling be great in erysipelas, the cellular texture, especially that of the scrotum will certainly die, and incisions are equally necessary and equally useful here, as after extravasation of urine. We have on several occasions seen patients die with symptoms that puzzled the lookers-on very much. A man, for instance, was in a hospital for some disease of the rectum. Suddenly he was attacked with low typhoid fever, without apparent cause. The typhoid symptoms increased, and in two or three days the patient died comatose. On dissection a circumscribed abscess was found in the deep cellular tissue of the pelvis, unattended by any obvious communication with either the urinary organs or intestines. After lithotomy such circumscribed abscess of the pelvis occasionally occurs. The symptoms are insidious :—faint rigors, or none—frequent pulse and low fever—perspirations—towards the close typhus and delirium. We merely mention this affection, and pass on.

### IV. Abscess of the Prostate Gland.

Dr. Macfarlane observes that when abscess occurs in the prostate, the investing and interlobular cellular substance of the gland is usually the part affected, the gland itself remaining sound.

Case 1. *Abscess of the Prostate discharged by the Urethra—Cure.*

W. H. æt. 36, admitted Dec. 9th, 1831. Had frequent, difficult, and painful micturition, pain in the loins and dull uneasiness, with occasional throbbing in the perinæum. He had some pyrexia. The introduction of a catheter occasioned severe pain at the neck of the bladder, where much irregularity was felt, and, on examination per anum, the prostate was found enormously enlarged, but smooth and soft. The symptoms had commenced two months previously, and in three weeks the abscess had burst, and the matter unmixed with urine was discharged by the urethra. The treatment now consisted of frequent leeches to the perinæum, small doses of ol. ric. every second morning —the hip-bath and an occasional suppository at night. The symptoms gradually decreased, the purulent discharge was arrested, and in three weeks the difficult micturition had disappeared.

" This was a distinct case of phlegmonous inflammation of the prostate, which ended in suppuration, and could not be traced to any obvious cause. When there is a free communication between the abscess and the urethra, a simple straining effort will, in many cases, be sufficient to expel along the penis a considerable quantity of pus, unmixed with urine ; but in general, the discharge in quantities takes place only when the patient empties his bladder ; and then it may either precede or follow this evacuation, or the matter may be so mixed with the urine, as to render it turbid immediately on its being discharged.

When the enlargement of the prostate is great, and especially when the abscess involves the middle lobe, there is always more or less difficulty in expelling the urine. This sometimes amounts to complete retention, and requires the introduction of a catheter; but this ought to be employed only in the most urgent circumstances, as the frequent introduction, or continued retention of the instrument, will be found highly injurious.

When suppuration is fairly established, which may either be in the substance of the gland itself, or in the cellular texture which surrounds it, and unites its lobes together, it is but seldom we can succeed in detecting its existence by manual examination. I have only met with one case, in which, on introducing the finger into the rectum, distinct fluctuation was felt in the tumour: it was large, and projected considerably into the cavity of the bowel. This was punctured three times with a trocar, and a cure accomplished. The pain during the expulsion of the feces was most acute, and there was distressing tenesmus, from a sensation as if there was a hard body lodged in the rectum; but the pains in, or the impediment to, the evacuation of the bladder, was less urgent than usual."

CASE 2. *Abscess of the Prostate—ultimate Cure.* W. P. æt. 20, was seized, after a horse had fallen on him, with acute pain about the neck of the bladder stretching to the glans, frequent and painful micturition, tenesmus, and violent throbbing in the perinæum. In three weeks he began to discharge large quantities of pus, separate from and mixed with the urine. In three weeks more he was admitted with these symptoms, accompanied with hardness and pain in the left epididymis, as well as in the prostate. Under antiphlogistic treatment, hip-baths, and anodyne enemata, he improved so much that he was dismissed in a fortnight nearly well. In four days after this, acute throbbing pain in the perinæum with almost complete obstruction of urine came on, but were partially relieved by a sudden and copious discharge of pus from the urethra. On the next day he was re-admitted with painful and difficult micturition, each attempt accompanied by tenesmus and a desire to go to stool. The prostate, especially its right lobe, was larger than before. Under leeches, baths, &c. the symptoms were mitigated, and the discharge diminished. But in a fortnight after this second admission he became feverish, with increase of the symptoms, and retention of urine. The bladder was distended above the pubes, and the swelling of the prostate was augmented. It was necessary to use a catheter. When it touched the prostate it produced excruciating pain, and could not at first be passed. On another attempt two ounces of pus suddenly escaped, and the instrument then slipped into the bladder, and gave issue to two pints of urine. It was not necessary to use it again. After this the symptoms were—a call to make water from every quarter of an hour to every two hours, pain in expelling the last drops, and purulent discharge. The muriated tincture of iron was tried, but it aggravated the symptoms. Leeches, baths, and liquor potassæ were then employed with more benefit, and in a month from the second admission he was discharged cured.

" When the irritation at the neck of the bladder is great, the testicle becomes often affected. In the last case, the inflammation seemed to have extended along the cord, producing hardness and thickening of the epididymis, without the gland becoming involved. This irritability of the prostatic portion of the urethra sometimes continues long after the abscess has closed, and is the cause of painful and impeded micturition. For this, in addition to the usual local and soothing treatment, I have experienced great advantage from small doses of the liquor potassæ."

Dr. Macfarlane observes that in old persons abscess of the prostate sometimes proves fatal; by giving rise to urinous extravasation. An instance of this is given. The patient was an old soldier, who had long had stricture. He was admitted with rigors and retention of urine. A gum catheter was passed, but gave excessive pain, and was immediately withdrawn. Purulent discharge from the urethra followed; in a few days it was again necessary to use the catheter, which was left in the bladder for some days. A copious discharge of fetid matter and typhoid symptoms rapidly carried the patient off. On dissection the substance of the prostate formed a large suppurating cavity, through which the urine was extensively extravasated into the cellular tissue of the pelvis and perinæum.

This completes the series of cases of disease of the urinary organs. We have only to advert to those illustrative of some of the affections of the rectum and external parts of generation.

### V. Prolapsus Ani.

Dr. M. relates three cases of this affection in order to direct attention to the utility of the treatment recommended by the late Mr. Hey. In each case the patient was advanced in years, and in each there was not simply procidentia of the mucous membrane of the gut, but also hæmorrhoidal thickening of part of it. In the second case related by Dr. M. there was hæmorrhage after the operation, and that to some extent, an instance among many of the paramount necessity of carefully watching all cases of operation about the rectum. In the present instance the dilator was used, and a small bleeding vessel secured within the sphincter. With these remarks, we think it only necessary to extract one case, the third, and the most severe.

*Case.* "W. A., aged 54, became a district patient in the beginning of February, 1829, on account of the above troublesome disease. The gut descended for more than two inches on every attempt to evacuate the bowels, accompanied by considerable pain and tenesmus. When he remained for a few minutes in an erect position, the same displacement took place slowly, although no propulsive efforts were employed; this, however, he could prevent by pressure on the anus. There was first projected from the anus, a circular fold of the mucous membrane of the rectum, at its verge, of a livid colour, and tuberculated appearance, soon followed by the complete descent of the bowel and hæmorrhage from innumerable points. He generally succeeded in replacing the part, by assuming the recumbent posture, and maintaining gentle, but continued pressure for a few minutes. At an earlier period, however, the protrusion often continued for hours, before it could be returned. His general health was greatly impaired, and he was unable to follow his employment, from the continued irritation and almost daily attacks of hæmorrhage.

On examining the anus after the gut was replaced, the surrounding integuments were found extremely relaxed. There existed such an unnatural looseness in the attachment of the skin around the anus to its corresponding cellular membrane, that it could be easily drawn out with the fingers, in the form of one or more large flaps. Having succeeded in two similar cases, which came under my care in the Royal Infirmary, during the summer of 1826, in completely curing the disease, by cutting off the loose integuments, as recommended by the late Mr. Hey,* I determined to try it in this case. The skin was drawn as far out as possible into broad flaps, and cut off with the scissors in a circular direction, until all the superfluous integument was removed, including a portion of the livid and tuberculated fold of mucous membrane which was projected from within the sphincter. The pain was trifling, and only a few drops of blood were lost. A soft compress and T bandage were applied, and he was strictly confined to bed. For a few days, a partial procidentia took place on every attempt to go to stool. He had a good deal of pain and inflammation around the anus, with complete retention of urine, which required the frequent introduction of

* "Practical Observations in Surgery, 2d edit. p. 444."

the catheter. In ten days after the operation, he was able to walk about, and void his stools, without any return of the disease, and in three weeks he was perfectly cured. Pressure was continued to the part for some time longer, occasional doses of castor oil were prescribed, and he was enjoined to avoid straining at stool."

Dr. Macfarlane adds the following remarks.

"There will generally be found in obstinate and long-continued forms of this disease, a great relaxation in the connexion of the rectum at its lower part, with the surrounding textures. This circumstance, although it may not be the original cause, is sufficient, in many cases, to account for the continuance of the displacement in chronic and inveterate cases, although I believe it is generally accompanied by a diminished power of the sphincter. If the rectum, in consequence of being much irritated, as in various bowel complaints, ultimately becomes relaxed, the tenesmus, which is an invariable attendant, may so overcome the sphincter as to give rise to a procidentia. But when, as in the case now detailed, the erect position is sufficient to cause a descent of the gut, we have grounds for believing that, besides the relaxed state of the rectum, there exists a want of power in the sphincter muscle, which part, along with the levator ani, is mainly instrumental in maintaining the rectum in its natural situation. In the cases detailed by Mr. Hey, there existed, in combination with relaxation of the integuments, one or more livid tubercles at the verge of the anus, which were also removed. He expected from this operation, that inflammation of the surrounding cellular texture would be excited, the attachments of the rectum consolidated, and the power of the sphincter improved. In a majority of cases, the disease will be found to yield (although the cure is often tedious and protracted) to the local applications and internal remedies usually employed. Should it continue, however, as sometimes happens after the exciting cause has been removed, we will occasionally find that the loose state of the skin around the anus, and the relaxed attachments of the rectum at its termination, become the primary causes of the continuance of the disease. It is, I conceive, in such circumstances that this simple operation may be beneficially adopted."

### VI. MORBID ENLARGEMENTS OF THE CLITORIS AND NYMPHÆ.

The clitoris and nymphæ are well known to be occasionally enlarged. Sometimes they present, especially the latter, a sort of warty tumour, which may attain considerable size. This may be attached by a narrow pedicle, or it may have an extensive surface of origin and growth. The nymphæ, in such cases, are usually removed with success. We will select Dr. Macfarlane's second case.

CASE.—*Enlarged Clitoris and Nymphæ successfully extirpated.*

Mrs. P. æt. 46, admitted Feb. 14th, 1827. The clitoris was nearly eight inches long, and of a pyriform shape; its pedicle soft, as thick as the wrist, and traversed by varicose veins: its most depending part hard, nodulated, and as large as two fists. The nymphæ hung beside the pedicle for two inches and a half, were irregular, covered by a thin white cuticle, and had a fleshy feel. The inner surface of the labia was covered by small tubercles, each about the size of a split pea, and situated immediately under the mucous membrane. On the 20th, the parts were amputated. The hæmorrhage was profuse. A catheter was introduced, and retained for several days, and when the wound began to granulate, the urine was daily drawn off. The parts were healed in about three weeks, and the patient has had no subsequent return of the disease. The diseased parts were solid and fibrous, the nodules appeared to be in some degree distinct, and were seated immediately under the investing integument.

"The clitoris of the female, as well the penis of the male, may be affected with cancer. When the crura are enlarged, indurated, irregular, and painful, we may rest assured that the disease is beyond the reach of the knife, especially when the pendulous part of the tumour exhibits the characters of carcinoma. I was present, several years ago, at an operation for the removal of a cancerous clitoris: in this case, the nymphæ were not affected. The patient was secured as for the operation of lithotomy, and an attempt made to remove the diseased crura. After a painful and protracted dissection, during which the patient lost above a pound of blood, it was found impossible to remove all the affected parts. In a few weeks, a small fungous tumour began to project immediately above the meatus urinarius: it gradually enlarged; occasioned acute pain; bled profusely; and ultimately produced death. We ought to recollect, however, that, in a simple, benign enlargement of the clitoris, the crura may also be affected; but that this ought not to militate against the amputation of the diseased parts; for if the crura be divided, and allowed to remain, they will soon shrink and disappear."

A case of carcinoma of the clitoris is given. The woman was forty-five years of age, and attributed the commencement of the disease to a kick, eleven years previously. The clitoris was about the size of the fist, hard and irregular, containing towards its apex several deep ulcerated excavations with thickened and everted edges, from which issued a thin sanious discharge, that produced painful excoriations. There were lancinating pains. There were also vaginal discharge, pains in the back and loins, and an indurated, painful, and apparently ulcerated state of the os uteri. She was sent home, and died in four months. The structure of the os uteri and clitoris was decidedly carcinomatous; there were diseased glands, some presenting the same structure, in the groins and pelvis: the disease had extended along the crura of the clitoris to their origin from the ischia and ossa pubis.

### VII. Imperforate Vagina.

A curious case of this kind is given from our author's private note-book. The operation was fatal, and no uterus existed.

Case. Mrs. C. æt. 28, consulted Dr. Macfarlane Feb. 4th, 1823. She was stout and well-formed, but pale and sickly. She had suffered from severe epistaxis, with vertigo, &c. since the age of 16: had never menstruated; had been married for twelve months, and complained of an obstruction of the vagina, which she was anxious to have removed.

"On inspection, the external orifice of the vagina was found completely closed by a thick, firm, muscular-looking substance, continuous with the inner margin of the labia, and adhering to the pubes below and around the urethra, so as to leave not the slightest vestige of an opening. It had neither the feel nor appearance of a membrane, but was granular on the surface; and when the labia were freely separated, bands of muscular-looking fibres were seen passing to it. The little finger could, by pushing this obstacle before it, be introduced about half an inch into the vagina, which was excessively small and contracted. The external parts were well formed, and the orifice of the urethra was natural, but the clitoris was unusually small. There was no swelling in the hypogastrium, the bladder and rectum were free of obstruction, the mammæ were plump and well formed, and the sexual appetite was not deficient.

From her great anxiety to have this malformation destroyed by an operation, I agreed to perform it on the afternoon of the day on which she first consulted me. Having placed her in the lithotomy position, I commenced, with the assistance of my friend, Dr. Weir, by making an incision, with a bistoury an inch and a half in length, in a perpendicular direction, through the centre of the fleshy-looking obstruction. I then thrust forward a sharp-pointed, narrow bistoury, for more than an inch opposite the orifice, into the vagina, directing the point rather downwards, with the blunt edge to the pubes, to avoid wounding the urethra. I found, however, from the great thickness of the substance, and the uncertainty regarding the position of the parts in the track of the vagina, that a continuance of this mode of procedure was neither safe nor justifiable. I could not succeed in forcing a probe or a director through the remaining portion; I therefore divided and removed, by means of the forceps and scissors, several layers of it. By keeping a catheter in the urethra as a guide, and by occasionally introducing the finger into the rectum, I was successful in clearing a passage fully two inches in depth. There was still, however, an obstructing portion, beyond which there appeared to be a free cavity. Through this a pair of dissecting scissors were passed, and the remainder of the obstruction divided by introducing a director into the opening thus made, getting it behind the upper part of the obstruction, and forcing it nearer the external orifice. The finger was then passed into the upper part of the vagina, which was lubricated apparently by mucus, but no uterus could be detected. A small piece of soft sponge, through which a strong thread was passed, was inserted, and a few strips of lint were placed between the external lips of the passage."

The patient could not be persuaded to remain in the house after the operation. On the 8th she was exposed to a snow-storm, and in the evening she was drunk, and committed other irregularities. Peritonitis succeeded, baffled all treatment, and proved fatal on the morning of the 12th, two hundred and three hours after the operation.

On examination there was found peritoneal inflammation. The ovaries were well formed and large—the fallopian tubes each one inch and a quarter in length, their fimbriated extremities being perfect. There was no uterus. In its normal situation was a portion of condensed cellular substance, about the size of a filbert, more than an inch distant from the uterine extremities of the fallopian tubes, and but loosely attached to the peritoneum. When the finger was introduced into the vagina, the cavity at its upper part was ascertained to be formed by the peritoneum, between which and the upper boundary of the obstruction was a free space, capable of containing a small lime. The vagina slit open presented an ash grey appearance. The urethra, bladder, rectum, and peritoneum were uninjured.

"The obstruction in this case was evidently congenital, and differed, both in situation and appearance, from that cohesion of the parts which is sometimes produced by neglected ulceration. An obstruction from the latter cause may occur both in children and adults; it is, however, rarely confined to the vagina, but more generally involves the external parts, as the nymphæ and labia. The most extensive case of this kind which I recollect to have met with was in a prisoner in the Bridewell of this city in 1817, during the short period of my attendance as surgeon to that establishment. From a long continued gonorrhœa and want of cleanliness, the lining membrane of the labia, and the inner surface of the nates around the anus, became excoriated and ulcerated, and adhesion by granulation took place through their whole extent, leaving only two small openings for the escape of the urine and feces. The adhesions were divided, and the parts restored to their natural state.

We are told that when the uterus is altogether wanting, or of a small size, the external parts are but imperfectly developed, and the vagina is either very short, or altogether obliterated. In this case, all the external parts were well formed and entire, and the mammæ were fully developed, yet no uterus existed—this circumstance is, however, easily explained, when we find that the ovaries were present. These organs are known to be more intimately connected with the growth of the external parts of the female than the uterus, and when they are absent, the marks of puberty are rarely if ever, exhibited."

---

### II.

CASE OF EMPYEMA—OPERATION SUCCESSFULLY PERFORMED. By WILLM. BRIGHAM, Esq. Surgeon, Manchester.

A YOUNG gentleman, 18 years of age, having generally enjoyed good health, but subject to slight cough, which he had had from infancy, was taken ill on the 15th May, 1830. He complained of severe and continued pain in the left side, in the back and left shoulder, with slight hemoptysis. These symptoms occurred after violent exercise.

When I was called to him, his pulse was fluttering, quick, and tremulous—respiration hurried—countenance suffused and anxious, and extremities cold. He appeared in a state of collapse. The pain was referred to the left side, but more particularly to the back and loins. He was directed fomentations—pediluvium—calomel—Dover's powder, and salines.

May 16th, 9, a.m. Had passed a restless night. The pains in the side and back much more severe—pulse 100 and full—skin hot and dry—breathing very difficult—the pain more fixed, and still referred to *the back*—tongue dry and furred—urine high-coloured and scanty—the bowels had been very freely moved. V.S. to syncope.

8, p.m. Relieved. Leeches to the side—blisters to the sternum.

17th. Had slept a few hours—leech-bites bleeding all night. Pains not so acute—breathing much relieved.

18th. Symptoms abated—pains relieved—breathing with difficulty.

From this period, for several days, there was but little pain; he had a constant, harassing cough, with much expectoration of viscid mucus, and, from the commencement of the attack, was unable to lie down.

June 1st. The breathing became gradually more distressing, and the patient required daily a more elevated position of the body. He lay on the bed, in a half-bent posture, with the head and shoulders leaning over his left side, endeavouring constantly to obtain a fixed point for support. About this time, the pulsations of the heart could be felt distinctly all over the right side of the chest, but more especially towards the point corresponding to the cartilages of the fifth and sixth ribs, and over the epigastrium. On the left side, the pulsations were entirely wanting—no beat could be felt. He was always reclined on his left side, supported by pillows, finding relief from moderate pressure applied to the same side.

June 9th. On this day Dr. Lyon was requested to see the patient. There was now great difficulty of breathing—the cough constantly troublesome—copious expectoration of frothy mucus—progressive emaciation—disturbed sleep—night sweats. Heart beating forcibly on the right side of the sternum, and not at all on the left—pulse 132—no resonance on percussion of the left side of the chest—œdema of the lower extremities—bowels rather loose—fæces generally dark or clay-coloured—urine turbid, high-coloured, and depositing a lateritious sediment—appetite capricious—spirits drooping.

12th. Aphthæ appearing all over the mouth and fauces—pulse 120.

13th. Aphthæ extending rapidly, occasioning much distress—cough incessant—great languor—strength daily declining—pulse 114—more generous diet allowed, with wine.

18th. Aphthæ subsiding—tongue cleaner.

Notwithstanding this improvement, all the symptoms occasioned by accumulation of fluid in the left cavity of the chest still continued. An operation, therefore, to remove this pressure now became a very important consideration.

The unpromising appearance of the constitutional symptoms, and the uncertainty of the extent of organic disease which might exist in the lungs, were reasons for hesitating to proceed at once to an operation, which seemed, nevertheless, to afford the only chance of recovery. Yet, the strength and spirits of the patient having partially rallied—the aphthæ subsided, the sputa being chiefly mucous—the voice clear and strong, and the patient anxious to submit to any means which might be recommended, it was determined to take the earliest opportunity for performing the operation of *paracentesis thoracis*.

About this period, the integuments over the middle of the 5th intercostal space were protruded, and formed a small fluctuating tumour; and, two days later, a similar prominence was observed below the left clavicle. The left side of the thorax was also ascertained to measure four inches more than the right.

*Operation.* On the 26th, the integuments were divided with a lancet to the extent of two inches, in the situation of the prominence before mentioned, nearly corresponding to the apex of the heart when in its natural position. A small trocar was then carefully introduced, and three pints of clear pus passed through the canula. The opening was then closed for a few minutes, to allow the patient to rally, as he seemed much exhausted; then three pints more were discharged. There was no difficulty in preventing the passage of air into the chest, nor any spasmodic action of the muscles of respiration. The canula was now removed; the wound closed, and a broad flannel roller passed round the chest.

After the operation, the patient, though much exhausted and faint, was evidently very much relieved and composed. In two hours a powerful opiate was given, which procured some sound sleep.

27th. Passed a good night, breathing with comparative ease—pulse 110. The wound had closed, and on separating the edges and introducing a probe, a pint of pus passed off. The wound was afterwards kept open by small pieces of lint for a few days, when the parts seemed no longer disposed to close.

29th. Respiration decidedly more free, and the patient could lie on his left side, from which pus continued to ooze in considerable quantity. The motions of the heart were still felt on the right side only; and there were no indications of the least respiration being performed by the left lung.

From this period his improvement was progressive, the aphthæ and dyspnœa ceased, the night sweats also gradually abated.

For the first three weeks the discharge was very profuse, and during the first week a pint of pus oozed freely from the wound daily.

In about a month he was able to change his bedroom, and soon afterwards took gentle exercise.

The heart continued to beat exclusively on the right side, the left seemed perfectly dense without the least resonnance on percussion.

August 9th. The patient went to the sea-coast and remained there a month with improvement in every respect.

The discharge continued gradually to decrease, except when he had catarrh or febrile symptoms, when it always became more profuse and more serous. This change was so remarkable that his mother could tell by the appearance of the discharges whether he had caught cold, before he began to complain of illness.

June 28th, 1831. The health has been gradually improving, he has gained flesh; appetite and digestion good; lives well and takes exercise in all weathers.

About five months after the operation, the left side of the chest was flattened, much contracted, and measured 1¼ inch less in comparison than the right.

June 28th. The left side of the chest is increased in circumference ¾ of an inch. The right side on percussion sounds quite clear—the left quite dull in every part. The pulsations of the heart are strong and full, still on the right side, but more inclined to the centre of the chest. The left side remains without any beat perceptible in the natural situation of the heart.

Oct. 27th. Slight fever, catarrh, with great tenderness about the edges of the wound, discharge rather checked from obstruction over the orifice.

Fomentations—cataplasms, &c. afforded much relief. The following morning the discharge as profuse as usual.

Dec. 10th. Progressive improvement. The discharge decreases slowly.

23d. Although 18 months have elapsed since the operation, and no untoward symptoms having arisen since that time, still the discharge from the chest continues profuse, and at times, on coughing or sneezing, is jetted out with *considerable force and in large quantities.*

April 6th, 1832. There is no further tenderness or uneasiness at the side. The discharge has been gradually decreasing for the last two months, and is much changed in its character, being quite serous.

May 15th. The discharge has entirely ceased, and the *opening closed.* No unpleasant symptoms occurring at the time. The patient is gaining strength and flesh rapidly, following his usual habits, and in perfect health.

Aug. 2d. Continues well. It is worthy of remark, that by an accurate measurement of the chest at this time, the left side is only ½ an inch smaller in circumference than the right.

---

III.

Wound of the Heart—Patient survived a Month.

Jan. 29th. A man, aged 40, attempted to commit suicide by plunging a large kitchen knife into his chest. It entered between the 5th and 6th left ribs, a few lines from the sternum. He fell down on the spot, and was carried insensible to the Hôtel Dieu. When the wound was examined, it was manifest that the cavity of the chest had been opened and probably also that the lungs had been injured, as the air entered and escaped freely during the act of breathing; adhesive straps were applied. Soon afterwards symptoms of great collapse came on; the pulsations were so feeble, as to be with difficulty felt at all; but their rhythm was regular. The patient every now and then groaned deeply, and could not be roused to answer questions. These symptoms were deemed to be the effect of a nervous shock, rather than of inward hæmorrhage; little blood had escaped outwardly from the wound; but it was uncertain whether it might have flowed into the chest.

The auscultation and percussion of the thorax were adverse to this supposition; for the præcordial region when struck gave out a clear sound; and the movements of the heart, although feeble, were regularly natural. A weak respiratory murmur was audible on both sides of the chest. The indication was to cautiously support the drooping powers of life—the patient was enveloped in warm blankets, and gentle restoratives were administered; on reviving, he vomited several times, coughed frequently, and expectorated a little mucus, without any admixture of blood. On the following day symptoms of reaction having come on, he was twice bled and purged; on the 31st he was again bled, with great relief to all his pains; the expectoration became more copious; yet there was no râle on auscultation. He continued to mend, and at the end of the week he thought that he was able to leave his bed; but upon attempting to rise, he was seized with dyspnœa, palpitations, and extreme anxiety; and now he was unable to lie on the left side.

By proper treatment these symptoms were relieved; but they again returned in all their violence, some days after. He was bled largely with benefit for the time. The severe pungent pains at the extremity of the ensiform cartilage caused much distress; and to these were added occasional delirium, sweatings, œdema of the limbs, diarrhœa, and dyspnœa; the lips began to assume a purplish hue. The diagnosis of this period was, that pleuritic effusion had taken place. On the 22d day from the receipt of the injury, the patient was seized with violent shiverings, which returned regularly at the same hour of midnight several times; the tongue was covered with a brown crust, the lips were parched, the face became haggard and hippocratic, and the breathing more and more laborious. He died on the 27th February.

*Dissection.* Two pints of a bloody serum in the cavity of the right pleura; texture of the right lung and pleura nearly healthy. On the anterior surface of the pericardium, opposite to the seventh intercostal space, was observed a transverse slit, which nearly corresponded to, but was somewhat lower down than the external wound, which was now found to have passed through, immediately exterior to the internal mammary blood-vessels. On introducing a probe through the opening in the pericardium, it was obstructed by the adhesions which had been formed between this membrane and the heart; these were however most numerous and firm, posteriorly and inferiorly; where they did not exist, a layer of fibrine had been formed; but there was no serum or purulent fluid in the bag. Opposite to the slit of the pericardium a transverse wound of the anterior surface of the right ventricle was to be seen; it was about five lines in length, and half a line in depth; its situation was at a finger's breadth from the septum ventriculorum; the edges were whitish, and were slightly coherent. Around the pulmonary artery, the apex, and the posterior surface of the heart, false membranes had been thrown out; and at these parts the investing serous membrane was deeply red and inflamed. The right proper auricle was invested with a grey coloured, and uneven layer of fibrine, which adhered to the adjacent parts; the right sinus venosus was highly injected, and the serous membrane could be easily torn from the cellular web underneath, which was slightly infiltrated with serum. All the cavities of the heart were dilated.

*Reflections.* The preceding case shews us that wounds of the heart are not necessarily, and certainly not in all cases quickly fatal. The circumstance of the wound being transverse, accounts for the very imperfect union of its lips. The great collapse, the coldness of the extremities, the smallness of the pulse, and the dyspnœa, are not pathognomonic of such an injury; they equally attend wounds of the diaphragm, and of any large internal vessels. The symptoms of pericarditis during life were not very distinct; the frequent syncopes, the palpitations, the irregularity of the pulse being either absent, or only imperfectly manifested. In the present, as in some other similar cases, which have been detailed by authors, the pain was felt chiefly at the xyphoid cartilage and radiated to the right and left sides in the direction of the pillars of the diaphragm.—*Journ. Hebdom.* No. 97.

### IV.

### New Theory of the Sounds of the Heart.

Dr. Rouanet, in an ingenious thesis, explains the phenomena of the two sounds in the following manner. The first, or dull sound, is produced by the shock, or impulse of the tricuspid and mitral valves against the auriculo-ventricular orifices, and this shock is caused by the pressure of the blood on the valves during the contraction of the ventricles. The second or clear sound arises from the succussion of the blood in the distended aorta and pulmonary artery, backwards upon the semilunar valves, during the dilatation of the ventricles; the author considering that at this moment, there is a tendency to a vacuum in the ventricle, and that therefore there must be a strong impetus, or rushing back of the blood upon the closed semilunar valves.

The first sound is heard at the commencement of the ventricular contraction; hence the idea that the sound was owing to the contraction of these cavities; and as the sound is momentary, it has been believed that the systole of the ventricles is also of the same brief duration. The cause of its being duller than the second sound is, that the tricuspid and mitral valves are larger, and that the parietes of the auriculo-ventricular apertures are thicker than the semilunar valves and the coats of the aorta and pulmonary artery.

The pulse is not quite synchronous with the first sound, but follows immediately after it. To explain the cause of this, we must remember that the tricuspid and mitral valves close at the very commencement of the ventricular systole, whereas the blood is not entirely expelled till the contraction is over. Such, however, is the rapidity of the contraction, that the second sound is heard almost immediately after the first. The second sound is more clear and sharp than the other, because the valves, by the shock of which it is produced, are smaller and thinner, and because the parietes which transmit the sound are more sonorous than in the former case.—*Ibid.*

### V.

### Reclamation of M. Pigeaux, in which he claims the Merit of discovering the true Theory of the Sounds of the Heart.

M. Pigeaux asserts that the opinions which Dr. Hope has published, on the causes of the double sound of the heart, are neither the most novel nor the most plausible of those which have been advanced, and that France may still claim the honour of having given the most satisfactory explanation of the phenomena. The four following are the propositions in which he unfolds his own views, claiming for them the merit of priority of announcement.

1. The sounds of the heart are referable to the shock of the blood against the parietes of the ventricles and of the great vessels, and not to the contraction of the ventricles and auricles.

2. The clear sound appertains to the ventricles.

3. The impulse of the apex of the heart against the ribs is not synchronous with the pulse of the arteries.

4. The silence or repose of the heart takes place after the contraction of the ventricles, and follows immediately the clear sound.—*Archiv. Général, July,* 1832.

---

### VI.

### On Spontaneous Ruptures of the Heart, &c.

When an ordinary muscle becomes ruptured, the rupture takes place almost always in a transverse direction ; in the heart, on the contrary, a longitudinal rupture of the fibres is much more common than a transverse one. In forty-four cases, out of 54 of fatal rupture of the heart, the perforation was found in the left ventricle ; in eight, the right ventricle was the seat of it ; and in the other two, it had taken place between the two auricles. The thickest parts of the ventricles were ruptured in 45 of the cases ; and the interventricular grooves in seven cases only. The ruptured cavities were dilated and attenuated in seven cases ; and appeared to be normal in structure in the remainder, except in three of them, where the parietes seemed to be partially or locally rendered thinner in substance. If to the 13 cases of general ramollissement of the affected organ, be added 27 cases in which morbid alterations of structure, varying, indeed, as to degree, but all tending to a solution of continuity, were observed, and nine cases in which the special alteration is not mentioned, we shall find that the opinion of M. Rostan, that almost all cases of rupture of the heart are spontaneous, is far from being correct. Equally remote from truth is the assertion of M. Bland, that the almost exclusive cause of this fatal accident, is a gelatiniform softening of the heart's substance : there are many other morbid changes besides this ramollissement. To recur to the comparative frequency of transverse and longitudinal rupture of this organ, it appears that 13 were of the former description and 36 of the latter, or in a direction parallel to the muscular fibres.

The difference of the direction is probably attributable to the nature of the preceding morbid change of the texture, and to the manner in which it is generated ; hitherto it has been a very common mistake to regard transverse rupture of the heart as quite analogous in its etiology to a transverse rupture of other muscles, such as the gastrocnemii ; but this is erroneous, for, in the one case, a morbid change always, and, in the other, seldom or never, precedes the accident. In ruptures of the heart, as in aneurisms of the vessels, the parts have almost always been found diseased in the first instance ; and the diseased depositions are either of an atheromatous, steatomatous, tubercular or cancerous nature. In some cases the rupture is incomplete, affecting only a certain set, or stratum, of the muscular fibres, and not penetrating through the entire thickness of the parietes. It is not improbable that, in many such cases of internal incomplete rupture, the membrane which lines the cavities of the heart may be burst at first, but afterwards may be renewed and repaired, so that the case may be regarded, on the autopsy, as one of simple general dilatation of the cavity, and not as originally a rupture of its parietes. If this idea prove correct, it will follow, that the number of internal ruptures of the heart is nearly equal to that of the external ones.

M. Cruveilhier says that a muscular apoplexy of the substance of the heart is one of the principal causes of rupture; but M. Pigeaux thinks that the morbid change, so designated, ought to be viewed rather as subsequent to, and an effect of, than the cause of lacerated fibres; the rupture, indeed, may not have been complete before the sanguineous effusion and infiltration, but probably takes place only in some of the muscular fasciculi. Another argument against the belief, that ruptures of the heart are analogous to ruptures of other muscles, and are caused by the force of the ventricular contractions, is, that these accidents are very rarely seen to occur in the auricles, and yet the force of their contractions is fully as great, in proportion to their thickness and strength, as those of the ventricles.

It is very probable that, when rupture of the heart does happen, it takes place rather at the moment of its dilatation than of its contraction; the shock and impulse of the blood, as it rushes in upon the softened and diseased part, operating as the immediate agent. This explanation accounts for the extreme rarity of rupture of the auricles, because, in them, ramollissement seldom occurs, and the rushing in of the blood is comparatively feeble; and we now no longer are surprised at the rupture of the ventricle being so frequently in a direction parallel with its muscular fasciculi.

Hitherto it has been too much the case to attribute dilatation of the cavities of the heart to some obstacle, or impediment, to the free flow of the blood from them: such was the opinion of Corvisart; but of late years, M. Pigeaux has called its correctness in question; and, after a most studious examination of the subject, has arrived at the following conclusions;—1, Dilatation of one or more of the cavities frequently occurs, without any contraction of their orifices. 2, Contraction of the orifices often is found, without any dilatation of the cavities. 3, The condition of the lungs has very little influence on the state of the right ventricle (this is proved by the fact stated by M. Louis, that out of 105 cases of phthisis, dilatation of the right side of the heart existed only in three), and, therefore, â fortiori, the state of the left ventricle is still less influenced by that of the right one. 4, The impulsion of the blood is the efficient cause of dilatation. 5, The mechanism of the formation of cardiac aneurisms is quite similar to that of the formation of arterial aneurisms. 6, The force of the ascent in the veins, and ingress of the blood into the auricles (a force which exerts a pressure equal to that of several atmospheres), compensates for the absence of, and is nearly equivalent in force to, the strong impulsion of the blood into the ventricles. 7, The weight of the blood which remains behind in any cavity, after its systole, may frequently co-operate, with the impulsion of it to give rise to dilatation. 8, A change in the firmness of the parietes of the heart is by far the most common disposing cause of dilatation. 9, Any mechanical impediment to the free egress and course of the blood, must favor and aggravate the effects of its impulsion.—*Journ. Hebdom.* 104, and *Révue Med. Sept.* 1832.

---

### VII.

### CURIOUS MALFORMATION AND ORGANIC DISEASE OF THE HEART.

A FEMALE, aged 25, was attacked with typhus fever in 1824. On the seventh day of the disease, she suffered much from violent palpitations, which lasted for several days, then ceased for a time, and again returned at intervals; distressing vomitings existed at the same time, and in the progress of the fever, delirium, cough, miliaria, gouty pains and œdema of the feet supervened. After the lapse of five months, during the greater part of which time she had been so afflicted with dyspnœa and palpitations that she could walk only at the slowest pace, a violent hæmoptysis came on. By depletion she recovered, and the dyspnœa and palpitations were abated; but every now and then they returned with great severity. In Feb. 1827, the catamenia were suppressed, and a general breaking up of her health ensued. She died five months after this period, and three years and a half after the attack of typhus. For some time before death, the palpitations had become most afflicting, and were always accompanied with vomiting; the voice also was lost.

On dissection, the heart was found flabby, and devoid of a right auricle; in its place was a venous canal, an inch long, and an inch and a half wide, which extended from the heart to the junction of the two venæ cavæ; the only trace of the sinus venosus was a small portion of the septum, but there was a more considerable vestige of the auricular appendage; its size was about that of a large pea. Where the venous canal was united to the heart, a circular ridge marked the place of the tricuspid valve; the opening, however, was so large, that the canal, and the upper portion of the right ventricle, formed but one continuous cavity; the lower portion of this ventricle was separated from the upper by transverse fleshy pillars, so that there was an upper and a lower cavity in the right ventricle; the pulmonary artery was considerably enlarged; the left side of the heart was of small dimensions, but of normal structure; the lungs were studded with tubercles.—*Hufeland's Journal, July,* 1831.

---

### VIII.

### Case of Pericarditis, with Remarks. By M. Roger.

A gentleman, 26 years of age, was suddenly seized with an acute pain in the præcordial region, and with dyspnœa. He was bled freely and relieved. When he entered La Charitè, four days afterwards, the following were his symptoms. He could lie on either side, without distress, but not upon his back; for the dyspnœa was always increased when he attempted to do so; the breathing was hurried, short, and much distressed—to speak caused him great uneasiness; a severe pain was felt in the region of the heart, extending to the epigastrium; there was a constant cough, but without expectoration; the chest was sonorous on percussion at every part, except over the cardiac region; the respiratory murmur was normal, save at one or two places, where a sibilant râle was heard. The pulsations of the heart were so feeble as scarcely to be felt, and when they could be it was found that they were irregular, and were not synchronous with the pulse at the wrist; the pulse was also very small, feeble, intermittent, and unsteady, sometimes rapid, and at other times not more than 65 or 70 beats. To the above symptoms was added general pyrexia. Demulcents, refrigerants, venesection, a blister over the heart, and low diet, were ordered. On the next day (20th), he was better; cough less troublesome, and the breathing easier. To continue the same medicines; to be again bled to 12 ounces, and to have an emollient enema. The symptoms continued to decline till the 23d, when the report was, that the pulse was less irregular, the cough almost gone, the pain had nearly ceased, the respiration free of all râle; the patient could lie on his back, and the fever had much subsided. On the 26th, the pulse was nearly regular, but frequent; the beats of the heart could not be felt by the hand, but were heard distinctly by the ear; their rhythm was quite normal, but, upon listening very attentively with the stethoscope over the region of the heart, a sort of creaking, or new leather sound, as if the two surfaces of the pericardium kept rubbing against each other, could be heard. On the 1st of June, almost all the symptoms had disappeared; the pulse was quite natural; the beats of the heart could be perceived both by the hand and by the ear, and the patient felt himself so well that he left the hospital.

*Remarks.* The preceding case is a good specimen of the ordinary course of pericarditis; we observe that there was an acute pain in the cardiac region, that it came on suddenly and without warning, that it was accompanied with palpitation and feeling of great oppression, with irregularity and intermittence of the pulse, which, at the same time, is often not synchronous with the systole of the heart; and that there was a dull sound over the cardiac region on percussion to a greater extent than is natural. It is not common to find all these symptoms combined in one case: and many examples are on record, where all the morbid signs of pericarditis have been noticed on dissection, while not a symptom of the disease had existed during life. Hence Andral mentions six varieties of pericarditis, according to the symptoms which it exhibits. 1, Pericarditis, in which all the symptoms are present. 2. When the pain is awanting, and the other symptoms are present. 3. When the pain and irregularity of the action of the heart and arteries are awanting, and the only symptoms are dyspnœa, and dulness on percussion. 4. When the only symptom is dyspnœa. 5, When there is no characteristic local symptom whatsoever, and there are only general or systemic symptoms of disorder of the nervous system. 6, When neither local nor general symptoms of any kind exist, and the disease is, therefore, altogether latent. The peculiar sound which we have denominated the new-leather sound, or that which we hear when a piece of new leather is pressed or rubbed, has also been recognized in cases of pleurisy. Our prognosis in pericarditis ought always to be extremely guarded, as quick alternations from better to worse often take place unexpectedly in the course of the disease, and the patient may expire at the very time when the most dangerous symptoms had vanished, and when the physician entertained just hopes of the recovery. According to M. Louis, there is, perhaps, not a case on record of pericarditis, which has been detected during life, with a fortunate issue; but to the truth of this assertion we cannot accede; and, independently of the above and many other similar cases, we may allude to the circumstance of adhesions of the pericardium to the heart being sometimes found after death, and yet the patient, who may have died of another disease, was never suspected of having at any time laboured under pericarditis. These adhesions, therefore, prove that the disease is not necessarily fatal.—*Journ. Complément. Aug.* 1832.

*Obs.*—No allusion is made by the author to the presence or absence of the "bruit de soufflet" in the preceding case. The practice of Dr. Latham, and of many others in this country, shews that it is very generally met with in pericarditis—the rationale of its occurrence being probably the increased irritability of the heart.—ED.

---

### IX.

### IDENTITY OF MAGNETISM AND ELECTRICITY.

UNQUESTIONABLY, the most important of all discoveries relating to the voltaic pile is that made by Œrsted, that a wire, connecting the two ends of a pile in action, has the power of turning aside from its direction a magnetic needle, which is brought within a certain distance from it. The polarity, declination, and variation of the needle had been long known and intimately examined, but no satisfactory hypothesis to explain the nature of the magnetic power had ever been proposed, although such men as Gassendi, Descartes, Euler, Bernouilli, and Hansteen had laboured in the field of enquiry; hence, magnetism was always regarded as a distinct physical agent or power, and not as one merely associated, far less identical, with any of the other agents of the natural world. The brilliant discovery of Œrsted has at once dissipated all uncertainty and doubt, and has given rise to many most interesting and important researches in every country of Europe. Ampèra has shewed, that two parallel currents of electricity mutually attract and repel each other, according as the course and direction of them are similar or not. Arago discovered that the fluid of the galvanic pile has the property of decomposing the magnetic fluid, under certain circumstances, and of generating or exciting a polarity in a steel plate. Schweigger and Poggendorf contrived a most ingenious instrument to exhibit any electrical current, however feeble and inconsiderable it may be, and, with the aid of this instrument, have been able to give more precision to any enquiries on the development and influence of electricity upon chemical actions.

And, lastly, Siebeck has shewn, that an electrical current may be produced in metals, without the intervention of any liquid, and merely by the action of caloric. By thus establishing the identity of magnetism and electricity, these authors have made a great stride to the knowledge of nature's laws; and when we reflect that there is a strong presumption that electricity, caloric and light are but one agent, or power, variously modified, and that there is a closer connexion between the phenomena of electricity and those of chemical affinity and of general attraction, we have reason to think that we are on the eve of some of the grandest and most important physical discoveries.—*Ibid.*

---

### X.

### Inversio Vesicæ.

The following case is published in the first number of a contemporary periodical, to which we bid hearty welcome, and wish every success—the Liverpool Medical Gazette. The author is Dr. P. J. Murphy.

"Jane R——y, æt. 4, admitted into the county of Meath Infirmary, July 9, 1829. Her mother stated that she had been seen by a medical gentleman six hours previously, who had represented the disease under which she was suffering to be prolapsus ani, but failed in reducing it, after a tedious trial. On learning that mortification would most probably be the consequence of its non-reduction, she became alarmed, and brought the child to Mr. Nicolls of Kavan, who, having satisfied himself that it was some unusual disease, immediately brought her to the Infirmary, where she was seen by Dr. Byron, the present surgeon to the Infirmary. For examination she stood on a table, with her face towards the examiners, and our first impression certainly was that of it being a case of prolapsus recti. We prepared to reduce it in the usual manner, by placing her on the back, elevating the head, and fixing the thighs on the abdomen. Catheters were also in readiness to empty the bladder. Immediately after having thus arranged the patient, the anus and perineum were plainly discernible. A closer examination now became necessary, and the following appearances were noted down. A pyriform tumour, the size of a small hen-egg, depends from between the upper portion of the labia-pudendi, colour of a dark mahogany, the base below, the apex above; the little finger oiled and introduced per anum, communicates no motion to the tumour, nor can anything unnatural be detected. On raising the tumour towards the pubis, the vagina was seen, but the meatus urinarius could not be traced. Some congenital deformity was now suspected, but the mother's answers, which were very clear, satisfied us on that point. We now sought to ascertain if the bladder were inverted. The orifices of the ureters were looked for, but not discovered until a very slight traction of the tumour downwards rendered the inversion complete. A small silver probe was passed up each orifice, which, on being withdrawn, was followed by urine, almost devoid of either smell or colour.

Replacement. — The neck of the bladder was steadied by the thumb and forefinger of the left-hand, and the fundus having been pushed upwards by the end-of a gum elastic catheter, its re-inversion was easily effected. The catheter was retained there for a few hours by an assistant. Some tenderness of the pubic region following, attended with vomiting, leeches, warm-bath, and castor oil were prescribed, to which those symptoms quickly yielded. On the 17th of July she was discharged cured.

Observations. — That the bladder could be completely inverted, I had, until then, deemed anatomically impossible: of course, it can take place only in the female. I am not aware that there is any case on record. I certainly have not been able to consult the 'Cas Rares.' It is true, that Mason Good says something about prolapsus vesicæ into the urinary passages, under two forms. He quotes from Sauvages.

First form, a protrusion of the inner membrane, in consequence of its separating from the general substance of the bladder, visible in the meatus urinarius, of the size of a hen's egg, subdiaphanous and *filled with urine.* Sauvages' case is quoted from Noel, who met with it in a virgin, who was, from the *first,* peculiarly troubled with retention of urine, accompanied with frequent convulsive movements. The state of the tunic was proved by dissection. But this case is no ways analogous to the one I have just related. I am inclined to consider it a case of congenital malformation from the word *first,* which signifies in the above case, from birth, or perhaps it was anasarca of the submucous tissue from inflammation. It is stated to have been *filled with urine,* but, if separated from the general substance of the bladder, how could it be filled with urine unless from some opening by ulcer, or otherwise? Mortification must have been the consequence of such effusion.

The second form, he tells us, is chiefly found among women who have borne many children. The protruding cyst drops down into the urinary passage to about the length of the little finger, and is sufficiently conspicuous between the labia.* He gives a case from Solingen. Where the anterior wall of the vagina has been destroyed, and a communication formed with the bladder, an inverted bladder is by no means uncommon. I do not remember any cases of inversion where the destruction was confined to the urethra alone. Anatomically considered, inversion is more likely to take place in the young than in the aged. In the child, the shape of the bladder, both in its distended and contracted state, is pyriform, the base above, the apex below; while its axis is almost perpendicular: in the adult, its form, when distended, is oval; when contracted, a flattened triangle, its long axis oblique, anteriorly pointing to the linea alba midway between the pubis and umbilicus, posteriorly if produced will touch the extremity of the coccyx. In consequence of the non-development of the pelvis of the child, the bladder is almost entirely in the hypogastric region, subject to the action of all the abdominal muscles, particularly that of the pyramidales and the lower divisions of the recti, from which it is separated only by a thin fascia. In the adult it lies altogether in the pelvic region, unless when distended, and, as it is only in the contracted state that inversion can take place, it is almost entirely withdrawn from the influence of the above-mentioned muscles; moreover, in the child the ligaments of the bladder are weak and yielding, the urethra absolutely shorter, and there is scarcely any angle formed between the bladder and urethra, which must favour inversion as much at this period of life, as the contrary form tends to prevent it at a more advanced time. Inversio vesicæ is not analogous to the inversion of any other part of the human body. It resembles that of the uterus more than that of any other organ. But the cause of the latter being inverted is easily understood, namely, a forcible separation of the placenta, polypus, &c.; and, did the same causes exist in the bladder, no doubt we should have inversion very common: but in the case I have just related the surface was minutely examined for either polypus or an adhering calculus, but its healthy appearance was sufficient testimony that none of those causes existed. The inversio uteri in the unimpregnated state, has been

* "Dr. Good seems rather to describe prolapsus vesicæ than inversio, but as he places both inversio and prolapsus uteri under the genus "Œdoptosis," there is some difficulty in understanding exactly what disease he intended to describe."

denied by some, and, no doubt, if in this state it had not been subject to polypus, the opinion would have been correct; but I have seen a polypus completely invert the uterus, although unimpregnated, and Dr. Byron mentioned to me another which occurred in his private practice. Could the inversion have taken place in the following manner? In its contracted state the internal surface of the fundus might have easily fallen down on the opening of the urethra, so as to form something like a partial inversion. In this case its serous surface would have formed a funnel, the concavity looking upwards, if a portion of intestine filled this cavity, a sudden exertion of the abdominal muscles might have completed the inversion."

---

### XI.

### On the various Sorts of permanent Flexion of the Fingers, and of their diagnosis.

1. The first that we shall mention, is that which is caused by a contraction, or puckering of the palmar aponeurosis. Dupuytren has the merit of having first distinctly pointed out the true nature of this affection, and of the treatment which it requires; namely, the section of this strong aponeurosis.

2. A permanent flexion of one or more fingers may be the result of some disease or malformation of their joints.

*Case.* A young man had white-swelling of the ankle-joint. The little finger of the left hand had been permanently contracted in the form of an arch, from his infancy; the phalanges did not move, the one upon the other; but there was free motion between the finger and the metacarpal bone. No hard cord or projection was felt in the palm at the root of the little finger, when this was forcibly bent backwards, or extended. In short, the permanent flexion in this case arose from an anchylosis of the phalanges. In some cases it is produced by a synovial cyst forming over one of the joints; this mishap is not very unfrequent among tailors; in others by an irregularity, or unevenness of the articular surfaces of the phalanges. We observe such cases among tailors, seamstresses, and especially among knitters. In them a contraction of the little finger is not uncommon, and it proceeds from some abnormal change in one or other of the joints.

*Case.* A young female, who worked in the manufactory of lace, applied to Dupuytren, to relieve her of a contraction of the four fingers of both hands upon the palms; they were bent so as to form nearly a quadrant of a circle. The phalango-metacarpal joints were quite free; when the first phalanx was strongly bent backwards, no tense tendon or cord was to be felt.

3. A third variety of the affection is, when it is caused by a division of the tendons of the extensor muscles. A person applied to Dupuytren under the following circumstances. The two last fingers were constantly bent upon the palm of the hand; yet on extension, they could be readily made even with the others; but no sooner was the extension withdrawn, than the fingers again became bent. While extended, no hard cord was to be felt on the palmar, or on the palmar surface of the finger; and moreover, each joint might be easily moved. The patient had received a sabre cut on the back of the hand, and the tendons of the extensors had been divided. Nothing could be done for him.

4. A puckered cicatrix of the skin will sometimes cause flexion of the corresponding finger or fingers; hence the importance of keeping the hand extended during the healing of any wound, sore or burn.

5. A lesion, or injury of the tendons of the flexors may have the same effect. This variety is apt to be confounded with, and mistaken for the first, or that which results from a contraction of the palmar aponeurosis: but in the latter case, the finger cannot be made to yield to any extension, and the tense cord, which was not to be felt before, is now readily recognized during the effort.

When, on the contrary, the malady has been caused by an injury of the tendons, the projection, which was very distinct while the finger is bent, becomes much less so, or altogether disappears when it is forcibly stretched. An example of this variety is detailed: a tumour had been excised from the finger, and during the operation the sheath of the tendon had been opened.

6. This last species of permanent flexion of the fingers is that which arises from the loss or wasting of the substance of the flexor muscles. This may be destroyed by a gun-shot wound of the fore-arm, or by laceration, from any violence. In such cases there is always more or less paralysis, in consequence of the injury done to some of the nerves. The different joints of the fingers remain quite flexible; but when they are forcibly extended, pain is felt at the cicatrix of the wound.

It must be altogether unnecessary to state that these different varieties of the above malady require different modes of treatment, according to the nature of the exciting cause.—*Journ. Comp. Sept.* 1832.

---

### XII.

### CASES OF DIFFUSED INFLAMMATION AFTER BLEEDING IN THE FOOT.

*Case* 1. A young woman was ordered to be bled in the foot, at the Hôtel Dieu, in consequence of amenorrhœa.

The operator made several attempts to open a vein at the external ankle, but failed; the saphena vein was afterwards opened. In a few days appearances of spreading inflammation of the skin and cellular substance shewed themselves, and afterwards great tumefaction, pain, and general disturbance. Leeches and poultices were applied; but the disease became worse and worse, and the patient was delirious, and suffered much from vomiting, purging, and extreme sensibility of the abdomen. She was brought into the surgical wards on the 20th day from the commencement of the symptoms. Bleeding from the arm was immediately ordered, and two long incisions were made along the foot; much fetid and sanious matter flowed out; and a third incision was made at the upper and inner part of the leg, where a large purulent collection had formed. Thirty leeches were applied to the epigastrium. Gangrene of the back of the foot came on; and an intense inflammation had invaded the upper part of the thigh. Forty leeches were immediately applied to the part. A long incision was made on the outer side of the thigh. It was not till the end of the sixth week that the inflammation of the limb had entirely ceased; the tendons of the extensors of the toes sloughed off. In two weeks more most of the incisions had healed, and the large exposed surface of the foot was healthily granulating. In six weeks more, she was able to leave the hospital, but the limb as yet was little able to execute any movements.

*Case* 2. A woman chanced to prick her finger with a thorn; inflammation of the arm speedily came on, and also symptoms of extreme gastro-enteritic irritation. When she was brought to the hospital, three weeks after the accident, she could give no account of herself; the whole limb was enormously swollen, and all the signs of a very aggravated typhus were present. She was immediately bled, and the part leeched and poulticed. Five days after this date, she began to complain of pain in the knee: when it was examined, a distinct fluctuation could be felt; free incisions were made at several parts; but as the inflammation abated in the arm, it increased in the leg, and all the disturbance of head and abdomen was renewed; she died on the ninth day after entering the hospital.

*Remarks.* The very formidable disease, which is illustrated by the two preceding cases, sometimes attacks the head, and is then not unfrequently fatal. It is of importance to observe that when the cellular tissue between the pericranium and the occipito-frontalis aponeurosis is the seat of the disease, and when it has gone on to suppuration, so as to detach the integuments from the subjacent parts, these integuments are not so liable to fall into a state of sloughing as the integuments of the extremities, and of other parts; and why is this? Because the blood-vessels, namely, the branches of the frontal, temporal, and occipital arteries are so very intimately connected with the skin of the head; whereas in the arm and leg, &c. the nutritious arteries of the skin are mostly small vessels which pass to it from the subjacent textures, and through the subcutaneous cellular substance; hence, when this web is destroyed by suppuration, the permeating arteries and veins are no doubt involved at the same time: in the case of the scalp it is otherwise; for its vessels directly appertain to it, and not to the pericranium and bone. It is of much consequence to ascertain whether the pus has not injured the pericranium; for when this membrane is destroyed, recovery is very rare; and when it is not, we may very generally entertain better hopes. Even in the worst cases, the scalp seldom becomes lifeless or sloughs; and for the reason of the peculiar arrangement of the blood-vessels above-mentioned. M. Dupuytren has seen in his vast practice only two cases of actual gangrene of the scalp.—*Ibid.*

---

### XIII.

### Two Cases of Hernia, in which the Operation was performed, although there were no External Swellings.

A man, aged 40, had an inguinal rupture on each side; the one of 12 years, the other of three years standing. After a violent effort, the former, or that on the left side became suddenly enlarged and very painful: it was after some time reduced, but the symptoms of strangulation continued, and on the 5th day he was carried to the Hôtel Dieu. The abdomen was very tender, and he suffered much from hiccup, fæcal vomiting, and pains of the bowels; there was no outward appearance of any herniary tumour; the abdominal ring when examined, were found more open than usual; there was a fulness in the right groin on the following day, and when the part was pressed, it was very painful. Dupuytren cut down cautiously to ascertain the state of things; he opened a small smooth-lined sac, from which a good deal of serum issued; but there was no trace of gut, or of omentum: when the finger was passed up into the abdomen, several adhesions could be distinctly felt. Dupuytren immediately operated upon the other side; the second incision opened a small sac, which contained a fatty mass, mistaken at first for omentum; but by making the patient cough, a layer was observed beneath it, and when this layer was divided, much bloody serum flowed out. Dupuytren, was now satisfied that the strangulation existed upon this side, in consequence of the difference of the serum from that which was noticed on the other side. In the sac a small portion of inflamed omentum was found, and on introducing the finger up the ring, a circular bridle could be felt; the sac was gently pulled outwards, and along with it a small portion of gut; a probe bistoury was cautiously introduced, and this bridle was divided upwards and outwards. The patient ultimately recovered.

*Case* 2. A man was brought to the Hôtel Dieu in an apparently dying state; he was hiccuping, vomiting, with cold extremities and tumid painful belly. It was with difficulty that he could tell that he had long suffered from a double rupture; but there was no outward sign of any swelling. The question was, whether the case was one of peritonitis, or of concealed hernia. Dupuytren ordered the patient to be bled, and to have repeated enemata given. Several copious alvine evacuations were procured, the vomiting ceased, but the hiccup continued. On the following day he was considerably easier and more collected, so as to be able to give some account of the progress of his malady. He had laboured under a rupture on each side for eleven years, they had never much incommoded him till the day before he entered the hospital; but suddenly after an exertion they became painful and irreducible for some time; both however were speedily returned by the taxis; yet most of the symptoms of strangulation continued. An incision was made over the right inguinal ring, and a smooth sac, which was recognized as a herniary one, was laid open; a grooved sound was passed upwards, and bloody serum then flowed out; the sac was drawn a little out, and its neck was found to be puckered, and drawn together like a cicatrix. It was divided, and the finger then might be easily carried inwards. The patient quickly recovered.

*Remarks.* In such cases, as the above, are there any signs by which the surgeon can determine, after the reduction of a hernia, whether strangulation continues or not within the abdomen? The abdominal ring should be carefully examined, to ascertain whether it be large and open, whether any pain is felt there, when the finger is firmly pressed upwards and outwards.—*Ibid.*

---

### XIV.

### PERICARDITIS.*

ALTHOUGH the utility of the stethoscope is now acknowledged, even by its enemies, yet we fear that many of its advocates are not sufficiently cautious in their diagnoses founded on auscultation. We are less confident now, after more than ten years' study of the stethoscope, than we were at the end of the first two years. Thus, for instance, we sometimes find it extremely difficult, we had almost said impossible, to distinguish nervous palpitation of the heart from hypertrophy of the same organ. Yet the tyro in auscultation will pronounce a decision in half a minute!

*Case.* A young boy, seven or eight years of age, presented himself to Dr. Thwaites, at the South-Eastern Dispensary, complaining of constant oppression at the chest and short cough, greatly increased on using any exertion, and causing suffocation. His health had gradually declined for three years past. He had had rheumatic fever, after which palpitation of the heart came on, with the embarrassment of respiration.

"Upon examination, the left side was considered somewhat more distended than the right; the action of the heart was distinctly visible at some distance; countenance anxious, complexion good, but lips approaching to a purple colour; pulse 100, synchronous with the heart's action and regular; pulsation of the carotid arteries greater than natural, tongue clean, bowels rather costive, appetite good, sleeps tolerably well; does not complain of any affection of his head, *bruit de soufflet* heard distinctly over the whole præcordial region, and on the corresponding position of the vertebræ posteriorly. *Fremissement* could be felt on the application of the hand to any part of the left side."

We need not detail the treatment by venesection, leeches, blisters, colchicum, &c. The Winter interrupted a regular observance of the symptoms; but when the little patient returned, in the succeeding Spring, the disease had evidently progressed. The left side was more bulged out—the blueness of lips increased—pulse 110—great anxiety—*bruit de soufflet* and *fremissement* augmented rather than diminished—a musical sound very distinctly heard, in three notes, commencing in a grave, and rising in the scale to a sharp thrilling tone. The case was sent to an eminent stethoscopist for opinion, and the following was delivered.

"The case is one of narrowing of the left auriculo-ventricular opening, with regurgitation into the left auricle. The signs are *bruit de soufflet* under the left mamma, which is produced by the passing of the blood through the narrowed opening; and in the same situation very distinct *fremissement* is felt when the hand is pressed flat against the side.

---

* Dublin Journal, No. V. November, 1832.

My reason for saying that there is regurgitation, is, that there is very distinct *bruit de soufflet* heard in the back on applying the stethoscope over the fourth dorsal vertebra, which I believe is produced by the passage backwards into the left auricle of some portion of the blood first sent forwards into the ventricle. I should be inclined to say that there is not hypertrophy of the left ventricle, but I cannot be positive, and it is a matter of very minor importance in the case."

At this period the hydro-sulphuret of ammonia was prescribed, in doses of four drops thrice a day, gradually increased—and with astonishing benefit, even in a week.

"His countenance was quite altered, as the great anxiety which had been so strongly depicted on it was much lessened, and when spoken to he smiled, and said that he 'liked to take the medicine, as it made him so much better.' His mother stated that he had not been taking the medicine long ere the oppression was so much relieved, that he began to talk and play as he was wont to do before he got so bad; and that she came to have the medicine renewed, as he had not got any thing which gave him so much relief. The *frequency* of his pulse was not in the least relieved, but the *violence of the heart's action* was most perceptibly so. The *bruit de soufflet* and the *fremissement* both continued, but the musical *bruit* was gone, and could never be heard again. But at this period I left town rather suddenly, and the patient came under Dr. Benson's care. Without having learned from me the treatment I was pursuing, he, for the same reason which induced me, also prescribed the hydro-sulphuret of ammonia in doses of six drops, three times in the day."

But all would not do. The essential physical signs diminished not—dropsy took place, and death closed the scene.

*Dissection.* "Upon raising the sternum, the heart presented itself greatly enlarged in its general appearance, and occupying the whole of the left inferior and anterior part of the thorax: the left lung was much compressed and reduced in volume, and the inferior part of the right lung was considerably displaced. Upon cutting into their structure, they did not exhibit any appearance of disease.

The heart was next removed from its situation for the purpose of obtaining a more accurate examination of its state. The sac of the pericardium was not to be found, the membrane itself having formed so intimate an adhesion to the whole surface of the heart and to the roots of the great vessels, that it could only be separated from them by the practised scalpel of a demonstrator, the adhesion having all the appearance of being of very long standing, except at the posterior and superior part of the left ventricle, where it exhibited appearances of more recent inflammation. When we cut into the right ventricle it was found hypertrophied to once and a half its original thickness, of firm consistence and of a natural colour. Among the cells formed by the carneæ columnæ in the walls of the ventricle, were discovered two substances resembling small lumps of fatty substance firmly attached to the lining membrane, and when cut into presenting a small cavity containing a trace of creamy-looking fluid on its surface.* The cavity of the ventricle was dilated, and the auriculo-ventricular opening was of a size proportioned to the adjoining cavities. The auricle was also proportionally enlarged in every respect. The tricuspid valves, the pulmonary artery and its valves, and also the carneæ columnæ, were perfectly healthy.

The left ventricle was considerably thickened in substance, but in no other respect exhibiting an unhealthy appearance. The auriculo-ventricular opening of that side was not diminished in size, but, on the contrary, held a proportion to the rest of the heart. At the insertion of the chordæ tendiniæ into the floating edge of the mitral valve, there were firm depositions of a fibro-cartilaginous consistence, which

* "To the best of my recollection these bodies have been noticed before by Dr. Townsend, but *where* I cannot recal to mind."

gave to the edge of that membrane a thickened appearance and feel. The internal surface of the auricle was partially covered with a firm gelatinous-looking substance, which was internally connected with the lining membrane, so as to present a roughened appearance when scraped with the scalpel. The valves of the aorta were perfectly healthy. At its origin, and for a short distance, the aorta was considerably dilated, bearing an exact proportion to the left ventricle, but rather suddenly assuming its natural size after giving off the great superior arteries. The exact correspondence in the size of all the parts was very remarkable, as well as the absence of those organic changes which we all had so confidently anticipated."

The veil being removed, pericarditis was unequivocal. The difficulty of ascertaining this important disease has, many a time, occasioned painful reflections in our mind—for many a time have we been deceived. Several distinguished pathologists, among others Andral and Louis, have confessed the difficulty. It is our intention to introduce an extended article on this important subject, in the next, or a succeeding number of this journal. The foregoing case meantime, will not be without its use to our readers.

---

### XV.

### CUTANEOUS ERUPTION IN CHOLERA.

IN a female who had just recovered from the worst symptoms of cholera, an eruption of lenticular papulæ, of a faint red colour, and surrounded with an areola was observed on the hands and arms. It was at first attributed to the irritation of sinapisms; but as it quickly appeared over all the body, this explanation could not stand; some of the papulæ presented minute pustules on their tops; and here and there they were so dense and small as to look like an erythematous rash. The same appearances were observed in two other cases; and in both of these they were contemporaneous with a decided amendment of the cholera symptoms. Is the eruption a critical one? M. Alibert repeatedly noticed it in many cases under his care. In one case the "measley inflammation extended to the mucous membranes of the mouth, pharynx, nose, and eyes.—*Journ. Complem. Sept.* 1832.

---

### XVI.

### ST. VITUS' DANCE; TREATMENT IN FRANCE.

A YOUNG girl had all the characteristic symptoms of this very curious disease; the twitching of the muscles of the arms, hands, legs, and face, the twisting of the mouth, the constant and involuntary shaking of the head, the unsteadiness of the gait, and of any attempt to lay hold of things, and the embarrassment of the speech, sufficiently announced the disease. The symptoms always ceased during sleep; they had existed for three weeks before Dr. Manners was consulted.

She had been twice blistered, and stimulating frictions had been used. The Doctor ordered 15 leeches along the spine; and the spine to be rubbed with a liniment made of the extracts of belladonna, hyosciamus, and opium, dissolved in water. Next day the patient took a tepid-affusion bath; a foot-bath to be used every night, and the feet to be afterwards enveloped in hot poultices sprinkled with mustard!!—besides these she was to have given to her every evening, a "demi-lavement" made of valerian tea; and at bed time she took a spoonful of the following mixture:—

Distilled water of lettuce, ℥iv.
Distilled water of orange flowers, ʒii.
Concent. water of cherry laurel, gtt. x.
Acetate of morphia, gr. $\frac{1}{4}$.

The quantity of the cherry-laurel water to be gradually increased. The ordinary drink of the patient to consist of orange-leaf tea. A shower-bath was employed every morning, then this was changed for a douche bath, and the temperature of the water was gradually lowered. The stream was directed on the head and nape of the neck, while the face and chest were protected by a veil ! ! On leaving the bath the patient was well rubbed with a warm towel, and drank a cup of hot elder tea ! In 10 days we are told she was vastly better; in other 20 days she was pronounced to be cured. The patient had taken 22 baths.—*Journ. Complem. Sept.* 1832.

---

XVII.

Cancer of the Stomach found on Dissection—no Symptoms during Life.

A man, aged 51, entered La Charité on the 5th July, 1832 ; he was a tailor by trade, and had therefore much sedentary employment. In 1826 he had a very severe attack of jaundice, which lasted for two months, and was treated by emollient and diuretic medicines alone ! ! [When will many of our French brethren be taught to adopt more active therapeutics ?—Ed.] The jaundice returned each year since, but with less severity. Eight months ago, his belly began to swell, and he felt uneasiness in the right hypochondrium; the swelling gradually increased, and when he entered the hospital, the following is the report of his case. The abdomen is enormously distended ; the edge of the liver extends down as far as the umbilicus, and may be distinctly felt in the left hypochondrium ; the abdominal parietes are hard and tense, and irregularly puffed out here, and depressed there ; they are painful on pressure ; the limbs are œdematous, the complexion of a pale yellow cast, and, in short, (says our author !) all the pathognomonic symptoms of a cancerous tumour of the liver are present. The patient's strength is exhausted by a constant slow fever, and a diarrhœa. He died in a few days.

*Dissection.* On opening the abdomen the diaphragm was found to be pushed upwards as high as the third ribs ; the sharp edge of the liver descended nearly to the crista ilii, and quite concealed the stomach and a large portion of the intestines. The pyloric end of the stomach appeared to be hypertrophied : when cut through, a cancerous fungus was found on the mucous coat of it : although the surface of the mass projected four lines at least beyond the rest of the stomach, the pylorus did not seem to have been at all obstructed. We now can understand how this formidable disease may have existed without causing any symptoms during the life of the patient ; for the tumour in the epigastrium, which is regarded as the pathognomonic sign of cancer of the stomach, was, in the present case, hid by the enlarged liver ; and as the pylorus was free and open, the food was not rejected by vomiting, as is almost always the case. To revert for a moment to the state of the liver—its size was truly prodigious, and it weighed 12½ pounds, whereas the average weight of a healthy liver is, according to Sœmmering, between two and five pounds ; when cut into, it presented the appearance which has been called " cancer in disseminated masses ;" there was a vast number of tubercles, varying in size from that of a pin's head to that of the point of a finger, and either isolated, or connected one with the other ; so thickly studded was the liver with them, that not a fifth part of it was of the ordinary parenchymatous texture ; when cut into, they were observed to consist of a lardaceous whitish substance, and of a central yellowish substance, which was streaked with red lines ; in some places they were hard, in other places soft and encephaloid. Around these masses, the parenchyma of the liver appeared injected, of a yellowish colour, and of a broken-down texture. No cancerous debris was found in the vena cava, nor in the hepatic veins ! !—*Ibid.*

Few of our English readers will admit that the preceding is a case of " cancer of the liver." The very description of the author himself must satisfy them of the contrary. We quite regret that the French, who have deserved so well of the medical profession by their pathological researches, should be the least accurate of all authors in their language. It is a question if real scirrhous disease has ever been found in the liver ; and if we abide by the definition of scirrhus, as it occurs in the breast and uterus, we shall probably be forced to acknowledge, that this morbid change of structure is seldom or never seen in parenchymatous viscera. To satisfy our readers still more of the vagueness and inaccuracy of French reports, we shall translate the following short passage. The author vauntingly enquires, "in such cases as the above, is a physician to exhibit strong purges and large doses of calomel, as they do in England and in India, where chronic hepatitis, and, consequently, cancer of the liver, are often caused by the abuse of purgative medicines"!! This requires no comment.—REV.

---

### XVIII.

### INFLAMMATION OF THE SPERMATIC CORD APT TO BE MISTAKEN FOR INGUINAL HERNIA.

THE following remarks, from the Dictionnaire abrégé des Sciences Médicales, are important.

"We have repeatedly witnessed cases, in which the surgeon has been puzzled to form a correct diagnosis. When a painful swelling in the groin follows a fall, or any violent effort, rupture is immediately supposed to have taken place; but perhaps quite another accident may have occurred. The following is a case in point.

A man fell from a considerable height, and on recovering himself, he felt severe pain in the inguinal canal, and the pain extended to the loins; vomiting came on, and a feeling of general distress and anxiety; the abdomen was drawn in, and was very painful on pressure; the pulse was small and wiry; and, upon examining the groin, an oblong, hard, and painful tumour extended from the inguinal canal towards the testicle: it might have been mistaken for hernia, but it was observed that it was identified with, and formed, as it were, a part of, the spermatic cord, and, moreover, that it did not terminate inferiorly, as a rupture does; that the pain followed exactly the course of the cord, and was of that sickening character which is so characteristic of any injury of the seminal apparatus. These particulars removed all doubt from the mind; the patient was bled, leeched and poulticed, and was speedily well." —*Annal. de Med. Physiol. June*, 1832.

---

### XIX.

### SINGULAR CASE OF RECOVERY OF THE MOTIONS OF A STIFF KNEE-JOINT DURING FEVER.

AN old artillery-man was much exposed to the weather at the siege of Toulon, in the second year of the French republic; the consequence was, that he became a martyr to severe and long-protracted rheumatism. At the expiry of three years, an abscess formed in the left knee; the discharge was sanious, and mortification was apprehended. Amputation was advised, but not acceded to. In course of time the wound cicatrized, but the muscles of the limb were contracted, and the tendons had become so stiff and hard, that the knee was quite immoveably bent at a right angle. The flesh of the thigh and leg gradually wasted away, and the patient was obliged to wear a wooden leg. In this state he continued for 32 years, but now he walks about almost as well as ever he did, without crutch or staff. The following is the history of this curious change.

In the month of February of the present year he caught a severe catarrh, which was accompanied with high fever; powerful sudorifics were given, and a very free perspiration was thereby produced. As symptoms of typhus came on, he was well vomited and purged. During the efforts of vomiting, he felt severe pains in all the joints, and a sensation of cracking in his bones, to use his own words. In a day or two after, he lost the use of his right arm, and considerable gastric distress came on; but it was quickly relieved by enemata, which brought away immense quantities of vitiated fæces; the perspiration and expectoration returned, and the pains of the bones abated. On the 12th day, the kneejoint was found to be more pliant, and more extensible than it had been hitherto; the limb was well rubbed, and, in the course of three or four days, the joint of the left knee was as supple as that of the right, so that the patient could move it about, and rest upon it in walking. It is still much atrophied, but, in other respects, is as sound as the other.—*Ibid.*

However extraordinary the preceding case may seem to many of our readers, we cannot doubt its authenticity; indeed, in our own practice, there occurred, about a twelvemonth ago, a very singular instance of a female recovering the use of the left lower extremity, after a lapse of 12 years' motionless inaction of the member. During the delirium of a fever, she rose of herself from bed, and walked a few paces about the bed-room, and from that period to the present, she has enjoyed the perfect use of the limb.—Ed.

---

### XX.

### Organic Disease of Stomach, without Symptoms.

A man, aged 68, was admitted into Sir P. Dunn's Hospital, 9th July 1830. He had slept in a damp bed four weeks previously, from which had resulted catarrh, dyspnœa, anasarca, for which he came to the hospital. His appetite was tolerably good, and he did not complain of any pain in the stomach, or nausea. He had slight diarrhœa. He died, after seventeen days' residence in hospital, of the diseases above-mentioned, and without making any complaint respecting his stomach.

"On dissection, the stomach externally presented the appearance of the hour-glass contraction; on opening into its cavity, this contraction was found to be connected with a tumour caused by a morbid growth from the mucous membrane. This tumour was of a medullary structure, and exhibited, at one portion of its surface, a bloody appearance. In size it equalled a large mushroom, its edges overhanging the base, which consequently had somewhat the appearance of a peduncle. It was situated in the greater curvature, about midway between the cardiac and pyloric orifices. The peritoneal coat over the tumour was adherent to the mucous membrane, and presented a corrugated appearance. There were two small tumours of similar structure in the neighbourhood of the large one, and like it, attached to, or growing from the mucous membrane by a peduncle; they differed, however, from the larger, in being connected with the mucous membrane alone, and in not having induced any morbid adhesions between that membrane and the other tunics of the stomach.

These tumours appeared to have been the result of a morbid growth totally unconnected with inflammation, and therefore occasioned no symptom indicating their presence. Had the man lived much longer, there can be scarcely any doubt that their structure would have undergone a remarkable alteration, and have become more vascular, so as to convert them into bleeding fungous masses. This change had already partially commenced on the surface of the large tumour."*

We have seen some cases where ulceration (which must have been accompanied by inflammation) occurred in the stomach, and gave no notice of its existence, till perforation of the coats and extravasation of the contents of the organ.—Ed.

---

### XXI.

### A Sketch of Broussais' Doctrines, drawn up by Himself.

It was in the year 1804 that I began to be sensible of the imperfect and erroneous notions of disease, which were then prevalent. I laboured diligently for five years, and in the year 1809, appeared the "History of the Chronic Phlegmasiæ." At that time I was at a distance from Paris, and engaged in the army hospitals. The work was one

---

* Dr. Graves. Dublin Journ. No. V.

of an entirely experimental nature, and obtained an honorable mention from those who were able to judge of its merits. At the time at which it was published, the attention of physicians had not been at all directed to the subject of chronic inflammations, especially of those which have their seat in the membranes lining the visceral cavities. Pinel does not even mention them in his Nosographie. In their place we find the unmeaning terms "organic diseases, or defects, and atrophy or decay without any appreciable cause." Corvisart too was ignorant of the true nature of internal morbid changes. If in a case, which was neither consumption, nor disease of the heart, nor any of those inward swellings denominated at that time organic alterations, he observed the progressive decay of a patient affected with a chronic malady, he attributed it to a state of mere feebleness and cachexy (malus habitus); expressions vague and utterly unmeaning, or rather we should say extremely mischievous, as leading to erroneous indications of treatment. The History of the Chronic Phlegmasiæ corrected these faults, and shewed that inflammation is the chief agent in the production of those diseased growths and changes which we meet with in the centre of the viscera, and also in the texture of their lining membranes, and that these are the real causes of that incurable exhaustion and decay, which had hitherto been always referred only to debility of the solids, and depravation of the fluids of the body. Moreover it proved that such debility and such depravation are often curable; it pointed out the signs and epochs of their curability, and also the means for their cure. Henceforth the term "organic disease" had a definite and appreciable meaning; medical men were satisfied in attempting to soothe, and not vainly to eradicate such; and their attention was chiefly directed to prevent its occurrence, or progress, as soon as they discovered the germ in those obstinate and intractable irritations which equally invade all structures and tissues. The treatment of such cases was no longer empirical, but rational and consistent. The class of fevers was at this period as imperfectly understood, as those of cachexies and of organic diseases. Continued fevers were divided into two sorts; the one attributable to the inflammation of a particular organ; the other being viewed as essential, or independent of any local affection; but such a phrase as "essential" means nothing; for we are still puzzled to say, to what cause are we to ascribe an essential fever; in this state of ignorance, the physicians tried to characterise such a case either by the predominating symptoms, or by vague and idle conjectures and speculations; if the secretion of the bile was much increased, the fever was termed "bilious;" if that of the mucous surface in any part, a "mucous or pituitous" fever; when the temperature of the body rose high, then it was a "burning" fever; and when it fell much, it was a "cold, or algid" fever; again fevers were called asthenic, putrid, nervous, and ataxic, as they happened to be attended with exhaustion, with putrescency, and with symptoms of disturbance of the nervous and muscular symptoms; there were also hospital, camp, and prison fevers; and fever with petechial, miliary, or other eruptions. In some cases, the character and name were drawn, it would seem, from an idea that a baneful agent was working its effects upon the patient, and defeating the physician's efforts; and hence such fevers were styled "malignant." Does this language convey any knowledge? or does it not rather prove the confusion and chaos in which the subject was involved, and mislead the practitioner not only in his attempts to reason on the nature of the diseases, but also in his efforts to cure them? Hence one set of men was drawing the indications of their treatment from the state of the biliary, or nervous secretions; while another set was talking only of debility, putridity, or malignancy. Pinel was the first to perceive, and to aim at correcting these errors of the schools. He tried to localise some fevers, as for example the bilious fevers, under the name of meningo-gastric, and the pituitous, or mucous fevers, under that of adeno-meningeal. But he has not told us in his Nosographie, of what nature is the gastric disorder, nor yet of the cause of the encreased quantities of mucus, which is characteristic of the second class ; moreover he fell into the egregious contradiction of attributing these two sorts of fevers, sometimes to irritation, and sometimes to a primitive change of the biliary and mucous secretions, at the very time that he designated them, essential fevers. Then he vaguely ascribed all his ataxic fevers to a disorder of the nervous system, and most confusedly classed together all fevers in which the vital energies are depressed, and termed them adynamic. Not only was there no localisation of the disease, but the indications of treatment in such a system were most injurious : for what is the cause of the loss of power, or debility in fevers, but simply inflammation of some of the viscera ? Such was the state of the science, when I published, in 1816, the first edition of my Examination of Medical Doctrines. In it I pointed out the absurdity of expecting to advance the science of medicine, merely by forming groupes of symptoms into particular diseases, which is not less absurd than to say that an assemblage of ten, or twelve symptoms is the virtual cause of those morbid changes which we observe after death. The symptoms in short are nothing but the external and obvious evidences of the malady which is involving the organs in abnormal changes. I proposed to view fevers, as we do inflammations, to localise them in certain parts, or organs, and I attempted to show that the morbid changes of structure which are going on, are the real existing causes of fevers, and not, as had hitherto been supposed, the effects of them. At the same time, I strongly inculcated the propriety of extreme caution in our reasoning on those cases, where the " mobile" of the fever is not apparent. These new doctrines encountered much opposition, but they gradually gained ground, and became more and more adopted, as will be seen by the following events. In 1812, was commenced the large " Dictionnaire des Sciences Medicales," the articles of which were deeply imbued with the doctrines of Pinel, till the year 1817, from which time some sprinkling of the physiological reasonings began to appear. Before the work was completed, the " Dictionnaire Abrégé" was brought out, and in it the physiological medium was preponderant. In the large Dictionary, fevers are enumerated as " essential, or idiopathic ;" in the abridged one, we read of none but such as are symtomatic. In both, the doctrines which I had unfolded in my History of the Phlegmasiæ, were insisted upon, in discoursing upon all the diseases which are included in the catalogue of inflammations. For the last sixteen years, almost all medical researches have taken their rise from, and have been based upon the physiological doctrines. The subject of inflammation is studied, discussed, and specialized, much more than it was formerly ; and where inflammation cannot be proved to exist, irritation is supposed to be present, and is now deemed to be the agent in producing a host of organic diseases, which were before merely enumerated in detail. The relations between the changes and vitiations of the fluids, and the different sorts and degrees of inflammatory and irritative actions, are attentively and most zealously examined. In 1821, appeared the second edition of my "Examen des Doctrines" which was published originally in one volume, then in two, and at length in the fourth edition in four volumes. Soon after this period I began my system of " Physiology applied to pathology ;" it was brought out in numbers for four successive years. It is devoted to the examination of the etiology of diseases, and to the tracing of those functional aberrations from the healthy to the diseased state, which are caused by the influence of the external agencies continually operating on the living body. This work, as well as the preceding ones, has received the honour of translation into several languages. In addition to these, the " Annales de la Médécine Physiologique" have appeared in monthly numbers for the last eleven years, and my Treàtise on Irritation and Madness in 1825, in which last work the cause of the "positive philosophy" was pleaded with a boldness and a freedom, which might have been dangerous to the author. Lastly the cholera morbus, which has so puzzled and bewildered all medical enquiries, must be viewed through the enlightening medium of correct physiology, before we can hope to arrive at a successful therapeutic. We have explained our views upon this subject in an essay which was lately published. According to the physiological school of medicine, no disease is ever primarily and essentially general, that is, affecting equally and at once every part of the system. Disease begins always in one individual organ, even when the disease depends upon a cause which induces an alteration in the humours, or fluids of the body, such as the exciting cause of small pox, typhus, &c. &c. The great aim of the practitioner ought therefore to be, to discover the primitive seat of the disorder, as he may thereby often be enabled to check, and even strangle the nascent evil. By following this mode of examination, French authors have surprisingly reduced the catalogue of malignant fevers, which are now seldom met with, except when medical assistance has been called too late, or has not been accepted. Look at the reports of the different hospitals, and mark how seldom we find the term of general or essential fevers, and how prevailing is the belief that all diseases ought to be regarded primarily as local affections. Even when the physician does not see the case, until the disturbance has been propagated to, and has involved all the functions of the economy, he can do much to relieve his patient by following the physiological method, and directing his attention to those organs which have chiefly suffered. Hence we perceive the absurdity of recommending in fevers only one set of remedies to the exclusion of all others; the remedy must be adapted to the condition and susceptibility of the affected organs. The operation of all agents which influence and modify the state of the system, ought to be our especial study, and the effects of these upon the moving and sentient powers, our only guide in judging of the results. Thus we admit no à priôri reasonings and conclusions, into our school; neither any preconceived opinions, nor any implicit following the "verba ullius magistri." Let it not be supposed that our doctrine is that the abnormal changes and modifications, which we have alluded to, are the only immediate causes of all diseases; we know that these causes may exist in the humours, in certain poisons, in the vicissitudes of heat and cold, in the imponderable agents, and probably also in influences, which cannot be appropriated by our senses, and we are not opposed to any researches calculated to explain or illustrate the action of these causes, and to discover antidotes to them, but we simply maintain that a disease can be recognised only by the aberration from the normal condition of the moving and sentient powers of the body, and that the study of this aberration, sometimes as excessive, at other times as impaired, or irregular action, can alone furnish to the medical man the correct method of ascertaining whether his remedies are beneficial, or injurious. Formerly a patient, who was getting worse, instead of better, while under medical treatment, was often told "Have patience, it is the medicine which is acting upon you;" a gouty man was consoled by hearing that his physician must not relieve him, for his sufferings were in sooth necessary for the object which nature had in view; and even in many acute diseases, the patient was congratulated on any increase of fever and restlessness, upon the ground that a salutary crisis of his malady was at hand! How often was the raging thirst of fever met with hot drinks, which only add fuel to the distress! We have a memorable instance of this folly in the late treatment of the cholera; but now, thank God, the poor sufferers are permitted to have as much cold water and ice, as they desire. There are indeed cases, in which the patient must submit to the annoying effects of certain medicines; but such cases are infinitely more rare than was formerly believed; but yet we must acknowledge that much injury is often done by their employment; thus, in dyspepsia, how frequently do we see stimulants and tonics ordered, although it is obvious that they must either increase the distress, or only change its character. In short, the art of sparing suffering and pain is not so ancient as many suppose, and has been fostered chiefly under the happy influence of the physiological method; for the very essence of this method is to bring relief to suffering organs, and not to regard diseases merely as assemblages of certain symptoms, which must either co-exist, or succeed to each other; by this foolish practice, we neglect the very increase of those evils which are successively added to the inseparable distresses of the malady. Is it not a common remark of a disciple of the old school, when summoned to a patient—"we must wait; the disease has not yet declared itself?" But such dangerous absurdities are gradually passing away; the mind of the physiological physician is occupied with facts and realities, and not with preconceived and à priori reasonings.

In enquiring into the nature of a disease, there are but four objects of research—1, The organ primarily affected. 2, The operating cause by which it has been affected. 3, The influence of this organ upon the other organs of the body; and, 4, The agents by which the disorder may be removed. There is nothing of idle speculation here—no empty hypothesis on the intimate or proximate nature of diseases, for these are beyond our intelligence; we do not pretend to give an explanation of the phenomena of life; our only aim is to take notice of whatever disturbs its moving and sentient powers, or disorders the texture of the solids and the composition of the fluids, and to compare it with whatever has the effect of restoring the one and the other to a state of normal rectitude. How, then, can any one be so unjust as to denominate the physiological method, a system of à priori reasoning and practice, or as a monomania, or a partial, contracted, and exclusive doctrine, which attends only to one set of measures, and disdainfully rejects every other?—*Annal. de Med. Physiol. June,* 1832.

In order to unfold the views of this distinguished reformer of medical science, we are induced to give a short abstract of the essay which he read in October, before the Academy of Sciences of the Institute at Paris, as a candidate for the chair vacant by the death of M. Portal. It is entitled "Memoir on the Philosophy of Medicine."

He remarks at the outset, that, at different epochs of history, very different opinions have been held on the meaning of the phrase "medical philosophy." Hippocrates deemed it to consist in closely observing the march of diseases, when they are little modified by the interference of art. Galen made it to reside in the study of the humours; Paracelsus and his astrological followers, in that of the stars; Vanhelmont and Stahl brought it down again to earth, and fixed it in the centre of every organ, designating it the "archæus" and the "animus" which presides over the body. At length, the immortal Haller shewed that it was more philosophical to search for its abode in the irritability of the tissues, and to ascribe health and disease to certain states of these, rather than to subtile and irritable nonentities. But soon a new set of theorists arose, who maintained that all the phenomena of life, both in health and disease, are referable to the action of the nerves; but, unfortunately, the nervous functions are little known, and in course of time a being or essence, semi-intellectual and semi-material, partaking somewhat, too, of the nature of the "archæus" of Vanhelmont, and of the "animus" of Stahl, was admitted into all medical reasonings, as the presiding influence of the animal economy. This new philosophy was simple, to all appearance; but it was speedily split, and two rival systems sprung from the division. In the one, which was upheld by Barther and the Montpellier school, a vital force or energy was made to reign over all the tissues and humours of the body; but its seat was not limited to the nerves, as was the case with its parent doctrine; according to this philosophy, there were as many vital forces as there were vital phenomena: in the other, the elements were "force" and "debility," terms which are synonymous with an "excess and deficiency of" excitability, or with "accumulated and exhausted irritability." Such was the Brownonian doctrine, a doctrine which led to a most pernicious system of therapeutics, for remedies were now employed with such whimsical and irrational intentions, such as to increase or to diminish excitement, to affect the irritability, as it chanced to be redundant or defective, just as, before, they were addressed to the archæi and animi, and with equal disregard to the effects which they produced on individual organs. It was Sydenham who first proposed the classifying of diseases; Sauvages seized the happy idea, and, comparing diseases (which are only modified conditions of a living body) to plants (which are living bodies themselves), gave birth to nosology! Nothing was talked of but classes, orders, genera, and species, as in botany; his adage was, "symptomata se habent ad morbos, ut folia, et fulcra ad plantas," an adage whose absurdity must now be apparent to every one, but whose influence, strange to say, has not yet entirely passed away. It is surprising that no one could see the folly of comparing pain and heat to a leaf and a branch, and a spasm to a flower!! It is useless to advert any longer to these idle abstractions and vagaries of the brain, or to expose more of the ridiculous conceptions which other authors have, at different times, propounded; for surely medical "ontology" is for ever abolished. It was formerly the great problem among physicians—"a disease being given, to find its remedy;" but latterly the problem had been somewhat changed, or rather a new problem was in vogue—"a disease being given, to find its place in a nosological catalogue." How humiliating to think that the minds of men were so diverted from the true path of enquiry, and that they allowed their judgments to be blinded and misled by the ridiculous nothingness of empty, sounding words; but let us not boast, but rather ponder on these errors, for the improvement of ourselves.

Our next theme is, to strive to find out some more rational system of medical philosophy. The eclectic school have put forth their claims; they tell us "there is something good and true in all the preceding doctrines—let us only reject the bad and retain the good." Such is the method of those who profess not to be "exclusives." But we need not waste time in refuting the reasonings of the eclectics, a name which ought to be reserved for close observers and able experimenters, whose time and talents are engaged in verifying facts which have been long admitted, and in seeking after others which are novel and interesting. How is it possible for an eclectic to arrive at any logical correctness, if, surrounded with so many statements and data, brought forward by one party and refuted by another, and encountering so many opinions and hypotheses utterly conflictant with each other? What could a "neophyte" do amid such a labyrinth of perplexities? Eclectics are not known in the true sciences, where the simplicity and the small number of facts appertaining to any theorem, render its demonstration easy and intelligible. The great error of most preceding writers has been, in seeking after the primary and proximate causes of disease; and, even in the present day, we hear men often regret the misfortune of not being able to attain to the knowledge of the essential nature of diseases; they are unwilling to admit any evidences or elements of certainty and truth in science, but such as are proved by the testimony of the senses, forgetting that there is another kind of evidences obtained, by inductive reasonings, from facts which have been well established; and such evidences are not less real than a shadow, a noise, a reflection, or evidences of a material object existing somewhere; they are admitted into mathematics, chemistry, mechanics, agriculture, and exercise a mighty influence in all discourses upon these sciences.

None can be ignorant of the stupendous results derived, through this means, by Cuvier in fossil geology. It is, indeed, difficult to obtain this sort of evidence in medical researches, but the cause of this is, that the facts, or rather data, are so very numerous and complex; but we must not be discouraged, for it is only by the medium of such evidence, that we can hope to arrive at a knowledge of the causes of disease—to a correct nosology, and a rational and beneficial system of therapeutics. This class of evidences is weak and deficient at present, but we must labour zealously in enriching its domain, for it constitutes the most important aim of medicine; yet such is the medical philosophy of our days, that rigid induction has been less esteemed than mere description; the former is falsely designated as an hypothesis, theory, a system à priòri, and an empty conjecture. If a treatise is written on any disease, the only aim and end of the author are, after describing its course and progress, and detailing the pathological changes in fatal cases, to establish that the disease could not be at all different from what it really was, or that the symptoms were merely the effects of the morbid states and actions which were going on in different viscera, and no attention is paid to appreciate the operation of the exciting cause, and of the agencies which modify the actual state of disease, and scarce any thing is thought of but the hunting after nostrums and specifics. The habit of expecting a necessary succession of symptoms during a definite period, in most maladies, and especially in those which have been denominated putrid and malignant fevers, typhus gravior, &c. is most pernicious and baneful. We have laboured to expose the dangers of such a method, and we deem it one of the noblest endeavours of a physician to shew, that the greater part of these affections may be arrested at the different epochs of their development, by means of a scientific application of those agents which modify and control the functions of our organs. In conclusion, the philosophy of medicine which we have proposed, consists in a more rigorous observation of the effects of all the external agents on the human frame, and of the influence of the different organs of the body on each other. —*Archiv. Gen. Oct.* 1832.

We have already said that the preceding is an analytical review of the essay which Broussais read before the Institute of France. It is customary, that the candidates for the honor of being enrolled members of that most distinguished association should present memoirs, in which they give an abstract of their scientific and professional labours and researches, specify the titles and honors which they have hitherto received, and assert their claims to the object of their ambition. The four candidates were MM. Broussais, Esquirol, Breschet, and Double. The latter read an essay on the "Influence of the Nervous System in the Production and Development of Diseases." The Commission appointed to enquire into their respective merits, selected MM. Double, Broussais, and Breschet to be presented to the Academy of Sciences; and the votes of 50 academicians were afterwards ascertained to have been awarded as follows: on the first scrutiny, 23 for M. Double—16 for Breschet—10 for Broussais, and 1 for Esquirol; on the second, 24 for Double—23 for Breschet, and 4 for Broussais; and, on the third, 36 for Double, and 24 for Breschet. M. Double was, therefore, declared the successful candidate.—Ed.

---

### XXII.

### Death of M. Portal.

This distinguished veteran died on the 23d of July last, at the good old age of 91, as rich in honours as in years. He was the Nestor of the French physicians; first physician to Louis XVIII. and Charles X., perpetual president of the Academy of Medicine, member of the Academy of Sciences, and of many other celebrated societies. His "History of Anatomy and Surgery" has long been esteemed and admired; but his greatest work was the "System of Medical Anatomy," which laid the foundation of, and rendered popular, the pursuit of sound pathology in France.

---

### XXIII.

### ON WATER, APPLIED EXTERNALLY, AS A REMEDIAL AGENT.

IN our last Number, we gave an extended analysis of a very valuable paper on the above subject; we now continue the subject, and shall direct our readers to the fittest method of employing the remedy. In most cases, especially those in which the nervous system is much affected, as in the 3d, 4th, and 5th series, it is of importance that the bath should be in the bed-room, or in an immediately adjoining apartment, as the patient ought to go to bed immediately afterwards; in other cases, as in those of the 1st, 2d, and 6th series, it is better that it be at some distance, as the exercise of going to and coming from the bath is beneficial. The heat of the bath ought to be about 86° or thereabouts; the affusions ought to be made with the water of the bath at first, and then gradually with cooler water. As a general rule, it may be said that the temperature of the bath should be about 86°, and the water for affusion from 6° to 10° lower. With regard to the duration of the bathing, this must vary according to the nature of the malady. In the cases of the 3d and 5th series, those, namely, characterized by lethargy and nervous stupor, from five to eight minutes is long enough; but, in cases of the 1st, 2d, 4th, and 6th series, viz. those of cerebral fatigue and exhaustion, fatigue of the brain and of the organs of sense, nervous fevers, and neuroses of the sympathetic nerve, 25 to 30 minutes may be mentioned as a proper time, the affusions being employed during the six or eight last minutes. Now the number and frequency of the baths is also a point of importance to determine: when the patient is afflicted with stupor, nervous fever, or lethargy (as in the 3d, 4th, and 5th series), it is seldom that a single bath in the 24 hours is sufficient; generally two baths at least are necessary; sometimes 3, 4, or even 6 and 8, according to M. Recamier, must be taken in one day, before the patient experiences decided benefit, whereas, in instances of the 2d and 6th series, one bath daily is amply sufficient. A third interesting question of enquiry is, the proper hour of the day for the use of the bath. In cases of the 1st and 6th series, the most fit time is shortly before the evening repast, or not long before going to bed; the appetite, in the one case, is often much invigorated, and, in the other, sleep may probably be soon induced; it may be proper that the patient should take a light supper after he is in bed.—*Révue Médicale, Sept.* 1832.

*Remark.* We have repeatedly witnessed the most agreeable effects from the employment of bathing, in a somewhat similar form to that recommended by the French physicians, in many cases of cerebral exhaustion, of melancholy, of general debility uncomplicated with organic disease, &c.; we suggest the cotemporaneous effects of effervescing draughts, such as the seidlitz powders, to the more robust, and the citrate of ammonia to the more feeble and nervous. The wonderfully soothing effects of a saline purgative are not very generally known, and may probably be rather despised, by those who have never experienced their effects on themselves, or witnessed them in others. Suffice it to say, that Lord Byron mentions it of himself, that when a fit of the "blue devils" was impending over him, a spoonful or two of Epsom salts always restored his sprits more quickly than the finest wines, and we know a writer, of distinguished talent, who confirms the truth of the remark by his own practice.—ED.

---

### XXIV.

### CASE OF ANEURISM OF THE LEFT GLUTEAL ARTERY.

MAD. S. aged 66, fell on her left buttock in the month of December, 1821. She was considerably hurt, and a small, hard, and painful tumour very suddenly shewed itself, about the centre of the injured part. In February, 1825, she again fell on the same buttock, and was much bruised; at that time, the swelling was of the size of a hen's egg; when examined, it was found to beat synchronously with the heart. Her medical attendant was not aware of the nature of her malady. The pains which shot from the hip to the foot were now so severe, as to rob her of sleep, and so much had the swelling increased in a few months, that in the following November it measured 21 inches in circumference; the pulsations could be felt over all its surface. The state of the patient forbad any operation. She died in the February of the following year.

*Autopsy.* The left common iliac artery presented here and there spots of ossification; the enormous aneurismal sac when opened measured upwards of 20 inches round, and contained a large quantity of broken down blood; the substance of the gluteal muscles had been almost quite absorbed; the gluteal artery was large enough to admit the forefinger; and its parietes appeared healthy to within an inch of the sac.—*Ibid.*

---

### XXV.

### Ulceration of the Stomach.

Dr. Graves visited an unmarried lady, aged 25 years, who had suffered much from pain in the stomach, nausea, vomiting, acidity, and indigestion. These symptoms first appeared after a severe shock and fright. She had sometimes intervals of five or six weeks at a time, free from complaint, but these intervals had lately become much shorter. She was pale and emaciated. She was free from pain for some months after Dr. Graves commenced attendance, then pain came on, attended often with a discharge of coloured fluid from the stomach, sometimes resembling coffee-grounds. This state continued for some months, when the pain and tenderness disappeared, though vomiting remained, after every kind of food. She was at length worn out. On dissection, an ulcer was found in the neighbourhood of the pylorus, but completely cicatrized. It had penetrated the mucous and muscular coats, but had been arrested by the peritoneal covering, there was no maturation or scirrhus in the neighbourhood. The site of the cicatrix was distended into a kind of pouch, which received a portion of the contents of the stomach, and thus formed a kind of valve which prevented the egress of chyme through the pyloric orifice, nutriment was thus cut off from the system, and death was the result. *Dublin Journal,* No. V.

P. S. Our esteemed Contemporary of the Sister Isle, has honoured us by the extraction of several articles, for which we feel obliged. But we trust that it is no unreasonable request that the source may be *correctly* given. "*Johnston's* Journal" is not a very clear designation for the "Medico-Chirurgical Review, edited by *Dr. Johnson.*" The term is merely from carelessness, we know, and we are sure the hint will not give offence. We borrow freely from the excellent Dublin Journal, and shall always feel gratified when any of our articles are deemed worthy of transplantation into such a fertile soil.—*Ed.*

---

### XXVI.

### On the secret Causes of Epidemic Diseases. By Professor Baron Alibert.

In a preceding article, the learned author had laboured to shew that the causes of Epidemics were altogether unconnected with mere localities; they are in no manner dependent upon the nature of the climate and may be developed in temperate, as well as in extremely cold and hot regions. It is a mistake to regard the Torrid Zone as the only cradle of pestilential diseases; we do not indeed deny that the germs of some diseases may exist in the country where these prevail, and that such diseases are very properly designated "endemic;" thus for example of cold and damp districts, intersected with woods and marshes, scurvy and all its offspring of evils, appear to be the natural denizens; while those which are dry, exposed and well watered, are usually much invaded with inflammatory disorders. We may go still farther, and assert that some diseases appear to be so affixed to certain countries, that they are seldom or ever found in others, unless transported by patients themselves; and then, they usually degenerate and are considerably modified from their original type. But the essential characters of an epidemic miasm, viz. the suddenness, and extent, and sweeping fatality of its ravages, are sufficient to distinguish a pestilence from a disease of mere climate, or in other words from an endemic. Hippocrates, in his Treatise on Air, Waters and Localities, acknowledges that such can never generate a pestilential disease; they may however modify its character, and impart a colouring to its features. What strongly argues the distinctive nature of an epidemic, is that it is not limited to any one, or two countries or districts, but sweeps over a great extent of the earth. We witness them to prevail alike in Russia and in Poland, among the Turks and the West Indians. It is a very prevailing notion that all the great epidemics have sprung up in warm climates, and have been diffused from south to north; but this is not quite correct; for example, the yellow fever is known to arise in the cold latitudes of Massachusets and New Hampshire, and in the temperate regions of Georgia and the Carolinas, as well as in the burning Antilles; and it not unfrequently ravages the most northern towns of the United States, while the inhabitants of the more Southern, and of the American Archipelago are quite free from it; so often is this the case, that the merchants between the tropics accuse the people of New York and Boston of having imported the disease. The great pestilence of 1798, which ravaged the United States commenced at Boston, situated in 45° N. and that which swept over so large a portion of Europe towards the close of the sixteenth century, shewed itself first in the Gulf of Finland, then passed over Livonia and other northern countries, to England, afterwards to Italy and the South of France, even to the Island of Crete. The dreadful plague of 1719 began at Aleppo, and is supposed to have furnished the germ of that one which depopulated Marseilles on the following year; and again, a previous plague of the early year of the 17th century, appears to have broken out at nearly the same time in the northern and southern nations, as Russia, Livonia, England, France and Turkey. It appears therefore from the preceding date, that epidemic disorders are confined to no particular climates, or latitudes; this is one feature of their character; another is, that the human system appears to become somewhat accustomed to their pernicious influences by a long residence in the countries visited chiefly by them, so that it is less susceptible of suffering from their attack; as a proof of this, we may state that the Creoles do not fall victims to the yellow fever in the same proportion as the other inhabitants of the West Indies, thus two-thirds of the fine French army sent in 1803 to St. Domingo, were sacrificed by this plague, at a time when the Creoles, Planters and Negroes, were enjoying complete exemption. The variety of a second attack of pestilential disorders is a subject worthy of attention. Dr. Russel states that out of 4,400 patients who had the plague, only twenty-eight were attacked by it a second time, a statement which shews that the animal system can in course of time adapt itself to the most deleterious atmosphere.—*Révue Med. Sept.* 1832.

*(To be continued.)*

---

### XXVII.

### USE OF TARTAR EMETIC IN PNEUMONIA, &c.

*Case* 1.—A female, aged 29, was admitted into the Hôpital Necker with symptoms of pneumonia; in addition to the ordinary symptoms, the crepitating râle could be heard distinctly. She was bled, and put on low diet. Next day she had thirty leeches on the side; on the following day (28th March), she was again bled; on the 29th, the dullness on percussion had increased; bronchophony was extensively heard, and likewise bronchial respiration. Bleeding repeated. On the 30th, a blister ordered. On the 2d of April, the symptoms were bronchial respiration; louder crepitating râle; almost the whole right side, sounding dull when struck; pulse intermittent. Sinapisms to the legs.

3rd. Tending to delirium; thoracic symptoms nearly as before. Infusion of polygala senega; a mixture containing four grains of emetic tartar ordered; and to be repeated in the evening with the addition of some syrup of poppies.

4th. The patient has been copiously purged; but not much vomiting; is better on the whole. Emetic mixture repeated.

5th. Has been only triflingly purged and vomited; the "tolerance" of the drug has been established; pulse again intermitting; the emetic mixture to be given twice with syrup of poppies.

6th. Several alvine evacuations; pulse intermittent; the chest is becoming more sonorous at the upper right side; the respiration and subcrepitating returning râle is heard. Omit the antimonial mixture.

7th. Great prostration; eyes dull and sunk; pulse small and intermittent; nevertheless since the auscultatory state of the chest is more satisfactory, the antimonial to be resumed, and the quantity encreased to eight grains, taken in two doses, at two hours' interval.

8th. Local symptoms of the peripneumonia still abating; only two stools.

9th. Better; no stool; pulse 75; chest has almost recovered its natural sonorousness. A mucous subcrepitating râle is heard.

From this time she gradually improved in health.

*Case* 2.—A female aged 25, was admitted on the 20th June, with all the symptoms of pneumonia, which had existed for several days. We need not enumerate the symptoms, and shall only allude to the auscultatory signs. The chest is dull behind on percussion; the respiration on the right side is bronchial; on the left, the crepitating râle is heard feeble, and as it were, at a distance. Venesection.

On the following day all the symptoms nearly the same: ordered, a mixture containing eight grains of tartras antimonii, to be taken, by a spoonful, every half hour.

22nd. Has vomited four times, and been purged about 20 times; a favourable change already visible. Mixture with six grains.

23rd. Not quite so well; has been neither vomited nor purged, so that the "tolerance" is now established. Mixture to be omitted, and simple pectoral medicines ordered.

24th. Much better; crepitating râle heard distinctly on the right side. She gradually recovered.

*Case* 7.—A carman aged 37, after being exposed to cold and wet, was seized with pain in the chest, cough, dyspnœa, &c. these symptoms had continued for three days before he entered the Hôpital Necker on the 2nd of January. When examined by percussion, the chest sounded dull behind; the respiratory murmur was not audible, but there was distinct bronchophony; no crepitating râle on the right side; whereas, on the left, the sound on percussion is clear, and the crepitating râle is well marked. V. Sectio. Mixture with eight grains of the tartras antimonii.

3rd. One stool; has not vomited, is sensibly better, respiration is less bronchial, and the air is heard to penetrate more freely into the right lung, as indicated by the presence now of the crepitating râle on this side. Continue the mixture as yesterday.

4th. Still better; the "tolerance" is now induced; no stool, nor vomiting. The dose of the antimonial to be encreased to 12 grains.

5th. No evacuation upwards or downwards, crepitating râle more distinct on the right side, and the bronchial respiration is less. Dose to be increased to 15 grains.

6th. The left lung, which at first presented the crepitating râle, has now returned to its normal condition. Pulse has fallen to 60. Mixture, with 18 grains.

7th. Continues better; the crepitating râle heard over all the posterior and right part of the chest. Continue the mixture, as yesterday.

He left the hospital quite well on the 28th.

*Case* 13. A man aged 42, laboured under pneumonia. He was ordered to be bled; but the surgeon failed in all his attempts to open a vein, so deformed and rickety was the patient. A mixture, containing six grains of the tartrate with an ounce of the syrupus papaveris, was prescribed. On the following day (15th Feb.), the patient was better; no vomiting, and only two stools. Mixture, with eight grains, and an ounce of the syrup.

16th. The auscultatory signs indicate that the pneumonia has advanced still more to resolution. Continue the mixture as yesterday.

17th. Is better; only a little mucous râle to be heard.

23d. Completely cured.

*Remarks.* M. Bricheteau, physician of the Hôpital Necker, and the reporter of the preceding, among many other similar cases, states that, in his experiments on the effects of the tartrate of antimony, he has been anxious to determine the particular cases in which the contra-stimulating practice, as it has been called by the Italian physicians, may be advantageously substituted for the antiphlogistic treatment. In almost all the examples which he details, bleeding had been employed at first; and, in most, it was only when depletory measures seemed to promise little success, that recourse was had to the antimonial administration. He selected 14 cases for trial, and, before ever exhibiting the powerful agent, most cautiously ascertained that there was no gastric or abdominal irritation to contra-indicate its use. He frankly admits that, when he commenced the practice, he was very sceptical of its good effects; but that now he regards it as a most potent, or, perhaps, the most energetic, of all curative remedies, in a vast number of pneumonic inflammations, especially in such constitutions as do not well bear evacuations of blood. The following is a brief abstract of its effects, in the 14 cases which are detailed in the author's paper.

In the 1st, the patient had been bled twice and had been blistered, without any decided benefit, before the antimony was used; and, although most unfavourable symptoms existed, he ultimately recovered.

In the 2d, a bleeding appears to have done little service, and two doses of the medicine sufficed to arrest the disease.

In the 3d, two bleedings had been ineffectually practised, without any amelioration of the physical signs; and yet four doses of the antimonial caused them speedily to vanish.

In the 5th, we find that a relapse took place after an abatement of the symptoms, which had been favoured by four bleedings. Two doses sufficed to restore the patient, and that, too, very quickly.

In the 6th, the good effects are also very obvious. In the 7th and 8th, the bleedings and antimonial were employed together on the first day of the treatment, as is the practice in Italy, the cradle of the contra-stimulating system. In one of these cases, the dose was raised to 18 grains daily.

In the 9th and 10th the results are also striking; in the latter, an exacerbation of the symptoms, which had been much mitigated, came on, yet promptly gave way to the antimonial, in the dose of 18 grains.

The 11th case was treated successfully with the antimonial alone; no bleeding was practised.

The 12th case is also a favourable instance. The 13th and 14th, patients died.

It will be seen that the medicine was never carried to the extent which has been recommended by the Italian physicians; and M. Bricheteau states that, whenever the resolution of the disease appeared to make rapid strides, he omitted it immediately, leaving to Nature the completion of the cure; when the resolution was more gradual, the dose was gradually diminished. He never exceeded 18 grains at once; sometimes he added small quantities of opium to it, to check the nausea which distresses many patients, and to hasten the "tolerance" of the medicine. It was usually exhibited in an infusion of chamomile and orange leaves, and given by a spoonful at a time, at very short intervals.—*Ibid.*

The preceding observations are well worthy the attention of every reader. We have given all the pith and substance of M. Bricheteau's memoir.—Ed.

---

### XXVIII.

### Dr. Graves on Periostitis.*

Dr. G. divides periostitis into two kinds —diffused and circumscribed. The former is that which usually terminates in necrosis, and falls to the care of the surgeon; the latter constitutes nodes. It is to the latter affection that Dr. G. limits himself, in a clinical lecture delivered at the Meath Hospital.

Circumscribed periostitis may arise from cold; most commonly from a specific cause, as syphilis, mercury, or scrofula.

It presents some varieties. First, it exists without detachment of the periosteum from the bone. The membrane becomes inflamed and thickened, the bone beneath vascular. There is now pain and tenderness in the part, sometimes with swelling and discoloration of the integuments. The affection becomes chronic, the symptoms rather decline. Then the periosteum becomes more dense, even fibro-cartilaginous. When this change has taken place, Dr. G. thinks it doubtful whether the diseased mass is ever again absorbed. We are not so sure of this. We see now and then, rarely, we admit, the most solid and bony nodes diminish under the influence of mercury. If healthy bone and fibrous tissue admit of absorption, why may not new osseous or fibrous structure? When ossification takes place in a node, the external lamina of the true bone beneath is in process of time absorbed, and, at the same time, a cancellated structure is developed in the node, the two orders of cancelli thus becoming continuous. The tibia of prostitutes are often much disfigured, from irregular bony elevations of this description.

Secondly, periostitis may be attended with detachment from the bone beneath, and of this there are several varieties. The first variety is this. In a space, varying from twenty-four hours to eight or ten days, an elevation appears on some part of a bone, with pain and tenderness on pressure, forming a tumour apparently solid, but, on accurate examination, somewhat elastic. Soon a gradual diminution of the pain and swelling occur, the fluid effused under the periosteum is absorbed, and the subjacent bone and periosteum become again united. This process generally occupies some time, but occasionally is more speedy. The tumours which rapidly form and disappear on the scalp are of this nature. The surface of the bone does not die. Usually ulceration of the skin does not occur in this variety; but, if it does, the cure is effected by granulations arising from the vascular surface of the bone. In the second variety, matter is effused beneath the periosteum, the bone affected becomes vascular at a little depth, but the surface is white and dead, and consists of a thin, worm-eaten, cribriform lamella, which, after some time, separates, and opens for itself a passage through the integuments. The cure is effected by granulations from the vascular part of the bone beneath.

Scrofulous periostitis is a simultaneous affection of the bone and periosteum. Sometimes, in vitiated constitutions, the periosteum becomes af-

---

* Lond. Med. and Surg. Journal, Dec. 29th, 1832.

fected, from ulceration commencing in the skin, as in rupia, ecthyma, &c. In all, except the first species of periostitis described, Dr. G. recommends cutting through the integuments and periosteum, as recommended by Mr. Crampton.

The scrofulous periostitis (node) is attended with milder symptoms—less pain and tenderness—less swelling—and occurs at an earlier period of life. Not but scrofula may be combined with the deleterious effects of mercury, or with syphilis. The history and the circumstances must determine this. Mercury and syphilis, too, may combine to produce node, indeed it is probable that, in most cases of node, this is the fact. Some of these cases are most perplexing and most miserable.

Dr. G. adverts to the treatment. As local means, leeches, blisters dressed with savine ointment, the antimonial ointment—in obstinate cases, cutting down to the bone—when ulceration takes place, and there are pale granulations, the introduction of a stick of nitrate of silver, and touching a given part of the surface every day. As to the general treatment, when the constitution is strong, and there is otherwise no objection, the use of mercury; and this Dr. G. considers the best mode, if the symptoms be violent, even though mercury should have been already given. Mercury carried to decided salivation, and continued three or four days afterwards, is particularly suited to the painful species of cranial periostitis already alluded to. When the symptoms are less violent, the pil. plummeri, blue pill in alterative doses; and, in debilitated persons, the corrosive sublimate perhaps, or De Velno's vegetable syrup. Dr. G. has seen several restored to health by the agency of the latter. A great objection to the use of mercury among the poor, even in hospitals, is their subsequent inevitable exposure to cold.

Next to mercury, Dr. Graves ranks colchicum and tartar-emetic; the former preceded by bleeding, and combined with an opiate and magnesia. Colchicum cannot well be combined with antimonials, on account of the disturbance of the stomach which they occasion. Narcotics are serviceable, during the whole course of the disease. When it becomes chronic, sarsaparilla may be given, with nitric acid.

We think these observations of Dr. Graves not unworthy of attention. The point to which we would particularly direct our readers' notice, is the employment of incisions when the pain is severe, the tension of the periosteum considerable, and inflammatory action present. We are quite alive to the powers of mercury in acute fibrous inflammation, and, indeed, in chronic: yet still, on the whole, we would rather do without it if we could. There is too much reason to suppose, that node would seldom occur as a sequela of the venereal *per se*, and even when, by mercury, we arrest the acute inflammation, we suspect that we are laying the foundation, in many cases, of ulterior mischief—of more extensive implication of the bone and periosteum. In the treatment of periostitis, we would rather try strict antiphlogistic treatment and incision first; and, if these failed, we would reluctantly be driven to mercury. It is with node as with some forms of the pustular and tubercular eruption: if we are too easily persuaded to introduce mercury, we not unfrequently complicate the case still farther, and diminish the chances of the unfortunate patient.

---

### XXIX.

### FRACTURE OF THE PATELLA AND NECK OF THE FEMUR.*

IT is well known that, if one patella be broken, there is a great liability to the same accident on the part of the other patella. How is this to be explained? Some have supposed that the cause consists in an unnatural brittleness of the bones of the individual affected. Sir Charles Bell takes another view of

---

* Ibid.

the matter. In an interesting clinical lecture, reported in our weekly cotemporary, he observes that the second accident is the result of the first. The patella being broken, that leg is rendered weaker, the patient is more liable to slip, and, in attempting to recover himself, he principally employs the other leg, the patella of which is exposed to the sudden action and whole force of the extensor muscles of the thigh, and snaps in consequence.

Why does not the patella commonly unite by bone? Because, says Sir Ch. Bell, when broken by the action of the muscles, there is no direct violence, consequently little inflammation or effusion of coagulating lymph, but, instead of it, merely an increased secretion of synovia.

When, however, the fracture is produced by direct violence, there is much more inflammation; and it is of great consequence to know this, and to act upon our knowledge.

*Case.* A coachman was taken into the Middlesex Hospital, with the patella broken by a blow. The house-surgeon, acting as he was accustomed to do in other cases, applied a bandage, and in twenty-four hours such inflammation had arisen in the joint, that nothing would subdue it, and the man died.

In these cases of patella broken by violence, the inflammation may be the means of producing ossific union.

Sir Charles Bell remarks that the neck of the femur may be broken in three ways.—1, In old age the bones become brittle, and an old lady, tripping upon her carpet, falls upon her hip, and dies from the injury. On examination, the neck of the bone and trochanter major are found "broken, like a tea-cup." 2, If a person fall with his foot in a hole, or jump out of a window, the neck of the bone is broken off in the joint, and the fracture being within the capsule, union will not take place, because, says Sir C. Bell, there is not sufficient inflammation. 3, Finally, the bone may be so broken, that the head and neck are wedged into the shaft, constituting a common fracture, which is followed by consolidation and re-union. To recapitulate; we may have a fracture in old age from a blow on the trochanter—fracture within the capsule—and, thirdly, fracture external to the capsule.

Sir Charles Bell gives an useful caution to surgeons, respecting shortening of the lower extremity after an injury to the hip. A person receives such an injury, and the surgeon, on a careful examination, can discover neither dislocation nor fracture. The case goes on, and, in a month or two, the patient hobbles about with a shortened limb, when some goodnatured friend informs him that you have treated the case improperly. The surgeon should always keep in mind this shortening, or apparent shortening, after inflammation, and mention the liability to it at an early period. It is too late to explain it when it has once arrived.

---

### XXX.

### Clot Bey on the Guinea-worm in Egypt.*

M. Clot, with his Egyptian pupils, appears to be exciting a great sensation in Paris. He is exhibited in the Institute and Académie—lionised at the hospitals—and a lengthy contributor to the journals. The French savans are delighted with the idea, that a Frenchman is active in re-civilising the land of the Pharoahs and the Ptolemies, under the influence of the extraordinary barbarian who now rules it; and in some of the periodicals, we find their enthusiasm getting vent in ejaculations of astonishment, and in dreams of the resuscitation of Egyptian grandeur. We will confine ourselves to the more sober task of extracting some particulars from M. Clot's communications.

The Guinea-worm was rarely observed in Egypt prior to the conquest of Sennar by Mohammed Ali. It has

* Annales de la Médécine. Aôut, 1832.

subsequently become very common, especially since numerous caravans from Ethiopia have arrived at Cairo, and the Ethiopians have been freely incorporated with the Arab regiments. When M. Clot went first to Cairo, in 1825, he found nearly a hundred persons affected with the Guinea-worm, in the hospital at Abow-Zabel. They were placed by the Government under the charge of two Arab surgeons, who were thought to be most acquainted with it, and most likely to treat it successfully. M. Clot contented himself, for some time, with watching their practice; but, finding it grossly empirical and extremely unsuccessful, he sent the Arab surgeons to the right about, and confided the patients to properly-qualified assistants.

We need not give M. Clot's description of the worm. It is familiar to the reading part of the profession. The length of the worm in Egypt varies from six inches to four feet—the longest that M. Clot has observed. He has obtained no certain information on the causes of its production or appearance. The inhabitants of Cordofan, Sennar, and Darfour attribute it to the heavy rains of April, May, and June. They think that it is a small animalcula, which attaches itself to the skin of those who bathe in the waters, but no person could affirm that he had seen such an animalcula. Though commonly observed amongst the negroes, M. Clot has seen it in a great number of Arab soldiers incorporated with the negroes, and in two Europeans. Dogs brought into the hospital have been attacked with it. It is seen in all parts of the body, but chiefly in the lower extremities. The same individual may suffer from several at the same time; indeed, so many as ten or twelve were not unfrequent. A friend of M. Clot's, Dr. Marrudri, who made the campaign of Cordofan with Defterdar-Bey, was successively tormented with twenty-eight.

The presence of the worm is detected in various ways. When placed superficially beneath the skin, it occasions a painful itching, which sometimes shifts its place, and the seat of the disease is transported to another part. In other cases, the worm may be distinguished in the form of a spiral cord, winding round the limb, and looking like an inflamed lymphatic or a vein. When present in parts not furnished with soft tissues, as the fingers, the articulations, &c. it occasions very severe suffering. When deeply placed in fleshy parts, it gives rise to an indolent swelling, which may remain for several days, and even months. In all cases, when it is about to make its exit, the pains become more intense, general symptoms arise, the part inflames, a small abscess forms and breaks, and a larger or smaller portion of the animal escapes. Sometimes the swelling is greater, and the whole of the worm is found rolled up in it. In other cases, which, however, are uncommon, the worm is not seen at first, and doubts of its existence may be entertained; in a few days afterwards it shews itself, or gives rise to a fresh abscess, more or less remote from the former. The suppuration from these abscesses is thin and serous.

The treatment varies with the part occupied by the worm, the manner in which it presents itself, and the symptoms which it occasions.

In ordinary cases, it is better to let the worm issue spontaneously; but, as soon as a part of it makes its escape, it must be tied by a piece of silk to a small cylinder of diachylum, round which it is to be rolled, a moderate degree of traction being exercised on the portion still imbedded in the body. The two extremities of the cylinder are made flat, and fixed in the vicinity of the abscess, on which is to be applied some simple dressing, or a light poultice, according to the amount of irritation. The worm is drawn out a little at each succeeding dressing, till the whole of it is removed, great care being taken not to break it, as this accident protracts the period of treatment.

If the worm does not soon make its way out spontaneously, and if placed so superficially as to be felt or seen, M. Clot has often made an incision on its track, seized it as near its centre as possible, tied it in the manner already mentioned, and thus drawn out both ends at once.

When the animal is deeply-seated, there is sometimes great swelling of the limb, deep abscesses form, sinuses result, and serous discharge continues for several months, without the worm making its appearance. In these cases, M. Clot employed general and local antiphlogistic measures, with benefit.

Two patients, in one of whom the worm was in the fore-arm, and in the other in the tibio-tarsal articulation, suffered the most excruciating pain, which produced cramps and convulsions. M. Clot relieved them by the cautery, after having vainly tried antiphlogistics, narcotics, &c. M. Clot has seen several patients with deep abscesses die, without the worm making its appearance. M. Clot has tried various remedies empirically, without effect; among these he enumerates mercurial frictions, liniments, the liquor of Van Swieten, oil of bitter almonds, sulphur, &c.

M. Clot is inclined to believe in the communicability of the disease by contagion. Thus, it is only seen among those Arabs who are in communication with the negroes: and M. C. has observed that it becomes less severe, less frequent, and at length ceases altogether, in proportion as the period of the incorporation of the negroes with the Arabs in the regiments is lengthened. For several years, no case of the kind has been seen in the hospital, no incorporation of negroes with the army having taken place during that time.

M. Clot concludes the memoir by the recital of some cases, and the insertion of reports from other surgeons, stationed at Sennar, Cordofan, &c. We shall content ourselves with glancing at one or two facts, that bear on the preceding general description.

*Case* 1. A negro of Darfour, æt. 25, who had served with the Egyptian troops for seven months, was admitted into hospital on the 2d April, 1825, with swelling of the scrotum, accompanied with fever. He was placed in the venereal wards, under the supposition that his disease was syphilitic. A poultice was applied next day, and he was bled in the arm, and the applications were continued for ten days. A larger swelling then appeared on the right side of the scrotum; it was opened, and, to M. Clot's surprise, a guinea-worm was discovered. The practice already described was employed, and the worm was extracted entire; it was twenty-three inches in length.

*Case* 2. M. Dussap, chief of the medical staff of the army of Egypt, had, in 1822, the charge of upwards of 400 persons, affected with Guinea-worm, in the Hospital of Souan. He himself contracted the disease. It commenced by a painful itching on the dorsal surface of the first phalanx of the index finger of the left hand. A vesicular tumefaction, with burning pain, succeeded, and increased daily. The whole limb became implicated, and the hand especially was the seat of such violent pain, as prevented, for many days, an instant's repose. The nature of the affection was not suspected, and it was treated on general principles. After some days the worm made its appearance, and was gradually withdrawn.

M. Dussap is a decided advocate of contagion, and, amongst other apparent instances of it, mentions the fact of several dogs, who fed on the poultices, &c. in the hospital, being attacked with the worm. It is remarkable, too, that the finger, the part of the surgeon most exposed to the influence of such a contagion, if it be one, was affected.

*Case* 3. M. Dot, a French teacher in the service of the Pacha, and constantly communicating with the negroes, observed a slight vesicle, with a red areola, between the first and second toe of the right foot. It was accompanied by trifling serous discharge and somewhat painful itching. At the end of fifteen days, M. Dot was obliged to discontinue his employment. Deep-seated pain and swelling of the foot now succeeded, and soon afterwards a portion of the worm made its appearance, and seven inches of it were drawn out, with great pain. The swelling increased, the pain augmented, and there was much fever. A second spot appeared above the external malleolus, and was soon followed by another worm, eleven inches of which were drawn out, with much suffering. The disease continued to make progress. Two other worms made their appearance on the external border of the tendo Achilles; two inches of one, and twenty-four of the other were extracted. The leg and foot were now enormously swollen, the pain intolerable, the fever very great. Deep incisions were made on the different points where the worms were found, much matter was discharged, and two portions of worms of different lengths were found. From this time the patient rapidly recovered.

M. Clot deserves the thanks of the Profession for this interesting paper. We trust we shall receive from the same hand, information on the diseases, or modifications of diseases observed in Egypt.

---

### XXXI.

### MR. ADAMS ON CONGENITAL HERNIÆ.*

#### I. *Congenital Enterocele.*

CONGENITAL hernia seldom becomes strangulated in very young children. But the object of Mr. Adams is to shew that it may be so; that practitioners should not only be aware of the fact but attentive to it; and that, if there be strangulation, they may boldly proceed to an early operation.

*Case.* W. F., aged 18 months, still at the breast, puny; was admitted into the Jervis-street Hospital, on Sunday evening March 18.

"The countenance was pale and sunken, the eyes languid, and surrounded with a dark circle; the child was restless, feverish, and thirsty, and every thing swallowed was instantaneously rejected from the stomach, while the bowels (which heretofore had been affected with an habitual diarrhœa) were now, and had been for the last two days, in an obstinate state of constipation. Upon exposing the abdomen, we observed that it was prominent, tense, and tympanitic, and the right inguinal ring appeared to be distended by the broad base of a pyramidal-shaped tumour, the apex of which was formed by the lowest part of the scrotum; this tumour had just the same tense feel that the abdomen itself had, and evidently contained intestine in a state of strangulation. The lowest point of the distended scrotum was intensely red from inflammation; this swelling, which constituted the hernia, was painfully sensible to the slightest touch, and the whole abdomen, both as to tension and sensibility, was nearly in a similar condition."

The child had been observed to be ill for 48 hours, the illness commencing with vomiting, followed by fever and constipation. The inguinal swelling had not been noticed till the day of admission.

The taxis and a tobacco enema were used without benefit. At 8 p. m. there was a consultation, and the operation was determined on, and immediately performed. We need not detail the steps of the operation; it was executed in the usual manner. The tunica vaginalis was completely divided up to the external ring, when the testis was disclosed below, and a portion of intestine of a deep maroon colour and polished surface above. The stricture was so tight that a director could not be passed through it. A probe was introduced into the abdomen, withdrawn, and the stricture divided by a small blunt-pointed bistoury, guided by the nail of the index finger of the left hand; the division was upwards and inwards. The child slept for three hours after the operation, and on awakening discharged flatus soon followed by a copious fæcal evacuation. The sutures were removed on the 4th day—no unpleasant symptoms whatever ensued—and the child is well, save that a hernia still exists, for which it wears a truss. Mr. A. expects that the truss will effect a permanent cure.

---

* Dublin Journ. No. vi.

II. *Congenital Encephalocele.*

This is a very dangerous, sometimes a very obscure disease. The protrusion takes place through a congenital opening in some part of the cranium, and may consist of cerebrum, cerebellum, or both, with their membranous envelopes. The following are the characters of the complaint, such as it has presented itself to Mr. Adams.

"From whatever part of the contour of the cranium, the tumour which constitutes the hernia projects, it is of an oval or spheroidal form, soft and colourless. It is attended with pulsations synchronous with those of the heart. These pulsations, when the patient is at rest, are sometimes indistinct, but are rendered very manifest both to sight and touch on the slightest exercise. The patient, when old enough to be able to give us an account of himself, says he never feels any pain in the tumour; if an infant, he seems to suffer no uneasiness, even when the swelling is subjected to gentle pressure; the size of this tumour is momentarily augmented by the efforts of coughing, sneezing, or even crying; during any respiratory effort, a blush of redness is seen rapidly to pass over it, through the skin, which is generally thin and semi-transparent where it covers the hernia. On carefully applying the fingers around the base of the tumour, the borders of the opening in the cranium, through which it has escaped, are easily felt, sometimes these borders are smooth and even; but I have in one case found them offering rough and elevated edges. The intellectual faculties in all the cases I have witnessed, at a period of life when these could be estimated, remained entirely unimpaired."

The characters of the complaint are not always so well marked. Thus, Lallemand relates a case in which the tumour was mistaken for a wen, and an operation was in consequence commenced. The congenital encephalocele does not occur at the fontanelles so frequently as might be supposed; it more commonly protrudes through some one point of the occipital region, in the median line. At birth, the tumour presents a distinct fluctuation; in the adult, the fluid has been removed and the prominence is softer. Encephalocele, like spina bifida, generally co-exists with hydrocephalus; but Mr. Adams thinks that spina bifida is the more dangerous of the two. Mr. A. has not seen, in any case, paralysis of the upper or lower extremities. The danger of these cases is chiefly confined to their early period, and from hydrocephalus being, in such cases, almost uniformly present, over-distention and ulceration of the hernial sac are the consequences to be dreaded.

"The chief bulk of the tumour will be found, on anatomical examination, to be formed of brain or cerebellum, and if examined, soon after birth, to be of a soft consistence, the convolutions flattened, and the whole structure infiltrated with water. The hernial sac is constituted by dura mater protruded before the brain, and lined internally with arachnoid membrane, fortified externally by that structure which in the perfect cranium constitutes its proper periosteum."

In one case, Mr. A. observed the tumour to project through the right portion of the os frontis. It has been seen at the root of the nose, where, for some weeks, a deficiency intervenes between the nasal processes of the frontal bone.

Mr. Adams observes that he has no confidence in the plan of treatment generally recommended by authors, namely, pressure. It is, he thinks, inapplicable to the first stage of the complaint, in which hydrocephalus so often co-exists, and useless in the latter stages. Mr. Adams speaks more favorably of frequent puncturing by a fine needle, after the manner recommended by Sir Astley Cooper in cases of spina bifida. He does not think a puncture made so carefully as to allow the limpid fluid to escape, but not to admit any air, a dangerous operation. The method of treatment which seems best, upon the whole, to Mr. A. is, to fortify the skin by astringent applications of oak-bark and alum; but, if over distention and ulceration of the the skin threaten, he would not delay the operation of puncturing the tumour.

In the second stage of the complaint, the only indications appear to be, to preserve the tumour from external violence. The medical treatment appears to consist, exclusively, in meeting any functional disturbances that may arise with appropriate remedies.

Mr. Adams relates some cases of the disease.

*Case* 1. "The head of the infant was the smallest I have ever seen; the forehead was depressed, and a tumour as large as an orange projected downwards and backwards from the occipital region. The chief bulk of this tumour was evidently formed by a fluid, as upon examining it, a distinct feeling of fluctuation was communicated to the fingers. The child for several hours after its birth seemed in a languid, dying condition; its respiration slow and imperfect; Dr. Macabe made a small puncture into the tumour, and a considerable quantity of limpid serum flowed out; after which the infant seemed to emerge from a state of stupor, its pulse became perceptible, it took some little nourishment, the puncture at once closed, but the child survived only nine days.

Upon dissection, I found that the sac (formed of dura mater) contained the entire of the cerebellum, and a great part of both posterior lobes of the cerebrum. These organs were softened, infiltrated with water, and surrounded with a quantity of limpid serum."

The calvarium was thin, but firm; the bones of the cranium touched in the whole line of the sutures, so that there was no fontanelle. The opening in the occipital bone, through which the herniary protrusion occurred, was comprised between the cuneiform apophysis in front, and the superior angle above and behind, the bone being thus cleft in the middle line by a large oval hiatus, two inches in its greatest diameter, and measuring transversely, in some parts, nearly an inch. This hiatus in the recent state, was divided into two by a membrane, the anterior division being the proper occipital foramen—the posterior larger, the ring through which the encephalocele had protruded.

*Case* 2. The head of the child was of the ordinary size; the tumour, of the size of a large orange, projected backwards and overhung the neck. The case was left to nature. The tumour slowly increased—ulceration gradually occurred and extended—a clear albuminous fluid escaped—the child was exhausted, and died convulsed at the end of the seventh week.

*Case* 3. Mr. A. has had for many years under his care a lad, now 20 years of age. The tumour, broad and flat, occupies nearly the whole of the right half of the forehead, and extends to the temporal fossa, occasioning hideous deformity. The roof of the orbit and superciliary arch are obliquely depressed to a considerable degree. The eye is much distorted, the cornea opaque. The tumour has an unequal surface, is soft, unresisting, pulsates synchronously with the heart, and receives an impulse on sneezing or coughing. The neck of the tumour is broad, the skin thick and pale, the edges of the bony ring, through which the tumour protrudes, marked by a rugged, uneven ridge. The mother of the lad states that he was born with the defect, and that he has never suffered other inconvenience than the affection of the eye. During the last seven years, the size of the face has increased, but the forehead and hernia have undergone no sensible alteration.

*Case* 4. A. B. æt, 6, a healthy-looking child, has at present a tumour, presenting the characters of encephalocele already enumerated. It is seated a little below the occipital protuberance, about the size of a hen's egg; its connexion with the head is narrow; the margins of the opening in the occipital bone, through which it passes, are well defined. The skin covering it is unequally thin and somewhat transparent; the surface irregular, and conveying the idea that the posterior lobes of the cerebrum are contained in the tumour; the head otherwise is well formed, and the fontanelles have been long completely closed.

Mr. A. saw this child very soon after its birth. The tumour was then as large, and nearly of the same form, as at present. The skin, however, seemed ready and likely to burst, and it was thought best to puncture it, which was done by introducing the needle where the skin was soundest and thickest. Half an ounce of clear fluid escaped, the sac became flaccid, but a tumour the size of a walnut remained, and formed the principal part of the protrusion. The wound was carefully dressed. It was necessary to repeat this operation seven times, once with a lancet; on that occasion only was there any subsequent fever or restlessness. Pressure was tried after one of the operations of puncturing; it produced convulsions, and was necessarily abandoned. Gradually the skin became thicker, the quantity of the fluid diminished, it was no longer necessary to repeat the operation, and the child is at present in the satisfactory state described at the commencement of the case.

This concludes Mr. Adams' paper. We have condensed it greatly, but we believe that we have presented most of what is important. It is a contribution to our stock of surgical knowledge, and not unworthy of the attention of surgeons.

---

### XXXII.

### Spontaneous Cure of Hydrocele.

The following case is related in our valuable contemporary of Dublin. It is extracted from a German Journal.

A man, aged 52 years, had been affected with hydrocele for many years. The tumour having been punctured, a quart of serum flowed out, and the testicles were found to be healthy. Nine months afterwards, the tumour had acquired the volume of a child's head. Dr. Krimer proposed to cure it radically by incision and extirpation of the tunica vaginalis, to which the patient consented. At the day appointed, Dr. Krimer and his colleagues having gone to the patient, were surprised at not finding the slightest trace of the hydrocele. The man said, that the evening before, having attempted to raise up a weight of 200lbs., he had felt a violent pain and a noise in the region of the inguinal ring. The pain continued most dreadful, as if the belly was being torn open. He then went to bed, passed much urine, and having fallen asleep after the pain, became somewhat easier, the next morning there was no trace of the hydrocele to be found. An ecchymosis existed over the left side of the scrotum. The spermatic cord and epididymis were varicose, the inguinal ring close, and there was not any trace of the liquor. He felt no pain. After a few days the ecchymosis and the varicose state of the cord disappeared under the use of fomentations.

There is a slight mistake, and we suppose it is in the translation. Dr. Krimer proposed, we are told, *extirpation* of the tunica vaginalis. We never heard of such an operation for hydrocele, nor for any thing else, nor is such an operation possible, without extirpation of the testis. It is not a very uncommon thing for a hydrocele to be accidentally ruptured, when the fluid escapes into the cellular membrane. The case before us does not seem to have been of this description; indeed it is somewhat marvellous, and looks as if it had been loosely observed or narrated.

---

### XXXIII.

### Bad Effects of Smoking Tobacco.

Such is the title of an article in our contemporary, the London Medical and Surgical. As our readers may like to know what these bad effects are, and as the article devoted to them is brief, though themselves may be long, we will gratify their curiosity by extracting it.

" The practice of smoking is now so general, that our atmosphere is strongly poisoned with tobacco-smoke. The air is sufficiently impure in this metropolis, without further contamination. At every corner we see a number of pipes or cigars, especially in the mouths of boys and young men. The bad effects of tobacco are first produced on the glands of the mouth, which form the saliva; these are excited and form an increased quantity of this fluid, which, instead of passing into the stomach, to assist in digesting the food, is expelled from the mouth. Great smokers have weakened powers of digestion, and are subject to bilious and nervous complaints, lowness of spirits, and diseases of the lungs and brain. The German medical writers allege, that of twenty deaths of men, between the ages of eighteen and thirty-five years, ten are destroyed by tobacco. This substance is one of the most powerful narcotics and debilitants; will produce stupor, drowsiness, head-ache, vomiting, and defective vision when used in excess; and, therefore, when long continued, and frequently employed, must be injurious to health."

If this be true, a pipe is indeed a Pandora's box, and woe to the hapless youth, be he of high or of low degree, who dares to open it. We must confess that we do not participate in the alarm of our esteemed contemporary at the contamination of our atmosphere. It will require many more pipes than are at present in circulation, to sully the smoky air of the Modern Babylon. We should like to know what are the diseases of the lungs and brain to which great smokers are subject, and how the German writers discovered, that ten out of twenty deaths of men, between the ages of 18 and 35, are produced by smoking. This latter is, we fancy, a piece of German fanfaronnade, and carries absurdity upon the face of it. In venturing to dissent from the sweeping condemnation of our contemporary, we beg to say that we are perfectly disinterested, being neither keepers nor frequenters of any "Cigar Divan," nor patrons of the "weed" in any shape. But really we think it hard that even an enemy should be treated unfairly; and old as the practice of giving a dog a bad name may be, we do not think that its antiquity consecrates its use.

If smoking were so prejudicial as its opponents assert, the world would ere this have been generally aware of it. The increased civilization and knowledge of the century have nearly banished excesses in wine and spirits from among the educated classes. Their reason and information convinced them that such practices were alike degrading and destructive. But tobacco-smoking is certainly on the increase, and we repeat, that the common-sense of mankind would speedily determine its injuriousness, if it were really injurious in a very perceptible degree. We say nothing of the custom, as a custom; it may be beastly, intolerable, or what not, but we feel well assured that it is not so pernicious as those who dislike it would seem to imagine.

---

### XXXIV.

### COMPOSITION OF THE BLOOD IN JAUNDICE.*

MR. KANE has examined the blood of a jaundiced patient. It had separated perfectly. Omitting the means by which he arrived at the results, we will merely give the latter.

The serum, when mixed with muriatic acid became grass-green in colour. Its sp. gr. was 1029.25. The quantity of blood sent weighed 3168 grains, and consisted of—

| | |
|---|---|
| Serum | 1420 |
| Clot | 1748 |

A quantity of serum having been carefully dried was found to consist of—

| | |
|---|---|
| Water | 893.1 |
| Solid matter | 106.9 |
| | 1000.0 |

A quantity of clot having been dried, was found to be composed of—

| | |
|---|---|
| Water | 656 |
| Solid matter | 344 |
| | 1000 |

---

* Dublin Journal.

The salts were found to be present in their natural proportion. The quantitative results of the further analysis were as follow:—

| | |
|---|---|
| Water | 762.28 |
| Albumen | 71.4 |
| Fibrine | 2.8 |
| Hematosine | 126.7 |
| Phosphuretted Fat; Oily matter & yellow colouring matter | 2.0 |
| Salts, loss, &c. | 34.82 |
| | 1000.00 |

"This analysis fully confirms the result of Lecanu as to the existence, in the blood of jaundiced patients, of the yellow colouring matter of the bile, and also as to the absence of cholesterine. In consequence of this latter principle being often a constituent, in minute proportion, of healthy blood, I sought for it carefully, but could not detect any trace of it. My results, however, differ in an important relation from those of Lecanu; he found that the quantity of colouring matter was less than in health; in the foregoing analysis it is very nearly the exact healthy average. I am not inclined to attribute great importance to the difference between our results in this respect, as the quantity of that principle varies in health between limits extending beyond both."

### XXXV.

The Principles and Practice of Obstetric Medicine. Parts XII. and XIII. By David D. Davis, MD., &c. &c. &c.

The two Parts before us are dedicated to the subject of menstruation. The physiology of this process is amply considered, and then we are presented with its aberrations. We shall endeavour to select some passages which may gratify the curiosity of some of our readers, or convey useful information. Our space is limited, and our notice must necessarily be brief.

### Influence of the Moon on Menstruation.

The apparent influence of the moon upon the tides must have been a source of astonishment in all ages; and it was not a very far-fetched idea which imputed to the same potent, yet mysterious agency the monthly flux of the female. The rigorous character of modern reasoning has, indeed, forbidden us to indulge in this philosophical dream, and we are forced to abandon the hypothesis of lunar influence. The following reasoning of Dr. Davis is conclusive against it.

"This theory of lunar influence in the production of the phenomena of menstruation is however liable to many and weighty objections. Experimental observations prove that the various relations of the moon to the earth's surface, are attended with no perceptible determinations or changes of situation of fluids when exposed to its action in artificial and graduated tubes. The column of mercury for example, in the barometrical tube, shows no sensibility to any possibly ascribable influence of the moon. Were this influence the actual exciting cause of the menstrual function, its effects would be uniform and universal upon all its subjects resident in the same latitudes, and therefore at the same time precisely similarly exposed to its agency; whereas the fact is, that women are known to menstruate indifferently at all ages of the moon. If, again, we suppose the agency of the moon to be really the cause of the menstrual tide or flux, as it has been called, we should naturally expect that the same position of that body would, at least in its relation to the same woman, be always productive of the same effects, and that no counter influence from any other quarter could ever by any possibility disturb it. But is this the fact? What is the history of the function in this respect? Why no such law prevails. The function, on the contrary, is capable of being disturbed as to the date of its periods by all sorts of influences. A familiar example will at once suffice to illustrate this statement. Suppose, then a young and vigorous female, a regularly menstruating subject, to become suddenly suppressed, in consequence of an imprudent exposure to the action of cold; and that this suppression, as frequently happens, should be attended by febrile and otherwise formidable symptoms. In that event, she would probably be induced to apply for the more or less early assistance of the medical attendant of her family. By bleedings, and other active measures, we will further suppose that gentleman to succeed in the restoration of the suspended function; but that that object is not attained till after the lapse of six or eight days subsequently to the suspension. Let it, however, be understood, that the function is eventually perfectly re-established, and the patient perfectly restored to her former state of health. Suppose our patient's constitution to have been at all times most delicately and regularly respondent to the law of the function; that, as in the hypothetical case of Haller, the first appearance of the secretion had always presented itself on the first day of her menstrual month from the commencement of the function to the date of its suppression. On the supposition of the moon being the cause of the function, on what day and in what week of the next, and all subsequent months, should the ordinary appearances of the function present themselves in future? Why surely on the first day of the first week, as in former times. That, however, will not be the case. The date of the next appearance will not correspond with those of its former periods; but with that of its restoration by the well devised and promptly-applied measures of the medical attendant. This fact, if well founded, and its accuracy admits of no dispute, is at least a proof positive, that the influence of the moon on the catamenial function, if it at all exist, must be of such a feeble nature as most easily to succumb to the operation of other influences; such other influences being competent not only to counteract and to disturb, but apparently to subvert, and summarily to dispense altogether with any supposed previously-existing influence of the moon. In the same circumstances of the moon, the same also should be those of all women equally and similarly exposed to its influence. But do we observe that such is the fact? Women on the contrary, notwithstanding the uniformity of the supposed influence, are subject to all possible varieties of circumstances as to the duration, dates of commencement and termination, modes of performance of the function, its effects upon other functions, physical and moral, as well as upon the general health of its subjects, the quantity, colour, consistence, sensible qualities, and all other appreciable conditions of the discharge. Aristotle on the generation of animals, B. iv. chap. 2. Galen De dieb. secretor. cap. 11. Ricard Mead, M.D. De Imper. Sol. et Lunæ, etc., Lond. 1746. Stahl. Theoria Medic. p. 386. Habenberg. Nov. Act. Erudit. Suppl. t. i. § 7. Shebbeare's Pract. of Physic, p. 261."

Our author notices, and at some length, the laboured theory of M. Le Cat. We have not room for it. But leaving speculation, most unprofitable as it always is on such subjects, let us advert for a moment to the secretion itself. It is curious to observe the opinions of the ancients on this head.

"Some of the best authorities amongst the ancients, among whom we should class Hippocrates and Aristotle, seem to have considered it as pure blood; and therefore make no mention of any properties which they imputed to it of a nature different from those of 'fresh blood from the victim,' as Aristotle expressed himself on the doctrine. The younger Pliny, on the other hand, speaks of its properties, as if they were possessed of the most poisonous malignity. His language is to the following effect: 'With respect to the menstruation of women, nothing could easily be found to cause more curious and fearful results. Unfermented wine-juice is soured by its vicinity to a person in that state. Corn is deprived of its nutritious qualities by it, grafted shoots are killed, and young garden-plants withered. The fruit of trees, on which menstruous women have sat, falls blighted. The brilliancy of mirrors is dimmed after reflecting their image, the edge of burnished steel is deadened, as is also the shining beauty of ivory. Hives of living bees are quickly deprived of their busy vitality. Copper and iron become rusted, in consequence of being exposed to the contact of the malignant fluid. Dogs that taste it become mad, and their bite infuses with it an incurable poison. They say, that even that small animal the ant can discover the vicinity of a menstruous woman, and that it throws away the provisions it chanced to be carrying at the moment of making that discovery, not taking to them again. This evil of such a nature and magnitude happens to women every thirty days; but prevails to a greater extent every third month. Some, however have the menses oftener than monthly, others have them not at all. These latter, however, do not bear children; for this periodical flux is the matter which assists the male in the act of generation.—Plin Secund. Natural Histor. tom. ii. lib. 7, cap. 15, art. 13. In the law of Moses, the secretion incident to the function seems to have been considered more in the light of an infirmity of nature and of offensiveness to our senses, than as being possessed of the malignant properties ascribed to it by other ancient writers.—Levitic. xv. 19—38. Isaiah, xxx. 22. Ezekiel, xviii. 6. In hot climates it is indeed probable that the menstruous secretion is very liable to become acrimonious under certain circumstances of inattention to the duties of cleanliness. Hence, no doubt, the origin of the strict regulations contained in the Mosaic ritual, and imposed upon the females of the Jewish nation during and for some days subsequently to the period of the function. The personal habits of the native women of India are such as to lead to the same conclusion. The frequency of their ablutions is such as scarcely to be credited, excepting upon the most veracious testimony."

Such is the unphilosphical nonsense of ancient philosophers. It was, indeed, a benefit to the human mind, when Lord Bacon broke the neck of antique sophistry. It is generally believed that the menstruous fluid contains no fibrine. There have not been an adequate number of experiments to determine this satisfactorily. Mr. Brande's analysis appears to deserve most confidence.

"Whilst engaged (says he) in observing the colouring matter of the blood, I received from Mr. Wm. Money, House Surgeon to the General Hospital at Northampton, some menstruous discharge collected from a woman with prolapsus uteri, and consequently perfectly free from an admixture with other secretions. It had the property of a very concentrated solution in a diluted serum, and afforded an excellent opportunity of corroborating the facts respecting this principle, which have been detailed in the preceding pages. Although I could detect no traces of iron by the usual mode of analysis, minute portions of that metal may, and probably do, exist in it, as well as in the other animal fluids which I have examined; but the abundance of the colouring matter in the secretion should have afforded a proportionate quantity of iron, if any connexion had existed between them. It has been observed, that the artificial solutions of the colouring matter of the blood invariably exhibit a green tint, when viewed by transmitted light. This peculiarity is remarkably distinct in the menstruous discharge. I could discover no globules in this fluid, and although a very slight degree of putrefation had commenced in it, yet the globules observed in the blood would not have been destroyed by so trifling a change."

M. Desonneaux concludes that, in some women, the menstrual fluid does contain fibrine. Dr. Davis criticises his opinion, and believes that, in a healthy condition of the secretion, fibrine is never present.

### Comparative Value of Life in Males and Females.

The investigation of statistic tables has produced results that, to the majority of men must be totally at variance with preconceived opinions. We can only be guided by evidence, and, though the evidence on the relative mortality of the sexes, and of the different classes of society is certainly yet imperfect, there seems good reason to believe that it approaches to the truth. That evidence leads to the conclusion, that the value of life of the female is greater than that of the male. It is not merely greater at particular ages, but at all ages—in childhood, maturity, and senectitude. That evidence proves, also, that the epochs of puberty, and of the cessation of the catamenia, are not so destructive to the female as is commonly supposed. The rate of mortality between the ages of 40 and 50 is much greater in men than in women, and the balance in favour of the latter is equally striking at the age of puberty. We will not stop to enquire why this should be; suffice it that it is. When we look at the habits of either sex, the riddle appears to be partly solved. From the cradle almost to the grave, the life of the male is more stormy—more subject to the influence of destructive agents than that of the female. It is the male who is chiefly occupied in the harassing businesses of life—convulsed by political excitement—exposed to the brunt of war and the caprices of the ocean—liable to accidents—and, by the usages of society, tolerated in excesses. Hesiod observed, many centuries ago, that the men were the working bees, the women the drones that fattened on the honey. Certain it is, that woman, whom the fiat of the Creator made, by the weakness and the peculiarities of her physical organization, the minister to man, finds in that very weakness her own best safeguard, and outlives her tyrant or her master.

Let us look at some of these statistic calculations.

Mr. Finlaison, the actuary of the national debt, observes that, in every census, there are found alive many more females than males, while, in the births and baptisms for every town, district, or kingdom, there is invariably produced at least 105 boys for every 100 girls. M. de Parcieux and Mr. W. Kersseboom have pointed out this difference. The result of the data given by the latter may be briefly stated to be this:—If there were ten classes of children at each age, the first under one year old, the last aged nine, and if the mean duration of life, which it was found each individual in a class had ultimately attained, were separately set out, the sum of the existence obtained by ten boys would be 369 years, and that of girls 402.5.

" In certain observations on the separate mortality of males and females at Chester, as set forth by Dr. Price, the same circumstance occurs. Taking the total existence of the first ten ages as before, there is for the boys 394.9 years, and for the girls 441.62. Yet Dr. Price, in like manner, disregarded the disparity. In other observations, taken at Montpellier, the fact was once more affirmed: the existence of ten males, as before, being 396.79 years, and of ten females 424.69. Again, on the whole population of Sweden, it was in like manner established; ten male children having 447.63, and ten females 471.26 years: and lastly, two additional tables have very recently appeared, showing the mortality of males and females respectively, both for the city of Amsterdam and the city of Brussels, for the males 397.97, the females 412.95. So that if the existence of the male children be represented in each of these six instances by the number 100 000, that of the females will by proportion stand as under, viz. anciently in Holland, 109.079; at Chester, 111.831; at Montpellier, 107.031; in Sweden, 105.279; in Amsterdam, 112.005; in Brussels, 103.764. All these results, it will be remembered, except the first, are founded merely on statistical data. But the superiority of female life is evident in every instance. Finlaison's Reports on the Evidence and Elementary Facts, on which the Tables on Life Annuities are founded, p. 17, Lond. 1829."

Mr. Finlaison's own calculations will, he hopes, remove all doubt whatsoever on this subject. They shew that, except under the age of 12 and above the age of 85, extreme periods, at which, perhaps, no distinction of mortality is apparent, there is at every other period a remarkable and decided advantage in favour of the female. This is first most evident about fourteen, after which the mortality among the female sex is observed to proceed onwards to the age of fifty-five, with the slightest imaginable increase, contrary to the received notions respecting nursing and child-bearing. Above sixty, the female mortality advances more rapidly, but is always, until the age of eighty, at least, very decidedly less than that of the males.

The mortality of males, from the age of 14 to that of 23, presents a remarkable increase, much greater proportionately, than that of females at the same period. This is probably owing, as Dr. Davis remarks, to the greater opportunities for indulgence, of all kind, offered to the male and accepted by him. Subsequently to the publication of Mr. Finlaison's Report, the Directors of the Eagle Assurance Association have introduced a considerable difference in the purchase-price of their policies, in favour of the supposed greater average value of female life; but the example has not been followed by any other life assurance society.

The facts already stated shew satisfactorily enough, that the establishment of the catamenia is not a source of danger to the female, that is, her mortality at that period, as at every other, is less than that of the male. The cessation of the menses has always been looked on as dangerous to woman's life, more dangerous than their establishment. Let us look at the evidence of facts on this point also. Are women liable to a greater mortality at this period than the male sex at the same age, or are women subject to a greater mortality at this than at any other period of their own lives? We must again refer to the calculations of Mr. Finlaison. The advantage in favour of female life, he remarks, is first most evident about 14, after which the mortality in the female sex is observed to proceed onwards to the age of 55, with the slightest imaginable increase.

"The following calculations of the rate of mortality of the government annuitants are made on a scale of 100,000 lives of members of both sexes and of all ages. The results, which are especially illustrative of the immediately current part of our inquiry, are distributed into three epochs of five years each, inclusive of the median line of life in both sexes, and in the female of the period of cessation of the menses. During each of these epochs, the rates of mortality of each sex are given as follows in their respective progressions. Out of 100,000 members alive of all ages, there will die in the five years after the age of 35, i. e. between the ages of 35 and 40, the latter inclusive, of males 7,042, and of females 5,738; in the five years after 40, i. e. between the ages of 40 and 45, the latter inclusive, there will die of males, 6,959, and of females 6,889; and after the age of 45, i. e. between the years of 45 and 50, the latter inclusive, there will die, of males 10,381, and of females 7,714."

Thus, in the government annuitants, there is less difference between the mortality of males and females, from the age of 40 to 45, than either before or after that period, a fact which goes to prove the prejudicial influence of the cessation of the catamenia. But some other calculations have recently been published at Brussels, by Messrs. Quetelet and Smits, in a pamphlet entitled, Recherches sur la Reproduction et la Mortalité de l'Homme, aux differens Ages, et sur la Population de la Belgique, 1832.

"A government order was issued in 1828 for a census of the entire population of Belgium, with directions that it should include returns of all the births, deaths, marriages, and changes of residence of the people during every year, and accompanied by an intimation that a similar assessment would be ordered for every ten years. The order for the first census was carried into effect towards the end of the year 1829. It is a peculiarity of this assessment that it has furnished distinct tables of results on all the points which it was charged to report upon for two classes of population, viz. for residents of towns and for those of country districts. Now it happens that, on these separate returns for the towns and for the country districts, its reports of the mortality of the different sexes exhibit a most extraordinary discrepancy with all former results on subjects of this kind. In Messrs. Quetelet's and Smits returns of the country mortality, the female deaths present an excess over those of the males during the three series of years corresponding with those just quoted from Mr. Finlaison's reports, in the proportion of something more than 24 to 18. Making 100,000, as in Mr. Finlaison's calculations, the numerical basis of their operations, the comparative mortality of the two sexes for the country districts of Belgium, as given by Messrs. Quetelet and Smits, are as follows; viz. out of 100,000 persons of both sexes alive, the rates of deaths within the five years between the ages of 35 and 40, the latter inclusive, are, of males 4,681, and of females 8,071; in the five years between the ages of 40 and 45, the latter inclusive, of males 5,975, and of females 8,536; and in the five years between the ages of 45 and 50, the latter inclusive, of males 7,692, and of females 8,056. Thus we see represented a most remarkable excess of female mortality in the country districts of Belgium; whereas in all the towns of the Netherlands, the returns would seem to correspond at least in principle with what has hitherto been considered in the light almost of a general law of mortality. Accordingly out of 100,000 inhabitants of the towns of Belgium, the rates of deaths in five years after the age of 35, i. e. between the ages of 35 and 40, the latter inclusive, of males 7,189, and of females 7,679; between the ages of 40 and 45, the latter inclusive, of males 8,894, and of females 7,153; and between the ages of 45 and 50, the latter inclusive, of males 8,678, and of females 8,062; giving a total of mortality for the three series of years together of 24,761 males and 22,894 females. But the excess of the mortality in some particular towns of Belgium very greatly surmounts the average results of Messrs. Quetelet and Smits. The author is indebted to the further kindness of Mr. Finlaison for the following quotation from a table of the average mortality of Ostend, constructed from the best possible materials by that gentleman himself, whilst recently residing temporarily in that town. For the convenience of an accurate comparison with the results of the foregoing calculations, the operation is given on the same numerical scale of 100,000 lives. With that understanding, the rates relatively of the male and female mortality at Ostend, during the tract of years intermediate between the ages of 35 and 50, as made out by Mr. Finlaison, and believed by him to be scarcely liable to the possibility of error, is as follows:—Out of 100,000 souls of both sexes and of all ages the rates of deaths after 35 years of age, i. e. between the ages of 35 and 40, the latter inclusive, are, of males 8,041, and of females 6,665; between the ages of 40 and 45, the latter inclusive, of males 11,107, and of females 7,094; and between the ages of 45 and 50, the latter inclusive, of males 13,079, and of females 8,188. From these results it follows, that the difference in the value of life in Ostend against the male and in favour of the female, at a period of life, inclusive in both sexes of the commencement of its decline, and in the female of the cessation of the menses, is in the very remarkable proportion of 32 to 21."

We are anxious to put the whole of the case, as stated by Dr. Davis, before our readers, the investigation being equally curious and instructive. It is unpardonable, now-a-days, for any educated medical practitioner to be unacquainted with statistical calculations of the value of human life. Dr. Davis concludes the subject by quoting the sentiments of M. Desonneaux. The facts brought forward by that gentleman are somewhat too vague to command implicit belief; but as they are, to a certain degree, borne out by the tables and calculations of Mr. Finlaison, already noticed, it may be right to notice them here. M. Desonneaux, it will be seen, is not one of those who consider the epoch of the cessation of the catamenia as on the whole a fatal one. M. Desonneaux observes that in an essay read by M. Bensiston de Chateauneuf to the Academy of Sciences, in 1818, but not published, "On the Mortality of Women at the Age of between Forty and Fifty," some facts are related, which he thus transcribes :—

"From the forty-third to the sixtieth degree of North latitude, along a line extending from Marseilles to St. Petersburgh, passing by Vevay, Paris, Berlin, and Stockholm, it is a fact that at no epoch of female life, from the thirtieth to the sixtieth year, are we able to discover any increase in the mortality of women, beyond what should be attributed to the natural progress of age ; whereas at all epochs of male life, from the age of thirty to that of seventy, we observe a greater rate of mortality of the male than of the female sex. This excess is most remarkable between the ages of forty and fifty. It results from these new observations, that the age of from forty to fifty is more truly critical for men than for women; and that seems to be the fact, whatever be the kind of life they lead, and whether they live in society or retirement, whether in the camp or in the cloister. Inasmuch, however, as it cannot be denied that a certain number of women die between the ages of forty and fifty, IN CONSEQUENCE OF THE REVOLUTION WHICH TAKES PLACE IN THEIR CONSTITUTION AT THAT PERIOD, a cause of mortality which does not exist in the other sex, what would be their decrement of numbers, already actually inferior to that of the male sex during the same period, and what might be expected to be the strength and duration of their lives, did they possess the additional privilege of not being subject to this law."

The whole of the documentary evidence is before our readers. It will require accurate observation and authentic tables, conducted and constructed in various places, and at various times, to determine in a satisfactory manner, the actual value of male and of female life. The contradictory facts observed in the towns and country parts of Belgium, show that we are far from comprehensively informed, and that we cannot yet venture to generalize or average. We trust that philosophic men will devote some of their time, and their attention, to the investigation of the effects of natural laws and human occupations on human life, as evinced by its value at different ages, in different sexes, and in different countries.

---

### XXXVI.

### ON HYBERNATION.

IN a very interesting paper on Hybernation, Dr. Marshall Hall remarks that there is a strong analogy between this singular state and that of ordinary sleep, the former being only a much exaggerated condition of the latter. In both, the necessity of breathing is lessened ; Messrs. Allen and Pepys first established the truth of this, in reference to ordinary sleep, and it is very probable that muscular irritability becomes at the same time more energetic, just in the same manner as in hybernation. These phenomena are more obvious in the ordinary sleep of the hybernating, than of any other animals ; for example, Dr. Hall observed a bat while asleep, and found that its respiration became very imperfect; that its temperature was only a few degrees above that of the atmosphere, and that it might be kept under water for 11 minutes without injury to life.—Similar observations were made upon hedgehogs : while active, their temperature was 95 while asleep, it was only 45°, that of the atmosphere at the time being 42° or 43°. The experiment was the more striking when one hedgehog was asleep, and another was lively, in the same box : the temperature of the former was ascertained to be 49°, and that of the latter to be 87°. Similar results were obtained in many experiments on dormice. These animals awake daily in moderate temperature, eat, and then fall into a state of sleep ; during which the respiration is very imperfect, and the heat of their bodies is little higher than that of the atmosphere. From these statements it appears that the ordinary sleep of hybernating animals is, at it were, intermediate, and forms a link in the chain of resemblance between the sleep of the other mammalia, and the state of perfect hybernation. When a hybernating animal is asleep, their need of respiration is much more lessened, and their temperature falls much lower than in their active state; now this sleep probably passes into true hybernation as the blood which circulates through the brain becomes more venous, and as the irritability of the heart becomes more exalted. Our readers will now perceive the great importance of ascertaining exactly the state of hybernating animals, in reference to sleep. When observations on the temperature of these animals are very carefully made, we have seen that, in the course of an hour or two, their heat may vary 30 or 40 degrees. The experiments of Mr. Hnnter, and more recently of Dr. Edwards, are faulty in this respect; as we are not informed whether the animals were quite lively, or dormant, or recently recovered from a dormant state: the hybernating animal, in a state of activity, is quite another vital being, from the same animal in a state of dormancy.

1. *Of the Respiration during Hybernation.*—That this function is very nearly suspended, is inferred from the following facts:—we cannot detect any movements of breathing; the air of the pneumatometer, in which the animal is confined, is scarcely changed; the temperature of the animal is the same as that of the atmosphere; and lastly, it is capable of supporting, for a great length of time, the entire privation of air. When a bat, or hedgehog, in a state of hybernating torpor, was carefully watched, no alternate movements, either of the chest or of the abdomen, could be perceived; but the least touch, the slightest shake, immediately caused the bat to commence the alternate acts of breathing, and the hedgehog to take in several deep and onerous inspirations. As to the effects on the air in the pneumatometer, the result of Dr. Hall's enquiries is, that a bat in a state of hybernation, which was kept in for ten hours, did not sensibly affect the air; whereas the same animal, when active, absorbed or converted 5·8 cubic inches of oxygen into carbonic acid, in the course of one hour.

It may be a question, whether the slight changes which we find to have taken place in the air, during some experiments, are referable to the cutaneous respiration. Dr. Hall very properly remarked, that this point is not very easily settled, because we are not positively to infer that the play of the lungs has entirely ceased, although we cannot appreciate it; there may still occur a slight diaphragmatic breathing. In judging of the temperature of an animal in a state of hybernation, the greatest caution is requisite, to avoid all disturbance of it, as the least motion will sometimes induce respiration, and the consequent evolution of animal heat. Dr. Hall details the results of 32 observations; in 29, the temperature of the animal was precisely the same as that of the atmosphere; and in the other three, it exceeded the latter only by half a degree: we may therefore assume, as an established fact, that the heat of animals, during hybernation, follows that of the atmosphere. It is singular to observe the very rapid elevation of the heat, when there is the slightest restlessness of the animal; a change from 48°, to 80° or 90°, is often observable in the course of an hour. Dr. Hall considers that Dr. Edwards has fallen into a great mistake, when he compares the young of those animals which are born blind, and which, he discovered, were less capable of resisting cold than the young of other animals (vide Review of Dr. Edwards' Researches, in our last number) to the hybernating animals. Under no circumstances do the former possess the power of evolving sufficient heat to maintain their requisite temperature; whereas the latter, when in a state of activity, preserve a uniformly elevated temperature: it is only when the animal is disposed to sleep, whether ordinary or hybernating, that the temperature begins to sink. The last proof of the almost total cessation of breathing during hybernation, is the capability of the animal to live for a considerable period in noxious gases, and even under water. Spallanzani narrates an experiment, in which he confined a marmot and a bat for four hours in carbonic acid gas, and yet they lived. Dr. Hall immersed a hedgehog and a bat in water of 41°; the former was kept 22 minutes, the latter 16 minutes, under the surface, without any injury to them. Now these animals, when active, are as speedily asphyxiated by drowning as any other of the mammalia; at least, such seems to be the case, according to Dr. Hall's experiments, in which he found that a hedgehog was killed in three minutes, from the time of immersion. Our author, therefore, differs from Sir A. Carlisle, and Dr. Edwards, when they assert that "animals of the class mammalia, which hybernate, have at all times a power of subsisting under a confined respiration, which would destroy other animals, not having *this peculiar habit.*"

2. *Of the Irritability during Hybernation.*—The very circumstance of the animal's living when deprived of atmospheric air, is an argument that their irritability or tenacity of life must be much increased; and this is powerfully confirmed, by finding that the heart continues to beat regularly for at least 10 hours, after decapitation and the destruction of the spinal marrow. A comparative experiment was performed on a hedgehog in its active condition; the spinal marrow was simply divided at the occiput. The beat of the right ventricle continued upwards of two hours—that of the left one ceased almost immediately; the left auricle was motionless in less than a quarter of an hour, and the right auricle had ceased to beat long before the right ventricle. It has been often asserted that the contractility of the voluntary muscles is impaired during hybernation; this, Dr. Hall says, is quite erroneous, and the mistake has arisen from authors confounding the lethargy of hybernation, with the torpor which is induced by extreme cold. We have only suddenly to arouse a bat or a hedgehog from their hybernating state, and we shall find that the former speedily flies about with great activity, and the latter walks about and does not stagger. In short, the phenomena observed are similar to those of awaking from ordinary sleep.

3. *Of the Sensibility.* Dr. Hall states that all preceding authors have committed a great error, in supposing that the sensibility of animals during hybernation is diminished; in truth, it is quite as perfect as in ordinary sleep; the slightest touch of one of the prickles of a hedgehog is sufficient to arouse it; and the gentlest shake causes a bat to respire. The sensorial functions are, indeed, nearly suspended; if a hedgehog, in its active state, be thrown into water, it immediately uncoils itself and betakes to swimming—in the hybernating state, on the other hand, no fear appears to be excited, and the animal would probably remain still and quiet for a very considerable period, if its sensibility were not acted upon by the contact of the water.

4. *Of the Circulation.* Dr. Hall, by a nicely-contrived experiment, was enabled to examine the circulation in the wing of the bat, in its state of hybernation; he found that, although the animal did not perceptibly breathe, the circulation continued uninterruptedly; the number of pulsations in the minute was about 28°. All the blood is venous, and the curious fact is, that the left side of the heart, and also the arterial system, are now veno-contractile. This phenomenon is one of the most remarkable in physiology; it accounts for the life of the animal being independent of respiration, and is, in short, the key to the right explanation of the susceptibility of some animals taking on the hybernating state, and of the insusceptibility of the greater number.

5. *Of the Digestion.* The bat does not shew any disposition to awake for the purpose of taking food during its continued hybernation; neither are any excretions passed. External warmth, or any excitement, is the only stimulus which arouses it. On the other hand, the hedgehog, if the temperature be about 40° or 45°, awakes every two, three, or four days to take food, and it then returns to its state of lethargy; under similar circumstances, the dormouse awakes daily. Hunger is, therefore, probably the stimulus which induces the animal to awake at intervals.

Having now considered the condition of the leading vital functions during hybernation, we can be at no loss to perceive the marked difference between this state, and that of torpor from extreme cold. We have seen that the muscles do not become stiffened and incapable of motion—that the nerves are not benumbed and paralysed, and that hybernation is a salutary change, and one conducive to the preservation of life; whereas continued torpor from cold, as is well known, generally proves fatal. Indeed we shall find that, when a hybernating animal is subjected to great cold, without any means of increasing and retaining its warmth, it often remains in a state of activity, while others of the same tribe, which are provided with wool or straw to make a nest for themselves, speedily become lethargic. It is, therefore, moderate, and not extreme cold, which disposes to true hybernation. During the Winter months, they seek some secure retreat for nests or burrows, or congregate in clusters; and, if the season is unusually severe, many are found to perish from the cold. Let these particulars be, therefore, well remembered, in order that we may not, in future, mistake the phenomena of torpor from cold for those of true hybernation. In one respect only do these two states agree, and that is, in the dangerous and often fatal consequences of overspeedy reviviscence; if the respiration of an animal, lethargic from hybernation, be suddenly restored and kept permanently excited, the effect is very often the death of the animal.

*Recapitulation.* The following conclusions contain the essence of Dr. Hall's observations:—

1. The natural sleep of hybernating animals differs greatly, but only in degree, from the sleep of other animals.

2. This sleep passes insensibly into the state of true hybernation, which becomes more and more profound as the blood loses its arterial qualities.

3. The respiration and evolution of heat are nearly suspended during hybernation.

4. The irritability of the heart and arteries is singularly augmented, so that they become veno-contractile.

5. The sensibility and general muscular motility are unimpaired.

6. The phenomena of true hybernation are very different from those of torpor from cold.

7. Severe cold, like all other causes of pain, rouses the hybernating animal from its lethargy, and, if continued, induces the state of stupor.

8. The phenomena of hybernation are attributable to the susceptibility of the heart and arteries to be stimulated by venous blood, and to the various organs of the body becoming much more irritable than during the state of the ordinary activity of the animal.—*Philosophical Transactions.*

---

### XXXVII.

### MARCH OF EPIDEMICS.

SCARLATINA has been prevalent in Great Britain for the last three months, and in Paris for the last six months, as may be seen by referring to our reports from the clinique of M. Bouilland. On perusing a late number of a German periodical, we find that scarlet fever appeared sporadically in May, 1831, at Konigsberg, and epidemically in December of the same year; it continued till April or May of 1832, when it totally ceased. We shall extract a few particulars on some of the leading features of the disease, as observed in Germany. In some cases there was no eruption, although the other symptoms, such as cynanche, the scarlet-fever tongue, desquamation of the cuticle, and consecutive dropsy were present; moreover, they occurred generally in houses where the eruptive disease actually existed at the same time.

In a family of three daughters, the middle-aged one was seized with cynanche maligna, accompanied with typhus fever; the eldest with mild angina tonsillaris, and the youngest, an infant 14 months old, with purging and feverishness. No sooner had they recovered from these ailments, than the youngest had dropsy, the middle one had otorrhœa, and the eldest violent convulsions. Now in none of these cases was there any appearance of exanthema or of desquamation; whereas, in another set of patients, the latter symptom, and also anasarca, followed attacks of "cynanche sine eruptione." The size of the scales thrown off during convalescence, was remarked to be generally in proportion to the darkness of the hue of the preceding eruption; where this had been purplish-red, the scales were large—where it had been pale and indistinct, they were small and furfuraceous. Swelling of the parotid glands, during the eruptive stage, was found to be an unfavourable symptom, as cerebral symptoms often attended it, and deep-seated ulceration followed in many cases.

The fever was generally the ordinary synochus; but sometimes a most violent form of synocha, with great tendency to inflammatory congestion of the head. The treatment of this latter fever required the utmost skill and exertion, because the transition from active phlogosis to typhoid depression was frequently very sudden and rapid, so that, while leeches and cold applications to the head were in use, it was necessary to exhibit stimulants inwardly.—*Medicinishe Zeitung.*

---

### XXXVIII.

### Radix Caincæ.

This Brazilian root has been highly lauded by some authors, especially by François (Journ. Générale de Médécine, Mai, 1830), as a diuretic and a sedative. The experiments lately instituted by Dr. Wolff, at Vienna, do not at all warrant the praises; in his practice, its effects were very analogous to those of the helleborus niger, exciting nausea, vomiting, purging, and only sometimes diuresis.—*Ibid.*

---

### XXXIX.

### Adhesion of the whole Pericardium to the Heart.

F. F. aged 26, was admitted into one of the Berlin hospitals under the care of Dr. Wolff. His symptoms, which had existed with varying severity for the previous six months, were a stitch in the præcordial region, palpitation and dyspnœa, amounting frequently to complete orthopnœa; his sleep was disturbed by sudden startings; he had a cough, with but little mucous expectoration; his urine was sparing in quantity, and of rather a brown colour. The complexion of the face was sometimes faintly livid. On attentively examining the region of the heart, it was found that its pulsations were so feeble, as not to be readily felt with the hand, and that, under the stethoscope, irregular, weak, and undulatory movements were perceived. The pulse was irregular, small, contracted, and frequent; no appearance was visible of any starting or pushing forward of the diaphragm, or of dragging inward of the lower ribs on the left side, synchronously with the diastole or systole of the heart. The case was supposed to be one of hydrops pericardii. After a very small bleeding, the infusion of digitalis, combined with nitre, was given with the most gratifying results, the patient being speedily able to lie and to sleep in the horizontal posture; but, unfortunately, the drug began to exert its narcotic and poisonous effects, and required to be discontinued. No sooner was this done, than the symptoms, in all their aggravation, returned, and the patient died in a few weeks.

*Dissection.* The lungs and cavities of the pleuræ healthy. The pericardium was found to be so closely adherent in all its extent to the heart, as to be separable from it only by the scalpel; the agglutination was firm and ligamentous; there was no appearance of any intermediate membrane, or of bands passing between the two surfaces, for, strictly speaking, there was but one membrane, or, in the language of the older pathologists, the pericardium was awanting.

*Remarks.* The symptoms deemed by Kreysig as characteristic of this disease, viz. the movement forward of the diaphragm and the pulling inward of the left lower ribs, synchronously with the alternate actions of the heart, are unsatisfactory and incorrect. Corvisart says that the absence of any strong palpitations, while the other signs of diseased heart exist, may lead one to suspect adhesion of the pericardium.—*Ib.*

---

XL.

CASES OF INSANITY, WITH POST-MORTEM APPEARANCES, By Dr. MACROBIN, Aberdeen Lunatic Asylum.

WE have selected the following cases from the reports transmitted to us by Dr. Macrobin, and regret that we have been unable to insert the others.—ED.

*Case* 1. Margaret Panton, æt. 70, from Aberdeen. Admitted 1808.

*Sectio Cadaveris,* 21*st Feb.* 1832, 12 *Hours after Death.*—The calvarium was thin, and its removal was effected with difficulty, in consequence of the very firm adhesion of the dura mater to the inner table, especially along the line of the longitudinal sinus, where the membrane was thickened and vascular; some patches of the membrane were left adhering to the occipital bone. A laceration, which had been produced in effecting this removal, allowed the escape of between four and five ounces of limpid, colourless serum, which had been deposited between the dura mater and arachnoid, and which lay over the surface of the right hemisphere of the brain; the dura mater was accordingly discerned collapsed, and thrown into folds. Upon reflecting this membrane (which otherwise presented a natural appearance), the whole cerebral substance composing the right hemisphere was discovered greatly compressed, and greatly diminished in bulk.

The convolutions were flattened and pressed together, and over a space of an inch and a half to two inches in diameter, and immediately above the lateral ventricle, they (the convolutions) had entirely disappeared, apparently from absorption, leaving an irregular, oval-shaped depression, the base of which consisted of the corpus callosum, the white colour of which was visible under the arachnoid and pia mater, which lined this depression, and which were much thickened, so that a layer of medullary substance, of three or four lines in thickness only, existed between the convex surface of the brain and the right lateral ventricle. There was another similar, but smaller depression or cavity on the surface of the anterior lobe of same hemisphere. In connexion with these appearances, the whole of the cerebral substance had undergone great compression from the presence of the fluid; the whole hemisphere having suffered a diminution to the extent of at least one-third of its ordinary bulk, and being sunk much below the level of the opposite hemisphere. There was no appearance of increased vascularity of the pia mater, and the substance of the brain generally presented a natural consistency and colour, with the exception of some venous congestion, the number of dark bloody points being very numerous.

There was no fluid over the left hemisphere, nor more than usual in either ventricle. Both choroid plexuses were turgid and hydatiform. The apex of the upper lobe of the left lung was tubercular, and the lower lobe of same lung more solid than natural, being without crepitus, and greatly infiltrated with serous fluid; and there were some traces of recent lymph, causing adhesion between the pleura costalis et pulmonalis. The right lung quite healthy. No disease of the heart or arteries, there being not the least tendency to ossification in them, or any other structure of the body.

Abdominal viscera sound.

The subject of the above appearances, a female in the lower ranks of life, had been confined to the Lunatic Asylum for upwards of 20 years. She had completely lost the use of the left upper extremity, which was quite rigid and bent, from contraction of the flexor muscles of the fore-arm and fingers. It is understood that she suffered some febrile attack (a "fever" the friends called it) about puberty, which recurred repeatedly, and which at length affected her mental faculties, and that the paralytic affection of the arm occurred about the same time; but little of the history can be relied upon previous to her admission in 1808, since which time she had continued to enjoy pretty good general health. Her insanity was marked by various hallucinations and illusions of the external senses, those of sight, hearing, and common sensation being perverted.

Her memory had also suffered, her memory of events since her attack being quite abolished, though she had a lively recollection of all the circumstances of her life previous to this. She was very loquacious, and highly irritable and ill-natured. The natural functions were performed in health to the last.

*Case* 2. David Bruce, æt. 47, from Aberdeenshire. Admitted 1st August, 1831.

*Sectio Cadaveris,* 16 *Hours after Death,* 4*th March,* 1832. *Head.* At the anterior part of the middle lobe of the right hemisphere, there was observed a space of about an inch square of the cortical substance in the state of distinct ramollissement, being diffluent and broken down, and converted into an ochre-coloured pulp, and which had stained the dura mater which covered it of a bright yellow, the proper membranes of the brain having been removed at this point by the morbid process. A similar change was observed to be going forward, to a smaller extent, on the convolutions of the anterior lobe. This softening and disorganization was best marked on the surface of the convolutions, diminishing in intensity as it extended inwards, and did not penetrate to the medullary substance.

The arachnoid, over the whole convex surface of the brain, and over the anterior as well as posterior lobes, was greatly thickened, and the pia mater was very vascular, which vascularity extended between the convolutions, and the membrane admitted of being easily removed.

The same membranes covering the base of the brain were not thickened. There was only slight effusion of serum over the surfaces and into the ventricles of the brain, not exceeding ℥ss. in the latter situation.

The subject of the above appearances was a labourer, and had led a sober and industrious life; and, previous to May last (1831), had exhibited no indications of mental disturbance nor morbid train of thought, though his bodily health had somewhat declined: but for two or three weeks previous to the accession of the maniacal paroxysm (which happened about the middle of May), he had began to exhibit a change of temper and disposition, being one time gloomy and melancholy, and at another irritable and excited. He continued to labour upwards of two months under violent maniacal delirium, when fatuity supervened, and it was then that he was brought to the asylum (1st Aug. last).

He was then unable to answer questions; though at times, if the question was repeated, and his attention roused by the speaker, he would seem to comprehend what had been said, for he would labour to give utterance to some words in reply, and probably, after the lapse of a minute, some answer might be obtained, while the words, which seldom amounted to three or four, were uttered slowly and with hesitation, and apparently with difficulty.

His aspect was very vague, and led to the impression that, in general, he did not comprehend what was addressed to him. His memory seemed to be nearly abolished. The pulse was feeble, and slower than natural. He was unwilling to move from his seat, or from the spot where he might be standing; and when he walked his gait was unsteady, and latterly he could not walk without staggering.

The appetite and the other natural functions, though pretty regular and healthy, were disregarded by the patient, and, unless when fed, he would never take food, nor even ask, or make signs that he wanted it. Once or twice during this state of fatuity, he exhibited some return of excitement, which was marked by singing, vociferating, and dancing, or rather whirling about.

He died 3d March, under appearances of much debility and emaciation.

*Remarks.* Some of these cases, as that of Hunter, Bruce, and Panton (with those of Simpson, Keith, Dawson, and Robertson, formerly reported), were to all appearance connected with inflammation of the membranes, as marked by the train of symptoms, and the morbid appearances ascribed by M. Bayle to chronic inflammation of the arachnoid and pia mater in his work on the Diseases of the Brain and its Membranes; and from whose researches we have pretty good evidence, that there is a considerable proportion of cases of insanity, (whether monomania or mania, seems to be a circumstance little connected with the *pathological characters)* arising from a state of increased action in the vessels of the arachnoid and pia mater.

I have at present several recent cases of insanity, where the physical symptoms leave little doubt of such a state being the cause of the mental aberration, while I have still a greater number where no such symptoms exist, but in which we find *nervous* excitement, without *vascular* excitement, the pulse every where being feeble, while any cerebral symptoms which may be complained of are only occasional, as vertigo, noises in the head, temporary headaches, &c. and generally much relieved on assuming the recumbent posture. This is more particularly the case with females, and those who have hereditary predisposition to insanity. The true pathology of such cases seems to be as little ascertained as that of many cases of hysteria, with which insanity is so frequently associated in the female constitution, and which seem to depend, in common with other affections of the nervous system, on a peculiar condition of the nervous organization, which, if influenced by particular states of its system of bloodvessels, as a necessary antecedent to disorder of its functions, is at least influenced by states of the vascular system, which, in most instances, escape our observation both during life and upon examination after death, and are certainly little, if at all allied to true inflammation. No doubt other diseases affecting the nervous system, as apoplexy and palsy, may be observed accompanied with a cold, pallid surface, weak pulse, &c. and that, when dissection will discover most indubitable signs of a previous state of vascular excitement; and it might probably, therefore, be alleged that the stage of excitement, in cases of insanity, had existed, though it had passed unnoticed, or that such might exist locally, and without materially affecting the general system. But if we have no characteristic *local* symptoms, and, in the absence of them, are not to judge of the nature of morbid actions by such *general* symptoms as may be present, and are to allow no weight to evidence derived from the obvious effects of remedial agents, and the absence of inflammatory injection or its proceeds, on inspection after death, then by what other means are we to arrive at probable conclusions, in regard to the nature of certain pathological causes? Do not most chronic inflammations of important organs betray, when accurately investigated, some degree of febrile action, and other appropriate symptoms?

The cases just alluded to do not improve under any system of depletion, but are most benefited by proper moral and mental discipline, along with a due attention to the state of the natural functions, but, unfortunately, remain benefited only so long as the application of such means can be enforced, since, from the predisposition—the great susceptibility to every passing impression, moral and physical, and the little resolution which such individuals are capable of exerting, we find them constantly returning to a state of mental aberration, and presenting a life spent between intervals of sound mind and healthy actions, and the feelings and the actions of insanity.

The former class of cases again requires a steady perseverance in antiphlogistic measures, limited, however, in general, to local depletion, and such other means, as do not depress too much the strength of the general system.

29th March, 1832.

---

### XLI.

### Aneurism by Anastomosis.

Dr. Macfarlane in his Clinical Reports makes the following remarks on the subject of nævus, and relates three cases not devoid of interest.

"That species of aneurism by anastomosis to which the appellation of nævus is applied, is a disease now often met with in children. During my attendance at the Infirmary, I have seen above thirty cases, the majority of which were treated as out-patients, and have had opportunities of comparing the merits of the different plans of cure which have at various times been adopted. In shortly noticing the size and other external characters of these tumours, I was careful also to preserve a description of their exact situation; and I find, in reference to this point, that more than two-thirds of them were confined to the anterior aspect of the body, and that considerably more than a half were situated on the head and face. All of these, except one, were observed at birth,—grew with greater or less rapidity, and varied in extent from about a quarter of an inch to fully three inches in diameter. They all projected more or less beyond the level of the surrounding parts: and, in a few cases, ulceration of the thin integuments with which they were covered took place, giving rise to troublesome hæmorrhage. In three of these there was gradually projected from the ulcerated surface a fungous tumour, which rapidly increased, assumed a pyriform shape, bled on the slightest touch, and appeared to possess all the characters of the original disease. When ulceration does not take place, then the superjacent skin, by the morbid enlargement of the subcutaneous vessels, is gradually and unequally elevated; but it is seldom that the diseased mass extends more than an inch beyond the level of the adjoining healthy parts. I have seen two cases, however, in which the disease did not increase in breadth, but continued to project, while the integuments were entire, so as to form livid and pendulous tumours."

Case 1. *Aneurism by Anastomosis—Ligatures—Convulsions.*

A child, nine months old, had a tumour, about the size of a grape, over the anterior superior angle of the left parietal bone, which had all the characters of aneurism by anastomosis. A needle, armed with a double ligature, was passed under its base, and each half of the swelling was tightly tied, so as to cut off its supply of blood. The child was teething: it cried bitterly, and was fretful and uneasy for several hours. During the following night it had an attack of convulsions, which continued for fifteen minutes, and returned with undiminished violence after an interval of two hours. The ligature was immediately removed, and the convulsions ceased. In four days the tumour sloughed, and a cure was speedily accomplished.

Case 2. *Aneurism by Anastomosis cured by temporary Ligature.*

Dr. Macfarlane was induced by the circumstances of the preceding case to leave the ligature only for a short period in another instance.

A child, 8 months old, was brought to the Infirmary to have a pyriform tumour removed from the edge of the under lip. It was, when observed at birth, about the size of a split pea, of a livid colour, and on a level with the surrounding integuments. It remained stationary for the first three months, after which time it began to increase rapidly, and to project in a pendulous form. The integuments were entire: the apex of the tumour, which was about the size of a walnut, was irregular and doughy; whilst the neck, which was not larger than a quill, was hard and smooth, and the pulsations of its vessels were distinctly perceptible. A broad ligature of tape was firmly applied, close to the base of the tumour, and removed in 24 hours. With the use of cold this temporary ligature was perfectly successful, the tumour sphacelating, and the lip cicatrizing.

We apprehend that this practice is more likely to succeed in cases like the preceding, where there is a narrow and distinct pedicle, than in others where the origin of the tumour is more diffused. It is sometimes difficult to include the whole of the disease by the ligature applied in the most efficient manner. Indeed, Dr. Macfarlane's third case is one in point.

CASE 3. *Aneurism by Anastomosis—incomplete Ligature.*

W. H. aged seven months, had a soft, unequal, purple-coloured tumour, about the size of half-a-crown, on the anterior surface of the left arm, two inches above the elbow-joint. It was elevated a little above the surrounding parts; and when firmly compressed, an obscure thrilling or slightly pulsatory sensation was perceptible. It was tied with a double ligature, as in the last case, and in six days the tumour separated. The exposed surface, which had at first a sloughy appearance, soon became clean and florid; and, except a small spot in the centre, it was evident that the diseased structure was completely destroyed. Here, however, a large spongy tumour formed, which was of a dark colour, and bled profusely. Pressure by means of a compress and bandage, the free application of nitric acid, caustic, &c., were ineffectual in checking its progress. The actual cautery was at length had recourse to; and by four applications of it, the morbid growth was destroyed, and a cure accomplished.

Whatever is capable of exciting inflammation in these vascular tumours, and of producing either ulceration or consolidation of their loose texture by the effusion of lymph, may put a stop to their progress, and ultimately lead to a cure. For this purpose I have used vaccination with success in five cases; and in one case, where the disease extended over the whole surface of the lower eye-lid, and where neither the ligature nor the knife could be employed without producing deformity, I succeeded in exciting inflammation of the tumour, by introducing a sèton close to its base, and retaining it till partial suppuration was established. In another case, where the disease was confined to the inside of the lower lip, the seton proved unsuccessful, and ligatures had to be employed.

There can be no question that the ligature, when applicable, is the least tedious and the most certain method of removing nævi. Next to the ligature probably comes excision. Vaccination we have tried, and seen tried without success. It is a pretty rather than an useful plan of treatment, at least we have found it such. We have not ourselves seen any bad consequences from the operation. Erysipelas followed in one child, but it did well.

---

### XLII.

### INJURIES OF THE HEAD.

"WHEN a large quantity of blood is effused under the scalp, its evacuation by incision is seldom required, unless for the purpose of ascertaining the state of the bone, and that only when symptoms of compressed brain exist. The extravasated blood will be slowly absorbed, and the detached scalp regain its former connexions, while the removal of the fluid by puncturing the tumour, will not unfrequently give rise to troublesome and extensive suppuration."*

Nothing can be more true than this observation. Most surgeons must witness cases of fluctuating tumour of the scalp after injury. For the most part there is little or no pain, nor any fever. But fever may be accidentally present, and from certain circumstances there may be some tenderness. The surgeon is then induced to puncture the swelling, fluid blood only issues, and a trou-

* Dr. Macfarlane's Reports.

blesome suppuration in the cavity ensues. We have seen this mistake made with this result. If the fluid collection of blood be small, it is well known how much it may simulate a depression of the bone. Blood is injected and coagulates in the surrounding cellular membrane, and the yielding fluid portion counterfeits depression. This mistake also is not uncommon. We know not whether fluctuating tumour of the scalp, apparently from blood effused, be common in newly-born children, but we have seen one instance of this description. The infant was one or two months old, the fluctuating tumour as large as an apple, and situated over the parietal bone; there was no pain, no cerebral disturbance. The labour had been difficult. But we will give Dr. Macfarlane's case.

Case 1. *Concussion—Effusion under the Scalp—Cure.*

A stout labourer, thirty-eight years of age, was struck on the left side of the head by a brick, which fell from a considerable height. He lay in a state of stupor for nearly an hour, when his sensibility was gradually restored; and in a short time longer he was able to return distinct answers to the questions put to him. He complained of vertigo, headach, and nausea, which were removed by venesection, and smart purging. On examining his head, the scalp was found distended by fluid blood. The swelling, which appeared to be covered by the tendon of the occipito frontalis muscle, commenced at the injured part where the integuments were not wounded, and gradually extended from the left to the right side of the head, and from the superciliary ridges to near the foramen magnum of the occipital bone. By the use of a strong lotion, composed of the murias ammoniæ and vinegar, this extensive effusion of blood was slowly absorbed, and without the occurrence of a single unfavourable symptom, the parts were restored to their former state in less than a month.

Dr. Macfarlane relates two cases of concussion, both of which were cured. In the first there was probably some extravasation, as the symptoms of concussion did not soon pass away, and on the day after the injury there was slight stertor. The symptoms did not disappear in less than a fortnight, though very active antiphlogistic treatment was employed. Dr. Macfarlane remarks that the symptoms of concussion often run so gradually and insensibly into those of inflammation that it is difficult to pronounce upon the boundary between them. This is true. There is, however, one good practical rule—to sin on the side of depletion. If the symptoms do not subside in a reasonable time, it is probable that either extravasation of blood or inflammatory action is present. Inflammation never proceeds to any extent without fever—a frequent pulse, a skin more or less warm, secretions more or less altered. This is the grand diagnostic mark of inflammation, and one that can seldom deceive.

Dr. Macfarlane observes, that bleeding from the ear is oftener met with in fractures of the base of the cranium, than in simple concussion of the brain. Out of four cases of concussion in which this symptom was present, and witnessed by Dr. Macfarlane, two proved fatal, and on inspection neither fractures nor laceration of the lateral or cavernous sinuses could be detected. Dr. M. considers, therefore, that both venous and arterial blood may be discharged from the ears as from the nose by laceration of its own blood-vessels. All who have seen many cases of injury of the head, will corroborate Dr. Macfarlane's view of the subject.

Case. *Concussion followed by Symptoms of Compression.*

"W. B., æt. 50, was admitted on the 31st of January, 1832, having received several severe blows on his head ten days before, which produced immediate insensibility, vomiting, and bleeding, from the left ear. He lay in this state for several hours, and then recovered so far as to be able to answer questions, though rather incoherently. In a short time, however, the stupor began again to increase, and, on his admission, the existence of partial compression was distinctly marked He was dull and drowsy, and when asleep his respiration was stertorous. It was only after shaking him, and speaking to him with a loud voice, that he could be induced to open his eyes, which had a vacant expression, the pupils being dilated and torpid. For the first two days no answer could be obtained to any question that was put to him, but after the repeated application of leeches and blisters to the head and neck, and the use of purgatives, he became more sensible, his pulse rose from fifty-two to eighty in the minute, but he was still incoherent. Calomel was given as an absorbefacient. When the gums were affected, the improvement became more decided, and, in ten days from his admission, the symptoms of compression had altogether disappeared. His mind, however, continued imbecile; his memory was much impaired; he could not recollect the names of his children, or of any of the objects around him with which he was most familiar, and he complained of deafness, headach, and vertigo. When dismissed on the 18th of February, his memory was partially restored, but a degree of fatuity still remained."

Dr. Macfarlane observes, with reference to the preceding case, that the symptoms which developed themselves subsequently to the subsidence of the primary symptoms of concussion, depended in all probability on extravasation of blood. For, as he remarks, it does not necessarily follow that when a small portion of the brain is lacerated, the hæmorrhage should be immediate. The collapse produced by the injury may prevent the flow of blood from the divided vessels at the time, and they do not bleed until reaction is established. Be this as it may, nothing is more common than to observe the symptoms of concussion to diminish, and symptoms of compression to come on, unattended with any evidence of inflammatory action. If, indeed, the extravasation is extensive, there is usually no pause, the symptoms of concussion being mixed in the first instance with those of compression, or merging indistinguishably into them.

It is not unfrequently said, and in works on surgery it may be seen, that patients may die from concussion alone. We must confess that we are very sceptical on this point, and whilst we are well aware that the thing does occasionally occur, we believe that the occurrence is extremely rare. That patients die without ever rallying from the state of collapse is most true, but in all the cases of this description that we have witnessed, there was more or less destruction of the organization of the brain, or effusion of blood, or other destruction of parts.

Dr. Macfarlane has met with three fatal cases of concussion attended with laceration of the brain, the laceration corresponding in none to the part of the skull on which the blow was inflicted. These injuries were confined to the upper and under surfaces of the cerebrum, and to the central commissure; the effusion of blood was considerable, and in one of the cases it was injected into the substance of the brain to some distance around the injured part.

Dr. Macfarlane thinks that secondary hæmorrhage may occur in the brain as elsewhere, and details a case in which he believes that it occurred. He is more confirmed in his opinion from the circumstance of a very similar case having been related by Mr. Brodie, in his excellent paper in the Medico-Chirurgical Transactions, a paper of which a full analysis was given in this Journal.

Case.—*Death apparently from Secondary Hæmorrhage.*

F. C. æt. 35, fell, Jan. 1, 1827, from a considerable height upon his head, and was carried home insensible. In three hours he had so far recovered as to be able to answer questions, but he lay for several days in a state of drowsiness, from which, however, he could be easily roused. On the 8th he was admitted into the Infirmary. His pulse was sixty-five, and feeble; his eyelids were half shut, and the pupils slightly dilated; but his respiration was natural, and free from stertor. No wound of the scalp or fracture of the skull could be discovered.

On the 9th, he had three severe attacks of convulsions, the last of which was followed by immediate and complete insensibility; his pulse sunk to fifty in the minute; his breathing became laborious; his pupils dilated and immoveable; and his urine and fæces were passed involuntarily. He died on the 11th.

On inspection, a considerable portion of the dura mater was found detached from the cranium. Two lacerations of the substance of the brain were discovered, both being situated on the upper surface of the anterior lobes. That on the left side was covered by a firm coagulum, about the size of a walnut; while, from the right, nearly three ounces of blood was extravasated, and spread over the surface of the hemisphere. This blood was fluid, and had every appearance of having been recently effused.

"The coagulum on the left side was probably formed soon after the injury, any farther hæmorrage having been prevented at the time by the free blood-letting, and other depletory measures which were employed. On the 9th day from the period when the injury was inflicted, an increased determination of blood to the head took place, which was occasioned by the attacks of convulsions, and a renewal of the hæmorrhage was the consequence. The effused blood extended over the right hemisphere, and to this occurrence the sudden coma and death of the patient were to be ascribed."

This case is very illustrative of Mr. Brodie's opinions. That able practical surgeon is inclined to believe, from the cases which he has witnessed, that convulsion is produced by slight extravasation, or other source of pressure, coma, stertor, &c. by a more extensive or powerful compression. It was evident from the symptoms of the patient until the last attack of convulsions, that there was some source of pressure or of irritation. The small coagulum on the left side was this source. The convulsions thus occasioned were the cause of the larger and fatal extravasation, which fully deserves to be considered a secondary hæmorrhage.

Dr. Macfarlane relates a case of secondary suppuration on the surface of the brain after injury of the head. There is nothing in the case to distinguish it from the many of a similar character that have been published. The rigor occurred on the 15th day, and the patient died on the 19th. She was not trephined. The injury was fracture of the cranium without depression. Pus was found on the anterior lobe of the left hemisphere of the cerebrum, and also between the cerebrum and cerebellum.

We notice the succeeding case more at length, because it involves a contested principle, that of trephining or not trephining in cases of compound fracture of the cranium, independently of the existence of symptoms of disturbance of the brain.

R. A. æt. forty, admitted February 1st, 1832, at 6 P.M. having on the preceding evening, at ten o'clock, been assaulted by two men, when he received several blows on his head with a bludgeon. He was stunned for a short time, but soon recovered so far as to be able to walk home. In the course of an hour he vomited two English pints of blood, after which he was said to have become drowsy. At the time of his admission into the Infirmary, he was perfectly collected, but irritable and peevish, and complained of intense pain in the head. The eye was suffused; the pupil variable; the pulse sixty, and of good strength. On the forehead, about an inch and a quarter above the middle of the left supra-orbital ridge, there was a lacerated wound, through which a portion of the frontal bone, about an inch in diameter, was observed to be fractured, and slightly depressed. He was immediately bled to twenty-four ounces; and on visiting him at nine, P.M., about three hours after his admission, Dr. M. found that his pulse had risen to eighty, and that he had had a slight rigor. The depressed bone was firmly fixed, but at one point the pulsatory motion of the fluid blood, which filled up the fissure, showed that the fracture extended through both tables of the scull. On enlarging the wound by a crucial incision, and dissecting back the flaps,

Dr. M. raised up the depressed bone with the elevator, and removed it in five separate pieces, along with a small coagulum. From the oozing of blood, the small size of the opening in the cranium, and the restless state of the patient, who was secured with some difficulty, it was impossible to ascertain whether the dura mater was injured or not. The edges of the wound were retained in contact by a suture, and the usual dressings, with a double-headed roller, applied. A bladder containing a refrigerative mixture was applied to the head, and a purgative exhibited. For the first nine days, the most active antiphlogistic measures were employed. Besides smart purging, nauseating doses of emetic tartar, *&c.*, one hundred and twelve ounces of blood were detracted by venesection. On the 14th, an abscess burst in the left ear, after which no unfavourable symptoms occurred. The wound healed slowly, the pulsations of the brain became gradually more obscure, and he was dismissed, cured, on the 19th of March.

"In such a case," says Dr. M. "nearly all the surgical authorities of the present day condemn the practice of applying the trephine, and removing a sound portion of the cranium, that the depression may be elevated, unless symptoms of compressed brain exist. They approve of the removal of such loose and detached splinters as can be readily laid hold of by the forceps, or raised by the elevator; but until symptoms of compression appear, either from the effusion of blood, or the occurrence of suppuration, they are, with one or two exceptions, unanimous in condemning the trephine. On the contrary, Pott recommends that the cranium be perforated, and the depressed bone elevated, not with the view of removing existing symptoms, but as a preventive of ill consequences. Sir A. Cooper, and Mr. Brodie, appear less hostilely opposed to this practice, than the rest of their contemporaries. The former gentleman says, 'I generally use an elevator to raise the depressed bone, but *rarely* apply the trephine;' whilst Mr. Brodie is of opinion, that, when the depression is slight, and the symptoms trifling, the trephine should be applied only when the injured bone is exposed, in consequence of a wound in the scalp. Considerable diversity of opinion also exists as to the cause of the suppuration, which so frequently occurs in the neighbourhood of a fractured and partially-depressed bone. The majority assert that this suppuration does not arise from the depressed bone, but from the violence inflicted on the brain by the original injury; and that, of course, the application of the trephine cannot prevent the formation of the pus, but, on the contrary, by the additional injury which it produces, the inflammatory mischief will be greatly aggravated: they have recourse, therefore, to active antiphlogistic treatment, which they maintain is generally successful in warding off the impending danger; and assert that the trephine ought not to be applied, till, from the accumulation of matter, unequivocal symptoms of compressed brain manifest themselves. To wait for this occurrence, is to delay till the chances of success from surgical interference are almost hopeless. We have no means of ascertaining the exact situation of the purulent collection, or whether it lies above or below the dura mater; and we shall often fail in evacuating it, even after the trephine has been employed. I have seen it spread over an entire hemisphere; and when it is so situated, and so extensive, the disorganization of the brain, resulting from its accumulation and diffusion, will frequently lead to a fatal result, even although it should be evacuated by an operation: but this cannot be accomplished when it is collected under the pia mater, a situation in which Sir Astley Cooper states that it will be generally met with. It does therefore appear, that if we can, by a cautious use of the trephine, remove the fractured and depressed bone, soon after the injury has been inflicted, we shall succeed in relieving the brain from a source of dangerous irritation, and be enabled more effectually to combat the inflammation, and ward off the suppuration, which so frequently ensue; and that, upon the whole, the result will be more fortunate than were the operation deferred till suppuration had commenced, and symptoms of compression were present. I have seen, during the last six years, five cases of compound fracture of the skull, with partial depression, treated in the infirmary, according to the Abernethian plan, but unsuccessfully. There existed at first no symptoms of compressed brain; it was therefore considered imprudent to have recourse to the trephine, the usual antiphlogistic means being alone trusted to. In from six to fourteen days, a rigor took place, followed by headach, drowsiness, and stupor, until at length stertorous breathing, slow pulse, dilated pupil, coma and death, supervened. In two of these cases, the trephine was applied, and a small quantity of pus, collected exterior to the dura mater, was evacuated, but without the slightest relief to the compressed brain. On dissection, the greater part of this fluid was discovered between the dura and and pia mater, and between the pia mater and brain; and in these two, as well as in the other fatal cases from suppuration, the inner table of the skull was more extensively fractured than the outer one, and an irregular portion of it thrust down on the brain. Finding that these unfavourable results are of such frequent occurrence, when the operation is deferred till suppuration is established, I have been long of opinion with Mr. Brodie, that, in compound fracture of the cranium, the early removal of the depressed portion of bone by the trephine, when it cannot be accomplished by the elevator, is the safest, and likely to become the most efficient practice. I have only had an opportunity of trying it in one case, and in this the result was successful."

Thus it will be seen that Dr. Macfarlane agrees with Sir Astley Cooper and Mr. Brodie in thinking immediate elevation advisable in cases of compound fracture of the cranium, attended with depression.

---

### XLIII.

### Mr. Middlemore on the Purulent Ophthalmia of Infants.

There is a sensible paper on the Ophthalmia Neonatorum in our valued contemporary, the Medical Gazette. We shall notice some of Mr. Middlemore's remarks on the causes and treatment of the disease. With respect to the first, Mr. Middlemore appears satisfied that it is always to be found in morbid vaginal discharges of the mother, there being at the same time some defect in the children themselves, or in the management of them, predisposing them to the disease, or aggravating it when present. Those morbid vaginal discharges may be gonorrhœal, gleety, or leucorrhœal; in short, they may be various, without corresponding, or at least recognizable, varieties in the character of the induced ophthalmia. This seems to be the pith of Mr. Middlemore's theory of causation.

"Infantile purulent ophthalmia more frequently attacks the progeny of the poor than the offspring of those in better circumstances; it is more common in premature children and twins, and is most obnoxious to those who are weak and delicate in constitution; it is also observed to be very prevalent in Foundling institutions—those, I mean, that receive infants deserted by their parents immediately after birth; whence I infer, that delicacy of constitution, want of cleanliness, defective nursing, and a vitiated and unwholesome atmosphere, are powerfully predisposing causes."

Now leucorrhœa is very common in women of the middling and upper classes, but in their children ophthalmia is very uncommon. This proves, without further argument, that other circumstances are necessary to render the leucorrhœal discharge operative in producing the ophthalmia. Cleanliness and clothing, and pure air and vigour, are probably the most essential protectors against contamination; but whether differences in the maternal discharge are operative in rendering the infant more or less liable, is a question that we cannot pretend to solve. It certainly does appear, that a person with gleet may or may not communicate gonorrhœa to the female with whom he is connected, and it is also likely that what is termed leucorrhœa, that is a mucous discharge from the vagina, not consequent on venereal infection, will occasionally, but not commonly, infect the male. We are not well acquainted with the laws that regulate the infecting power of vaginal discharges. But let us pass to Mr. Middlemore's method of treatment; It is rather judicious than novel.

In slight cases, an astringent lotion and a dose or two of magnesia will effect a cure. The majority of cases are not so slight, and much depends on obtaining the management of them at an early period. In such a case, at such a period, we may use a solution of alum and a slight aperient. The eye should be washed every half-hour, or, if necessary, oftener, with a well-made ivory or pewter syringe, the point not too sharp; the extremity is to be placed between the lids, at the outer angle of the eye, and should pass it, for a short distance, almost parallel with the surface of that organ, and not so obliquely as to hurt the eye-ball, or oppose the conjunctiva as an obstruction to the issue of the fluid. The eye is thus to be syringed with a little warm milk and water, after which a weak solution of the sulphate of zinc or alum (gr. iij. ad ℥j.) may be thrown in, and repeated, with the same preliminary washing, every second hour. Mr. M. recommends the nurse to apply a little fresh butter, or sweet oil, or mild ointment, along the tarsal margins, whenever the child is about to sleep, a precaution which renders unnecessary the tedious bathing of the lids. This should be combined with a dose of magnesia, or, if the child be jaundiced, as sometimes happens, of hydrargyrus cum cretâ.

The inflammation may be severe, with chemosis, &c. Mr. M. then recommends the application of a leech to the root of the nose, or upper lid of each eye, according to circumstances. Of course great care must be taken to prevent the bleeding from proving too considerable. If the lids are much swollen, with obstructed circulation, and if any vessel be particularly large, it may be opened with a lancet, and the bleeding encouraged by warm water.

If the case has proceeded farther, the cornea having ulcerated or sloughed, the chemosis diminished, and the conjunctiva become comparatively pale, "it would be right to use a solution of the nitrate of silver (two or more grains to the ounce) two or three times a day, the eyes being frequently bathed with zinc lotion. If suppuration of the eye-ball has taken place, the surgeon should not be too tardy in puncturing the cornea, and discharging the contents of the eye-ball.

Mr. Middlemore protests against "the harsh applications" of Dr. Vetch, particularly the undiluted liquor plumbi acetatis. He also thinks that, at the present time, there is an "extraordinary partiality for the early employment of stimulants." He would not wish them used till the proper time, that is, till the acute stage has passed by. Mr. Middlemore cannot coincide with Mr. Guthrie, in using the unguentum argenti nitratis in the earliest stage of the purulent ophthalmia of infants.

As Mr. Middlemore's strictures are levelled against stimulating applications generally, when employed in the early stages of ophthalmia, we may be permitted to make this remark—that at Mr. Guthrie's Ophthalmic Hospital, stimulants are not found to be productive of those mischiefs which their opponents attribute to their employment. For the results of Mr. Guthrie's treatment, we can safely refer to a report from the Ophthalmic Hospital, published in a late Number of this Journal. The cases there detailed are satisfactory evidence of the advantages, we put it broadly, the great advantages, of stimulating *early* in certain conjunctival inflammations.

---

### XLIV.

### ON THE EMPLOYMENT OF ISSUES, AND OF OTHER DRAINS, IN THE PREVENTION AND TREATMENT OF DISEASES.

THE author, M. Chauffard, of Avignon, strongly insists upon the good effects of these means in a variety of morbid affections. Several cases are adduced of their prophylactic and curative efficacy, in strumous and cachectic children; thus, obstinate coughs, headachs, convulsions, hæmoptysis, dyspnœa, have yielded to their use, when every other remedy had failed. We need not say that strict attention must be paid to regimen and dietetics at the same time, although we deem these latter means to be often unavailing by themselves. Fabricius Hildanus and Ambrose Parè state that they had more confidence in issues and setons on the nape of the neck, for the cure of obstinate epilepsy, than in any other measures. "Quo solo, innumeros cerebi mortiferis morbis correptos, ad sanitatem, ulceribus diu fluidis permanentibus, perduxi." Cases not unfrequently occur, where all the symptoms of confirmed, or at least of threatened, phthisis disappear, upon the supervention of a large anal abscess: for example—a soldier, in the second stage of the disease, had cough and purulent expectoration, hectic fever, night sweats, and was greatly wasted in flesh. A large abscess formed in the perineum, broke, and discharged copiously; in time it healed, and two issues were established on the inside of the thighs. The patient quite recovered. [How vexing it is that medical men will not use their ears, and affix to the reports of their cases the auscultatory signs; without these, we are not warranted in drawing any safe conclusions.—Ed.]

"My own experience (says Baumes) induces me to think, that the early stage of consumption is best counteracted by issues, sufficiently multiplied and renewed. Euryphon covered, in some degree, the bodies of his patients with them, and did not expect much benefit unless the discharges were copious, and from different parts. Louis, a high authority in this disease, reports favourably of the same means, and states, that if we have the courage to employ in time a sufficient number of these derivative channels for the acrimonious humours, very marked advantages may be expected from the practice. Dr. Mudge considered that he cured himself of pthisis by establishing a large issue on his back; it held fifty peas. The practice has the authority of Celsus:—"Exulcerandum est ferro condenti, uno loco sub mento, altero in gutture, duobus ad mammam utramque, item sub imis ossibus scapularum."

The chronic inflammation of the larynx, accompanied with an ulcerated state of the mucous lining, is another species of pthisis, which is as generally fatal, as the true pulmonary consumption, and which, in many cases, is equally benefited by the establishment of permanent purulent drains. The author alludes to one case, which was cured by repeated moxas on the sides of the neck, by absolute quietude and silence, and by a sojourn in the neighbourhood of Naples.

We shall give the particulars of another case, not only because the subject is one of importance, but also as it affords an occasion to introduce to our readers a specimen of a prescription from a French physician.

A gentleman aged 38, was affected with a chronic irritation of the larynx; the voice was hoarse and feeble; the expectoration was copious, purulent, and streaked with blood; it was brought on by paroxysms of a cough, which was not deeply seated; the palate and mouth were inflamed, covered with livid vesicles and œdematous; hectic had wasted the patient's strength. He was ordered warm clothing, light farinaceous and milk diet, and absolute silence for a year, or more; small doses of the syrup of morphine were taken twice a day; he was occasionally bled to a small amount, or leeched; a laudanum poultice was put round the neck; and an issue, to hold four peas, inserted on the nape. The lunar caustic was applied to the rectum and inside of the mouth. By a rigid observance of the above treatment for four months, the patient's health was much improved; the issue was kept freely open for more than five months, and the discharge was so copious, as to require fresh dressing twice daily. M. Recamier was consulted at this time, and the following is an abridged copy of his "response."

"The facts which are to be attended to, in considering this patient's case, are the supervention of hæmoptysis, occasioned by long-continued exercise of the voice; the existence of a chronic irritation of the larynx, with hoarseness; and lastly, an unhealthy place of abode, and an occupation unfavourable to recovery. I therefore prescribe a mild diet of butcher and poultry meat, farinaceous vegetables, ripe fruits;—water, as the only beverage; and asses' milk every morning and evening. An alum gargle is to be used for the throat, a small blister is to be applied on the front of the neck; and the patient must keep absolute silence for at least twelve months after the cure; he should avoid all drafts of air, and when he takes exercise, it must be gentle and easy;—much heat also is hurtful; and hence large fires, stoves, &c., are not to be approached."—*Trans. Med. Aug.* 1832.

### XLV.

### Aphorism.

"Immo vero ex physiologia omnes antedicti sapientiæ professores ostendunt, neminem posse morbos commode curare qui corporis universi naturam non perspexerit."—*Galenus*.

### XLVI.

### Analogy between the Asiatic Cholera and the Sweating Sickness, or "Suette Epidémique."

Dr. Dubun-Peyre-Longue, physician in the "arrondissement" of Pontoise, considers that these two diseases are very closely allied to each other. He resided, in 1821, in a district which suffered much from the "suette;" and had thereby an opportunity of eminently studying its character.

During last year, cholera existed contemporaneously with it; and many cases of the "suette" assumed in their progress all the symptoms of the former pestilence. The skin, in the one disease, and the mucous surface of the bowels iu the other, are the organs in which the essential symptoms originate, and are chiefly manifested, so that cholera may fairly be termed an internal "suette;" and this again, a cutaneous cholera. Dr. Gendrin, in his excellent Monograph on Cholera, says,—"If I had to find a place for this disease in a nosographical code, I should arrange it along side of serous diarrhœa; and not far from the "suette" of the 15th century; that frightful cutaneous phlegmorrhagia, in which the skin was the emunctuary by which the blood lost so quickly its watery portion. The most appropriate name for cholera would be "phlegmorrhenteria."—*Ibid.*

### XLVII.

### Case of large Hydatid Cysts in the Abdomen and Thorax.

A man, aged 28, fell from a ladder on his right side, in March, 1831. In April, 1832, he had another fall; and after this time he felt a severe pain in the right hypochondrium and right shoulder; jaundice came on; the pain got worse and worse; the abdomen, especially over the hepatic region, increased in bulk; and dyspnœa was added to his other sufferings. The appetite and digestion, however, were not impaired. On examination, Dr. Gendrin found a large elastic swelling occupying the right hypochondrium, and extending upwards, so as to elevate the ribs, and downwards and laterally to the umbilicus and left hypochondrium. Fluctuation was distinctly perceived. MM. Gendrin, Recamier, and Dupuytren, gave it as their opinion, that it was probably a large hydatid cyst, developed, either in the substance of the liver, or on its convex surface; and repeated applications of the potassa fusa were advised, for the purpose of opening it. But this was not effected in the course of two months; and as the swelling had much increased during that time, M. Gendrin, after ascertaining as well as he could, that it was adhering anteriorly to the abdominal parietes, plunged a trocar into it, and let out four pints of a colourless, inodorous, and thin fluid; a gum elastic tube was left in the orifice: tepid water was for several days injected, and allowed again to escape: it generally had obtained a yellowish colour, and was muddy and highly fetid. The discharge increased, and became mixed with yellow, transparent, friable shreds, which appeared to be formed of semi-concreted mucus or albumen. The patient died 23 days after the operation.

*Dissection.* The fistulous opening in the side led in to a large cavity, which was partly situated in the excavated substance of the upper portion of the liver, and occupied the right hypochondrium, the epigastrium, and also a share of the left hypochondrium: it was thus placed between the diaphragm, liver, and abdominal walls; its inner surface was lined with a yellowish, wrinkled, and powdered membrane. On further examination, this immense cyst was found to communicate with another cyst in the thorax, by an aperture through the substance of the diaphragm; this aperture was about an inch across, and its edges were lined with a false membrane similar to what lined the cysts; it was situate, a little above and to the outside of the vena cava; the thoracic cyst occupied the whole of the right cavity of the chest; the lung of this side was squeezed backwards on the spine, and its substance very much compressed.—This cyst contained a considerable quantity of turbid and offensive cerum, in which floated a pellicle of a yellowish colour, transparent, friable; and so large that it covered a foot and a half square of surface: it did not adhere at any points to the cyst; and it had no trace of any vessels in its texture. The substance of the liver was nearly natural, being only somewhat hardened in the neighbourhood of the cyst.—*Ibid. Sept.*

---

### XLVIII.

Chemical Pathology of the Blood in Cholera, by M. de Canu, Professor to the School of Pharmacy at Paris.

The most remarkable and obvious change, is the singular increase of the proportion of the solid matters, to the watery portion: the following experiments clearly demonstrate this fact.

| | | | |
|---|---|---|---|
| In the first: | | | |
| Water ...... | 66 | | |
| Solid matter.. | 34 | | |
| | 100 | | |
| In the second: | | Mean proportions. | |
| Water .... | 74.9 | Water ...... | 63 |
| Solid matters | 25.1 | Solid matters | 37 |
| | 100. | | 100 |
| In the third: | | | |
| Water ...... | 48 | | |
| Solid matters . | 52 | | |
| | 100 | | |

In healthy blood, the proportion of the watery part is about 0·78; and if we take this as a standard, the blood of cholera patients contains sometimes twice the normal quantity of solid materials; hence we can readily explain the treacle-like appearance of this fluid, as it flows from a vein in the arm. I have not detected any considerable changes in the fibrine, albumen, and colouring matter; this last, indeed, was of darker hue in the arteries, and seemed to have experienced a sort of modification, analogous probably to that which takes place in the conversion of venous into florid blood. In attempting to ascertain the exact proportions of the different constituents of cholera blood, much uncertainty arises from the difficulty of separating the serous portion; but my observations do not agree with those of Hermann, of Moscow, who states, that cholera blood offers sensible traces of acidity. The relative diminution of the alkaline carbonates is always appreciable, sometimes indeed to such a degree, that they can with difficulty be detected; and since these salts, as well as albumen, and an extractive matter, which has been compared to osmazome, are found in the stools, we may fairly attribute the increased thickness of the blood to the draining of the serous part by the intestines.

The white and apparently fibrinous matter observed in the rice-water stools, seems to me, to be rather of the character of mucus, than of fibrine; and I am more inclined to this opinion, as the fibrinous portion of the blood was not found defective.—*Ibid.*

---

### XLIX.

**CURIOUS CASE OF MORBUS CÆRULEUS, CAUSED BY THE AORTA COMMUNICATING WITH BOTH VENTRICLES, AND YET NOT OCCURRING TILL SEVEN DAYS AFTER BIRTH.**

FOR the first six days of his life, the boy was apparently in all respects healthy; and the colour of the skin was of the usual pale reddish hue. On the seventh day, after a violent fit of coughing, the skin suddenly assumed a purple tinge. Notwithstanding this, the general health was moderately good, and the child throve well; he began to walk at the end of twelve months, and passed through dentition, measles, and vaccination, favorably; but his constitution was feeble, flabby, and scrofulous, and the blue colour of the skin was especially deep on the ears, cheeks, and lips, and extended inwards down the throat as far as could be seen; the limbs became atrophied; and the nails of some of the fingers were so curved round, and bent upon the bulbous ends, as to prevent them being used in grasping small objects. The respiration was triflingly hurried and uneasy, and digestion went on well: the pulse, although feeble, was of ordinary frequency, and synchronous with the pulsations of the heart. The child was repeatedly exhausted by hæmorrhages from the mouth; and died in his twelfth year, in consequence of epistaxis.

*Dissection.* The lungs were not above one half their usual size; their texture was hepatised in most parts, and their colour was of a yellow hue, except at those places where their texture was healthy. The blood in the two venæ cavæ was unusually black; the pulmonary veins were almost empty. The heart was somewhat enlarged, and its substance softened; and softening was apparent in all the other muscles: the large vessels arose regularly from the heart: but the pulmonary artery was small and contracted; the canalis arteriosus, which communicated with the right branch and not with the trunk of the pulmonary artery, was obliterated at its junction with the aorta, but open in the rest of its extent; the right auricle, and foramen ovale, were perfectly normal; the right ventricle communicated by one orifice with the aorta, and by another, which was smaller and contracted by a membranous projection, with the pulmonary artery; the left auricle was healthy, and also the corresponding ventricle, except the aortal orifice, which was smaller than the one in the right ventricle. The septum was of its ordinary thickness, except at the upper part, where it was perforated by an opening whose lower margin was formed by the muscular substance of the septum, and the upper one, by the walls of the aorta; by this aperture both ventricles communicated with the aorta, the edge of the septum forming a projection into its canal; the semilunar valves were healthy; but one of those in the mouth of the pulmonary artery was ossified, and much obstructed the passage, which was thus reduced to a diameter of three lines.—*Ibid.*

---

### L.

**ON PULMONARY EMPHYSEMA, IN CASES OF SUDDEN DEATH, AND IN ASPHYXIA FROM HANGING.**

DR. PRUS, after adverting to the causes of death in such cases, from closure of the larynx, with or without rupture of its cartilages, or of the trachea, from lesion of the spinal cord by luxation or fracture of the cervical vertebræ, from congestion of, or hæmorrhage in the, substance of the brain, &c., endeavours to prove that death may also be partly occasioned by an emphysematous state of the lungs.

CASE 1. L. A. aged 69, entered the Bicetre Infirmary on the 10th January; he laboured under a constant oppression and sense of suffocation when he walked. Auscultation and percussion abundantly shewed that the lungs were perfectly normal; and the only disease which could be ascertained was a slight hypertrophy of the left ventricle. One morning, he was found hanging from the roof of the bed by means of a cord, which serves to enable the patients to raise themselves up.

*Dissection.* No lesion of the larynx and trachea. The upper lobe of the left lung, and the upper and middle lobes of the right one, were affected with vesicular emphysema; and, at one part, the air had escaped from some ruptured cells under the pulmonary pleura, and had thus formed three large bladders of air, each nearly an inch across. The appearances in the other viscera were those usually found, as congestion of the cerebral vessels, &c.

*Remarks.* One of the most frequent phenomena in the bodies of those who die from hanging is the fluidity of the blood, observed long ago by Valsalva. The blood was perfectly fluid in the preceding case. By keeping this in mind, we can more easily account for the purple congestions and infiltrations in the skin, mucous linings of the bowels, bladder, and urethra, which Morgagni alludes to. It remains for future enquirers to ascertain whether the emphysema, which we have pointed out, be a constant, or merely an occasional phenomenon. Littre, in the Mem. de l'Acad. Roy. des Sciences, 1704, says that he found the lungs of a woman, who had been strangled, forcibly distended by the air which they contained, and the pleura entirely separated from the subjacent pulmonary tissue. Bonetus, in his Sepulchretum, says that, in one case, there was "fistulam spumâ pulmonum impletam." In some of the cases reported by Orfila, we observe that the lungs had been found emphysematous.

In all researches on asphyxia, it is of the first importance to remember that death may often be induced, where the mechanical constriction or obstruction of the air-passages does not totally prevent the ingress of the air. Foderé has seen a case, in which complete and fatal asphyxia was caused by a small portion of the shell of a nut, which, on dissection, was found lying free, and not at all impacted, in the bifurcation of the trachea into the two bronchi. Some instances of very sudden natural death are probably caused by an emphysema, either vesicular or interlobular, of the lungs. Dr. Prus alludes to a case of an old gentleman who was found dead in his bed; and the only morbid appearance on dissection was an immense sub-pleural emphysema.

A man in the Bicêtre Infirmary became quite suddenly alarmingly ill; the face was purple, no pulse could be felt, and the extremities were cold and motionless; the physician predicted either hæmorrhage within the skull, or pulmonary apoplexy, or perhaps rupture of the heart or of the large bloodvessels. On opening a vein no blood flowed out, and the patient very speedily died.

*Dissection.* The only lesion which could satisfactorily account for the above seizure, was an immense subpleural emphysema of the whole inferior lobe of the left lung. The observations of MM. Leroy d'Etiolles, and Piedagnel, confirm the idea which we have set forth, that many cases of sudden death are attributable to pulmonary emphysema; and it is, therefore, very probable, that the speedy occurrence of this lesion may be one of the causes of death from strangulation.—*Ibid, October.*

---

### LI.

### On the Accidents caused by the Presence of Air in the Circulatory Organs. By Dr. Forget.

Perhaps the very best memoir on this interesting subject is, to this day, the fifth letter of Morgagni's admirable work. He adduces two cases of very sudden death, apparently from apoplexy; but nothing unnatural was found upon dissection, except the presence of air in some of the cerebral vessels. He reminds his readers that Hippocrates admitted a "gaseous apoplexy." "Si quidem plurimi flatus per universum corpus discurrant, totus homo syderatur, si per partem, pars illa percutitur." Valsalva mentions that he once found the heart and the veins distended with a gas; Grotz witnessed the same, in a woman who died of suffocation, and Ruysh reports a similar phenomenon, which occurred in a case of very sudden death. In the above examples, the air seems to have been generated in the sanguiferous system, and, according to some authors, the blood of tortoises, and of a few other animals, always contains air. Wepfer is the first who experimented on the effects of introducing air into the veins of animals; it is said that he killed large animals, as oxen, dogs, &c. by blowing into the crural veins. Brunner, Redi, Rudolphi and others, have followed in the same path; and all agree in attributing the quick death to the distention of the right cavities of the heart with air. Portal, in 1771, repeated many of the experiments, and was led to believe that the true cause of death, in such cases, was cerebral apoplexy; Bichat, in his General Anatomy, ascribed it to the unusual impression made on the brain by the air, and not merely to its pressure, as Morgagni thought. "When (says he) a bubble of air enters a vein, the animal utters piteous cries, struggles, and is convulsed before death; are we to attribute this to the contact of the air on the common membrane (of the veins)? I think not; for usually a moment or two intervene between the injection and the cries of the animal; we should rather suppose that the pain is felt at the moment when the 'air strikes the brain,' after having traversed the lungs." Nysten, in his Recherches Physiologiques, 1811, inclines to the opinion of the old experimenters, and shews that the distention of the heart by air can satisfactorily account for the death. Medical men were at that time not aware that the very accident, which had been artificially and designedly produced in their experiments, may happen most unexpectedly in the human subject. No doubt many instantaneous deaths must have occurred to surgeons, during operations, long before the real cause was even suspected; for the pain, alarm, or syncope of the patient were then deemed a ready and sufficient explanation of such catastrophes; but it was not till 1821 that Magendie pointed out the fallacy of such opinions. This able surgeon was in the act of extirpating a large tumour from a man's neck, and when the operation was nearly over, the patient on a sudden cried out, "My blood falls in my body —I die;" at the same moment he became stiff and pulseless, and expired 45 minutes after the commencement of the operation. On dissection, it was found that the internal jugular vein had been divided near to its junction with the subclavian; bubbles of air were found in the cerebral vessels. In the following year, a similar accident occurred in the Hôtel Dieu. Dupuytren was engaged in dissecting out a tumour from a girl's neck; suddenly a whistling noise, like that of the rushing in of air along a pipe into a vacuum, was heard; the operator was astonished, and exclaimed—"if we were not too far from the air-passages, we should believe that we had opened the trachea." Scarce had he finished these words, and had divided the last attachment of the swelling, when the patient cried out, "I am dead;" and forthwith she was seized with a universal trembling, and expired.

*Dissection.* The right auricle was found blown out with air, which rushed out, unmixed with blood, when the walls of the auricle were divided. In the other parts of the circulating system, the blood was so blended with air, that on pricking many of the vessels, frothy bubbles oozed freely out. The lungs and other viscera were perfectly sound.

M. Leroy d'Etiolles, in the Archives Générales for 1823, inserted a memoir, the purport of which was, to prove that the death, in such accidents, is attributable to asphyxia from emphysema of the lungs. He says—"the introduced air is driven, by the contractions of the right ventricle, into the capillaries of the pulmonary arteries, bursts them by its expansion, and thus causes a general emphysema of the substance of the lung; and this is the real cause of the stoppage of the circulation." Veterinary surgeons have met with the same accident, in bleeding horses from the jugular vein. In the "Lancette," Nov. 1830, several cases, which occurred in the human subject, are adduced by M. Clemot; in one, during the excision of a tumour in the axilla; in another, when he was tying the subclavian artery; and, in a third, when he was dissecting a tumour from the neck. In the two first cases the patients recovered; a noise like that of aspiration, or of a bellows, was heard; the finger was placed on the spot, and the noise ceased: when it was withdrawn, the blowing was again heard; the vein, which was not very large, was then tied, and no bad effects resulted. It appears, therefore, that Bichat was not correct in supposing, that the admission of a single bubble of air suffices to cause death. Dr. Barry, in 1825, specially directed the attention of the profession to the influence which the pressure of the atmosphere on the external surface, and the suction of the right side of the heart, have on the propulsion of the blood in the veins. He states that the black blood is moved forward only during inspiration; and it is very generally known that hæmorrhage from, or engorgement of veins, may often be abated by a few deep-drawn breaths. Professor Berard, in the Archives Générales, 1830, points out the anatomical condition of some of the large veins, by which they are more exposed to the suction of air into their canals than others. The most recent example of this accident occurred in the La Charité Hospital last Sept. M. Roux had nearly finished an operation of extirpating a tumour from the neck, when suddenly a whistling or sighing noise was heard, and immediately afterwards a gurgling or bubbling within the chest; the patient uttered some plaintive cries, and was convulsed; syncope supervened, and every one thought that she was dying; she, however, recovered gradually from this state, and the operation was finished by putting a ligature round the narrow neck, by which the tumour was attached; it separated upon the 10th day, and on the 13th she died of a stomach ailment, according to M. Roux.

*General Remarks.* The veins of the neck and adjoining parts are those chiefly exposed to the introduction of air during an operation. As we have hinted above, it is probably necessary that the vein be attached closely to the surrounding tissues, whether the attachment be normal or not, to keep the orifice of the divided vessel open and gaping. Whenever such a vein is cut across, it will be prudent to tie it as quickly as possible, especially if the least whistling murmur be heard. Magendie has the hardihood to propose a remedy, which recals to our minds what Bichat advised in desperate syncope, and which has been employed with success in the lower animals. The remedy consists in introducing a silver canula into the jugular vein, and, after directing it towards the heart a syringe is to be fitted on, and the air and blood to be pumped up. No trial has ever been made.—*Ibid.*

---

### LII.

### Assassination of M. Delpech.

On the 29th of October, a ruffian, of the name of Demptos, ran towards Delpech's cabriolet with a double-barreled gun in his hand. He fired, and the ball entered the left side of the chest: he fired a second time, and killed the servant on the spot. Delpech died in a few minutes. The assassin was a native of Bourdeaux, and aged 36; he had some time before applied to M. Delpech, on account of a varicocele, which, by proper treatment, was speedily made better. He returned from Montpellier to Bourdeaux, and there fell in love with a girl, whose parents, however, refused their consent. On being urged, however, to explain their reasons, they admitted that M. Delpech had been consulted by them, and that his opinion was not a favourable one. Demptos forthwith repaired to Montpellier, resolved either to force a retractation from Delpech of what he had said, or to assassinate him. On the evening before the murder, Delpech was in the theatre along with his son; Demptos went up to him, and demanded a letter, which might confute the opinion given to the parents of the girl; the Professor refused to comply; and the villain left him, with threats of revenge.—*Ibid.*

---

### LIII.

### ON THE GOITRE, AS IT IS OBSERVED IN THE INHABITANTS OF THE PLAINS OF THE ANDES. By M. HUMBOLDT.

THE districts of Europe in which goitre and cretinism are chiefly found, are the narrow, confined, damp, and very warm valleys of Switzerland and Savoy. In the low-lying and warm countries of New Grenada, and the plains of the Rio Magdalena, situated between the second and ninth parallels of southern latitude, many of the people are affected with the most hideous goitres. All along the tract of this river, from its source to its confluence with the Cauca, a long extent of country, whose altitude above the level of the sea varies from 1800 to 180 feet, and which is called by the natives "Terra caliente," this singular malady is found to prevail. Now in this wide extent there is every sort of locality; at one part there are extensive forests and marshes, where the atmosphere is always stagnant and damp; at another part there is an immense plain, where the heat of the sun is so excessive as to scorch up all the vegetation, and the winds are often high and tempestuous, and yet here more goitres are seen than in the other region, where the air is moist, and never fanned by a breeze. If we follow the river Magdalena, above Tacoloa, between the 9th and the 11th degrees of latitude, all the goitres have disappeared; and what seems still more remarkable, this disease is never seen in the valley of the Rio Cauca, which flows parallel to the Rio Magdalena, between the same degrees of latitude. M. Calder, in his treatise on the Influence of climate, printed at Santa Fe de Bogota, says, that when we leave the Rio Magdalena, and follow the course of one of the streams which fall into it, we no longer observe any traces of goitre, nor yet at Caserez, nor in Antioquia, whose climate is warm and moist, and, in almost every respect, resembles that of the plains along the banks of the Magdalena, where the goitre is so very prevalent. Illustrations of the same incongruities are to be observed in Switzerland. We cannot suppose that the disease is attributable to any peculiar properties or impregnations of the water which is drunk. At Maraquita, where it is very common, the climate is temperate and delightful, the winds are refreshing, and frequently stormy, and the water is pure and wholesome; in short, no localities can differ more from one another than those of Maraquita and of the valleys of the Magdalena, or of Switzerland. If we continue our travels higher up the mountainous country of the Andes, we lose sight of the goitre, and might, therefore, be inclined to suppose, that as the climate becomes cooler, so does the disease become less and less frequent; but we are astonished to meet it again on the immense plateau of Bogota, situated upwards of 8000 feet above the sea. The mean temperature is about 59°; no country can be more widely different from the Valais, or the valleys of Savoy, where cretinism is endemic. What is very singular is, that the goitre was not known at Bogota thirty years ago, and that it is now on the increase, although no appreciable change in the climate, food, or drink has taken place. Many of those first affected with the complaint had never visited any place where it prevails. M. Restreppo, minister of state of Columbia, in his last report to the Congress in 1823, calls the attention of his government to the subject of the goitre (enfermedel de los Cotos) in the following words:—" Every day it is extending its distressing sway over New Grenada, and now exists not only in the warm and temperate valleys, but in the icy ridges of the cordilleras." Let us briefly recapitulate the preceding observations. The disease of goitre prevails, not only in the low countries along the course of the river Magdalena (from Honda, to the confluence with the Cauca), and in the higher lands along the same river (between Nieva and Honda,) but also in the elevated table land of Bogota, 6,000 feet above the bed of the river. The first of these regions is occupied with thirty woods; the second and the third are quite exposed, and have little vegetation; the first and the third are extraordinarily humid; the second is very dry; the winds are high and tempestuous in the second and third, while the atmosphere of the first is stagnant and impure. To these striking differences others may be added; thus the thermometer in the first and second regions ranges from 74° to 78°, and in the third from 39° to 60°. The water which is drank at Maraquita, Honda, and Bogota, is not snow water, but flows from rocks of granite and lime-stone. The most horrible goitres are those seen at Maraquita, where the spring water is purer than at Honda and Bogota, and where the climate is warmer than along the banks of the Rio Magdalena. The Indian or copper-coloured aborigines, and the negroes, are almost quite exempt from this malady. The African traveller Caillaud also informs us, that he did not observe any goitres in the black inhabitants. Humboldt did not see one in the countries on the banks of the Arinois, Rio Negro, &c. where not a breath of wind is felt, to the southward of the cataracts of Atures, and where the heat and moisture of the climate are almost unbearable; while on the lofty plateau of Quito, and in the villages of Alvasi and Chichinche, at more than 9,000 feet elevation, in a climate whose temperature ranges from 50° to 60°, cretinism exists in the descendants of the white people.

The observations of M. A. de Saint Hilaire, in his travels through Brazil, confirm many of the preceding statements; the reader may therefore advantageously consult his work, and also the remarks of Mr. Coldcleugh, in Daniel's Meteorological Essays.—*Journ. Complem., Octr.*

---

### LIV.

### On the Miliary or Epidemic Sweating Fever, which prevailed at Auxi-le-Chateau (between Picardy and Artois) in the Months of June and July last.

This disease has been more frequently observed in Picardy and the adjoining dictricts, than in the other parts of France: hence it has been called "la suette des Picards." In the year 1821 it raged with great severity in the departments of the Oise, and of the Seine-and-Oise. M. Rayer has given an excellent description of it. Last year it broke out, three weeks after the cholera had made its appearance, and fortunately it did so, for the progress and fatality of this latter scourge was much arrested; many of the cases indeed of the suette, were accompanied with symptoms of cholera, or at least of cholerine; but whenever the sweating broke out profusely, the disease generally terminated favourably. Dr. Defrance treated between 40 and 50 cases and did not lose one. Most physicians are now agreed that this epidemic is not necessarily or essentially contagious; its cause appears to reside in some unknown state of the atmosphere; and no valid proofs can be adduced to prove its communicability from one to another, at least in the great majority of cases. The premonitory symptoms are headache, anorexia, general languor, nausea and feverishness; in the course of a short time, varying from a few hours to one or two days, according as the patient exposes himself or keeps his bed, the sweatings come on, and soon after these, the miliary eruption appears; this eruption is generally preceded by a most annoying itching over all the surface. The duration of the sweatings varies in different cases; but usually the patient begins to be convalescent on the third day, and then complains only of extreme weakness, which requires a week's quiet nursing and management. The sweatings have frequently a most offensive odour, like that of rotten straw, and oblige the patient to change his linen six, eight, or ten times in the course of one night. It was uniformly observed, that while the sweats lasted the pulse was full, strong and frequent, even in weak and debilitated constitutions. The importance of attending to this, is obvious in a therapeutic point of view. The urine is almost always scanty, red, and deep-coloured, till the sweating ceases or abates.

The preceding account of the disease applies very faithfully to the mild sort of the suette; but cases occur in which there are numerous and formidable complications; not unfrequently the symptoms of gastro-enteritis are present; or there are most distressing palpitations in the epigastrium; these are synchronous with the beats of the heart, and appear to be quite of a nervous character; in other cases, intense cephalalgia, stupor, or delirium; and in a third set, dyspnœa, cough, and other symptoms of bronchitis add much to the danger of the case. The treatment must necessarily be modified according to the prevailing, or casual type of the fever. An emetic of ipecacuan is in general a safe and efficacious remedy; but if any local congestion or inflammation be present, bleeding, either general, or, what is better, local, must be adopted. The convalescence from this fever is tedious and troublesome; the slightest error in diet, or any exposure to vicissitudes of temperature, will often cause a relapse. —*Ibid.*

---

### LV.

### Memoir on the Functions of the Encephalon, including a brief Notice of most of the recent Doctrines.

M. Rolando considers that his experiments have proved that the medulla oblongata is the centre of sensibility, the focus and source of life. Treviranus regards it as the centre of animal life, at least. Fodera deems it to be the "hypomoclion" or under lever of life. It is, according to him, the main exciter of respiration, circulation, digestion, and of voluntary motion; the influence which it exerts, proceeding in an especial degree from the point whence the pneumo-gastric nerves arise; this point is the line of demarcation between the medulla oblongata and spinalis. The portion of the medulla situated below this point serves only to transmit impressions received; and the portion of the medulla oblongata situated above this point, is not the source of any influence, nor the centre of any action. The brain and cerebellum are only appendages to the medulla oblongata; and all irregularities of the sentient and moving powers, which are observed in diseased states of the mass of the encephalon are referable to sympathetic derangements in the functions of the medulla oblongata; for this latter part, is invariably the only real and true seat of palsy. According to M. Serres, the whole encephalon is endowed with sensibility, but the medulla oblongata is the principal seat of it. M. Flourens places the source or centre of vitality, and of the nervous power, in the medulla oblongata. All other parts of the nervous system, he says, are dependant on it. If the medulla be divided below the point at which the pneumogastric nerves arise, it dies, and the encephalic mass remains alive; if the section be made above this point, the very reverse ensues. Hence this point is termed by him the vital knot, the central bond of the nervous system, the base of the nervous tree, the encephalon representing the trunk, and the spinal cord and nerves, the roots of it. Respiration altogether depends primarily upon it; and it is not less indispensable for voluntary motion.

To none of these doctrines do we subscribe; the source and centre of life do not reside in, nor depend upon, any special organ, or set of organs, but each part is endued with vitality, and this vitality is a result of its organisation. Neither animal nor organic life has an exclusive centre of influence or acting power; and, therefore, sensibility in its widest acceptation has no special, or particular seat. Every system, nay, every part of any and more especially of the nervous system, has in fact an independent existence or vitality, and hence we cannot admit to any part exclusively what some authors have called "primordiality of action." Before we proceed further in our account of the functions of the medulla oblongata, let us determine what is the precise portion of the nervous system to which that term is to be applied. The older anatomists, and the most celebrated modern ones, include not only the rachidian bulb, or tail of the medulla oblongata, but also, the tuber annulare, and the crura cerebri et cerebelli; but we object to this classification, and while we are inclined to restrict the appellation to the rachidian bulb, and the ganglions which it comprehends, viz. the pneumo-gastric, olivary or vocal, the acoustic, or auditory, and the gustatory or trifacial ganglions, we annex to them the optic and olfactory bodies or lobules, which have usually been considered as belonging to the cerebrum. The reasons of our disjoining the tuber and the crura cerebri and cerebelli are that their organisation, their mode of development, and their relative bulk, as well as the results of experiments upon them, do not agree with the structure, growth, or functions of the medulla; whereas, in these very particulars, there is a striking resemblance between the optic and olfactory lobules, on the one hand, and the rachidian bulb on the other; and we have therefore associated them together. The medulla oblongata, as thus defined, is, as it were, superadded to the spinal marrow, and the functions of the one are analogous to, but are more complicated and more perfect than those of the other; and again it is subordinate, alike in organisation, position, and complexity of function, to the other parts of the encephalic mass. The medulla oblongata is to the organs of special sense what the spinal cord is to the organs of general sensation and locomotion, (the skin and ordinary muscular apparatus.) The former, viz. the organs of the four special senses are provided not only with ganglionic nerves (for the purposes of organic or nutritive life) and nerves of general sensation and of general muscular motion, but also with two sets of particular or superadded nerves; the one being destined to transmit peculiar impressions from the organ to the nervous centre; the other, to receive the motive or existative influence from this centre, and to convey it to the organ. Now with these nerves of special sensation are associated certain peculiar or proper motor nerves; thus the hypoglossal is associated with the lingual, the third and fourth pairs, the ophthalmic of Willis, and the 6th pair, with the optic; and the facial with the auditory. The sense of smell does not require any general movement, and its muscular apparatus or appendages are imperfect; it is therefore not provided with a special or proper motor nerve, but is supplied with twigs from the facial and trifacial nerves. The relation which we have thus pointed out to exist between the auditory and facial nerves, co-operating simultaneously for the same function, although in a different mode, and the consideration of the mutual relative development of these two nerves, shew us the error of those who have regarded them as quite unconnected with, and independent of each other. In the same manner, the pneumo-gastric nerve, on the one hand, and the spinal, on the other, are in reality only two parts of the same nervous apparatus; the one consisting of sensitive filaments, and the other of motive filaments; co-operating with this apparatus, are other cords of filaments sensitive as well as motor, which proceed from different parts; these cords are the glosso-pharyngeal, the hypoglossal, the phrenic, and the intercostals. The first phenomena, or set of actions, which follow any impression made on an organ of sense, and transmitted along its nerve to its respective ganglion, whether of the medulla oblongata, or spinalis, take place in the locomotive, or muscular apparatus with which the organ affected is more especially associated, certain movements occur, and for the purpose either of defending it from the too powerful impression of the external agent, or of better accommodating and arranging it, for such an impression; thus in the case of the eye, or of the ear, the first movements which follow a noise, or strong light, are certain automatic and involuntary actions of the muscles subservient to the organs of vision, or of hearing; the object of which actions is either to mitigate, or to increase and concentrate the external impressions. Now these actions are under the influence solely of the medulla oblongata; but the perception of the impressions belongs to the brain, and to the brain alone; for the brain is the organ of the faculty which wills, and of the faculty which perceives and judges. We are therefore not to regard the medulla oblongata and spinalis as the seat of special and general sensation; they are, so to speak, points of transition, or organs intermediate between the external part on which the impression is made, and the brain which perceives the impression; the function of the medulla being rather to excite a train of movements (those of general locomotion), destined to withdraw the individual organ, or to approximate it to the external agent, or, at other times, to modify and alter the condition of the organ itself; in short, to determine and regulate those special actions which are necessary to the accomplishment of the functions of sense.

MM. Majendie, Dumeril, and Serres, suppose that the trigeminal nerve is fundamentally the real nerve of all the special senses; that it bestows, by a mode of influence which we do not understand, the special sensibility on the optic, olfactory, auditory, and gustatory nerves; and that frequently, especially in the lower animals, it takes the place, and exercises the functions of these nerves. The following are the chief arguments adduced in favour of these opinions. 1. In the mole, there is no optic nerve, but instead of it, there is a twig of the fifth pair; in fishes, there is no auditory nerve; in the cetacea, no olfactory nerve; and yet these animals have in all probability a certain degree of the corresponding sensations. 2. When the fifth pair is divided, all the special senses are destroyed, or at least enfeebled; when the special nerves alone are divided, the animal still retains in part the capability of perceiving odours, tastes, sounds, and light; but if the fifth pair is divided at the same time, these sensations are utterly and entirely annihilated. 3. Certain of the special sensations may be produced in other ways than the contact or impression of their ordinary stimuli; thus, for example, sound may be heard, although the external ear be plugged up, if a watch be applied to the head, or put between the teeth. 4. The occurrence of amaurosis, anosmia, and agenstia, from neuralgia of the trigeminal nerve. In answer to these arguments, we may state that division of the fifth pair does not immediately and instanter destroy any of the special senses; that a certain interval elapses, and during this interval, certain changes have taken place in the texture of the parts on which the nerve was distributed; thus in regard to the eye, the cornea becomes opaque, the conjunctiva inflamed and the humours muddy: the same results follow any injury or disease affecting this nerve; very different, however, is the effect of the division of the optic nerve; the sight is gone instantaneously and for ever. As to hearing the ticking of a watch, when put between the teeth, it is easily explicable on the well-known laws which regulate the transmission of sonorous vibrations; in a similar manner can we understand that those animals which have no external ear, as seals, moles, fishes, &c., may yet have the sense of hearing.—And again; can we not hear the noise of our own voices, even when the ears are firmly plugged, and excluded from any atmospheric impulsions on the tympanum? We readily admit, that the 5th pair is intimately connected with the functions of the four special nerves; but this connexion is indirect, and arises from the trigeminal being the nerve of general sensibility, and of common mobility to the hand and face; and when it is destroyed, or disused, certain morbid changes take place in the organization of the structures which are dependent upon it. But let not our readers be deceived, and suppose that these organic changes which ensue, are a proof that the trigeminal nerve has any direct influence upon the circulation, absorption, exhalation, and secretions of the organs, either in a state of health, or of disease: all these functions, and all morbid states of them, such as inflammation, ulceration, œdema, &c. are dependent upon, and regulated by, the great sympathetic nerve, which we know to be connected by several twigs with the fifth pair. The only direct result of the injury of the fifth pair, is the loss of common sensibility in the exterior surface of the eye and face, palsy of the muscles of the face and tongue, and an immobility of the eye. It is curious that any lesion of the great sympathetic, even in the neck, is followed by inflammation, dulness and atrophy of the eyes; in short, by all the consequences of an injury of the fifth pair itself.

Tiedmann observes that the great sympathetic, by the influence which it has on the general phenomenon of nutrition, and therefore on the organic condition of parts, and on the functions of these parts, such as the secretion of the humours of the eye, and of the mucus of the nose, must necessarily exert a great control over the phenomena of sensation, more especially of smelling and of sight. We have said above, that Majendie and others assert that the mole has no optic nerve; Carus and Geoffry St. Hilaire, however, are of a different opinion, and state that this animal has at least the orbitary portion of the nerve, that it is of the size of a hair, and is united to the ophthalmic branch of the fifth pair, to form with it the retina. This is still a disputed question; and although Hilaire supposes that the functions of any of the sensorial nerves are never transposed to, nor performed vicariously by, another nerve or nerves, yet we cannot deny, that in the invertebral animals, and even in some families of the vertebral ones, the divisions of the trigeminal nerve appear to supply the place and perform duties of the proper nerves of sense. In those animals which have no olfactory or gustatory nerve, or in which they have been cut, the probability is, that the sensations which belong to these special nerves, are reduced to the phenomena of ordinary and general feeling, or sensibility. There may be, quite possibly, a certain positive action of light on the minute and pulpy twigs of the fifth pair in the eye: for light must be regarded as a species of matter, and must therefore make an impression, however weak, and almost unappreciable, on a sentient part; but this impression is only a modification of the general phenomena of touch. The transformation, therefore, of nerves of general sensibility, into nerves of special sensation, is to be regarded as erroneous and unsatisfactory. Bell and Shaw have pointed out the many fallacies into which Majendie has been led, by confounding the phenomena of special, with those of common sensibility; and Eschricht has lately published a suite of experiments which are at utter variance with the conclusions of the French physiologist.—*Ibid.*

---

### LVI.

### External Use of Cyanuret of Potassium in Neuralgia.

This new remedy is employed in the form of solution in water (four grains to the ounce), and applied to the pained parts in severe neuralgias, migraines, and obstinate nervous headaches. A man applied at La Charité for a tic douloureux, which had tortured him for four months; he was cured in eight hours; but the pain returned a week afterwards, and again it was subdued. He still feels the remains of his former sufferings, but is free from those dreadful paroxysms, whose agony defied all language to express. In another case, under the care of M. Recamier and Trousseau, the patient had been affected for 15 years; every remedy even the division of the upper maxillary nerve had been ineffectually tried. The solution was applied, and, after having been used for ten days, there was decided amendment; the frequency and violence of the paroxysms being very much abated.

II. INTERNAL USE OF THE CYANURET.

Some have supposed that the smallest dose would prove deleterious: this is a mistake. M. Andral, of the Pitié, has given, for several days in succession, one and two grains, without observing any other effects but those which result from the exhibition of a few drops of prussic acid. He uses it in nervous affections, as in palpitations; in one case he gave four grains a day: but it is right to observe, that the patient had previously taken prussic acid in doses of twenty-four drops.—*Bulletin de Therapeutique, Oct.* 1831.

---

### LVII.

### AFFUSION OF COLD WATER IN TETANUS.

THE stream of cold water should be directed chiefly upon the head and spine, and in order to produce its sanative effects, it must be continued till a state of syncope is induced. Every one knows the amazingly powerful effects of this remedy, properly applied, in convulsions and other spasmodic diseases; and Drs. Arnoldi and Doucet, of America, after having used with little success, emetics, calomel, and purgatives, in tetanus, had recourse to the cold affusion till syncope was brought on; the patient is then to be put to bed, covered with warm clothes, and large doses of opium and sal volatile, or Madeira wine, are to be freely given with the view of bringing out a copious perspiration. To those who have experimented with nux vomica, it is known that animals poisoned with this drug, are more quickly and certainly restored by cold affusion, than by any other means.

In one case of opisthotonos, the patient, a girl of three years, was cured by taking a coffee spoonful of the following potion:—valerian, 2 oz.; tartarized antimony, 2 grains; and spirit of mindererus, 1 oz.: she was also immersed in a warm bath, impregnated with mustard. Frictions with camphor, opium, &c., may also be used with advantage.—*Ibid. Nov.* 1832.

---

### LVIII.

### CASE OF LACERATION OF THE WHOLE PERINEUM CURED BY MEANS OF THE QUILLED SUTURE.

A YOUNG female, during a first labour, was delivered by means of the forceps; and unfortunately the perineum was lacerated. Fifteen months after the accident M. Roux first saw her; the edges at that time were perfectly cicatrised, but soft and pliant; the anus and vulva were laid open into one, and the fissure extended to the depth of an inch through the recto-vaginal septum. The sphincter ani had lost its power, and the patient was in the habit of taking opium in order to confine the bowels. One suture was applied to the septum; and then four needles to the wound in the perineum. M. Roux did not find it necessary to make any lateral notches in the lips of the wound, as has been recommended by Dieffenbach. The needles were withdrawn in a few days; but unfortunately no adhesion had taken place. M. Roux then determined to practise the quilled suture, as he thought that the curved needles might be pushed deeper, and made to embrace more of the flesh than the straight ones, and the ligature would not so squeeze and confine the lips of the wound, but would merely draw them together, and retain them in apposition. Before the operation, the patient was kept on a very low diet, for the purpose of inducing constipation: the edges of the wound being made raw by means of scissors and a scalpel, the needles, armed with double ligatures, were then introduced—the needles withdrawn, and the ends of the ligatures tied on either side over pieces of a gum catheter; and, in order to prevent the edges of the wound from being forced too much forward, or from pouting outwards (which is one of the disadvantages of this sort of suture), small separate threads, which had been added to the ligatures, were now tied across the wound, so as to combine the interrupted with the quilled suture. The patient was kept on the most rigorous diet, and the bowels were not relieved till the twenty-second day after the operation, when the adhesion was firm and perfect. A partial unglueing of the wound, next the anus, occurred, but this healed by merely introducing pieces of lint into the rectum, as is done after the operation for fistula ani. Still there was a communication between the rectum and vagina, a small recto-vaginal fistula, which admitted with difficulty the point of the little finger.

The preceding case shews very satisfactorily, that, in many similar instances, the quilled suture, which has been nearly exploded from surgery for many years, is better adapted than any of the other kinds; we conceive that it may be practised, even in harelip, with success, and one very obvious advantage of it over the twisted suture is, that the flesh is not so much twisted and forcibly compressed.—*Ibid.*

---

### LIX.

### Cyanuret of Mercury in Syphilis.

M. Parent lately addressed a memoir on the good effects of this new remedy in venereal complaints, to the Academy of Sciences. He has published 27 cases illustrative of its operation. MM. Larrey and Boyer have been appointed to make a report upon the subject.—*Ibid.*

---

### LX.

### Phlegmonous Erysipelas, treated with large Blisters.

*Case* 1. A man, aged 40, strong and plethoric, labouring under diffused inflammation of the fore-arm and arm, was admitted into Montpellier Hospital. M. Delpech ordered the fore-arm to be enveloped in a blister. On the following day, the swelling and pain were greatly diminished; but the inflammation had extended higher up the arm; a blister was applied to it, and the patient was speedily well.

*Case* 2. Diffused inflammation of the upper extremity had followed venesection; the above treatment was resorted to, and the disease was quite arrested in 48 hours.

M. Dupuytren recommends free and deep incisions in such cases as the above; when the inflammation is seated in the scalp, they afford almost the only chance of saving the life, or, at least, of preventing long-continued sufferings and danger.—*Ibid.*

---

### LXI.

### Treatment of Hooping-Cough. By Professor Autenrieth.

No internal medicine is given, except for contingent symptoms, and the only remedy used is friction with tartar-emetic ointment (ʒiss ad ℥j. axungiæ). A portion, of the size of a nut, is to be rubbed on the epigastric region three times a day. On the second or third day, an eruption of vesicles and bullæ appears; the friction is to be continued; the eruption spreads, and becomes pustular like that of small-pox; the friction may now be applied to any other part, but the eruption on the epigastrium is not to be permitted to pass too quickly off. In order to insure the efficacy of the treatment, we must not be satisfied merely with producing pustules; the rubbing ought to be continued till small ulcerations in the intervals between the crusts appear. The above treatment is to be steadily pursued for 8 or 12 days; if the eruption prove very painful, no application is so good as a hemlock fomentation.

Autenrieth has found this method most successful in two severe epidemics, during which he did not lose one patient. He does not approve of the watery solution of the tartarised antimony, and of the tincture of cantharides, recommended by Struve. Henke, an eminent German physician, has seen numerous cases of pertussis much benefited by a combination of bark and opium; he has also employed, with advantage, various stimulant antispasmodics, as ætherial preparations, ammonia, &c. but very judiciously urges the practitioner to draw the distinction between inflammatory and spasmodic hooping-cough, as it must be quite unnecessary to say, that a plan of treatment applicable to the one form may be most pernicious in the other.—*Ibid.*

---

LXII.

### ON SUGAR, AS AN ANTIDOTE AGAINST POISONING FROM COPPER.

ORFILA, in his celebrated work on Toxicologie, strongly recommended sugar, in all cases of poisoning from any of the salts of copper. He found that, at the boiling point, sugar decomposed the acetate of copper, and converted it into a protoxide, of an orange-yellow colour; but this effect was not produced at lower temperatures; how, then, could it be supposed that the sugar could neutralize the cuprous salt in the stomach? He performed a new set of experiments, and his conclusion from these was, that sugar was no "contra-poison," and that its only effect was to sheathe the irritated surface of the stomach. He subsequently proposed albumen as a useful antidote, as he found that it has the property of precipitating solutions of coppery salts; reducing these to the state of an oxyde, and of forming an insoluble compound with this oxyde.

M. Postel has lately directed his attention to this very interesting subject, and has published his observations in the Journal de Pharmacie. He took two dogs, of nearly equal size and strength—he introduced into their stomachs a drachm of verdigris, mixed with four ozs. of water. In a few minutes, both began to moan, vomited, and passed a stool, which was tinged with blue; he then injected gradually, by means of an œsophageal tube, a large quantity of albumen into the stomach of one of these dogs; and into that of the other a quantity of strong syrup. Both of them vomited, and were purged several times, appeared tolerably easy, and drank freely of water. The first or that to which the albumen had been given, died in the course of the night; the other one recovered. A similar experiment, performed a few days afterwards, gave the same result; but, in a third experiment, the dog to which the sugar was given died, while the other one got well. To ascertain whether MM. Orfila and Vogel were correct in their statements, that sugar has no effects on verdigris, except at the boiling heat, M. Postel performed numerous experiments. He mixed verdigris with sugar, and exposed them to a heat of from 86° F. to 97°. No sooner were they brought into contact, than he perceived a sensible change of colour, and, after a few moments, several points of a yellow hue, which gradually spread over the whole of the mixture; the same result was always obtained. Instead of the common verdigris, he now employed the crystallized acetate; the only difference in the result was, that the colour of the mixture assumed a deeper tinge. Even when the above mixture is exposed to the ordinary temperature of the air, the same phenomena are observed, but only more slowly.

If to a solution of the crystallized acetate of copper, be added a certain quantity of clarified syrup, at the ordinary temperature of the air, the liquor loses its blue colour, and passes to a green hue. In a few minutes, it becomes opaque, and we perceive flocculi through it; these gradually fall to the bottom, and have a deep red colour. If more syrup be now added, the solution becomes almost colourless; the precipitate was found to consist of protoxide of copper. M. Postel wished to ascertain the effects of poisonous doses of copper, when the animal was prevented from vomiting by a ligature put round the œsophagus.

*Exper.* 1.—He injected into the stomach of a middle-sized dog 30 grains of crystallized acetate, dissolved in two ozs. of water; a short time afterwards, four ozs. of brown sugar in four ozs. of water were injected, and the œsophagus tied. The animal remained quiet for 20 minutes; it then struggled to vomit —passed two stools, which were of a blue colour, but did not utter any cry; two hours after the operation, it lay extended, and made no efforts to vomit, and in another hour it died.

*Exper.* 2.—The same steps were followed as in the preceding case, only, instead of the sugar, the whites of four eggs, blended with water, were introduced after the copper; the dog died in five hours after the operation.

The conclusions which M. Postel draws from all his observations are—

1. That sugar decomposes the acetate of copper, not only at the boiling temperature, but at the ordinary one of the atmosphere, although more slowly—that this decomposition proceeds more or less rapidly, according to the concentration of the fluids; and that, under all circumstances, the coppery salt is reduced to a protoxide.

2. That sugar exerts an analogous effect in the stomach, since animals to which it is administered, resist the agency of the poison much longer than when it is not; and since the morbid appearances on dissection differ considerably in the two cases.

3. The morbid appearances, when albumen has been given, are nearly the same as when sugar has been given.

4. Sugar may, therefore, be considered among the direct antidotes against poisoning from salts of copper.—*Ibid.*

### LXIII.

### Visit of M. Clot Bey to the Hôpital St. Louis, and his Remarks on Elephantiasis.

This distinguished physician of the Pacha of Egypt, and founder of the medical college at Abouzabel, near Grand Cairo, recently visited the wards of this large hospital, where more cases of skin diseases are to be seen than in any other. The Baron Alibert, one of the physicians, accompanied M. Clot.

Among the cases was one of elephantiasis of the lower extremities, an affection very common, and indeed endemic, in Egypt. The treatment found most effectual, in that country, is compression with straps of diachylon plaster, The whole limb is enveloped with the straps, which are to be neatly applied, the one overlapping the other, and made to cross on the fore part. The effect of this treatment is to cause a more or less copious exudation from the diseased surface. The straps are to be kept on for a considerable time, and removed when necessary; in removing them, we should divide them across with scissors the whole length of the limb—then the whole may be taken off, like a long gaiter, at once.—*Rèvue Med.*

### LXIV.

### The Variolous Epidemic at Turin, in 1829.

M. Griva, an eminent physician at Turin, has published an interesting memoir on this epidemic. He is a strong advocate for vaccination, and states it as his opinion, that upwards of 4000 inhabitants were saved by its means; that it is a better safeguard against small-pox, than even one attack of small-pox is against another; and that the disease, modified by vaccination, is milder than modified by a previous attack of the variola itself. He points out, with great exactness, numerous forms of variolous and vaccine papules, and also of other eruptions which are apt to be mistaken for these, and urges the necessity of carefully attending to the discrimination of these. He has made numerous experiments on the comparative efficacy of the virus, taken directly from the cow, and of that which is obtained from the arm of a person who has been vaccinated; and draws the conclusion, that its properties are not changed, nor modified, by passing through the human body. He says that vaccination is to be regarded not only as the best prophylactic of variola, but also as a curative means in many other diseases, such as agues, ophthalmias, scrofula, hooping-cough and cutaneous affections, and supports his views by numerous observations from his own, and from the experience of authors.—*Ibid.*

---

LXV.

ON THE USE OF SUBCARBONATE OF IRON IN THE GASTRALGIA OF FEMALES.

THE form in which it is recommended is that of pills, and the dose from 6 to 24 grains in the course of the day. M. Trousseau alludes to the great frequency of gastralgia, in connexion with leucorrhœa and chlorosis, and to the errors committed in practice, by mistaking it for chronic gastritis.—*Archives, Sept.*

---

LXVI.

CASES FROM THE "CLINIQUE" OF DUPUYTREN.

1.—*Perpendicular Fracture and Dislocation of the Lower Extremity of the Tibia; it is also termed "Luxation of the Foot backwards and outwards."*

The limb was at first laid upon the apparatus for fractures of the fibula, and the cushion placed behind, in order to counteract the tendency of the foot to fall backwards. The compressor of Dupuytren was subsequently applied; but gangrene supervened, and the patient died. On dissection was found a perpendicular fracture of the articulating extremity of the tibia, and one portion had been driven forwards and the other backwards.

CASE 2.—*Spontaneous Gangrene of the Leg, with Disease of the Femoral Artery.*

A female, aged 40, had experienced for some time a feeling of numbness in the right leg; gangrene supervened, and, although it was checked by bleeding, the patient died. The crural artery, about the middle of the thigh, was contracted, and contained a filiform clot, of a lively red colour. Higher up the limb, the artery was of its usual calibre, but was hard, incompressible, and plugged up with a coagulum, which extended to the common iliac trunk, and into the internal iliac on the right side.

CASE 3.—*Gangrene of the Foot, supervening almost immediately upon puncturing the Abdomen for Ascites.*

M. Dupuytren was puzzled to explain the cause of this accident.—*Gaz. Med.*

---

LXVII.

POISONING BY SULPHURIC ACID.

A MAN swallowed two ounces of sulphuric acid; and, strange to say, the pain at the epigastrium was very inconsiderable, although all the other symptoms of poisoning from such a virulent agent were strongly marked.—*Ibid.*

---

LXVIII.

FRACTURES OF THE LOWER END OF THE RADIUS MISTAKEN FOR LUXATIONS OF THE WRIST.

M. GOYRAND states, that the injury which so frequently happens from falling on the palm of the hand, and which is characterized by the prominence of the extremity of the ulna, and by the diameter of the wrist-joint being diminished transversely and increased antero-posteriorly, is not a dislocation either of the radio-ulnar nor of the radio-tarsal articulation, but is, in truth, a fracture of the end of the radius. Dupuytren has directed the attention of surgeons to this point. M. Goyrand recommends graduated compresses to be applied to within an inch from the wrist-joint.—*Ibid.*

---

LXIX.

PRECOCITY OF DEVELOPMENT OF THE GENITAL ORGANS IN AN INFANT.

IN this infant, at the time of its birth, the mammæ were unusually large, and the mons veneris "garni" with hair At 3 years of age the catamenial discharge appeared, and has continued regularly to the present time, a period of 18 months.—*Ibid.*

---

### LXX.

### Short-Windedness in the Horse.

This affection is generally dependent upon pulmonary emphysema, either of the vesicular or the interlobular kind. The French veterinary surgeons employ the stethoscope with great advantage in their practice; the feebleness of the respiratory murmur, the friction sound, the crepitant and sibilant râles, along with strong resonance of the chest on percussion, are the diagnostic marks of pulmonary emphysema in the horse, as in man. According as these signs are heard over a great or small extent, so is the disease to be considered general or local.

---

### LXXI.

### Insanity in France.

The number of the females exceeds that of the males, in almost all the lunatic houses throughout France. Between the 30th and 50th year, the influence of age appears to be nearly the same on both sexes; but many more men than women become deranged before the 30th year, and the reverse holds good after the 50th year. Not many cases of insanity occur in young persons under 15, nor among the aged after 60, except of that form which some have termed "dementia senilis." As to the influence of marriage on the frequency of insanity, it is observed that nearly twice the number of cases occur in bachelors as in married men, and more than twice the number in single than in married women. If the cases of insanity are grouped according to the threefold division of mania, monomania, and dementia, we find that more than one-half the number belong to the first class, about a fifth of the number to the second, and only a tenth to the third class, viz. that of idiotcy. Mania often succeeds to monomania, and both these states not unfrequently pass into dementia. It is much more rare to observe the idiot become maniacal, either generally or upon one subject. The most common pathological appearance observed upon dissection, is effusion of a sero-lactescent fluid between the arachnoid and pia mater; sometimes wasting or degeneration, at other times increase in the bulk, of some of the cerebral convolutions is distinctly marked; and in several cases, the morbid alterations have been found well to accord with the phrenological positions of the organs. It is right, however, to remember that, very frequently, not an appreciable trace of disease is found on examination of the brains of insane persons.—*Annal. d'Hygiene, &c.*

---

### LXXII.

### Fracture of Bones by the Action of the Muscles.

A young man, aged 23, while amusing himself by throwing pebbles to a distance, suddenly felt a snap and a severe pain at the lower third of the arm; all the signs of fracture were most apparent; yet he had received no blow, nor had he fallen. The arm was put into splints soon after the accident. Seven months subsequently he entered the Hôtel Dieu; the fracture had never united. The patient was under treatment at the date of the report.

*Remarks by Dupuytren on the precedin Case.* The bones most commonly fractured by mere muscular force are the patella, os calcis, and the olecranon of the ulna. Dupuytren has seen upwards of 20 cases of fractured patella; and the mode in which the accident generally happens is this—a person with a heavy load on his back stumbles forwards; he attempts to recover his equilibrium, and, during the effort, the violent action of the extensors of the knee causes the patella to snap. He has seen the olecranon broken in a young man playing at tennis, while aiming to strike the ball. When the long bones break spontaneously, the cause is, in the majority of cases, to be found in a cachectic state of the system, such as that in syphilitic, scrofulous, scorbutic, and cancerous complaints; hence many authors have said, that a long bone cannot possibly be fractured by the mere action of the muscles in a healthy constitution; but several authentic examples are on record in the Philosophical Transactions and other works. M. Curet once saw the accident occur in a sailor, who was "pissing overboard" from the forecastle while the ship was rolling, the violent action of the muscles of the thigh, to keep him erect, caused the femur to break.

Leveille, in his Nouvelle Doctrine Chirurgicale, mentions the case of a young boy, aged 11, in whom the humerus snapped while he was throwing a pebble.—*Journ. Hebdom. Dec.*

In the Révue Médicale, for Nov. is reported a case of spontaneous transverse fracture of the sternum. The patient, a woman aged 37, of a healthy constitution, was in the act of lifting a heavy weight of olives into a cart; the head and body were forcibly thrown back, to enable her to rest the weight partly on the stomach, and, while making the effort to raise it higher, she felt, as it were, a crack at the middle of the sternum; this was followed by an acute pain, which forced her to press upon the part with her hands. A fracture was at once detected by the surgeon; and the lower portion of the bone was found to be projecting, and the upper one to be depressed. Compression and a bandage were applied, and in the course of a month the union was complete.

*Remarks.* Fractures of the sternum may be occasioned in three ways; either by a direct blow—by a fall upon the back, giving rise to a "*contre-coup*"—and, lastly, by the violent opposed actions of the sterno-pubic and the sterno-mastoid muscles.

---

### LXXIII.

### Death of Dr. Spurzheim.

This distinguished philosopher died at Boston, in America, on the 10th of last November. He had been seized with the symptoms of continued fever about three weeks before: but they were of rather a mild form, and did not excite apprehension till within a few days of his death. The only symptom at all unfavourable, was a restlessness and irritability which could not be soothed; this is not unfrequent, in those whose minds have long been exposed to the "wear and tear" of deep and anxious thought. Like many professional and literary men, he had a great aversion to bleeding and almost all medicines; and it appears that his constitution was easily "impressionable;" a drachm of Epsom salts purged him violently. On dissection, no satisfactory morbid appearance was found in any of the cavities.—*Ibid.*

Our readers may remember the account of Lord Byron's last illness; it agrees in many particulars with the preceding case. Medical men should be much on their guard, in their treatment of literary and scientific men, or, indeed, of any whose minds have often felt the exhaustion of intense thought or of harassing anxiety.—Ed.

---

### LXXIV.

### The "Clinique" of M. Bouillaud, at the Hospitals La Pitié and La Charité.

M. Bouillaud alludes to the great improvements in medical practice since 1816, when Broussais effected a revolution in the doctrines of the schools, and established the sure foundation of physiological medicine. Even those who have been, and still may be, most hostile to it in their writings, adopt its principles at the bed-side of their patient; thus, although they will not admit that typhus is one of the numerous forms of gastro-enteritis; their treatment of it, however, is strictly antiphlogistic. Numerous cases of variola, scarlatina, and rubeola, have been admitted, during the last six months of the year 1832, into the hospital. It is now generally admitted, that the eruption on the skin does not in itself constitute the exanthematous disease; that the eruption is merely one of the elements or phenomena of it, and that, without denying the existence of other simultaneous complications, such as changes of the blood, direct affections of the nervous system, &c. by far the most common and most important is an inflammatory congestion of the encephalic, respiratory, or digestive organs. Now it is proper to observe that the internal phlegmasiæ, which so very frequently accompany eruptive fevers, assume sometimes a special or particular character; thus, the angina in scarlet fever is ordinarily of the diptheritic kind; but yet it would be a most serious error, if we supposed, with some able physicians, that these specialities forbid the employment of anti-inflammatory remedies. In the 13th bed of the ward St. Jean de Dieu, you saw a young man in whom scarlatina was accompanied by an intense diptheritic angina, extreme exhaustion, intermittent pulse, and great dryness of the skin; the prognosis was certainly, to all appearance, most unfavourable. He was bled, and had leeches applied several times to the throat, and all the worst symptoms speedily vanished. Certainly we are not to overlook the various forms which inflammations may assume; but we must be always careful not to lose sight of the very foundation of the disease; the old adage is generally a true one—"la forme emporte le fond."

In the disease which has been called typhus, or typhoid fever, entero-mesenteric fever, dothinenteritis, exanthema intestinalis, and which we have designated entero-mesenteritis, because, in reality, the inflammation of the small intestines and of the adjoining mesenteric glands is the fundamental element and starting-point of the disease, the antiphlogistic treatment has been uniformly and most successfully adopted. We have very seldom employed the chlorurets at the same time; these remedies, however, form excellent adjuvants, in many cases of those fevers which are termed "essential." They were first recommended in the treatise which I published in 1826.

In "*erysipelas*," the treatment has consisted in bleeding, when the disease was severe, and in the exhibition of ipecacuan and the employment of the antiphlogistic regimen when it was mild.

The progress of "*tubercular bronchitis*" was very sensibly arrested, in many instances, by means of a low diet and the occasional application of a few leeches, of the cupping-glasses, and of revulsives. There was one case of partial phthisis (a cavern at the summit of the right lung), which was completely cured in this manner.

"*Partial Dropsy.*" Many examples of this affection have been witnessed by our students; they have been almost all traced to some impediment to the venous circulation, arising from obliteration of the vessels by fibrinous concretions in their cavities. Professor Chomel (Lancette Française, No. 81) mentions a case, in which the œdema was confined to the upper half of the body; see, also, a paper "on the Obliteration of the Veins, and its Influence in producing Partial Passive Dropsies," in the Archives Générales de Médécine, 1824. Several cases of diseased heart, of peripneumony, of effusion into the chest after pleuritis, of hæmoptysis, of typhus fever, of colica pictonum, of jaundice, are detailed; the same general therapeutic means were employed, namely, bleeding, general or local, varying in quantity according to the symptoms, and the other parts of the antiphlogistic regimen, with very striking advantage.

'Tis most gratifying that the indications of cure, in very many diseases, are now so simple and well understood, and that the practice of medicine is becoming every day less and less empirical, and acquiring a more rational and satisfactory character. This change of things we owe to the great mind of M. Broussais, the father of reformed medicine.

M. Bouillaud has used the antiphlogistic treatment in cholera with certainly as great success as can be fairly claimed for any other method proposed. In 34 cases, lately under his care, only 9 died, although 14 were in the last stage of the disease. Numerous cutaneous diseases have been cured, nay, we might rather say strangled, by the repeated application of leeches. In one case of icthyosis, the employment of sulphur-baths was very successful.

Several tertian agues, of one, two, or even three weeks' standing, yielded to one or two general bleedings, without having recourse to the quinine. A case of chorea, of 8 months' standing, in a girl aged 13, received much benefit from a venesection, cupping between the shoulders, baths, and the use of valerian; she entered the hospital on the 21st May, and went out, on the 9th of July, so well that she could work with her needle.

A patient, while in the hospital, was seized with erysipelas of the left forearm and hand, accompanied with violent pyrexia and much cerebral disturbance. He died on the 4th day. On dissection, several enormous abscesses were found under the integuments of the left arm, the veins of which were inflamed. Morbid appearances were discovered in most of the joints of the body.

*Arteritis.* A man, aged 34, had experienced for 15 days a sense of numbness in the right arm, and at times a most acute pain, extending from the hand to the elbow; the limb was swollen, but there was no redness; in the course of a few days, no pulse in the radial artery was to be felt, and the vessel was hard and motionless under the finger. A few days subsequently the humeral artery, up to the axilla, became also pulseless; leeching and fomentations abated the pain, but the pulse did not return; the patient, however, left the hospital nearly well.

"*Exanthemata.*" The measles, scarlet fever, and small-pox have been very prevalent and very fatal in Paris for the last two or three months; the dangerous complications of these diseases are almost always of an inflammatory nature; thus, measles is attended with severe bronchitis, and scarlatina with diptheritic angina, which, in the worst cases, affects the larynx and trachea. The small-pox has frequently been confluent, and the number of deaths has been considerable. [Were we to hazard an opinion on M. Bouillaud's practice, which, on the whole, we much approve of, we should say that large depletion by bleeding, &c. in the early stage of small-pox, or, indeed, of any of the exanthemata, is seldom borne well. In the cases of fatal confluent variola, mentioned in his "clinique," one patient had been copiously bled twice from the arm, and had 36 leeches applied. On dissection, the lungs were found engorged, and the cavities of the heart empty; the other was bled once freely from the arm, upon the second day of the eruption, and when the pulse was only 84 and the skin was moist.

The exanthemata ought not to be too much interfered with; they have a course to follow and the object of a prudent physician is rather to regulate and control, than to arrest or to divert. —Ed.]

*Inoculation of Small-Pox Matter in a Person who had been previously vaccinated.* The virus was taken from some ripe pustules on one of the preceding cases, and inserted into the arm of a young man who had been vaccinated when young, and in whom the cicatrices of the vesicles were very distinct. Four days after the operation he felt himself not well; but there was no fever. On the 5th day, a small papula appeared on the site of the inoculation; it was hemispheric, depressed in the centre, and surrounded by a red areola. On the 6th, several small papulæ, having the same characters, appeared around the first one. On the 7th, the pustules had lost their umbilicated form, and were more rounded. On the 8th, scabs had been formed; and, on the 10th, these had fallen off, leaving slight traces, which disappeared in the course of a few days.—*Journ. Hebdom. Nov. et Dec.*

### LXXV.

### Foreign Body in the Trachea for Five Months.

A child, 5 years old, in frolic swallowed a French bean; immediately all the signs of suffocation came on, and, after continuing for two hours, they ceased, and the little patient was cheerful, and apparently well: but again the same distress in breathing and the shrill croaking voice returned, and again they vanished—these attacks every now and then came on. After the lapse of 5 months, the child was seized with a violent paroxysm of cough and excessive dyspnœa, which had been brought on by leaping, and in the course of a short time the child died suffocated.

On dissection, two pounds of serum were found in the left pleura, and there were numerous adhesions between the lungs and ribs upon this side; the left lung was much congested, and did not crepitate—its colour was a deep purple. Where the bronchus enters the lung, an inflammatory ring of six lines in diameter was observed; here, no doubt, the bean had lodged during the five months, till it had been displaced by the shaking of the body from the leap, into the right bronchus, where it was found hermetically plugging up the tube.—*Rêvue Med.*

---

### LXXVI.

### Epidemics independent of any appreciable Changes in the Atmosphere.

Baron Alibert very justly observes, that we cannot well account for the suddenness of the invasion, the quickness of the transition from perfect health to most formidable and fatal disease, the inefficacy of all prophylactic as well as of curative means and many of those inexplicable symptoms which mark the history of most pestilences, by reference to any alterations in the physical or chemical condition of the atmosphere. He does not deny that these alterations may seriously affect both man and the lower animals, and may considerably modify the type and general character of many of their diseases; but then daily observation abundantly proves that these very alterations may take place rapidly, and to a great extent, without bringing along with them that host of horrors and that desolation which marks the invasion of a pestilential scourge. Tacitus mentions that, during the terrible plague at Rome, in Nero's reign, there was "nulla cœli intemperies quæ occurrerit oculis," but that some earthquakes and dreadful storms had been experienced about that time. The weather which preceded the invasion of the plague in London, in 1665, was pleasant and healthful, according to Sydenham; but while it raged, there was also great mortality of the lower animals, especially of sheep and cows; and it is worthy of remark, that in the years 1664-5-6, three comets had been visible, and Mount Ætna was in a state of constant activity.

According to Fernel, the pestilential dysentery which desolated Europe in 1538-9, was not accompanied by any appreciable changes in the state of the atmosphere; but violent earthquakes and eruptions from Mount Vesuvius occurred at the time. He comes to the conclusion, that it is needless to search for the germ of an epidemic in the air; that it is a poison sent from Heaven upon earth—"inquinamentum a cœlo demissum." In 1580, Egypt was visited by a plague more terrible than any since the time of Pharaoh; in Cairo alone, half a million died in the course of eight months; and, during the same year, Rome, Hamburgh, Lubeck, and many other towns in Europe, were ravaged by a most fatal epidemic catarrh or influenza; there was also great mortality among all sorts of cattle, and, moreover, a comet was visible for two months. An interesting feature in the history of epidemics is, that they are often present in numerous places, widely remote from each other, and each having very different climates, at one and the same time; and, on the other hand, certain places or districts frequently escape, while all around is the scene o their ravages. Similar occurrences are met with in the vegetable world; one field of corn will sometimes be found destroyed by the blight, while the adjacent ones are sound and productive, even although their soil is alike, and they have been treated in exactly the same manner. The plague of 1576 depopulated Verona and Padua, and spared Vicenza, situated between them; on the following year Vicenza was visited, and the two other towns escaped.

Again, sudden and very terrible changes in the atmosphere have been known to occur without inducing any pestilential disease. The famous epoch in the 6th century, when storms and tempests so utterly confounded all distinctions of the seasons, that the end of the world was deemed to be at hand; when the sun became pale, and gave only a feeble light for twelve months —'toto anno eo, lunæ instar sine radiis lucem tristem præbuit plerumque defectam patienti similis,' when eclipses added to the darkness and horror, when the plants of the earth were dried up and withered away, and when famine stalked abroad among the nations, even then no pestilential disease existed. Many other instances might be mentioned, but it seems unnecessary. We are, therefore, justified in our conclusion, that the air may serve as a vehicle for the dissemination of the germs of pestilential diseases, but that it is not the source which gives them birth. —*Ibid.*

---

### LXXVII.

### LECTURE ON TETANUS. By Mr. MORGAN.

THIS terrible disease seldom occurs in this country, except after wounds or contusions. Mr. Morgan observes that, when it comes on after the healing of a wound, there is generally a premonitory pain in the part, or an increase of pain, if the part be not healed. We need not stop to state any of the symptoms of the disease, as they are too well known. Mr. Morgan doubts whether, in the successful cases, the remedies or the constitution effected the cure? He has never seen an instance of recovery from *acute* tetanus, nor *heard* of one! This seems rather strange. Mr. Morgan must exclude printed reports from under the head of *hearing*, otherwise he might hear of numerous cures of acute tetanus, by making one of his pupils read to him for half an hour. We recommend to his perusal Dr. Morrison's little book on the subject. Mr. Morgan considers tetanus as a disturbance in the functions of the brain and general system, caused by their connexion or sympathy with the nerves of the injured part—*and by that only*.

"Now, whether all diseases, and all morbid phenomena, do or do not owe their origin to nervous sympathy *alone*, is a question that will never be settled in our time. So far, however, as my own opinions go, I firmly believe that *every* disturbance in the system may be fairly traced to the sympathy of the brain with a local impression, and not *necessarily* to absorption, or to any other agent. Let me not be misunderstood: I do not dispute the well-known fact of the absorption of morbid matter by the veins and absorbents, nor of the necessary subsequent conveyance of this matter (whether it be gaseous, fluid, or solid) through the whole circulating system; but what I contend for is this —that neither its *contact with the brain*, nor its entrance into absorbent vessels, are *essentially necessary* to its operation on the living body. I believe that the sentient extremities of nerves are the parts upon which an impression is first made; and that the different degrees of morbid susceptibility, observed in different structures, may be more satisfactorily accounted for (whether in reference to the application of a poison to the inner coats of absorbent vessels and veins, or to the cellular textures around them) by the theory of nervous communication, than upon the hypothesis of the *necessity* for absorption and the *contact* of the morbid agent with the brain."

Mr. M. was able to produce every symptom of tetanus in animals, by the introduction of *chetik*, an Indian poison, into wounds. He next endeavoured to counteract these phenomena by the introduction of other poisons of an opposite nature. This was the ticunas.

"I found, that in cases of *artificial* Tetanus, brought on in dogs by the means I have mentioned, I could readily *control* the severity of the spasms, and prolong life by the subsequent inoculation of the ticunas; but that the influence of the remedy depended upon its *early* application. In more than one case, I have perfectly restored the animal to health by bringing this antidote into operation as soon as the *first* effects of the chetik were observed, and by regulating its after-consequences by partially, or altogether, cutting off, from time to time, by means of a ligature, all nervous communication between the wound into which it was inserted and the brain. If this latter precaution was not observed, I always found that the ticunas, being the more powerful poison of the two, inevitably occasioned death in a few minutes. In all cases, I took care to insert a quantity of the tetanic poison more than sufficient to destroy life, provided no remedies had been used."

Mr. M. does not indeed recommend his readers to inoculate their tetanic patients with ticunas. His only object is to draw attention to the possibility of obtaining, at a future time, from some one or other of its (now unknown) ingredients, a useful remedy for disease, instead of a means of destroying life.

"Taking this view of the subject, my own plan of treatment, in cases of Acute Tetanus, has been, and always will be, to exhibit the most powerful remedies, in every possible way, until the constitution becomes obviously affected by them, and not to begin by half-measures. If, therefore, the stomach appears incapable of conveying the influence of a remedy to the brain and nervous system, I consider it right to make use of it in the form of an enema; and, failing in my object, then I would introduce it into the veins by means of injections. I have never yet had a case under my care in which the last mode of treatment became necessary; for, by the use of strong enemas, I have always been able to accomplish my purpose; though in most cases of Chronic, and in all cases of Acute Tetanus, without finding that the obvious and powerful impression, produced upon the system by the use of remedies, was of any avail in checking the progress of the disease.

I have of course met with a few exceptions; but I do not feel justified in recommending as remedies the medicines which appeared beneficial in these instances; for, in other cases I have found them perfectly inert. Of one thing, however, I feel confident; namely, that more hope of relief is to be expected from a steady perseverance in the use of one remedy than in a wavering or confused practice. By a wavering practice different medicines are changed daily, and even hourly; and by a confused one, all manner of powerful medicinal agents (sometimes producing opposite effects) are thrown into the stomach together, without rhyme or reason."

---

### LXXVIII.

### On the Phenomena of Hearing.

"A gentleman was slightly deaf, but with the aid of a trumpet he could hear tolerably well; sufficiently to enable him to collect every word uttered in the debates in the House of Commons. He could distinguish sounds best with the left ear; and into this the point of the trumpet was generally placed. On the occasion of a more than ordinary subject being debated, and his attention completely engaged, his trumpet was accidentally and suddenly thrust into the meatus, to some considerable distance, which caused great inflammation; the effects of which rendered him incapable of ever hearing again with that ear; and the other became so much impaired as to incapacitate him, but with the greatest difficulty, from carrying on a conversation even with the use of the trumpet. I have observed him frequently to apply one extremity of a cane to a piano and other musical instruments, and to place the other between his teeth, and by this conductor to distinguish with great accuracy the air which was being performed. This means of assistance did not long avail; for after a while, and to the time of his decease, (which was some years after this accident,) the conductor became useless, for by it he could neither hear note nor tone."

"A plan which Mr. Abernethy used to recommend to be adopted for the removal of peas and such like from the meatus, and which I have more than once found useful, was that of placing the side of the head, on which the ear is situated that contains the pea, on a pillow, and holding it close whilst the pillow is struck with considerable force, thus to shake it out as it were. I have removed a cherry-stone and a glass bead in this manner. Peas become moist, and swell if they remain any length of time in the meatus, and produce excessive pain, and are with considerable difficulty extracted. The hardened cerumen, if it has long existed in such a state in the meatus, requires some time before it can be completely removed by syringing; and a plan to be observed is, to moisten the meatus and cerumen with a few drops of oil a day or two previous to using the syringe; and a common syringe is of no utility whatever: the one recommended by Mr. Abernethy (and which bears, as characteristic of its superior merits and its inventor, the name of 'Abernethy's Ear Syringe,') is much the best for the purpose."

"The Eustachian tube is sometimes nearly obstructed at its pharyngeal extremity by enlarged tonsils. I remember a case of this kind when House Surgeon of St. Bartholomew's.—A young woman was admitted with enlarged tonsils, which enlargement had existed from birth,—at least she had been deaf in some degree since that period; but when she was admitted as a patient her hearing was much more impaired than it had ever been till within a short time previous. She had had remedies innumerable applied to her ears, and the adjacent parts; such as blisters, setons, various sorts of medicaments applied within the external meatus, syringings, &c. &c. Mr. Lawrence, however, saw that the affection originated from pressure on the pharyngeal orifice of the Eustachian tube, by which it was made impervious, and recommended the excision of the tonsils. The operation was performed by him, and the deafness was immediately and completely removed."

The preceding extracts are from an ingenious Essay on the Physiology of the Organ of Hearing, by Mr. Caswall, of London. It evinces very considerable research, and is creditable to the author's industry and zeal.

---

### LXXIX.

### ON A SINGULAR AFFECTION OF THE ORGAN OF LANGUAGE, PRODUCED BY THE ACTION OF MORPHIA. By WILLIAM GREGORY, M.D. F.R S. E. Sec. Phren. Soc.

"THERE can be no doubt that different remedies produce different effects on the mind as well as on the body; and, if medical men, acquainted with the principles of Phrenology, were to direct their attention to the action of remedies on the minds of their patients, a new and interesting field of inquiry would be laid open, and much light would probably be thrown on many obscure points in the history of mind.

Every one is familiar with the fact, that ardent spirits excite strongly the feelings of those who indulge in them. It is stated in the works on Phrenology, that the predominant organs are commonly excited more than the others; but I think we must all have observed, that this excitement is most frequently observed in the lower propensities and the sentiments, while the intellect rarely participates in it.

On the other hand, the intoxication of opium is generally manifested by an increased vividness of intellectual perceptions, without that activity of the lower propensities especially Combativeness and Destructiveness, so often observed in ordinary drunkenness.

It appears to me, then, probable, that the action of alcohol is directed more to the posterior, and that of opium to the anterior, part of the brain. But opium is a substance so complex, that we can hardly draw accurate conclusions from observations on its action, unless those substances which it contains be separated from each other, so as to avoid the confusion arising from different and even opposite actions going on at the same time.

When morphia, the most active ingredient of opium, is purified and combined with an acid such as sulphuric or muriatic, the resulting salt is an anodyne of very great power and uniformity in its operation: and it seems to me to produce effects on the mind which are well worthy of being studied with attention by those who have the opportunity.

The results which I have the honour to offer to the Society, are derived from experiments, chiefly involuntary, made on my own person.

About two years ago, while occupied in examining opium, and especially the salts of morphia, I had acquired a bad habit of tasting the solutions; and it happened more than once, that, by repeated tastings, I received into the system a quantity sufficient to produce effects which I was at first far from attributing to the true cause. The first effect which struck me was, that, in reading, the words, which I saw distinctly, conveyed to my mind an impression which I could not define, but which was certainly different from the right one. On attending as closely as I could to what passed in my mind, I was conscious of nothing but that the words seemed to have lost their true meaning. When this effect had passed off, I was unable to recall what the erroneous impressions had been. A few days after the first occurrence of this affection, while still engaged in the same experiments, I was suddenly taken ill, and had nearly fainted. On recovering, I observed my eyes to be affected in a way to which I am subject, in common with others of my family, when the stomach is slightly deranged. This affection of the eyes consists in an unpleasant vibratory motion of zig-zag lines before the eye, rendering vision partial, and accompanied by nausea. In ordinary cases, it is soon followed by a headach, confined to behind the eyeball when the sight becomes clear. On this occasion, the affection of the eye was unusually great, which led me to predict a violent headach. In a few minutes the headach came on. It was very severe, and confined to that part of the brain situated behind the eyeballs. As soon as I could see clearly, I was astonished to find that I was affected as I had formerly been in regard to words, but to a much greater degree. Not only was I incapable of rightly reading written language, but words addressed to me conveyed a meaning different from the true one. I think also, but of this I am not certain, that a few words which I spoke were observed to be incoherent. But during the whole of this time, my mind was perfectly clear, and I was quite conscious that the erroneous impressions were confined to the faculty of Language.

I began now to suspect that I had suffered from my imprudence in tasting the solutions; which idea was confirmed when I found that a friend who had accidentally taken a large dose of muriate of morphia, had suffered in a manner somewhat similar to what I have described, in regard to the fainting and sickness produced. This gentleman did not observe any affection of Language, but he was in a state of such complete prostration, that he lay for two days without being able to raise his head, and consequently did not attempt to read. I resolved to abstain in future from tasting solutions of opium; but, from habit, I did taste some about a week after; and, a third time, I experienced the same effects, as to language, but without the headach. Having overcome the habit of tasting, I have not since experienced any thing of the kind.

Here, then, is a very marked derangement of the faculty of Language, amounting to a dissociation of the sign and the thing signified, produced by an overdose of a salt of morphia; and, in the most severe instance, it was accompanied by violent headach in the situation of the phrenological organ of

Language. But, it must be remarked, that, in the other two cases, there was no headach; and that I have frequently had headach in the same situation, from derangement of the stomach, without *observing* a similar affection of Language. It by no means follows, however, that this affection did not exist.

A very interesting question now arises, viz. What is the effect of a moderate dose of the same medicine? And, in my own person, I can state distinctly, that, in this case also, the faculty of Language is affected, but in a very different way. If I take from twenty to thirty drops of the solution of muriate of morphia, it produces, in the course of an hour, a very agreeable state of calm; and, for some hours after, the organ of Language is so strongly stimulated, that, so far from having any hesitation in finding words, I find it difficult to stop when I begin to speak; and I have repeated this experiment, which is attended with no inconvenience, so often, that I am quite confident of the result.

Dr. Montgomery Robertson, in a paper on the salts of morphia in the Edinburgh Medical and Surgical Journal, recommends the muriate to nervous people who have to make an appearance in public, on account of the calm collectedness of mind which it produces. I am disposed to concur in this recommendation for another reason, viz. the stimulus which it affords to Language.

I am inclined to believe, that all the intellectual organs participate in this stimulus. At all events, when under the influence of this drug, I have always observed an increased flow of ideas, and a greater power of following out a train of reasoning; and I have never experienced from it any excitement of the lower propensities. I have often had occasion to remark, that, even when the muriate of morphia does not cause sleep, the patient rises refreshed, although the intellect has been actively employed all night. It appears as if, to use a strong expression, the mind was awake while the body slept. This remark is confirmed by the experience of others.

I consider it, therefore, probable, that the action of morphia is directed to the anterior lobe, and, in some individuals, more particularly to the organ of Language, and that an overdose causes entire derangement of that faculty. These conclusions will have to be confirmed, or otherwise, by the observations of intelligent practitioners. Since writing the above, my attention has been drawn by Mr. Simpson to the description of the effects of opium contained in the 'English Opium-Eater,' and Madden's Travels, both analyzed in this journal.* I shall only remark here, that, in the English Opium-Eater and Mr. Madden, all the knowing organs, except that of Language, appear to have been affected, and the propensities and sentiments do not seem to have participated in the stimulus, which affords a strong and unexpected corroboration of the conclusion to which I have been led by my own observations.

I have only farther to add, that, at the time these observations were made, I was ignorant of Phrenology, and knew nothing of the seat of the various organs, and consequently was utterly at a loss to account for the phenomena.

In my head, the organ of Language is *rather large*."†

---

### LXXX.

### Institute of France.

We propose, in future, to give a short abstract of the proceedings of the Academy of Sciences and of the Academy of Medicine of the French Institute.—

Our readers will thus be regularly made acquainted with the progress and advance of medical science on the Continent, and be enabled to contrast the labours of foreigners with those of our own countrymen. It might be deemed more advisable to have begun with a new year; but the extreme irregularity of the publication of the French jour-

---

* Vol. ii. 428, and iv. 138.

† Phrenological Journal, No. XXXV. p. 161-4.

nals has prevented us, and we were unwilling to have delayed our intentions for other three months.

I. Academy of Sciences—*Sept.*

M. Geoffroy St. Hilaire described a very singular discovery which he had made in the anatomy of the marsupial animals. He found that two canals passed from the interior of the fundus uteri, and opened into the abdominal cavity, conveying into it atmospheric air, which, by means of a very curious arrangement of the labia of the vulva and anus, had been previously introduced into the vagina and womb. The contact of the atmospheric air must necessarily have a great influence on the functions of the ovaria, and on the development of their corpuscula. M. Hilaire regretted that, for the last two years, he had not had an opportunity of repeating his observations on any marsupial animals.

M. Baudelocque presented a new instrument which he had contrived, for the purpose of cutting to pieces in a moment the body of a fœtus during difficult labours. He had used it successfully in two cases.

*October.*

Milk a Remedy for Dropsy.

M. Legrand adduced two cases of ascites—one complicated with hydrothorax, the other with hydropericardium, and both symptomatic of diseased heart, in which the water was quickly discharged by no other means than by drinking largely of milk, after all the class of diuretic medicines had been used without avail. In a third case, one of general œdema, supervening upon convalescence from cholera, the patient was cured by taking every morning, for some time, a few cupfuls of milk. Dr. Kapeler, chief physician of the Hospital St. Antoine, narrated a case of ascites, which occurred in a patient affected with chronic enteritis, and whose health was so weak as to preclude the use of ordinary diuretics, speedily removed in the same way.

Orthopedy.

M. Dupuytren, in the name of the section, or Committee of the Surgery Prizes, made a report on the essays which had been sent in, to contend for the prize of 6000 francs, offered by the Academy in 1830, for the best memoir on the following subject—"To determine, by a series of observations and experiments, what are the advantages and what are the disadvantages of mechanical remedies and of gymnastic exercises, in the treatment of the deformities of the osseous system." The reporter stated that five essays had been sent in to the Commission; but that the authors had not illustrated the subject in the manner which the Academy had desired, and that the Commission, therefore, did not intend to award the prize to any one this year; but that its value should be raised to 10,000 francs, to be given to the best memoir on the same subject in 1834.

*November.*

Prize for Experimental Physiology.

M. Flourens, the reporter, stated that no work purely on experimental physiology had been deemed quite worthy of the prize proposed; but that many of the memoirs were valuable and important, and that, therefore, a gold medal was awarded to each of the following authors—1. M. Carus, for a memoir on the movement of the blood in the larvæ of certain neuropterous insects. 2. M. Müller, for his researches on the structure of secretory glands. 3. M. Ehrenberg, on the organization and geographical distribution of infusory animals. 4. MM. Delpech and Coste, on the evolution of embryos. 5. M. Lanth, on the anatomy of the human testicle; and, 6. M. St. Ange, on the circulation of the blood in the human embryo and fœtus.

Blue Urine.

This phenomena is of so very rare occurrence, that it is not even alluded to in works on the urinary secretion. In 1824, M. Fontanelle analysed some blue urine of two patients, and ascertained that the colour was owing to the presence of a hydro-ferro-cyanate of iron. Soon afterwards, M. Braconnet met with a similar case; he attributed the colour to a peculiar substance, which he called "cyanine." Since his experiments, M. Majon, professor of chemistry at Geneva, and M. Cantri, professor at Turin, have detected the ferro-cyanate of iron in blue urine. It is right to mention that Fourcroy, many years ago, detected this salt in the urine of a woman who was subject to frequent and strong convulsions.

ADJUDICATION OF PRIZES FOR THE YEAR 1832. PHYSIOLOGY. Vide preceding Column. PRACTICE OF MEDICINE.

1. 1,500 francs to M. Rousseau for his experiments on the employment of holly leaves in agues.

2. The same sum to M. Lecanu, for his chemical researches on the blood.

3. The same sum to M. Parent, for his experiments to ascertain at what point the steeping of hemp is injurious to health.

4. 4,000 francs to M. Manec for his work on the ligature of the arteries.

5. 2,000 francs to M. Bennati, for his researches on the voice.

6. 4,000 francs to M. Deleau for his improved method of distinguishing and treating the diseases of the ear.

7. 1,500 francs to M. Merat, for his zeal in extending the use of pomegranate bark in cases of tenia.

8. 1,500 francs to M. Villermé for his researches on the comparative duration of life, and on the frequency of disease in easy and in poor life.

9. 2,000 francs to M. Vitry-le-Français, for his discovery of the alkaline principle, salicine, and of its febrifuge qualities.

The Academy has observed with much interest the improvements introduced into the operation of lithotrity by MM. Jacobson, Heurteloup, Tanehon and Amussat; but defer their opinion on the particular methods till next year.

SUBJECT FOR THE MEDICAL PRIZE OF 1833.

"To determine what are the morbid changes of the different organs in continued fevers; what are the relations between these changes and the symptoms of the diseases; what are the therapeutic conclusions to be drawn from these relations; and lastly, to ascertain what are the physical and chemical changes of the solids and fluids in continued fevers."—*Surgical Prize, vide preceding page.*

10*th Dec.* M. Clot communicated some observations on the Dracunculus, or Guinea-worm, as observed in Egypt. It is developed in all parts of the body, in the nose, tongue, upper extremities, trunk, and scrotum, but much more frequently in the lower limbs. In 1828 M. Clot saw a Negress, in whom a dracunculus had appeared within one of the orbits, and had caused considerable ophthalmia; it moved about between the conjunctiva and sclerotic coats, advancing from the outer canthus towards the cornea, and then turning upwards. Sometimes it could not be seen at all. The whites, who have communications with the Negroes born in Africa, are occasionally affected.

17*th.* Mr. Warden sent a table of the population of the United States of America, according to the last census of 1830. 1st. The total population amounts to 12,856,154, of which number, 10,526,058, are free Whites; 2,010,629 are slaves, and 319,467 are people of colour, and free. 2d. Of the Whites, 5,358,345 are males, and 5,167,299 are females; of those of colour, 153,495 are males, and 165,972 are females; and of the slaves 1,014,345 are males, and 996,284 are females. 3d. Among the whites, there are 5244 deaf and dumb; among the black and people of colour, 684. 4th. Out of the whole population 3983 are blind. 5th. The total number of white centenarians is 508, of whom 274 are men, and 234 are women; being one in 20,720;—whereas, among the negroes, there are 718 men and 668 women, or one in 1450; and among the people of colour there are 266 men and 361 women, or one in every 510 persons. This difference, in respect of longevity, is very interesting.

Dr. Villermé read a memoir on "epidemics considered under their relations to statistical and political economy." According to his researches it appears that in thickly-peopled countries, vaccination does little more than merely displace or retard death ; but that in districts, where the inhabitants may at will extend the cultivation of the soil, or where the means of life exceed their wants, it really and truly has the effect of increasing the population. Even in the former case it is advantageous ; because by prolonging life, it considerably augments the opportunities of production, and thus favours indirectly the increase of the population. But this effect is trifling, in comparison of what is ordinarily ascribed to vaccination.

In unhealthy localities, which are perhaps every year visited by some epidemics, as for example, those in the neighbourhood of marshes, the medium age of the inhabitants is much lower than in drier and more healthy districts; but to counteract this evil, it is found that the people are more prolific, so that the actual population may be nearly on a par. In like manner, the number of births is generally found to be greatly increased, after the devastation of any passing epidemic, and the rate of mortality is at the same time diminished, the people becoming " plus vivaces, ou moins sujets à mourir."

Séance of the 24th.

M. Fontenelle read some extracts from a work lately published by Professor Mojon of Geneva, entitled "Conjectures on the Nature of the Miasm of Cholera." He supports the doctrine, that it is animated, or, in other words, that it consists of innumerable imperceptible animalculæ diffused through the atmosphere. This is an old hypothesis, in regard to contagious diseases, for we find that it was believed in by Lucretius, Columella, Vitruvius, Valisnerius, Lancisi, Scuderi, Hartsoeker, Adam Freer, Linnæus, Legendre, Rasori, Crawfurd, and many others.

M. Leuret informed the Academy of his discovery of the brain having a lamellated structure ;—in every part, it is composed of small laminæ, which are quite distinct and inseparable from each other ; and the exterior surface of the brain is formed by the union and apposition of the peripheral edges of these.

M. Pelletier announced his discovery of "paramorphine" in opium. It agrees with morphine in its elementary composition, but not in its chemical qualities ;—these are so distinctive that it cannot be confounded either with the " codeine" of M. Robiquet, or with any of the other ingredients—its taste is like that of pyrethrum, and it exerts a very energetic influence on the animal economy ; a small dose killed a dog in a few minutes.

M. D'Arcet read a paper on the alimentary effects of the jelly prepared from bones. For the last three years, this food has been largely used at the Hôpital of St. Louis; the cooking apparatus has been in constant operation, night and day, and has supplied in that time 1,059,701 rations of soup, and 2,192 kilogrammes of fat, which is used instead of butter and lard, in preparing ragouts, &c.

Upwards of 29,000 persons have been supplied with this food, and have so much liked it, as to be unwilling to return to the ordinary diet. Thenard, the celebrated chemist, added his testimony to the accuracy of the statements of M. D'Arcet, and stated that he had frequently sent to the hospital of St. Louis for some of the soup prepared there, and had found it both palatable and very nutritious. Majendie and the other physicians of the Hôtel Dieu condemned it; but probably the apparatus which has been employed there, is not a good one, or perhaps the cooks have not paid sufficient attention.

---

### II. Academy of Medicine.

*October*.

Vaccination. M. Bousquet, while he admitted the importance of well-formed pustules on the arm, gave it as his opinion, that the vaccine virus may affect the system and preserve it against variola, even though no pustule be formed. M. Stour stated that some constitutions seemed to require a copious eruption of small-pox, to insure them against a second attack; if the eruption proved scanty, the patient was still exposed to the influence of infection. M. D'Epinal thought that no reliance should be placed on the preservative powers of vaccination, unless the system experience a certain degree of feverishness after the operation; and to induce this, he recommended making numerous punctures in the arm. M. Delens adverted to the occurrence of the well-marked exanthematous fevers, without any rash in the skin; this is not unfrequent in many epidemics of measles; and if it takes place with one of the exanthemata, why should it not with the others, and with vaccinia among the number?

CHOLERA. Is it communicable from the mother to the child at the breast? Four cases were adduced, and in all of these the children escaped; in three of them, the mother recovered; in the fourth she died, but the infant did not catch the disease. Many may hence be inclined to consider the preceding facts, as a strong argument in favour of the non-contagion of cholera.

### THE GUACO PLANT.

The "mikania guaco" is of the syngenebrious corymbiferous family, and of the tribe of the eupatoria. It is an herbaceous plant, although its stem rises sometimes to 30 feet. It has a strong sickening smell, which stupefies serpents. It acts as a sudorific, anthelmintic, and stomachic.

### CONTAGIOUS PALPEBRAL OPHTHALMIA.

M. Piorry read a paper on an epidemic ophthalmia palpebrarum, which he observed in the Hôpital de la Pitié, last August—he considered it contagious; and stated that when ophthalmia appears in situations where typhus prevails, as in crowded and filthy lanes of a city, it often assumes a typhoid character, and is more benefited by local compression than by antiphlogistic remedies.

### ON DIPHTHERITE.

This disease is closely allied to croup, and derives its name from the Greek work "diphthera," which means a membrane or skin, because a fibrinous membrane is formed upon the mucous surface of the palate and air-passages. It is epidemic in the departments of Loiret, Cher, Loir-et-Cher, Indre-et-loir, &c. &c. M. Miguel, a physician at Amboise, proposes in severe cases to perform "transversal" tracheotomy, and then to drop into the trachea a weak solution of nitras argenti. Four cases successfully treated in this way are adduced from the practice of MM. Bretonneau and Trousseau. Numerous experiments on the lower animals have shewn that the instillation of a solution of the nitrate of silver into the trachea is not very dangerous. The chief objection to the propriety of the operation arises from the extreme difficulty of ascertaining whether the false membrane is limited to the larynx, or whether it has extended into the bronchi; under the latter circumstances the practice is quite inadmissible.

### RESEARCHES ON ACEPHALOCYSTES, OR HYDATIDS. By M. KUHN.

The author treats of the primitive and spontaneous origin of these entozoa within living organs; of their re-production, and their faculty of multiplying, in man by their interior surface, in the lower animals by their exterior; and lastly, of the artifice which the living power employs to defend itself against these singular beings, by first forming around them cysts, which isolate them from the surrounding textures; then these cysts are filled up with a substance consisting largely of carbonate and of phosphate of lime, whereby the hydatids are gradually converted into tubercles. These tubercles are however very different from the pulmonary tubercles which give rise to phthisis. MM. Dumeril and Breschet agree with the German naturalists in considering hydatids to be merely organic productions, and not as true animalcula. Percy has seen them move; but this takes place only on the application of heat, whereas other worms contract without this stimulus. M. Itard has long thought that hydatids are a sign of organic decomposition.

Cow-pox.

M. le primer de Tallyrand, in a letter lately addressed to the Academy, says, that the original cow-pox, viz. that which occurs on the udders of cows, has not been seen in England for the last 23 years.

School of Medicine at Abouzabel, in Egypt.

At the "séance" of the Academy on the 13th of Nov. M. Clot, the founder and director of the school, accompanied with twelve of his Egyptian "élèves" was present. These twelve foreigners have been sent by the Pacha of Egypt to complete their medical education in France. Among them, some are Ulemas, and others, although born and educated at Mecca have left their homes, wives and families, to be entered as pupils at the School of Abouzabel; an occurrence unexampled in the annals of Islamism. Their names are—Ahemi El-Rachidi Vlema. Mahomet Mansour. Ibraham En Nabraoni. Hussein El Hibaoni. Issaoni En Nabraoni. Mustapha El Subki. Hussein El-Rachidi Vlema. Mahomet El Chabassi. Mahomet El Succari. Mahomet El Chafei. Ahomet Behit. Mahomet Ali El-Bagli.

M. Clot, or rather Clot Bey (for he has been made a Bey by the Pacha), gave the following interesting details. "After having taken the degree of Doctor, at Montpelier, I went to Marseilles to reside, while there, I was invited to go out to Egypt as a physician; I did so, and soon after my arrival in the country strongly recommended to the Pacha, the establishment of a Council of Health on the model of that at Paris—my advice was followed, both as regarded the councils, and also the organization of hospitals in different parts. To enhance the respect due to professional men, the medical service was put upon the same footing as the military service; and the ranks in the two were assimilated. My next object was to found a School of Medicine; but what difficulties to conquer!! On the one hand how to convey to foreigners the language of a science, so full of technical terms; and, on the other hand, how to commence the teaching of anatomy in a country where the act of merely touching a dead body is deemed the most profane sacrilege. I explained my wishes to Osman Bey, who promised to communicate them to the Vice Roy; but he, although favourable to the plan, was unwilling to interfere with the religious prejudices of his people. I therefore boldly resolved to address myself at once to the Ulemas, or priests; they objected, and gave as their reason, that to cut a corpse was to inflict pain upon it; to this I answered, that if dead bodies really do feel pain, it must be much more severe to be gnawed away by worms than to be dissected by the scalpel. I also pointed out to them how necessary a knowledge of anatomy was to the right understanding and treatment of diseases, and urged the importance and dignity of a profession, at once most useful and honourable, and which would tend so much to augment the credit and influence of the priesthood. They at length consented, and forthwith I began to accustom my pupils to seeing and handling the dead body. All Cairo soon knew that dissection was practised at Abouzabel, and although the feeling against it was strong at first, reason soon overcame prejudice. At the end of the first six months, we had a public examination; and during that time were translated into Arabic, a Treatise on Anatomy, Majendie's Physiology, Begin's Surgical Pathology, the Internal Pathology by MM. Sanson and Roche, and the works on Military and Naval Medicine by Percy and Kerandren, and also Orfila's book on the Recovery of Persons who have Drowned and asphyxiated. We were obliged to coin many new Arabic words to convey the meaning of the originals; and so diligent has been the Academy of translation, that now we have a vocabulary of upwards of 6,000 words, whose meaning is perfectly understood. Attached to the school, is a class of Pharmacy and of Veterinary Medicine; and we propose to add immediately one for

Midwifery, to be attended by Negro and Abyssinian women. The affairs of the school are conducted on the plan of a college, where the pupils are lodged, boarded, clothed, taught, and rewarded, at the expence of the Viceroy. Some of the pupils are Christians; and they are treated quite as well as any of the others. At the time of the glorious expedition into Syria, 250 surgeons were sent from the school to join the army; and during the disastrous invasion of the cholera, the zeal, skill, and kindness of the pupils were most nobly and praiseworthily displayed. In Cairo alone 60,000 were cut off in 29 days; most of the European residents, and among these the medical men, with the exception of two or three, fled from the place. Of the 150 pupils who gave their professional assistance, 30 died. In the village of Abouzabel 900, out of a population of 1,800 inhabitants, perished. The particulars will appear in a Memoir which will be speedily printed at Marseilles.

The object of our present visit to France, is to complete the education of these twelve Egyptians in all the branches of medical science, and to improve their knowledge of the French language, in order to enable them to fulfil the duties of Professors upon their return home. The School of Abouzabel musters already more than 400 scholars; and out of these I selected 16 who appeared to be the most industrious and clever; four of them I left in Egypt to superintend the education of the others during our absence; and twelve have accompanied me to France. The Academy will much oblige me by examining and questioning them, that a correct idea may be formed of the progress which they have hitherto made."

Great applause followed this speech. M. Pariset explained the reason of M. Clot-Bey appearing in the Oriental costume:—" The viceroy of Egypt (said he) has formed very exalted ideas on all matters of religion; and, in testimony of respect for M. Clot's great services, he has honoured him with the dignity of Bey, and requested him, upon leaving Egypt, to bear the garb and insignia of the office in France, that Europeans may know that the Viceroy can reward merit, without expecting a man to abjure his country or his religion. Thus, M. Clot, although a Bey of Egypt, remains still a Christian, and a subject of France." M. Clot added, 'that the Viceroy has uniformly treated him with the highest respect and kindness, and that he had raised the emoluments of his office, independent of those of the professorship, from 8000 to 36000 francs.'

26th. Chégoin communicated a memoir " on some displacements of the uterus, and on the pessaries best fitted for their relief." The preference is given to the ring pessaries, as the author deems it to be of much consequence, that all pressure be removed from the mouth and neck of the uterus. The exact form of the pessary must vary with the state of the displaced parts, and the particular conformation of the patient; the same form will not answer for all; but, as a general rule, the flat, smooth, ring, ivory pessary will be found to be the best: the ring is not equally large at all points; it is better that the posterior half of the ring, or that opposite to the sacrum, be considerably larger than the anterior half.

Dr. Marie presented an interesting specimen of a carbonate of lime calculus, which had been expectorated by a young phthisical patient.

1833—SÉANCE OF 8TH JANUARY.

M. Bally resumed the reading of his memoir on cholera. An instructive discussion followed, whether there be such a species of the disease as dry cholera, or cholera unattended by any intestinal discharges.

M. Gerardin mentioned two fatal cases, in which no evacuations from the bowels had taken place; but all the other symptoms of the concentrated disease were present. In the first, no post-mortem examination was made; and, in the second, the intestines were found distended with the rice-gruel fluid; and, on tracing the rectum, it was ascertained that, for the extent of three or four inches, the gut was so much contracted, that a goose-quill could not be passed through. Now we cannot properly admit that these were cases of dry cholera; as the characteristic secretions had actually taken place into the bowels, and were prevented from being discharged by stool, only in consequence of the spasmodic drawing-together of the rectum. Almost all the members of the Academy agreed in this view, and to none had occurred any case of the disease, in which the bowels were found quite empty after death.

---

### LXXXI.

### Calculus in a Child extracted by Weiss's Forceps — Extravasation of Urine—Cure.*

W. A. æt. 7, admitted Oct. 17, 1831. He had had constant pain and difficulty in making water for more than six months. For two days there had been complete retention. A sound in the urethra detected a calculus one-fourth of an inch within the posterior boundary of the scrotum, where a small circumscribed painful swelling was felt externally. Several attempts had been made to hook it hence with a probe, and to favour its escape by dilating the anterior part of the canal with bougies, &c. The bladder was now much distended, the urine had been retained for forty-eight hours, there was much constitutional excitement, and the portion of the canal where the calculus lay was inflamed, tumid, and painful. Dr. M. had seen a similar case which nearly proved fatal, and Sir A. Cooper has seen two fatal cases of the same description.

"Before attempting to grasp the stone with Weiss's catheter forceps, or to push it back behind the edge of the scrotum, and cut down upon it, I passed a steel sound along the urethra, to ascertain its exact situation. I then found that it was dislodged from its former position, and had passed back as far as the bulb,—probably by the pressure of the boy's fingers during the painful attempts at micturition. From the dilated state of the canal posteriorly, the sound was easily introduced into the bladder, pushing the stone before it. Although the urethra was now freed of its irritating cause, and there no longer existed an impediment to the discharge of the urine, yet I regretted that the stone was again lodged in the bladder, in as much as the operation of lithotomy, which might be required for its removal, was a more serious procedure than cutting down upon it in the urethra. It appeared, however, that the parts were in a favourable state for attempting to seize the stone, which was evidently of small size, and to extract it through the urethra. I therefore passed, with ease, into the bladder, a pair of catheter forceps intended for an adult; and on opening their blades, gave exit to a stream of urine, which from the small size of the catheter portion of the instrument, continued to flow for a considerable time before the distended bladder was perceptibly reduced. After moving about the expanded instrument in the bladder, I could not ascertain whether the stone was laid hold of or not, until I had partially withdrawn it. It passed freely out as far as the posterior edge of the scrotum, when its progress was arrested. I then discovered, by external examination, that the calculus was between the blades of the forceps, which were separated nearly one-fourth of an inch. As the narrowest part of the canal was still to be passed, I considered that the removal of the stone by an incision in the perinæum would be the safest practice. I found, however, on again attempting to withdraw the instrument, that the resistance was comparatively trifling; and as the calculus, so far as could be ascertained by external examination, was fairly embraced, and even covered by the blades of the forceps, I determined to continue slowly and cautiously to extract it. There was some difficulty experienced about the centre of the scrotum, and at the orifice of the urethra; but this was gradually overcome without force, and a stone, broken into fragments, was extracted. The patient complained but little of pain, and not more than three or four drops of blood were lost."

---

* Macfarlane's Reports

Dr. M. ordered an elastic catheter to be introduced as soon as the patient was placed in bed. In this the house-surgeon failed, and at 12, p.m. Dr. M. found that only a small quantity of urine had been passed by the penis, and that there was swelling of the scrotum, which had commenced three or four hours previously. There was evidently urinary extravasation. *Large-sized catheter passed, and to be retained—scarifications—fomentations.* On the 18th the swelling and dusky redness had increased, and extended over the pubes. *More scarifications.* On the 20th the inflammation had extended over the pubes as far as the anterior spines of the ossa ilii. On the 21st the catheter was found obstructed, and the urine passed by its side. On the 23d he had an attack of convulsions, which did not recur, and next day a small stream of urine issued from an ulcerated opening on the dorsum penis, close to the pubes. On Nov. 12th he was dismissed cured.

"When the calculus is small, whether the disease occurs in a child or an adult, I would prefer attempting its extraction by the urethra, to the painful and hazardous operation of lithotomy. When it is too large to pass along the whole canal, it may be brought into the perinæum, cut down upon and extracted. For this purpose that ingenious instrument, the catheter-forceps, invented by Mr. Brodie, and recommended in his valuable Lectures on 'Calculous Disorders,' as published in the Medical Gazette, is preferable to the one employed by Sir A. Cooper. It is, however, liable to several objections; the most important of which are the small size of the catheter part of the instrument, and the impossibility of ascertaining when the stone has been laid hold of. Mr. Brodie, in the only case in which he employed this instrument, states, that when the bladder was empty he endeavoured to close the forceps, but found that he could not do it, the stone being seized. I have only to add, that in the instrument belonging to this Infirmary, which was made by Weiss, it is impossible, when it is in the bladder, to ascertain whether a calculus is between its blades or not."

Last year we saw a man with calculus in his urethra behind the glans. It could be pushed backwards and again brought forwards to its former situation, but it could not be impelled by any pressure from behind through the glans. We made various attempts with probes, directors, &c. accompanied with pressure from behind to hook it out. The attempts were unsuccessful. At length we crushed it by means of a very fine pair of dressing-forceps. Some swelling of the extremity of the penis and of the anterior part of the urethra succeeded. The urethral inflammation passed away speedily, but the prepuce continued much infiltrated, and after a week or so the corpora cavernosa behind the glans felt hardened. In some days more it was evident that there was a collection of matter. This was discharged by a puncture, and the opening soon healed. The cavity of the abscess never communicated with the urethra, but whether it actually extended into the corpora cavernosa, or was merely in the external cellular texture, we cannot pretend to determine.

Dr. Macfarlane relates a case of calculus in the bladder of a female child, three years and four months old. Weiss's dilator was introduced and retained for ten minutes, and subsequently the calculus, as large as a pigeon's egg, extracted with forceps. The child has recovered without inconvenience.

---

### LXXXII.

### Loss of Memory from Old Age, or Accidents.

The following remarks on this subject are taken from the third edition, just published, of Mr. Mayo's Outlines of Physiology.

"It has been remarked, that of the powers of the mind, the memory is the first to decay. Old age, which impairs the mind, makes its first inroad on the memory. Wine, while it raises the animal spirits and stimulates the fancy, at the same time disturbs the memory. After injuries of the head, that have eventually been followed by idiotcy, the failure of the memory has been the first mental symptom observed.

The failure of memory is perhaps sometimes considered the only impairment of the mind, when in fact its other powers are likewise injured, inasmuch as an alteration is much more readily detected in the memory than in any other faculty. It is possible likewise, that in many instances it is more properly the recollection than the memory which suffers; the remembrance is not lost, but the facility of finding it in the mind; so that the change would be more justly stated to be in an impairment of the liveliness and rapidity of association. I have heard it observed of old men, that their memory has become impaired, but that their understanding has remained as strong as formerly, only slower in its operations. A supervening slowness of association would account in these instances for all the phenomena observed.

How much we may remember, which we cannot recollect, is shown by a curious class of cases, several of which have been collected by Dr. Prichard, in his work upon the nervous system, from which I shall extract the following.

"A man was brought into St. Thomas's Hospital, who had received a considerable injury of the head, but from which he ultimately recovered. When he became convalescent, he spoke a language which no one about him could comprehend. However, a Welsh milkwoman came one day into the ward, and immediately understood what he said. It appeared that the patient was a Welshman, and had been absent from his native country about thirty years. In the course of that period he had entirely forgotten his native tongue, and acquired the English language. But when he recovered from his accident, he forgot the language he had been so recently in the habit of speaking, and regained the knowledge of that which he had originally acquired and lost."*

"A student at an university in the United States, who is now one of the most respectable clergymen in that country, possessed a tolerable share of classical knowledge, when the consequences of a fever, which affected his brain, deprived him entirely of his former acquisitions. In fact, he had now become so ignorant, that he was not only unable to read a Latin book, but even knew nothing of the grammar. When he had regained his bodily health, being of a persevering disposition, he began again the first rudiments: every thing was quite new to him: he passed through the accidence and syntax in his grammar, and was learning to construe, when one day, as he was making a strong effort to recollect a part of his daily lesson, the whole assemblage of the ideas which he had formerly acquired and lost, suddenly re-appeared to his mind, and he found himself able to read and understand the Latin authors as he had done before his illness."

The first thing to be satisfied of in all questions of this nature is the precise accuracy of the particular facts. We will suppose these facts to be not merely generally but altogether accurate. We do not think that Mr. Mayo has exactly stated the case. When an old man begins to lose his memory, it is well known, that it is the memory of recent events that is impaired. He remembers full well the scenes of his boyhood. In the case of the Welshman it was the language recently acquired that was forgotten; and this we believe to be usually the case after injuries of the head. Patients do not so well recollect what occurred about the period of the accident, or subsequently, as that which took place at a time antecedent to it. Mr. Brodie mentions an instance of this in his paper on Injuries of the Brain. These facts seem to prove that events which have occurred, or ideas that have been formed at an early period of life, have a stronger hold upon the memory, that is, have made a more lasting impression on the mind, than those presented to it in later years.

But, in reasonings of this kind, we must always be careful not to erect words into things, nor reason on non-

* Dr. Tupper's Inquiry into Gall's System of Craniology.

entities as if they were things which have existence. What is memory? We should say it is the preservation of ideas that have been received through the medium of the senses from without, or of impressions made on the sensorium by the organic sympathies within. Now as early life is that in which the senses are most acute, and the sensorium most apt in the reception of impressions, it is not difficult to conceive that it must be more affected by those impressions, and more lastingly influenced by them, than in later years when the senses are more obtuse, and the impressions which they convey are consequently more feeble.

But this mode of reasoning is inapplicable to the case of the gentleman who forgot the Latin language, after having suffered from a fever affecting the brain, and suddenly regained the recollection of it. This, by the way, seems to us rather favourable to phrenology than to metaphysics, rather favourable to the theory that the brain is an assemblage of organs, than a whole, which, as a whole, must be affected. It is more easy to explain the case on the supposition that a particular portion of the brain was organically affected, that a slight effusion, or some other partial lesion, occurred, than on the supposition that the brain is "one and indivisible," the organ of thought, and that being affected in totality, the power of recollecting one language was lost.

We will, however, look at the case in a more metaphysical point of view. Mr. Mayo adverts to the distinction between remembrance and recollection; the former appears to be the endurance of the idea *in* the mind; the latter, the power of obtaining it *from* the mind. He explains then the case of the clergyman, by saying that, after the fever, the remembrance of his Latinity was still present in his mind, but that he temporarily lost the recollection of it. Analyse this and it explains nothing. Remembrance and recollection are arbitrary terms, and are merely representatives of certain facts. To say then that the clergyman remembered the Latin language, but did not recollect it, is simply to express the fact, is merely to affirm, in conventional language, that the case was such as it was. It is obvious that this is a sort of algebraic expression of facts, but not an explanation of them.

Neither do we think that a slowness of association is an explanation of these phenomena. What does slowness of association imply? It is a nonentity; it is merely the expression of certain phenomena; it is a relative term; which means no more than that one idea presented to the mind does not call up another idea in some degree, or in some manner linked with it, as speedily or as fully in one case as in another. To say then that the clergyman did not recollect his latinity, because he was laboring under a slowness of association, is simply to say, when the term is resolved into its elements, that he did not recollect his Latin. It drives the difficulty a little farther off, but it does no more, at least it appears so to us. For instance. The clergyman did not recollect the Latin language, because certain of the associations connected with that language did not, as under ordinary circumstances they would do, call up the ideas which constitute that language. Does this explain the matter? The memory is made to halt at one point rather than another, but after all it is made to halt, and we know no more of the wherefore or the how, than we did before. We make these remarks, not from disrespect to metaphysics, but merely to put in a prominent point of view the truth which most men, we believe, acknowledge, that when we express facts metaphysically, we simplify their expression, or we generalize it, but we do not usually explain the facts themselves. Mr. Mayo we know to be a good metaphysician, and perhaps he may smile at the imperfect manner in which we have put the case.

In our next we shall take some notice of Mr. Mayo's very interesting and useful work.

---

### LXXXIII.

### Aphonia. Dr. Webster.

Some curious cases of this affection are related by Dr. Webster in our esteemed cotemporary the Medical and Physical Journal, for September last. They occurred in the practice of Dr. W. in the St. George's and St. James's Dispensary. In these individuals, there was principally observed a peculiar affection, amounting almost to a total loss of voice, without any decided proof of disease in the larynx or glottis. From these and other cases, which Dr. W. has observed, he comes to the conclusion, that the complaint is occasioned by a paralytic state of the nerves distributed to the larynx and glottis, from pressure or some diseased condition in the cerebrum. We can only make room for one of the four cases related by this intelligent physician.

" *Case* 1. George Wright, æt. 16, groom. When first seen, this patient had an attack of bronchitis, which was, however, soon removed by the exhibition of demulcent and aperient medicines, and the application of a blister to the chest: on the 14th of January last, he was considered free from all pectoral complaints.

Nevertheless, at this period an affection or weakness of voice shewed itself; and, on the 16th, it became so remarkable that Wright could only, with the greatest difficulty make himself heard by the bystanders, being scarcely able to speak, even in the lowest whisper. Notwithstanding the presence of this symptom, he did not complain of any pain in the throat or chest, nor was there any dyspnœa; but the patient now for the first time mentioned that he had a severe headach, accompanied by drowsiness and deafness; and, on examining the pupils of both eyes, they were observed to be very much dilated, and almost insensible to the influence of light. It was from these circumstances that attention was especially directed to the cerebral symptoms, which ultimately led me to consider this affection of the head to be, in reality, a principal cause in producing loss of voice in this individual; and subsequent investigation, confirmed by the successful treatment adopted, demonstrated the correctness of the pathological views then entertained.

Five grains of the extract of conium were given at night, followed by an aperient in the morning; and a demulcent medicine was also ordered to be taken twice or three times a day.

Little or no benefit, as might be expected, followed this mode of treatment: on the contrary, the loss of voice still continued, and it was latterly even so much affected that the patient sometimes could not articulate a single word; the air during these efforts to speak appearing to pass through the rima glottidis, as if he were blowing a musical instrument, without a note being produced. It also merits observation, that if any alleviation of the pain in the head occurred, and especially if, at the same time, the pupils became less dilated, and were sensible to the impression of light, the voice invariably became stronger and more distinct, thus shewing that the condition of the brain, and consequently the function of the nerves distributed on the larynx and glottis, was materially concerned in the disease; and, therefore, it appeared this affection of the head must first be removed, in order to restore the natural tone and strength of the voice.

In accordance with the above reasoning, a blister was applied to each temple on the 24th, which discharged freely, and the patient, when seen on the 26th, was found to have materially improved; the headach was almost gone, excepting over the right eye; the pupils appeared less dilated, and sensible to light, and the words, when speaking, could now be more distinctly articulated, although still in a low tone: the countenance likewise looked clearer, was not so anxious, and, to use the patient's expression, 'he felt almost quite well,' excepting the continued weakness of his voice.

Extract of conium, which had been taken for the last two nights, was continued, and an aperient mixture prescribed in the morning; at the same time more nourishment was allowed, as the appetite and digestion had improved. On the 18th, the patient felt considerably better; the pupils contracted when exposed to a strong light, and the deafness and headach were almost removed; whilst the tone of the voice was nearly natural, and he articulated words correctly, although not strongly. A week afterwards, he had almost recovered his usual voice, had no head affection; and, when seen for the last time, in the middle of February, he was convalescent."

The other cases are equally satisfactory, and we recommend the subject to the attention of practitioners in general.

---

### LXXXIV.

### Of the Organs of the Human Voice. By Sir Charles Bell, KGH. FRS., &c. &c.

Sir Charles Bell is always sure of being favourably received when he appears before the public as an author. We are not surprized therefore that the present Essay in the Philosophical Transactions should have attracted the notice of most of our contemporaries. We will lay an abstract of it before our readers.

Sir Charles observes, that the subject has been negligently treated, that physiologists have been too much accustomed to the consideration of parts with the view to one function only, that the human vocal organs are remarkable for their complications and combinations, and, finally, that although there is one question to which he would direct particular attention, namely articulate language, still he thinks it best to consider all the parts of the subject systematically. He accordingly divides the inquiry into three heads;—the *trachea*, the *larynx*, the *pharynx*.

And first of the trachea.

"We read that the trachea is formed of imperfect hoops of cartilages, joined by membranes, and that it is flat on the back part, for these reasons: that it may be a rigid and free tube for respiring the air, that it may accommodate itself to the motions of the head and neck, and that it may yield, in the act of swallowing to the distended œsophagus, and permit the morsel to descend. This is perfectly correct; but there is a grand omission. Whilst all admit that a copious secretion is poured into this passage, it is not shown how the mucus is thrown off.

There is a fine and very regular layer of muscular fibres on the back part of the trachea, exterior to the mucous coat, and which runs from the extremities of the cartilages of one side to those of the other. This transverse muscle is beautifully distinct in the horse. When a portion of the trachea is taken out, and every thing is dissected off but this muscle, the cartilages are preserved in their natural state; but the moment that the muscular fibres are cut across, the cartilages fly open. This muscle, then, is opposed to the elasticity of the cartilages of the trachea. By its action it diminishes the calibre of the tube, and by its relaxation the canal widens without the operation of an opponent muscle.

The whole extent of the air-passages opens or expands during inspiration; and then the trachea is also more free; but in expiration, and especially in forcible expectoration and coughing, the trachea is diminished in width. The effect of this simple expedient is to force the passage of the accumulated secretion; which, without this, would be drawn in and gravitate towards the lungs. When the air is inspired, the trachea is wide, and the mucus is not urged downwards; when the air is expelled, the transverse muscle is in action, the calibre of the tube is diminished, the mucus occupies a large proportion of the canal, the air is sent forth with a greater impetus than that with which it was inhaled, and the consequence is a gradual tendency to sputa towards the top of the trachea. In the larynx, the same principle holds; for as the opening of the glottis enlarges in inspiration, and is straitened in expiration, the sensible glottis, by inducing coughing, gets rid of its incumbrance. Without this change of the calibre of the trachea, the secretions could not reach the upper end of the passage, but would fall back upon the lungs."

Sir C. Bell refers to experiments, illustrative of this view, which were made with another object by M. Favier. The tracheæ of birds are formed of cartilages of complete circles, without compressing muscles. "Is this (says Sir Charles) because all the air-tubes of birds are dry, because their lungs are motionless, and because in the air respired in them there is no moisture."

The trachea, and that part of the windpipe between the larynx and the lungs, may be looked on as the portevent, or tube conveying the air from the bellows to the reed of the organpipe. If this vibrated, and gave out sound, it would confuse those which proceed from the glottis. Now the imperfect circles of the tracheal cartilages and their isolation are opposed to sound; but Sir Charles conceives that the thyroid gland, placed like a soft cushion on the trachea, and strapped to it by muscles, is still more operative in producing such an effect. In birds, in which the sounding apparatus is at the lower part of the trachea, and the remainder of the tube does convey sound, the thyroid gland is absent, while the trachea itself is a firm tube, with cartilages of entire circles.

"But it is easy to prove that the trachea has no influence upon the voice. Both in the open pipe or flute, and the pipe stopped at the bottom, as the syrinx, the length determines the note,—lengthening the tube depresses the note, and shortening it makes the sound more acute. A similar effect should result from the elongation and shortening of the trachea, if the changes of the voice depended upon it: but, on the contrary, the trachea is lengthened during the higher note, while it is shortened as the voice descends, and the notes become graver.* I have no ear to determine what harmonic sounds attend the human voice; but supposing that sounds proceed from the trachea, which is shortening, at the same time that they proceed from the upper part of the tube, which is lengthening, it is clear to demonstration that the two portions of the tube can never consent to keep any proportion in their vibrations.

For these reasons I apprehend that in the structure and condition of the trachea, the design manifestly is to suffocate the vibrations of sound, and so to impede the motions originating in the larynx from being propagated downwards."

Sir Charles next proceeds to the consideration of the larynx.

He observes that the common opinion, that the glottis is the primary seat of sound, is confirmed by experiment and by every analogy.

"But to consider the motions of the glottis, and even the modulations of the air in the larynx, as the sole source of sound, would be incorrect. Ferrein described the edge of the glottis as being like the strings of the violin, and the air brushing over it like the bow. But even in that supposition, though the vibration of the string of the violin is necessary to the production of sound, yet that sound receives modification through the form and condition of the instrument. As the same chord, vibrating in the same time, will produce a sound the quality of which varies in different instruments, so will the sound of the chordæ vocales be influenced in the pharynx. As a tuning-fork, or a moveable musical instrument, will have the quality and power of the tone changed by its position and the material with which it is in contact, so will the vibrations of the human glottis be affected by the parts above and against which the sound is directed.

The breath, which plays inaudibly in respiration, becomes vocalized when the ligaments of the glottis, or chordæ vocales, are braced so as to cause the edges of the glottis to vibrate in the stream of air. In a wind instrument, the air must be impelled with a force

* "Fabricius ab Aquapendente, seeing the contraction and elongation of the trachea during the changes of the voice, presumed that these motions must be the cause of them. Dodart shewed the incorrectness of this.

to make the sides of the tube vibrate; so, in the production of sound from the human organs, there must be a certain pressure of the column of air. But in the organs of the voice there is this superiority, that there are not only the means of regulating the pressure of the column of air, but of adjusting the vocal chords, so as to suit them to the most delicate issue of the breath. The metal tongue in the organ-pipe is, by lengthening or shortening it, accommodated so as to vibrate in time with the air contained in the tube. So is the edge of the glottis regulated; but with an apparatus for adjustment the most perfect.

Besides the adjustment of the vocal chords, there is a very superior provision in the motions of the chest which supply the air, to that of any musical instrument. Although the organ has allotted to each note a separate pipe, whose relative dimensions are proportioned with mathematical precision, yet the air propelled through the pipes can never be so regulated as it is by the combination which exists betwixt the motions of the chest and the glottis. The church organ could not be made to approach the precision of adjustment in the human organs, were there as many pairs of bellows as there are pipes, and each adjusted by a weight or spring, to accommodate the pressure of air to the dimensions of the pipes."*

Sir Charles next describes the glottis; it is not necessary for us to follow him in that description. He thinks that the sacculus laryngis has much influence on sound, and, setting aside certain anatomical considerations, it appears to him that the ear-piercing cries of such animals as the Beelzebub ape, in which this cell is large, confirm the notion. Sir Charles has repeatedly witnessed, after wounds in the throat, the motions of the glottis in man, both during simple breathing and in speaking. On every inspiration the glottis is dilated; on the patient's attempting to speak, the glottis has moved as well as the lips, and the motion has been in correspondence with the efforts of the other organs of voice.

"We have already understood the necessity of the tongue of the organ-pipe being adjusted in its length, both to the force of the wind from the bellows, and that it may vibrate in correspondence with the column of air in the tube. Granting that the analogy between this instrument and the organ of the voice is just, we must acknowledge the very superior means possessed by the living parts, of drawing out the margin of the glottis, to that by which the tongue of the organ-pipe is adjusted.

If we should adopt the fancy to compare the membrane which is stretched over the ligament to a drum, then the arytenoid muscles would be the braces to tighten the membrane, and the ligaments would be as the snares on the reverse of the drum. But all such comparisons serve to show that, taking this portion only of the apparatus for the voice, it surpasses every instrument in the property of accommodation—of sounding in unison with the rest of the tube, and with the column of air."

Sir Charles passes to the pharynx. Its anatomical characters we may omit.

The breath is vocalized, it is acknowledged by the larynx, but the musical notes in singing and the vowels in speech are affected by the form and dimensions of the pharynx. When a person who has divided the pharynx and exposed the top of the wind-pipe attempts to speak no sound issues from the larynx, though by great effort a noise may be produced. There can be little doubt that the vibrations necessary to articulate language are influenced by the condition of the walls of the pharynx.

"In this part of the air-passage we shall find an exact correspondence with the flute or pipe, in as far as it is lengthened during the grave sounds, and shortened in the acute. Even if it were proved that the note is made to rise and fall by the contractions of the glottis, the great apparatus employed to move the pharynx cannot be useless. We are countenanced in concluding,

* "Which is attempted in some automata."

that as the tube of the organ is adjusted to the reed, so is the condition of the pharynx made to correspond with these contractions of the glottis. It is impossible to see a singer running up the notes to the highest, without admitting that there must be a powerful influence produced through the alternate shortening and elongation of the pharynx and mouth. To allow the cavity to be shortened in the greatest degree, the larynx is raised, and the lips retracted; on the contrary, the trachea descends, and the lips are protruded, to lengthen the cavities, and to give out the lower or graver notes."

### Of Articulation.

In pronouncing the simple continued sounds, the vowels, and the dipthongs, the pharynx, says our author, varies its form or dimensions without interrupting or cutting the sounds. But Sir C. undertakes to prove that in articulating or forming the consonants the pharynx is a very principal agent, and that this smaller cavity is substituted for the larger cavity of the chest, to the great relief of the speaker, and the incalculable saving of muscular exertion. It is on the principle of the hydraulic press that this is effected, on the principle that its piston being loaded with a weight of one pound, the same degree of pressure will be transmitted to every part of the surface of the reservoir, equal in magnitude to the base of the piston; and vice versâ, that, supposing the power to be employed on the reservoir for the purpose of raising the piston, it would require the weight of a pound on every portion of the superficies of the reservoir, equal in extent to the base of the piston, to raise the piston with the force of one pound. Our ingenious author gives many illustrations from the human body of the operation of this law. We regret that we cannot notice them. However, it is by virtue of this law that the muscle of the glottis, not weighing a thousandth part of the muscles of the trunk of the body, is able to counteract them and close the glottis in spite of the operation of those muscles that tend to expel the air from the chest. Thus we may be enabled to understand that much is gained if the "appulse" necessary in articulation be given by the pharynx, instead of by the greater cavity of the chest.

In a patient in whom the bones of the upper part of the face were lost, Sir Charles Bell saw the operation of the velum palati. During speech it was in constant motion, and when the explosive letters were pronounced, the velum rose convex, so as to interrupt the ascent of the breath in that direction; and as the lips parted or the tongue separated from the teeth or palate the velum recoiled forcibly. The next is a long quotation and yet we know not how we could abridge it.

"In speaking, much of the sound, as of the vowels and dipthongs, is the uninterrupted issue of the vocalized breath, modulated by the passages, and differently directed, but not checked or interrupted. The consonants are the same sounds checked by the tongue, lips, or teeth. At the moment of this interruption, the pharynx, being distended, is prepared to give an appulse by its muscular action exactly in time with the parting lips.

If we grasp the throat whilst speaking, so that the fingers embrace the bag of the pharynx, we shall feel that each articulate sound is attended with an action of the pharynx; and preceding each explosive letter, we shall be sensible of a distention of the throat. By a close attention to the act of breathing, we shall perceive that whilst the distended chest falls gradually and uniformly, the bag of the pharynx is alternately distended and compressed in correspondence with the articulated sounds.

We can now conceive that if each appulse of the breath in speaking arose from the action of the chest, it would be attended with great and unnecessary exertion; since in proportion to the size of the reservoir and the smallness of the tube that gives issue, would be the force required on the sides of the reservoir to produce an impulse along the tube. If each consonant and accented syllable required the action of the whole thorax, we should find that a man, instead of being able to deliver an oration of some hours in length, would be exhausted in a few sentences; like a person who bellows and gives pain by the violence and consequent ungracefulness of his action.

If we enter into a more particular examination of the formation of the consonants, we shall perceive that, without the action of the pharynx, those letters must have been mutes, which, through its operation, do in fact give the greatest force and distinctness to language. The circumstance which I have to notice could not altogether escape the observation of grammarians. They speak of the guttural sounds as belonging to the production of certain consonants. Bishop Wilkins expresses this by referring to that *murmur* in the throat before the breath is emitted in pronouncing these letters. Thus grammarians distinguish the mute letter P, which has no sound previous to the parting of the lips, from B, which has a guttural sound before the explosion of the lips.

Had the cause of this sound been investigated, these ingenious men would have presented the subject to us in greater simplicity. 'This guttural sound,' they say, 'is produced by a compression of the larynx or windpipe:' but this has no meaning, and cannot pass for an explanation. This murmur, like all other sounds, proceeds from the vibration of the glottis; but as we have seen, the glottis cannot vibrate without the ascent of the breath through it;—how then is this murmur to be produced when the mouth is closed, and there is no aspiration? The air ascends because the bag of the pharynx, or arrière-bouche, is filling. It is during the distention of the bag, that the breath ascends and produces the sound which precedes and gives the character to some of the explosive letters; and it is this preceding murmur which distinguishes these letters from others, produced by the same position of the 'organs' in the mouth, but which are mute or nasal. Thus the triad of consonants D, B, G (hard), are called semimutes, because, without the assistance of any vowel, they are attended with a faint sound, 'which continues for a little time.' The letters T, P, K are produced by the same position of the organs in the mouth, but they are preceded by no murmur; and therefore it is that they are called mutes: whereas, in D, B, G, the pharynx fills, preceding the parting of the lips. It is this filling of the pharynx, and consequent murmur in the glottis, which gives reason for the grammarians to say that these letters, D, B, G, are accompanied with a sound, though not joined to a vowel, and to call them semimutes.

Grammarians admit 'that the mouth is not the proper organ for producing sound, but only the organ for modulating and articulating the specific sounds;' and having explained the formation of the vowels, they proceed to the formation of the consonants, accounting for their peculiar sounds by the position of the lips, tongue, and palate.

We perceive that their explanation must necessarily be imperfect, owing to their ignorance of the anatomy, and especially of the action, of the pharynx. For example, P, B, and M, they say, are consonants formed by the application of the lips to each other; but this leaves the peculiar character of each letter unexplained, since all three are formed by the lips. The real difference is this: P gives no sound previous to the parting of the lips; it is the vowel abruptly sounded by their separation. B differs only in as much as the sound precedes the opening of the lips in the manner I have just explained; and as the pharynx, after being distended, contracts and forces open the lips, this letter is very properly called explosive. M, too, is in part owing to the articulation through the lips; the sound, commencing in the vowel, is interrupted by the shutting of the lips; after which it continues in a murmur; with this difference from the guttural murmur,—that it ascends into the cavities of the face, the velum being lifted. The same difference is shown in other letters, as F and V. If we attempt to articulate certain letters in a whisper, we shall find how much the distinctness depends on the swelling of the pharynx. In a whisper it is with much difficulty that we can distinguish P from B, or T from D, or G (hard) from K.

Thus we see that the consonants, classed according to their formation in the mouth, have varieties consequent on the action of the pharynx. 1st, The consonants formed by the closed lips; 2nd, Those formed by the meeting of the lips and teeth; 3rd, Those formed by the tip of the tongue and palate; 4th, Those formed by the dorsum of the tongue and palate. All of these admit of variety by the operation of the pharynx and velum; viz. they are mutes, explosive semimutes, and nasal liquids. For example, taking the position of the tip of the tongue against the teeth as forming a consonant, we have T, the mute; D, the semimute, in which the sound precedes the explosion; and N, the sound which rings through the nasal cavities after the closing of the passage through the mouth.

From the same misconception of the actions which combine to form the voice, it may be, that grammarians do not give us a very clear account of emphasis and accent. We perceive that there are two sources of the force with which the words are uttered,—the chest, and the pharynx. The emphatic delivery of several words or syllables must proceed from the forcible expulsion of the breath by the effort of expiration; but the emphasis on the single syllable, and the forcible enunciation of the letter on which the clearness and distinctness, and sometimes the meaning, of words depend, must be produced by the effort of the pharynx."

Sir Charles Bell next advances 15 facts, as proofs of the correctness of the opinions advanced. The conclusions which he draws from the facts in question are these; 1st, That the trachea gives out no sound of itself; 2nd, That when the passage of the trachea is much encroached upon, the column of air is not sufficient to move the cords of the glottis; 3rd, That whatever interferes directly with the motion of the glottis, reduces the voice to a whisper; 4th, That when the larynx is separated from the pharynx delicate sounds are not produced, and therefore an influence of the pharynx upon the stream of air is necessary to the production of such sounds; 5th, That any permanent opening or defect of the velum which shall prevent the distention of the pharynx and the closing of the passage of the nose, renders articulation defective; 6th, That the removal of the cells of the face, equally with their obstruction, deprives the voice of its body and clearness; 7th, That in nervous relaxation of the muscles of the throat, there is sound, but its nature evinces how much the proper action of the muscles is necessary to the voice.

Sir Charles Bell concludes with a summary of his opinions. It would really be a pity to omit it, and this with the quotations we have already made, and the portions of which we have given an abstract, will put our readers in possession of, we might almost say, the whole of this very ingenious and highly interesting essay.

"It is curious, and not without its use, to observe how many parts must conform, and how many actions must accurately correspond, to produce the simplest sound; and how many additional combinations there must be for the formation of articulate voice.

As we may audibly breathe through a trumpet without producing a note of music, so we breathe without the tremor of the glottis to produce voice properly, but only the whisper. To vocalize the breath, there must not only be a certain strength of impulse in the column of air, but there must be an adjustment of the vocal chords in the glottis. The mere impulse of the breath, however forcible, as in sneezing, does not necessarily move the chords of the glottis.

The chordæ vocales being strung by the action of their muscles in correspondence with the forcible expulsion of the breath, they vibrate: this vibration is reverberated on the column of air; and by an adjustment of the passages above, there is a correspondence between the motions of the glottis and the vibrations of the column of air. The breath, thus vocalized, forms the several open sounds or vowels by the change or modulation of the passages: for by the more or less contraction and dilatation of the tube, these sounds are modified; the vibrating air being differently directed, and impelled against different portions of the tube.

The musical notes are in the same way produced by changes in the force with which the voice is propelled, the degree of tension in the chordæ vocales, and the modulation or change in the form of the open passages. There is nothing more surprising than the precision with which the notes of the human voice are produced, as when we hear it rising above the sound of the church organ, the notes more liquid and distinct, and descending in a solfeggio of notes and half-notes, as if each arose from a different pipe, or were struck on a distinct instrument. Yet these falls are consequent on muscular action which alters the diameter and form of the glottis, and the length and diameter of the pharynx. This minute accommodation of action does not merely evince the perfection of the organ, but shows a most surprising command possessed over it: and in this respect the muscular apparatus of the throat does not yield in comparison with that of the eye itself.

Struck with the perfection of the human voice, its precision, expression, and variety excelling the finest instruments mathematically constructed, we have more to admire in the production of those conventional sounds which become the instruments of thought and the source of all we know. Articulation results from a still more complex action of the organs of voice. In speaking, the voice is much influenced by the modulation or varying forms of the open passages, before it is articulated in the mouth; whilst with each motion of the tongue or lips there is a correspondence in the action of the velum and pharynx; so that the compression of the thorax, the adjustment of the larynx and glottis, the motions of the tongue and lips, and the actions of the pharynx and palate, must all consent before a word be uttered.

There is one part of the subject which I have omitted in the body of the paper. In speaking, the play of the chest is not the same as in the common act of breathing: the diaphragm is used less, and the ribs a great deal more. A man, preparing to speak, elevates his chest, whilst the abdomen is drawn flatter; the effect of which is to give more play to the elastic cartilages of the ribs, and the falling of the elevated chest is easy and unembarrassed; whereas, to expel the breath beyond a certain degree, requires the action of the muscles of expiration, and makes the act of speaking still more complicated.

When we think of the number of parts which must combine in office to produce the simplest articulate sound, we see the necessity for a corresponding intricacy of nervous connexions, and are less surprised to find the voice defective through derangement of the nervous system. In a person who stutters, the imperfection is obviously in the power of combination, not in the defect of any single part. Whilst he cannot combine the murmur from the glottis with the action of the pharynx, he can speak in a whisper; that is, he can articulate the faint sound of aspiration, whilst he cannot at the same time vocalize the breath. So he can sing his words without hesitation, or impediment, or spasm; because, in singing, the adjustment of the glottis and the due propulsion of the breath by the elevated chest, are accomplished and continue uninterruptedly. Neither does he experience any distress in pronouncing the vowels and liquid consonants, for the same reason: and if he study to commence his speech with a vowel sound, he can generally add to the vibration already begun, the proper action of the pharynx. Another necessary combination distresses a person who stutters, I mean the actions of the expiratory muscles and those of the throat. He expels the breath so much in his attempt at utterance, that to produce a sound at all, the ribs must be forcibly compressed. To remove this necessity, if he be made to fill his lungs and elevate the shoulders, the elasticity of the compages of the chest will come into play so as to expel the breath without effort, and he will speak with comparative facility and comfort. Accordingly, to commence speaking with the chest fully inflated, to pitch the voice properly, to keep a measured time in speaking, and to raise the voice on a liquid letter or vowel, are some of the common means recommended for the cure of stuttering; and they are certainly those which tend to overcome the difficulty in combining the organs of speech when the defect arises from no disorder or malformation of these organs taken separately."

Sir Charles Bell concludes by observing, that he hopes he may be indulged in a future attempt to unravel the nerves of the neck and throat. We cannot part from the subject without expressing a hope, that this able physiologist will long continue to delight the profession by his original and philosophical investigations.

---

### LXXXV.

History of the Glasgow Royal Infirmary. By *Moses Stephen Buchanan*, M.D, &c. Glasgow, 1832.

This is really an interesting history of an institution of an extremely valuable character. But, interesting as it is, we do not draw attention to it as a history, but because the excellent and able author has mooted some points important to us all. We shall leave to our good friends in Glasgow the rise and progress of the Infirmary to maturity, and merely notice those points, which, as we have already mentioned, touch the interests or feelings of the whole medical community.

The medical periodical press has, of late years, been urgent in its exhortations to hospital surgeons and physicians, to publish reports of the cases which have come under their notice. Some it has coaxed, and some it has kicked, and what between friendly remonstrances and opprobrious denuncitions, many clinical reports have been given to the world. It is a mere act of justice to the surgeons of Glasgow to admit that they began soonest, and have persevered longest, in what cannot but be considered as the good work. This is a tribute which we award to them with great pleasure; and we do it the more openly for this reason. In one way or other, a considerable prejudice has arisen, of late years, against the medical officers of our charitable institutions, and particularly of our hospitals. We will not stop to enquire, at present, why this should be the case, suffice it that it is so. Dr. Buchanan observes—"I know full well, that the task devolved on the physicians and surgeons of this and all similar establishments is one of great responsibility—and in these days of surgical radical reform, it requires no small degree of fortitude, and conscious rectitude, to withstand the lashes which have of late years so fearlessly, so unmercifully, and so ignorantly been applied to hospital surgeons in mass." The worst of it is, that the feeling is rather gaining ground than declining; and whatever may be the cause, the prejudices against hospital surgeons and physicians, particularly in this metropolis, are undoubtedly and deplorably general.

Now the best means of correcting such prejudices are the adoption of measures calculated to impress on the public the belief, that the gentlemen in question have the interests of science and of the profession at heart. Amongst those means, we may fairly include the publication, on an extended scale, of clinical reports; and this brings us back to Dr. Buchanan and the Glasgow Infirmary.

In the Report of that Infirmary for 1827 were the following remarks:—"It having been long matter of regret, that the Reports of the Infirmary had not been rendered so available as they might be to the purposes and improvement of the Glasgow Medical School, it was resolved in 1826, by the Directors of this Hospital, that in future, besides the usual annual record regarding the income and expenditure of the charity, another should be prepared strictly professional, or devoted solely to the medical and surgical details of the hospital."

In accordance with this plan, the following resolutions were adopted by the Directors.

"1st, That, for obvious reasons, it becomes highly desirable that as much light as possible be thrown on the nature, localities, usual progress, and termination of the diseases received into the Infirmary, from Glasgow and the wide district around, so that materials be annually furnished for a medical and statistical history, or medical and statistical tables of the state of health and disease in the said city and district.

2d, That with this view, each Physician and Surgeon, more especially each *Physician*, besides noting, as usual, the names and ages of his patients, be requested also to specify, the following particulars: their trade or occupation, their place of residence,—if in Glasgow, what part of the city; if in the country, what shire or county; whether their abodes be in dwellings apart from others, or in hamlets consisting of a few houses, or in towns or villages; with remarks, if such occur, on the healthiness or unhealthiness of the situation.

3d, That in order to procure an accurate meteorological journal of the weather, a barometer, thermometer, and rain-guage, each of the most approved construction, be established at the Infirmary; that an exact note of the temperature and weight of the atmosphere, of the quantity of rain, and also of the points of the wind be taken, and recorded each day by the apothecary, or some of the clerks appointed for the purpose.

4th, That each Physician be requested to lay before the medical committee on the last days of December, a list verified by his signature, of such diseases as he has treated during the preceding year, with the results as to complete recovery, partial amendment, or death. That each surgeon be requested to deliver at the same period, a similar detail, also verified by his name, and embracing the same circumstances, together with a list of operations performed; also, if the operation admitted of different modes, specifying the mode pursued; as, for instance, if amputation, whether by the circular incision or the flap; if lithotomy, the particular mode preferred, and the instruments employed, &c."

Dr. Buchanan goes on to state that—

"In accordance with the above resolutions of the Directors, a short sketch of the medical and surgical practice of the Hospital was given in the annual report, for the year 1827, and had a similar effort been made in succeeding years, the most valuable results would have been the consequence; most unfortunately, however, a blank took place in the year 1828, and the previous darkness, in regard to the first of these important branches of statistics, has ever since brooded over this great medical storehouse. Not so, however, with regard to the surgical department of the Hospital. The records of the house, 'tis true, do not contain any account either of the surgical or medical practice, after the above period; but at the commencement of the year 1827, the Glasgow Medical Journal started into existence, and has continued ever since to flourish, and in its pages may be found the accumulated experience of all the surgeons who have since successively appeared on the Glasgow Hospital arena."

It is much to be lamented that a plan, in principle allied to the above, is not adopted in every public hospital in the empire; it is much to be lamented, that the medical officers of those institutions do not exert themselves to carry such a plan into effect; and it is to be lamented not only for the sake of science, but for that of themselves. The usual annual reports that emanate from our charities are, professionally speaking, but so much waste paper, hardly interesting to even the lay governors. A statement of receipts and disbursements, an eloquent eulogy of all concerned, a flourish about Providence, and a quotation from St. Paul, are the pith and marrow of most of them. Let such reports continue to be addressed to the Governors in general, but let us, for God's sake, have something more substantial for ourselves.

Such Reports, Medical Reports we allude to, should comprise a general statement of results, and a series of particular facts. The one enhances the value of the other. Whether such Reports should be annual or quarterly, the aggregate of the practice of all, or in turns the record of that of each, we will not at present stop to inquire. Let the medical officers be once persuaded to recognize the principle of medical reporting, and the details will be an after and an easy consideration. Dr. Buchanan makes one or two suggestions with which we must drop the subject for the present.

"In the preface to this work, I remarked, that in making up these surgical Reports for the Glasgow Medical Journal, every surgeon was allowed to consult his own judgment, as to the cases he might deem of most interest, and this liberal scheme I highly approve of; but here my commendation must terminate, for, with one or two exceptions, no tabular lists have been given of the diseases treated, operations performed, or inspections made under the eye of the attending surgeons or clerks. Now, if it is allowed me, I would here suggest the propriety, in future, of every surgeon giving, as an index to his report, 1st, An accurate list of his cases and the results, 2d, A table of his operations and their termination, and 3d, A list of his deaths and the inspections. By a comparison of these, one year with another, one might, at some future period, be able to judge of the success of this or that mode of treatment, or of operating, and thus results of the utmost value might be brought forward. I am well aware of the additional labour, which such a task would impose upon the attending surgeons, whose time is so precious; but even granting that such individuals could not take so much trouble, or *would* not, still, the above interesting tables might be, with much accuracy, drawn up by the surgical clerks of the Hospital, and for all statistical purposes, they would be of equal value to those penned by the surgeons themselves."

The next point on which we touch is that of clinical lectures. Of the advantages to be derived from good clinical lectures there never has been and there never can be any question, nay, more, wherever and whenever good ones are delivered there will always be a disposition evinced by pupils to attend them. If clinical lectures are ill attended we may feel assured that some error somewhere exists. We are not sufficiently acquainted with the economy of our London hospitals to venture to pronounce how the system of clinical lectures works in the metropolis, but of the general truth of the observation we have made, we entertain very little doubt. The plan adopted in the Glasgow Infirmary has been this.

"At various periods during the history of the Glasgow Royal Infirmary, I have remarked that attempts were made to establish regular courses of clinical lectures, but in consequence of the discordant opinions of the two licensing bodies of this city, these instructive practical courses were never, till the year 1829, duly enforced. The Directors of the Royal Infirmary, however, steering clear of both the above parties, in their most unprofessional warfare, judiciously determined to act for themselves, and in their turn became legislators for the benefit of the pupils of this valuable practical school. Accordingly, a senatus consultum of the Directors was promulgated, during the year 1829, of a more important nature than any other recorded in the Hospital history, it was as follows: 'That all students feeing the Infirmary, should also, at the same time, be obliged to fee the courses of clinical medicine and surgery, which thereafter were ordered to be regularly delivered by the attending Physicians and Surgeons on the cases under their charge in all the medical and surgical wards of the establishment.'"

We are for our own parts averse to these compulsory attendances, especially where money is required for them. We are well aware that it will not do to leave all to the discretion and industry of youth, and none are more thankful in after-life for salutary compulsion than those who have submitted to it. But whenever men are forced to pay others for what they may or may not require or wish, an imputation always lies against the remunerated parties, and it is difficult, if not impossible, to divest the public of the idea that they partake of the characters of a selfish monopoly. Such a suspicion against teachers is injurious in a double sense, injurious to the teacher and injurious to the taught.

All that is necessary to secure attendance on clinical lectures, and to effect the good which such lectures can effect, is to attend to these two important considerations;—first, that the lectures be good, and, secondly, that means be adopted for rewarding the diligent, and shewing the careless that they suffer from their carelessness. All other methods are but dangerous nostrums or idle expedients, that stop one hole and make another.

But how are these desiderata to be obtained? In reasoning easily enough, in practice not so easily. The great security for good clinical lectures, is a good system of election of the lecturers. If the latter be appointed to hospitals without reference to merits or popularity, it cannot be expected that their lectures should be in general popular, and there is the source of the evil. But which is the good system of election, and where is it to be found? Is it in London, or in Glasgow, or in France—by interest, relationship, or by the *concours?* Is its essence favouritism, or nepotism, or public examination, or what is it? We may hereafter look at this question more narrowly.

There are not so many obstacles in the way of rewarding the diligent, and virtually punishing the idle. But of this more presently. In the mean time we will return to Dr. Buchanan. Speaking of the advantages accruing to the pupils of the infirmary from the concentration of cases within it, and the out-patient system pursued by the medical officers, he goes on thus:

"There is also another great advantage attending the surgical practice of this Infirmary, which I believe exists no where else, I mean the election from among the pupils, *without fee*, of ten dressers, each quarter, to assist the Surgeons. When this free education is compared with the £52 10s. premium for the obtaining of a dressership to some of the London Hospitals, the result ought to tell on the education of the Glasgow pupil. I might say much here, also, as to the care with which the cases, admitted into the Hospital, are recorded in the Journals, and the daily reports taken at the bed-sides of the patient, often of themselves forming a good clinical lecture. These, the pupil of this infirmary, has not only an opportunity of seeing in the hands of the clerks, who accompany the medical officers in their rounds, but also may be perused by them during four hours of the day in the Hospital. The operations and inspections also are most punctually advertised, in a conspicuous place in the pupil's room, the day before they occur; and at the top of every patient's bed, may be remarked, on a small card, his name, date of admission, disease, diet, treatment, &c."

Dr. Buchanan goes on in another place to observe.

"It has long been proposed, that the clerkships, and all the other professional offices, belonging to this establishment, shall be obtained alone by competition or *concours*, a jury of medical men being the umpires; but I am not such a utopian as not to see faults, even in this French system of election, greater than in that which it has been proposed to supersede. One thing is certain, the talented and long experienced Physicians and Surgeons, whose names are inscribed in the interesting list prefixed to this Chapter, would, almost to a man, have scouted the idea of being tested, by any conclave of medical men, whom the Directors might, in their wisdom, have made choice of for such a purpose; and even their juniors, I am convinced, would not have condescended to be questioned in this, to them, unprofessional-like manner; besides, even granting that a jury is, in many respects, superior to that of open election, I scarcely know one single instance, in the history of this Hospital, where the Directors had to regret an appointment which took place,—and unless something of this nature does occur, my opinion is, that the election of medical men, at least, should continue as hitherto. Even in regard to clerks, I think, that the election by concours, is, in many respects, very objectionable; for it must be remarked, that all these juvenile office-bearers are, when elected, admitted, as it were, into the Hospital family circle; and unless their *private* character is correct, however superior their professional appearance may be, I am of opinion, they ought to be rejected. True it is, some young men have been admitted, by the present mode of election, into this Hospital, who have been a disgrace to those connected with them; but the security for good conduct and eminence, is greater, I think, by the open votes of the Directors, (who become, as it were, sponsors in such cases,) than by those of a medical jury; and therefore, in this respect, I would by no means counsel any change. The only improvement which I would suggest, is in regard to the price paid for bed and board annually by the clerks, which, instead of being £30. I would increase to £50. or £60. at least. Laying out of the question the advantages which these young gentlemen enjoy, in a professional point of view, the bed and board which the Hospital affords, I am convinced, cost more than the sum which I have recommended: this should not be."

Thus it will be seen that Dr. Buchanan makes it a subject of congratulation in one place that the dressers are elected without fee, and in another he laments that the fee of the clerks is not sufficiently high. We agree with the former principle, and disagree with the latter. We think that those who have paid their entrance fee at the hospital, and God knows that is often heavy enough, should thenceforward be free to all the hospital appointments open to them. But an opinion, whether it be our's or that of any other person is, as an unsupported opinion, worth not one straw.

Is it or is it not advisable to encourage merit? Is the substantial encouragement of merit the best way, or is it not, to promote industry? We will venture to suppose that both these interrogatories will be answered in the affirmative. If that affirmative be granted, and it cannot well be refused, it must follow, that to confer house-surgeoncies, dresserships, clerkships, on those who are most industrious and best informed, is a surer, and in all respects a better way of encouraging industry and promoting information, than the practice of giving them by rotation or interest, or both, on the payment of a certain premium.

But how are we to test industry and information? We answer that we should do it in the manner in which it is done at the universities, by examination. There may be objections to the examination of surgeons or physicians who are candidates for public institutions. They may not acknowledge a tribunal competent to try them. But this objection cannot lie against the examination of young men by their teachers. If they can submit to be taught they can submit to be examined, and he who comes forth best from such examination, is surely rather honored than degraded. But Dr. Buchanan objects to this, that private character should be considered as well as public fitness. To be sure it should. None whose moral character is bad should be eligible, and there would be still the same means of ascertaining moral worth or moral turpitude, and more than the same means of stigmatizing the one and encouraging the other, in the system of public than of private elections. We repeat then our firm opinion, entertained for the reasons we have stated, that no fee should be paid by pupils for clerkships, dresserships, house-surgeoncies, or any appointments of the kind, but that these should be the reward of merit, ascertained by a public examination, and encouraged in this public manner.

We purpose to return to the consideration of these questions, which are highly deserving of the attention of all who wish well to the profession.

### LXXXVI.

### SOUNDS OF THE HEART.

THE following explication of the sounds of the heart has been given by Dr. Billing, Physician to the London Hospital, in an Essay read at the Anniversary of the Hunterian Society. The passage came under our notice too late in the quarter to make any observations on the Doctor's statement. The hypothesis (for it is only an hypothesis) is subtle and ingenious. We leave it to the determination of our readers.

"Upon applying the ear, or stethoscope to the chest of a person in health at that point where the heart may usually be seen and felt to pulsate—that is, between the cartilages of the fifth and sixth ribs on the left side, you feel a beat, accompanied by a sound, as if the sound was produced by the blow against the ribs, and immediately after a sound, rather shorter and weaker, appearing more distant, as if produced by the falling back of the body which gave the blow; there is then a pause, rather longer than the time occupied between the first and second sounds. I have elsewhere stated that the beat, or push of the heart, was between the first and second sound, which will be found to be the case, for though the first sound and blow come together, just as when a book or other hard substance is laid against the head, and struck by the hand, yet the push lasts some time,—nearly until the second occurs. Now these phenomena are caused by the ventricles and the valves, for, contrary to the opinion of the immortal Laennec, the auricles have nothing to do with the production of the sounds; the push is caused by the swelling up of the ventricular muscles in their systole to expel the blood; the first sound is caused by the tension produced in the shutting of the auriculo-ventricular valves, and the second sound is caused by the tension produced in the shutting of the ventriculo-arterial valves. The cause here assigned might be thought inadequate to the production of the sound heard, but the little instrument used by gamekeepers to call partridges, may be heard at least at the distance of a quarter of a mile, though consisting only of a bit of bladder stretched over a thimble, a membranous expansion, which is five or six times less than the valves which act together in the heart, and would give a much louder sound if not surrounded by soft parts. The first sound is loudest and longest; the second sudden, and terminates instantaneously, because the semilunar valves have a regular, defined attachment, and are smaller than the auriculo-ventricular valves, the attachment of which to the cordæ tendineæ, and their irregular shapes, render the termination of the sound less abrupt. This is a simple, unsophisticated, explanation of the causes of the beat and sounds of the heart, and you will find that the morbid signs are all explicable as alterations of these."

---

### LXXXVII.

### ST. GEORGE'S HOSPITAL.

### SOME CASES OF DEATH FROM LITHOTOMY.

THE question of death from lithotomy has lately attracted much attention. It is generally believed that when death occurs within a short period after the operation, it is usually occasioned by peritoneal inflammation. In the Lectures of Mr. Brodie, published in the Medical Gazette and in this Journal, this opinion is stated to be incorrect. Mr. Brodie observes that the cause of death in such cases is diffuse inflammation of the cellular membrane, that peritonitis may or may not be present, and that when it does ensue it is secondary to the cellular inflammation, and merely subsidiary to it in producing a fatal termination.

Two provincial surgeons, Mr. Fletcher, of Gloucester, and Dr. Macfarlane, of Glasgow, have lately published some cases of lithotomy, and given the results of their experience to the public.*

* The works of both these Gentlemen have been fully noticed in this Journal.

Mr. Fletcher maintains that peritonitis is frequent, and that much depletion is necessary to extinguish it; Dr. Macfarlane, on the contrary, denies its frequency, but agrees with Mr. Brodie in considering cellular inflammation as a common consequence of the operation, and agrees with him also in thinking depletion rather injurious than beneficial. When discrepancies of this kind occur in statements of facts, the only method of arriving at truth is to observe carefully, and chronicle faithfully, the facts themselves.

When cellular inflammation occurs, is it the result of extravasation of urine, or of the lesions of tissue produced by the operation, independently of any extravasation? When we examine the bodies of those who have died after the operation, it is difficult, in the majority of cases, to determine whether the urine has or has not been effused. The cellular membrane near the wound is more or less sloughy, and beyond the wound is an effusion of pus, or of lymph, or of bloody serum, and it really is far from easy for the inquirer to satisfy himself of the presence or absence of a small quantity of urine. The morbid appearances presented by diffuse cellular inflammation in the pelvis and behind the peritoneum, are precisely similar to those which cellular inflammation offers elsewhere. If a limb in which diffuse cellular inflammation has existed be examined, the following successive stages, or phases, of the inflammatory changes may always be discovered; 1, effusion into the cellular tissue of a bloody serum, more or less abundant, and attended with partial vascularity of the tissue; 2, the cells more or less completely occupied by a gelatinous-looking lymph or thin pus, or a mixture of both, accompanied with some serum, but no vascularity, or very little; 3, the cells completely filled with a deposite intermediate in consistence between pus and lymph, or here with pus and there with lymph, no blood-vessels observable, and the cellular texture more or less dead, apparently from the deposite into its cells having totally destroyed its circulation. These are the appearances presented by a limb in which extensive and severe cellular inflammation has existed, the last-mentioned alterations being present where the inflammation has obtained for the longest period of time, or has been most severe, and the others presenting themselves in an inverted order, as that part is receded from.

Now this is precisely the chain of alterations developed in the cellular membrane of the pelvis and loins after the operation of lithotomy. In the immediate vicinity of the wound, the cellular tissue is actually sloughy; further on it is infiltrated with pus, or merely with lymph, and still more remotely, for instance, in the iliac fossa or the lumbar region, there is merely an effusion of bloody serum.

Thus, there is no appreciable difference between the characters of cellular inflammation in the interior, after lithotomy, and those of cellular inflammation, near the surface of the body, in an extremity. But cellular inflammation may occur, and constantly does so, in a limb, independently of the application of any irritating substance, or diffusion of any deleterious fluid. A compound fracture, a bruise, a wound, any lesion of the tissue, is known to give rise to it in the one instance; and, therefore, we may fairly suppose that the mere injury of the operation may also be sufficient to occasion it in the other. Cellular inflammation does appear to be most frequent after those operations of lithotomy in which the stone is large, and much effort and many trials are necessary to effect its removal, in which, in point of fact, the cellular membrane is much lacerated or much bruised, in which it is most injured, and, therefore, most disposed to inflame.

With these observations, we pass to the narration of some cases which have occurred at this hospital. The two first have been noticed in a former report in this Journal; but we feel disposed to re-introduce a very brief abstract of them, as they bear particularly on the present subject.

Case 1. *Lithotomy—Death on the fifth Day—Cellular Inflammation, with slight Peritonitis.*

William Cockerell, æt. 4½, admitted,

Sept. 26th, 1829, under the care of Mr. Keate, with the usual symptoms of stone in the bladder.

The operation was performed on the 1st October; a double-edged knife was employed. The stone weighed ʒij. gr. x., and the external laminæ consisted of the phosphates.

5, p.m. No pain but in voiding the urine, which passes freely through the wound. Pulse quick—skin rather hot.

10, p.m. Has slept.

*Syrupi papav.* ʒj.

Oct. 2d. Has slept during the greater part of the night—urine retained longer — skin cool — pulse quick — tongue white—bowels not open.

10, p.m. Pulse more accelerated, and skin warmer—there was slight pain in the abdomen at an earlier part of the evening, but it has been quite relieved by the warm bath—no tenderness nor tension on pressure—urine flows freely.

Oct. 3d, 9, a.m. Extreme tenderness over the whole abdomen, without tension—pulse 150, hard and sharp—skin hot—tongue white—bowels not open—urine passed through the urethra this morning—no unusual appearance about the external wound.

*V.S. ad* ℥iv. *Hirud.* viij. *abdomini; posteà fotus. Ol. ric. stat. et donec, &c.*

4, p.m. Tenderness slightly relieved, and more limited to the hypogastrium — pulse quick, softer — tongue white—perspires much—bowels have acted freely. Blood is highly buffed and cupped.

*Rep. Hirud.* x. *abdomini. P. c. fotu.*

9, p.m. Leeches last applied have afforded no relief—pulse weak, compressible—skin clammy—tongue white—urine passes away freely, partly by wound, partly by urethra. The child was now seen by Mr. Brodie, in consequence of Mr. Keate's absence in the country. Mr. Brodie ordered—

*Sago, arrow-root, wine.*

He introduced his finger into the wound; a slight hæmorrhage followed.

Oct. 4th. Has had some sleep—pulse a little fuller (the effect, probably, of the stimuli)—tongue brown in centre, and dry—countenance placid—abdomen becoming tympanitic, without tension—bowels have acted once—urine passed freely.

*Beef-tea, brandy, &c.*

*Vesp.* Has vomited several times—abdomen more distended—pulse weaker—sinking.

He died at 3, p.m. next day.

*Sectio Cadaveris.* Little or no serous effusion in the peritoneal cavity, but partial injection of the peritoneum, and some slight depositions of lymph. Lymph in the cellular membrane external to the peritoneum, in the loins, &c. Serum in the pelvic cellular membrane, but not actually sloughing. The incision had extended through the prostate and its capsule.

CASE 2. *Lithotomy—Death on the fifth Day after the Operation—some Peritonitis and Cellular Inflammation.*

Richard Gray, æt. 4½, admitted June 16th, 1831, under Mr. Brodie, with stone in the bladder. The child was very healthy.

The bowels were opened with castor oil, and the operation was performed on the 17th. Mr. Brodie first attempted to make the incision in the bladder with a common scalpel, but the urine escaped by the groove in the staff; and, after one or two trials, the scalpel was abandoned, and a beaked knife substituted for it. With this the bladder was readily opened. Two small stones, apparently consisting of lithic acid, were extracted. The operation lasted 10 minutes. The patient was put to bed, with a pillow under his knees, and the wound was left to itself.

19th. The patient has hitherto gone on tolerably well, but the pulse has been very frequent; the urine has escaped freely. He has taken only slops, with strawberries, and things of that description. At present, the skin is rather warm—countenance slightly anxious—pulse very frequent—urine said to pass freely—bowels not opened since the operation.

*Haustus sennæ statim, et rep. se re.*

5, p.m. Bowels opened—symptoms continue, with more restlessness, some tenderness of belly, and disposition to draw up the knees.

*H. salin.* ℥ss.—*Vin. ant. tart.* ♏v.—*Liq. op. sed.* ♏ij. 4*tis horis.*

*Calomel*, gr. ij. *stat.*—*Balneum tepid. Postea fotus.*

10, p.m. *Calomel*, gr. ij. *stat.*—*Hirud.* vj. *abdomini.*—*Cal.* gr. ij. 4*tis hor.*

20th, 9, a.m. Slept during the night, quieter this morning—abdomen rather less tender, and knees less drawn up. Pulse continues frequent—lips dry—tongue whitish, with reddened papillæ.

1, p.m. *Rep. H. sal. sine Liq. op. sed.*—*Omr. Calomel.*

6, p.m. More heat of skin—anxiety—small, frequent pulse—apparently more abdominal tenderness—no rigor, nor sickness—delirious, which, indeed, he was to some degree last evening.

*Hirud.* viij. *abdomini.*

11, p.m. Little alteration—rather more depression.

*H. salin.* ℥ss. *c. V. ant. t.* ♏iij.—*Liq. op. sed.* gtt. iij. 3*tiis vel* 4*tis horis. c. Cal.* gr. j.—*Emp. canth. abdomini.*

21st, 1, p.m. Passed a drowsy night, and this morning there is some remission of the symptoms. Sometimes the knees are drawn up, sometimes they are not—position ordinarily on the left side—lies with the eyes closed, but frequently turns his head suddenly from side to side, and cries aloud—looks pallid and anxious—complains of pain when the abdomen is pressed—pulse very frequent—tongue loaded with thick crust—lips desquamating—motions frequent and green—urine escapes freely.

*Hirud.* vj.—*Rep. Haust. sine Liq. op. sed.*—*Rep. Cal.* 4*tis horis.*

The child now sank rapidly, and died in the night of the 22d.

*Sectio Cadaveris*, 24*th*, 1, *p.m.* Intestines rather distended with air—here and there vascular injection, not considerable, of the intestinal and parietal peritoneum. Some turbid, deep yellow serum in peritoneal cavity.

Some serum in the pelvic cellular membrane, around the wound—no distinct evidence of sloughing or urinous infiltration—wound in bladder not very large—nothing further satisfactorily made out.

In the calyx of one kidney, some calculous matter—kidney itself rather soft, and partially vascular.

Case 3. *Lithotomy—large Mulberry Calculus—Death in about* 43 *Hours—Diffuse Inflammation, with commencing Peritonitis.*

George Shorter, æt. 15, a strumous-looking lad, but not otherwise unhealthy in appearance, admitted, Feb. 13th, 1833, under Mr. Hawkins.

He had the usual symptoms of stone in the bladder; the urine was acid. On the 16th, he was sounded by Mr. Hawkins, and a stone was felt. Mr. Hawkins supposed it to be large, but it was placed so far back, that, even with the assistance of a finger in the rectum, he could not satisfy himself on this head.

He was said to have suffered from symptoms of stone since the age of two or three years. He was then sounded by Sir Astley Cooper: but no stone could be felt. He was usually under the necessity of taking much exercise, which occasioned a good deal of suffering. He walked from Brentford to the hospital on the day of his admission.

He was ordered some doses of castor oil, and on the 21st, at 1, p.m. the operation was performed, the bowels having been emptied by an enema early in the morning.

Mr. Hawkins employed a beaked knife, the blade of which was not broad, and the beak was set off at an angle from the flat surface. There was some little difficulty in lodging the beak in the groove of the staff. On the withdrawal of the knife, which was followed by the flow of some, but not much urine, the blunt gorget was introduced, and the staff withdrawn. A more copious flow of urine succeeded the introduction of the gorget. The forceps were introduced upon the gorget, the stone seized, and brought into the wound. Here, however, it slipped from out the forceps, and this happened several times before it could be extracted. The stone was round, about the size of a walnut, of the mulberry species, with many asperities.

9, p. m. Looks pale—pulse rather frequent—skin not hot—has retched and felt sick once, but has not vomited. Not much pain in wound now. About 7, p. m., the urine escaped freely from the wound, but it had not previously been voided; it is dark, bloody, about eight or ten ounces in quantity.

22d. 10, a. m. Passed the night with little sleep. Was sick an hour ago, and vomited mucus tinged green with bile. Pulse 100, not presenting any peculiar character—face pale, and aspect rather anxious—skin little augmented in temperature—tongue whitish. The abdomen is increased in size and rather tympanitic—pain on pressure in the iliac, inguinal, and pubic regions. Passed about half a pint of bloody urine at 7, a. m.

12, *Merid.* The sides of the wound have been separated by the introduction of the finger—no urine was collected in the bladder.

*Hirudines* xx. *hypogastrio.*

At 1, p. m. he was ordered saline effervescing draughts with spirits of nitrous ether and wine of antimony every four hours, and small doses of calomel and Dover's powder every three hours. The abdomen was fomented.

*Vesp.* 8, p. m. Aspect pale, anxious —not the slightest delirium or even hurriedness of manner—respirations rather increased in frequency—pulse rapid, irregular, so weak that it can scarcely be distinguished at the wrist —skin hardly warmer than natural—tongue moist, not coated. He is rather thirsty, and has vomited many times during the day, the matter ejected retaining the same character as before. Abdomen more distended, tympanitic—he dreads pressure on any part of it, especially on the regions formerly mentioned ; the pain is increased by deep inspiration.

He was ordered arrow-root, and was placed in the warm-bath, on his removal from which he felt relieved. The pulse, however, became quite imperceptible at the wrist, and the collapse grew more decided. Between 4 and 5, a. m. of the 23d he died.

*Sectio Cadaveris, 24th, 2, p. m.* Abdomen still distended. On opening the abdominal cavity partial vascularities were observed on the parietal peritoneum, especially towards the pelvis, and on the peritoneum immediately investing the viscera. There was a small quantity, say an ounce, of bloody serum in the peritoneal cavity. The chief inflammatory injection was seen in the pelvic peritoneum, and in that of the loins.

*Wound and Cellular Tissue.* The wound had a dark and sloughy appearance, particularly in the vicinity of the opening into the bladder. Here there was some infiltration of dirty-looking pus and lymph, which was limited, however, to the pelvic cellular membrane. There was infiltration of dirty, bloody serum in the cellular tissue of the right iliac fossa, and in that extending behind the peritoneum as high as the meso-colon. The peritoneum was actually raised from the right iliac fossa by the effusion. It was found impossible to determine whether there was any effusion of urine. The left half of the prostate and its capsule were completely divided, and the vesical wound extended for a quarter of an inch beyond the prostate. This gland was so small that it was utterly impossible for the calculus inclosed in the blades of the forceps to have been withdrawn from the bladder, unless the prostrate and its capsule were either cut or torn. The coats of the bladder were rather thickened.

There was nothing deserving particular attention in the state of the kidneys. The thoracic viscera were sound. The head was not examined.

Mr. Brodie observed in the dead-house that a stone of the form and dimensions of that extracted from the present patient, could hardly be removed from the bladder of a young subject, without a serious risk of a fatal termination. Mr. Brodie has observed that when the capsule of the prostate is cut or torn, and the wound extends to the parietes of the bladder, the chance of cellular inflammation is materially increased. In this instance the vesical wound was necessarily of great dimensions. Mr. Brodie made one or two other remarks deserving of attention. Want of sleep on the night succeeding the operation was, he said, a bad symptom. Pain in the back was another unpleasant symptom, and indicative of cellular inflammation. This patient was reported by the house-surgeon to have laboured under pain in the back. Mr. Brodie remarked that he never knew such cases otherwise than injured by depletion. He said that when these symptoms appear in adults, they are almost always fatal, but that he had seen children recover from them.

Our readers will observe from the foregoing cases that the symptoms may be described to be—pyrexia, characterized by a frequent pulse, a skin not very warm nor flushed, ushered in and accompanied rather by irritability of stomach and vomiting than by rigor—appearing from the first to the third or fourth day after the operation—some tenderness of the abdomen, chiefly observed in the iliac, inguinal, and pubic regions—after a time, some tumefaction of the abdomen and tympanitis, depression of the vital powers, and death within the first seven or eight days after the operation.

On the examination of such cases there is found cellular inflammation more or less extensive, usually accompanied with some peritonitis, but the latter not sufficient to account for the symptoms, nor the fatal termination. It is quite fair to conclude that the peritoneum is affected subsequently to the cellular membrane, and that the inflammation extends to it in consequence of its contiguity only.

Case 4. *Lithotomy—Hæmorrhage after the Operation—Death from Pericardial Inflammation.*

George Brooks, æt. 15, admitted August, 1832, under the care of Mr. Babington, with symptoms of stone in the bladder. He was an errand-boy, and consequently obliged to use much exercise. He was tolerably healthy, but looked pale, and was thin. The pulse was generally frequent, the tongue whitish, with red papillæ.

He had laboured under the symptoms of stone since the age of two years, and had been sounded by many eminent surgeons, but no stone had been discovered. For four years previous to June, 1832, he had experienced a respite from his sufferings, but the symptoms became so severe in that month that he was obliged to relinquish his occupation altogether. When admitted into the hospital, much difficulty was experienced on attempting to introduce instruments into the bladder. An instrument was passed every day for a week, before that difficulty was removed, and then a calculus was felt. It is right to mention that the lad had been subject to epileptic fits.

The treatment after his admission, consisted in the exhibition of opiate injections, and saline medicines, to relieve the urgent and prominent symptoms. After this he was allowed the ordinary diet of the hospital, and medicines were discontinued.

On the 13th Sept. the operation was performed by Mr. Babington. There was some little difficulty experienced in introducing the staff. During the operation the rectum, as not unfrequently happens, was wounded. The incision of the bladder was made with a beaked knife, the forceps introduced on a blunt gorget, and the stone extracted with facility. The stone was nearly two inches in length, and almost an inch in its transverse diameter. After the operation it was thought advisable to lay open the wound of the rectum into the perineal wound. A blunt-pointed bistoury was therefore passed from the perineal wound, through the wound in the rectum into the gut, and, the forefinger of the other hand being introduced into the gut through the anus, the sphincter was divided from within outwards, as in the operation for fistula ani.

Soon after the operation the bladder was injected with tepid water, and a dose of Battley's sedative laudanum exhibited.

*Vespere.* Pulse now frequent, full, jerky—skin warmish—tongue white on the dorsum, red at the sides and tip. In the afternoon, no urine having flowed since the operation, Mr. Pollard, the house-surgeon, introduced his finger into the wound, and broke up some coagulum which plugged it. The urine flowed freely soon afterwards. There is still, 10 p. m., some coagulum in the wound. He was ordered an opiate if he should not be able to procure sleep.

14th, 2 p. m. He slept during the night, and is disposed to be drowsy at present; he had the opiate. At 9 a. m. this morning Mr. Babington examined the wound with his finger. Bleeding to the amount of six or eight ounces immediately occurred, and Mr. Babington plugged the wound with common and blue lint, first washing out the bladder. At 1 p. m. he removed the common, and left the blue lint.

There is now a good deal of coagulum in the wound. Skin warm—pulse 120, not powerful, but jerky. *Catheter introduced.*

10 p. m. Countenance more anxious. Slight tenderness on pressure in the pubic and left inguinal regions. Has voided about six ounces of bloody urine with coagula; it came in gushes, on two occasions, and on the last the catheter slipped out.

*Some blue lint and coagulum removed—catheter re-introduced—warm water injected. Ice has been applied.*

15th, *Mane.* Slept last night—less pyrexia.

*Vesp.* Pulse again 130 to 140—symptoms resembling those of yester-evening. No more hæmorrhage from the wound.

*Bladder again washed out—fæces return with urine by the catheter, and urine is voided by the rectum.*

16th. Pulse very frequent, and weak—urine mixed with fæces, and the fæces with grumous blood.

*Beef-tea—an opiate at night.*

17th. *Vesp.* Aspect anxious, pale—respiration hurried—inspiration imperfect, and attended with pain in both clavicles—slight cough occasionally on full inspiration. He says he has been subject to such symptoms previously to an epileptic attack. Pulse galloping—tongue moist—skin warm. Slight pain on pressure above pubes, and in iliac regions.

On the following morning, 18th, he was no better. The pulse was 130 to 140—there was pain on pressure of the sides of the chest. He was said to have slept well in the night. He had passed some clear urine.

*Hirud.* xviij. *abdomini.*

*Merid. Emp. canth. abdomini. Mist. camph. c. Liq. op. sed.* ♏viij. 6*tis horis.*

*Vesp.* Less pain—no material alteration.

19th. *Mane.* Had a profuse perspiration last night. Slept well, and now dozes much, apparently in consequence of the opium. Respiration still imperfect and hurried—pain complained of under the lower half of the sternum—pain also in the sides of the neck. Pulse very rapid, tumultuous—pulsation of the vessels at the root of the neck very perceptible.

*Merid. Hirud.* viij. *pectori. Rep. Haust.* 4*tis. horis.*

*Vesp.* Quieter—has had again profuse perspiration to-day—tongue white, patchy.

*Emp. canth. dorso—adde H. tinct. digitalis,* ♏viij.

20th. 4, p. m. Passed a very bad night, probably from the irritation occasioned by the blister—had a profuse perspiration during the night, and again this day. Symptoms much the same—the tongue has a peculiar appearance, there are patches of white upon the dorsum, and a macerated appearance of the remainder; an appearance very similar to that frequently observed in cases of the purulent deposites. The aspect of the patient is also very similar to that of patients labouring under that affection.

On the following day he had again perspiration. On the 22d, he was ordered—

*Pulv. digitalis,* gr. j. *Ext. conii,* grs. viij. 8*vis. horis.*

The symptoms were somewhat relieved until the afternoon of the 24th, when they again became aggravated. The action of the heart was most tumultuous; he seemed to prefer lying on the left side; he had emaciated greatly. Since the 21st he had had no sweating. We should state that for many days there had been nothing about the wound or the urinary organs to deserve particular mention.

On the morning of the 25th the symptoms were again slightly mitigated. We examined the anterior part of the chest, for we did not wish to disturb the lad by moving him so as to apply the stethoscope posteriorly. The respiration was puerile anteriorly on both sides; stethoscopists know that this may exist, with fluid in the dependent parts of the pleural cavity, or even alterations of the posterior part of the lungs; consequently, by itself it proves little or nothing. The impulse of the heart was confused; no distinct bruit-de-soufflet—some apparent retraction of the cardiac intercostal spaces.

Unfortunately we possess no further notes of the case and must therefore write from recollection. We will merely add, that the symptoms were not materially relieved by any means employed, and the patient died in some days after the last report.

On dissection the heart was found pretty generally adherent to the free pericardium, partly by old adhesions, partly by recent lymph. There was active hypertrophy of the left chambers, dilatation of the right. The lungs were generally adherent, by old attachments, to the costal pleuræ, their further condition we do not exactly recollect.

There was no collection of matter, nor was there any affection of the veins discovered about the pelvis.

It is right to mention that this boy had formerly been subject to acute rheumatism, a disease well known to produce or entail very frequently pericarditis and subsequent enlargement of the heart. The enlargement in the present instance, and the old adhesions of the pericardium as well as of the pleuræ may therefore be fairly referred to this source. Probably, too, the existing alterations of the heart, and the former occurrence of pericarditis predisposed the patient to the fatal attack after the operation. It is well known that operations and injuries, or even febrile states of system, however induced, are very apt to give rise to fatal inflammation of the pleura or the pericardium, and the present patient would probably be more liable, from previous attacks, to such complications. However this may be we have stated the fact as accurately as possible.

The subject of the present report being that of death from lithotomy, we have of course selected only unsuccessful cases. It cannot, therefore, be imagined that the present is a sample of the success of the surgeons; we are narrating only the failures, in order to point out, as clearly as a few facts can do so, the real causes of death after the operation.

### Abscess of the Pelvis.

It would seem that abscess, that is, a circumscribed collection of matter, in the cellular membrane of the pelvis, is not an unfrequent cause of death after lithotomy. In some instances this would appear to be preceded by the symptoms of general diffuse inflammation of the tissue, which, being checked in some degree, subside into those of limited collection of matter; in other cases, it would seem that there is not extensive inflammation in the first instance, but the symptoms of abscess gradually steal on. There was one feature common to all the cases we have witnessed, a frequent pulse, commencing after the operation and kept up throughout the subsequent stages of the case. This is usually a bad symptom after any operation or accident. The purulent deposites occur for the most part in those in whom it has been present.

It is not at all surprising that abscess of the pelvis should occasionally follow the operation of lithotomy. If diffuse cellular inflammation be a common consequence, the wonder is, not that a collection of matter occurs sometimes, but that it occurs so rarely. How seldom does such inflammation take place in other parts of the body without giving rise to collections of matter. The truth is that abscess does not often follow diffuse cellular inflammation of the pelvis, because the patient commonly dies before the period for its formation has arrived.

Again, if acute cellular inflammation be frequent, it is, à priori, probable that chronic, or less severe cellular inflammation will occur occasionally, and abscess is the result of such inflammation. The unavoidable violence of the operation, and the circumstance of urine being necessitated to pass through cellular membrane recently divided, is likely to give rise to both forms of inflammation of that tissue. It seems to us surprising that so little should have hitherto been said about abscess of the pelvis, for really, from the cases which have occurred at the hospital, it would seem to be sufficiently frequent to attract attention.

CASE 5. *Recto-vesical Lithotomy—Death three Weeks after the Operation—Abscess of the Pelvis.*

Thomas Bickerton, æt. 25, admitted, Oct. 4th, 1830, under the care of Mr. Brodie, with the symptoms of stone in a severe degree. His health was tolerably good, but he was irritable, and rather anxious.

The symptoms had been coming on for about 20 years, and 12 or 14 years before his admission, a calculus had been discovered by sounding. Then his father, hearing of a *solvent*, employed it, and for six years he suffered little. At a subsequent period he again took the solvent, and again felt relief, but it was not permanent. The solvent was bi-carbonate of potass. Many surgeons had examined him, and all, we believe, except one, had refused to perform the lateral operation, on account of the size of the stone. That one gentleman had *not* examined the patient by the rectum. Baron Heurteloup would not drill the stone; he thought it was too large. The calculus, when felt from the rectum, appeared to be of extreme size, and its anterior extremity was partially impacted in the prostatic part of the urethra. The high operation had been proposed, but this was considered unadvisable, for if the stone was of the magnitude imagined, the sonde-à-dard could not have been employed with facility. On mature consideration Mr. Brodie adopted a modification of the lateral and rectal operations, which may prove of great service on future occasions.

Mr. Brodie performed the operation on the 20th October; an injection of one quart of warm water was thrown up on that morning, in order to empty the bowels.

Having introduced the staff he cut upon it with the knife in the perineum in the usual way, and passing the knife along the groove of the staff into the bladder he divided part of the prostate. He then took Blizard's knife, passed it along the staff into the bladder, completed the section of the prostate and part of the neck of the bladder, and turning the edge of the knife towards the right, nicked the wound in the prostate on that side, by which means he acquired additional room at a small expense of division of parts. The staff had lain on the right side of the stone; it was now withdrawn. Mr. Brodie took a curved probe-pointed bistoury, with a sharp-pointed bistoury-blade concealed in it, and capable of being pushed forward and retracted at pleasure. He passed this to the bottom of the wound, and introduced the fore-finger of the right hand into the rectum. Having felt with this finger the button-end of the bistoury, the sharp-pointed blade was pushed forwards, and the wall of the gut penetrated on the finger. This blade was then withdrawn—the probe-pointed bistoury had entered the rectum—and its division was completed from within outwards, by drawing out the bistoury with the probe-end resting on the fore-finger, as is done in the operation for fistula ani. The opening in the rectum at the bottom of the wound was not exactly opposite to the wound in the bladder, in order that a kind of valve might be formed, to prevent the ready transit of the contents of the bowel into the bladder. The remainder of the rectum was divided on its anterior and left lateral aspect. The forceps were introduced and the stone was readily extracted. It did not prove so large as had been anticipated by all who had, on different occasions, examined the patient. It was long, its anterior end was impacted in the prostatic part of the urethra, and from being always held in this situation, it had appeared to be fixed by its magnitude, and the finger in the rectum could never reach its posterior boundary.

The wound obtained by the operation was capable of admitting the egress of a calculus of very large size. The operation itself was simple, scientific, performed without difficulty.

Immediately after the operation he was ordered an anodyne. In the course of two or three hours, hæmorrhage took place, and half a pint of blood was lost. The bleeding appeared to issue from the right side of the wound, and from the depth of about an inch; it was arrested by compressing for half an hour, with the finger, the pudic artery against the ascending ramus of the ischium. He was blanched by the bleeding, and required wine afterwards.

Nothing further of consequence occurred on that or the following day; the pulse, however, was frequent. On the 22d, Mr. Brodie introduced his finger into the wound, and a catheter into the bladder. In the afternoon, the patient said he could only make water by straining much, and he was rather feverish. He had, and for some days afterwards continued to have, some soreness on pressure of the hypogastrium. On the 24th, he was allowed meat. On the 27th, we find it reported that he had less heat of skin, implying, what was indeed the fact, that the skin was generally warmer than natural, as the pulse was more frequent; there was always, in short, pyrexia. The tongue was moist, clean, and chapped. Fæces and urine were passed by the wound. A flexible metallic catheter was passed into the bladder, and retained in it, in order that the urine might drain into an urinal.

*Ordered, Quinæ and acid.*

In the evening he had partially withdrawn the catheter, which was bent; it was re-introduced by the house-surgeon. The patient was irritable and fidgetty. On the following day Mr. Brodie re-adjusted the instrument, with the assistance of the finger in recto; the urine escaped freely. The bowels were rather loose, the tongue inclined to be dry and fissured. In the night the patient again pulled out the instrument. On the next day it was retained in the bladder by means of tapes, &c. The pulse continued frequent, and was rather jerky—the skin hot at nights—the tongue chapped, reddish, rather dry, the bowels rather loose. The urine, which was fæcal, only came through the catheter on pressure of the abdomen.

On Nov. 1, he was ordered salines with bark and hydrargyrus cum cretâ with Dover's powder. On the 2d, a small abscess was discovered at the root of the scrotum anteriorly; it was opened and stinking urinous pus discharged.

On the 3d, the pyrexia continued with a disposition to perspiration, and to relaxation of the bowels. On the 5th, the mouth was slightly aphthous; the urine was alkaline.

*Ordered, chalk mixture, with aromatic confection and laudanum. Port wine.*

6th. Sweated much last night, and had a slight chill. Now very irritable, yet says he is better. Elastic gum catheter passed into the bladder and finger into the wound. The catheter was retained in the bladder for a short time, when he had a rigor, and it was withdrawn.

8, *Vesp.* Patient much worse—very restless—complains much of pain in the wound—slight tenderness in right hypochondrium, and obscure pain in the chest.

*Haustus anodynus.*

He slept during the night, and the nurse reported him better on the following morning. At the house-surgeon's visit, 10, a. m., he found him insensible, in the state of sinking, with subsultus tendinum, small pulse, &c. Brandy was given, and he lingered till 8, a. m. of the 10th, when he died.

*Sectio Cadaveris, 2, p. m. 11th.* The body was considerably emaciated, the loss of flesh having occurred subsequently to the operation.

*Thorax.* Some old adhesions of right lung. At lower margin of left lung a portion attached to the pericardium by recent lymph. The substance of the lung here contained a circumscribed purulent deposite. There were one or two other deposites of lymph, with a sloughy state of the lung, bounded by a distinct false membrane or cyst.

*Abdomen and Pelvis.* Kidneys small, puckered, flabby; their interior dark-coloured, containing some encysted abscesses of dark matter apparently mixed with blood. In some parts calculous matter deposited in portions of the kidney. Ureters large, dark-coloured, rather injected.

Bladder rather contracted—its parietes thickened—its mucous membrane dark-coloured, injected. On its right side a small aperture leading into a kind of cyst in the substance of the bladder, and containing yellow pus. Prostate nicked on both sides—wound not extending beyond the prostate, its margins encrusted with calculous matter.

Rectum generally dark-coloured in its interior—wound not extending very high up, partly united below by lymph, organized and assimilated to the structure of the part. Communication of rectum with prostatic part of bladder, not beyond it.

External to the urethra, rather anterior to the bulb, a sloughy small abscess, not opening into the urethra, but apparently communicating round it with the wound. The abscess descended into the scrotum, on the outside of the tunica vaginalis. The testis was enlarged and hard. There was loose fæcal matter in the prostatic part of the bladder and urethra, but not beyond that.

On the left side of the pelvis, in the cellular membrane external to the peritoneum, an abscess the size of a large egg, with sloughy-looking parietes, and bridles of sloughy cellular membrane running across it. It had no obvious communication with the bladder, wound, nor rectum.

*Cranium.* Not examined.

We have given the preceding case in detail because it is interesting on many accounts. If the symptoms are carefully noted, they will prove to be those which form the leading features of cases of this description. In our note-book we find the following reference to some cases of abscess of the pelvis alluded to by Mr. Brodie on the occasion of the examination of this patient.

*Case A.* Sir Everard Home operated in an old case of stone, in which there was alkaline urine, with ropy mucus. The only reason which induced him to operate was the excessive suffering of the patient. The patient died soon after the operation, and such an abscess was found.

*Case B.* Mr. Brodie operated on a lieutenant in the Navy, who, he believes, was in a pretty good state of health previously to the operation. He died, and such an abscess was found.

*Case C.* Mr. Brodie operated on a clergyman, who was a stout man, and able to preach up to the time of the operation. He died, and such an abscess was discovered.

Mr. Brodie was inclined to believe, when he made these remarks, that the abscess existed previous to the operations. Whether he continues to hold that opinion we cannot say.

CASE 6. *Lithotomy—Death on the tenth day—Abscess of the Pelvis.*

John Barnett, æt. 63, agricultural labourer, admitted Feb. 1st, 1832, under the care of Mr. Keate, with symptoms of stone in the bladder. His health was pretty good. The urine was acid, and not albuminous. On sounding him Mr. Keate felt a stone, apparently about the size of a walnut, and smooth. The stone could be felt from the rectum. The prostate seemed enlarged.

The symptoms had commenced twelve months previously. Three months before his admission he had been sounded, but no caculus could be discovered.

After his admission he was feverish, and there was some pain in the perinæum and in the hypogastrium, on firm pressure. These symptoms were quite removed by some saline medicines, demulcents, and leeches applied to the perinæum.

On the 16th the operation was performed. After the incision into the urethra, some arterial hæmorrhage took place. A beaked knife was used for the incision of the bladder; a small quantity of urine followed its withdrawal, but a larger gush ensued on the introduction of the forceps. The stone was readily felt at the *bas-fond* of the bladder, and seized, but in its long axis. Some difficulty was experienced in consequence, the forceps slipped, the stone was seized a second time and in its short axis, and then it was withdrawn with little force. It was about an inch and a half in length, by an inch in its short diameter, but flattened from side to side. Another stone was felt and extracted with facility; it was of a similar shape, but smaller. Some hæmorrhage continuing, apparently from the bulb, the patient was removed to bed, and then a gum-catheter, with blue lint wrapped round the part of it opposed to the bulb, was introduced into the wound and bladder and retained there. The wound was also stuffed with common lint, and an assistant was ready to make pressure if necessary.

The calculi appeared to consist of the triple phosphate externally, with a lithic acid nucleus. The operation was completed in a short time, four minutes and a half, and to say that it was performed with Mr. Keate's accustomed dexterity is to say much. The patient bore it well.

In an hour after the operation the patient was still bleeding, and had lost in all two or three pints of blood. The lint was now removed and pressure maintained on the bulb by the finger of an assistant for three quarters of an hour. The bleeding was thus arrested. The bladder was washed out several times with warm water, and an opiate was given.

9, p. m. Tolerably easy—pulse 80—constant dripping of bloody fluid through the catheter—bladder again syringed out. This was repeated at midnight, and an opiate again administered.

17th, 9, a. m. Has passed a quiet night, but had little sleep—complains of slight pain in the pubic region, somewhat relieved by fomentations—pulse 96, soft—skin warm—tongue moist—fluid not so discoloured.

10, p. m. Worse. Some heat of skin—pulse 120, irregularly intermitting, which it began to be this afternoon—tongue rather dry in centre—bowels not open. Urine flows pretty freely through the wound; has taken a little beef-tea.

*An anodyne.*

On the next morning he was ordered castor-oil, of which he took three doses; they did not act till towards the evening. On the 18th, there was little alteration, save that he complained of cough, and of stiffness in the arms.

*An anodyne.*

There was little worthy of notice on the 19th. The symptoms were perhaps a shade better, but he was annoyed by purging; the pulse continued frequent and irregularly intermitting. He was ordered an opiate and aromatic draught, and directed to have fish on the following day.

20th, 2, p. m. Passed a tolerably good night. Pulse more frequent, not intermitting—skin warm—tongue rather more dry—aspect slightly sallow—more cough with mucous râle. Urine is retained for a quarter of an hour at a time, and then comes freely.

*Emp. canth. sterno. Linctus in tussim.*

21st, 11, a. m. Countenance not improved—tongue rather more dry—pulse irregularly intermitting. Urine passed with some little difficulty and pain. A catheter was with some trouble introduced into the bladder per urethram. In the evening the pyrexia was rather increased, and there was some tenderness on pressure in the region of the bladder. We need scarcely mention the particular prescriptions; they consisted of salines, with diaphoretics and expectorants, and occasionally narcotics.

In the evening of the 22d, erysipelatous inflammation appeared around the perinæum, and over the nates, and lower part of the back. The general symptoms were little altered.

On the following morning he was ordered *port wine, beef tea, and quina.* On that evening the erysipelas had spread up the back and down the thighs. The pulse was weaker.

On the 25th the erysipelas had spread over the abdomen—the scrotum was of dull red colour, and its cellular texture loaded with solid lymph. The symptoms were those of typhus. He was ordered *a mutton chop and porter.*

On the 26th, he was in a state of complete prostration—the cellular membrane of the scrotum was evidently sloughing, and the skin beginning to slough also. A catheter was passed into the bladder, but no urine was drawn off. Incisions were made in the scrotum. After this the patient sank rapidly, and died at midnight.

*Sectio Cadaveris.* Body rather emaciated.

*Cranium.* Not examined.

*Thorax.* Lungs rather emphysematous—old adhesions of pleuræ on right side. Mucous membrane of bronchi dark, inflamed.

Heart large, with simple hypertrophy of its left ventricle. Aorta dilated, with varicose vasa vasorum. Arterial system generally deficient in elasticity of tissue.

*Abdomen.* Left kidney pale—no other alteration.

*Pelvis and Perinæum.* Bladder rather contracted, thickened—its mucous membrane corrugated, dark, in a state of chronic inflammation. A curious appearance observable about its neck. At the *trigone*, a projection, of the size of a small nut, in the site of the third lobe of the prostate. The incision had completely divided the left lateral lobe, but we cannot exactly say whether the bladder was cut or torn in any way, beyond it. On the opposite side a projection larger than that first described. Whether this arose from the right lateral lobe of the prostate, or was a sort of fungous growth, we cannot exactly say; its structure, when cut, was rather softer than that of the prostate, but of similar colour.

The bulb of the urethra appeared divided. The sides of the whole wound were rather sloughy.

From the wound in the prostate there commenced an abscess, with sloughy parietes, which passed backwards to the left side of the fundus of the bladder, then behind the rectum, occupied a large share of the cellular membrane between the latter and the sacrum, and reached the right side of the intestine, but did not actually touch the right side of the bladder. The contents appeared to be a mixture of pus and urine, or, at all events, of pus and a thinner fluid.

In the wound, near its external opening, was found a body, somewhat like an almond in shape and size, attached by a sort of stalk to the right side of the neck of the bladder, within the wound in it. Was this coagulum partially organized?

CASE 8. *Lithotomy—two Calculi—Symptoms of Diffuse Cellular Inflammation after the Operation — subsequent formation of Abscess. Event yet undetermined.*

Edwin Winter, ætatis 8, admitted Jan. 30th, 1833, under the care of Mr. Keate. The child had the usual symptoms of caculus in the bladder. Mr. Keate introduced a moderate-sized instrument, and was of opinion that the stone was impacted in the neck of the bladder. On examination by the rectum the stone seemed immoveable. The child was delicate, of irritable temperament, remarkably precocious in intellect, though totally uneducated. He almost appeared to illustrate the sarcastic remark of Voltaire in his tale of the *Ingénu,* that education in his day was like a pair of stays, crippling the mind into a modish configuration, and destroying its simple and symmetrical *tournure.* But to the point.

The operation was preceded by the warm-bath and opiates. In the morning of Feb. 7th, on which it was performed, an injection was thrown up. There was slight difficulty in finding the groove of the staff. A bistoury-knife, cutting for a short distance on the concave was tried and abandoned. The bistoire-cachée was then used. The stone extracted was longer than a Barcelona nut, with a projecting nipple; it was extracted readily. Another stone was felt. The bladder was now contracted, and it was necessary on introducing the forceps, to dilate the bladder, by opening the blades before the stone could be extracted. It nearly corresponded to the former in size and shape. The child was much excited during the operation, and correspondingly depressed afterwards.

Very soon he complained of pain in the belly, and in the evening there was some tenderness.

*Hirud.* viij.

8th, 11 a.m. Was very sick during the night — this morning is restless — pulse frequent—tongue moist, furred—bowels not open — skin warm — some thirst—still tenderness of the abdomen.

*Rep. hirud.* viij. *H. Sal. efferv. c. Mag. sulph.* ℨss. *4tis. horis.*

9, p.m. The preceding reports are copied from the note-book of a friend. On this evening we saw the patient for the first time after the operation. Face pale, somewhat anxious—skin warm—pulse frequent — tongue rather dry — sickness continues. Respiration slightly hurried—cerebral functions undisturbed. Belly rather swollen, tympanitic —still some tenderness on pressure, chiefly about hypogastric, inguinal, and iliac regions—urine said to issue freely from the wound.

9, *Mane.* Passed a better night—rejects by sickness his medicine, but retains tea, &c.—rather less abdominal tenderness—bowels have acted once or twice—countenance improved.

*Pulv. ipec. c. Hyd. sub.* āā. grs. ij. *4tis. horis.*

*Vesp.* Continues to be sick on taking medicine or even food.

10th, *Mane.* Has taken five powders. Bowels have been freely opened, and the last stools were of a green colour. Pulse frequent—tongue covered with a brownish fur, and rather dry. No pain now, save on firm pressure.

*Omr. pulv. H. Salin. efferv. c. Conf. ar.* grs. viij. *4tis. horis.*

*Vesp.* Belly no longer swollen—vomits only his medicine.

11th. Passed a good night—no sickness since 1, a. m. Pulse 120—tongue still dry—still tenderness in right iliac, inguinal, and pubic regions on firm pressure.

*Beef-tea,* ℔j. *Hirud.* vj.

12th. Looks better—pulse still 120 —and still tenderness in regions mentioned.

*Acidi nit. dil.,* ♏v. *Syrupi,* ʒij *Aq.* Oss. *quotid. bibend.*

13th. *Hirud.* vj.

14th. Since last application of leeches tenderness is quite gone, and abomen has regained its natural flaccidity.

15th. Again slight tenderness in right inguinal region. The sides of the wound are coated with the phosphates.

*Hirud.* iv.

After this he had no return of pain, and on the 24th he was allowed beef-tea and meat. On the night of the 25th the urine passed through the urethra for the first time; it issued again by this passage on the following morning. There was still pain in the inguinal region, and some induration was felt there.

On the morning of the 27th he had a severe rigor, which occurred about half an hour after the passage of urine through the wound, and was attributed to that circumstance. Nothing further occurred till March 6th, when he had evidently pyrexia, and complained of pain in the right groin, where there was tenderness, with much thickening and induration. The pulse was 140.

*Hirud.* iv. *Rhubarb and magnesia.*

*Vesp.* Is and has been very sick—aspect pale, anxious, having all the appearance of persons labouring under the collections of matter — tongue dryish, colourless. On making firm pressure in the groin and separating the edges of the wound in the perinæum, much thin flocculent matter issued from the latter with a spurt. On diminishing the pressure in the groin the matter ceased to flow from the perinæum; on repeating the pressure, the matter again issued, shewing most distinctly a connexion between the abscess in the inguinal region and the wound. After a short time the fulness in the groin was sensibly lessened. The child was put in a warm-bath, and an injection was administered.

Since that period there has been no return of rigor, nor of sickness. Matter has continued to flow from the wound, and it has been mixed with urine. There is still, however, induration and tumefaction in the groin—the boy's aspect is pale, though not anxious—he has sensibly emaciated—and the pulse, whenever we have seen him, has been 120 or upwards. These cannot but be looked on as serious symptoms. There must be a considerable abscess extending from the cellular membrane at the neck of the bladder, along the cellular membrane behind the os pubis, to that in the iliac fossa. Under such circumstances any slight increase of inflammatory action, any accidental retention of the matter, might speedily give rise to that formidable train of symptoms too frequently observed, and characterizing purulent collections or deposites. We shall state hereafter the result of this interesting case.

LXXXVIII.

Surgical Account of the Siege of Antwerp.

Dr. Paillard, actuated by a most laudable zeal for the acquisition of surgical knowledge, repaired to Antwerp two days before the close of the siege. "I had then (says he), on the one hand, the magnificent spectacle of an army attacking, with all the resources of the most perfect science, and with a courage truly superhuman (!), a formidable citadel, defended with the most gallant obstinacy; and, on the other, an ample field for observing almost all the accidents of military surgery." The French army crossed the Belgian frontier on the 15th Nov. in their progress to Antwerp. To each division of the army was attached an "ambulance," consisting of a "chirurgien-major," an "aide-major," four "sous-aides," and two dispensers.

M. Zinck, principal surgeon, directed the medical establishment. His ambulance, called the ambulance of reserve, was at head-quarters, and there almost all the wounded were received, and the different operations performed. It was situated at Berchem, a village near to Antwerp: and in order that relief might be instantly given to the soldiers under urgent circumstances, a smaller ambulance was detached from the main one, and posted immediately behind the trenches. This ambulance, in siege warfare, may, therefore, be said to correspond to the "ambulance volante" in an open engagement. Our readers are probably aware, that Baron Larrey has the high merit of having introduced this admirable improvement into the medical service of the army, in the year 1792, during the campaign on the Rhine.

A surgeon and two assistants were constantly attached to the "ambulance de tranchée;" but relieved frequently from Berchem. Their duties exposed them to very considerable danger, as the balls and bombs of the enemy repeatedly passed through the walls of the church and house in which the ambulance was established. When the wounded were brought to the chief ambulance, they were immediately dressed or operated upon, and, after a few hours' rest, sent forward either to Antwerp or Malines. This was done twice a-day—in the morning, and again in the afternoon.

In addition to the wounded French, upwards of 70 of the Dutch garrison were discharged, after the 24th of Dec., from the citadel upon the military hospital of Antwerp; these poor fellows were in a deplorable state; not that we attach any blame to the Dutch surgeons (as has been most unjustly done in some French and Belgian journals), for, considering the difficulties which surrounded them, and the terrors of the sufferers themselves, we are ready to exculpate them from all such charges. We have only to remember that 350 wounded were crowded into a narrow place, lying on straw, and so near to each other that they literally were in contact, and that the surgeons were obliged to stoop down almost on their hands, and to sit astride their patients (so low was the roof of the casemates) while dressing their wounds or performing the necessary operations. So offensive was the air in this hospital, that they could not breathe with ease. One requires to have seen the citadel, after its surrender, to form any adequate idea of the wreck and chaos which had been made. From the 10th of December to the 23rd, the day on which it capitulated, almost all the buildings had been shivered or burned; and that part of the garrison which was off duty, had no where to shelter themselves but in the galleries, and these were so crowded, that one-half of the soldiers had to stand, while the other half lay down to take a few hours' rest. In the few last days of the siege, part even of these shelters had been demolished, and there was scarcely a place of safety left. So incessant was the fire of our artillery, that, according to calculations, a ball or bomb fell on the citadel every minute. Even the casemated hospital was pierced in several places by the shells, which exploded either in the midst of, or close to, the wounded men; and you might have seen the poor fellows trying to crawl away from the reach of the shivers. How, then, can we be surprized that many of the wounds and other injuries exhibited such a lamentable condition. By virtue of an article of the capitulation, the wounded Dutch, amounting to about 300, were conveyed from the citadel on board vessels to Bergen-op-Zoom. There had been upwards of 200 of the garrison killed, and these had been hurriedly buried immediately under the soil, so that when this was kicked away, the half-putrid corpses were exposed. The French army had rather more than 100 killed, and nearly 700 wounded. An ample field was thus most unfortunately presented for observing all sorts of gun-shot injuries.

Among the medical men attracted by a zeal for their profession, were several young English surgeons. We shall select a few of the most interesting observations, with a preliminary remark or two.

### Contusions.

Dupuytren, in his admirable lectures, now in the course of publication, admits four degrees; the first consists of a rupture of the small vessels of the part, infiltration of blood and ecchymosis; the second of a rupture of vessels of a larger size, laceration of the texture of the part, and considerable effusion of blood into its substance;—in the third degree of the injury, there is a deeper and more complete alteration, and very frequently mortification; and in the fourth, there is an absolute contrition and entire disorganization of the wounded part. As is well known, little danger attends the first description of these injuries, except when the ball has struck upon a part, under which there is any important organ, or upon glandular structures, or upon some of the organs of sense, as the eye; the following is an illustration of one of these exceptions.

### Amaurosis from Contusion of the Eye-ball.

F., aged 26, was in the trenches on the 7th December. A bomb fell among the detachment where he was; all the soldiers immediately fell flat upon their faces; it exploded between the legs of our patient, and he only was wounded by its fragments—the left limb was so much injured as to require immediate amputation; his cap was shivered to pieces, and he received a number of contusions on different parts of his body; a small fragment had struck the eye, which caused considerable pain at the time, and was followed by ecchymosis of the lower eyelid—but as this appeared to be of little moment, the surgeon overlooked it. The man speedily recovered from the operation; but then unfortunately it was discovered that there was complete amaurosis of the injured eye, although no appearance of the injury remained. Many similar cases are observed in hospitals, and are generally produced by a blow of the fist, or thrust of any blunt body on the eyeball;—the amaurosis is generally incurable, although the humours of the eye retain a perfect transparency. It is in cases of the second degree of contusion that we observe those deceptive swellings, which, when they occur on the head or chest, have frequently misled the young surgeon to suppose that the subjacent bone has been fractured and depressed. The effused blood forms a circumscribed puddle, and the surrounding cellular substance becomes prominent, firm, and unyielding, so that it may be readily mistaken for the edge of the broken bone. It is worthy of remark also, that these swellings sometimes pulsate strongly for several hours, and thus somewhat simulate aneurisms; in a short time, however, the parts become so distended with the blood, that they must resist any further effusion, and the pulsations cease. Under the fourth degree of contusions are comprehended those most destructive injuries, in which the skin remains unhurt, but all the subjacent structures are frightfully changed, the bones smashed, the blood-vessels torn, and the muscles and sinews beaten into a jelly. These accidents used to be attributed to the wind of the ball, or to an electrical cause, brought into play by its rapid fraction with the air; but it is well known now by surgeons, although the old opinion still prevails among soldiers, that they are the result of a ball nearly spent actually striking the part. We shall very briefly mention one or two cases, to shew the different effects of point-blank and of spent shots. A soldier in the trenches was struck on the belly by a cannon-ball in its full fury. The whole of the abdominal parietes was carried away, and the crista ilii was literally cleanly cut, or sliced off;—the intestines hung out of this horrid wound, but they had not been injured; at least there was no appearance of any lesion;—the poor fellow died almost immediately. Contrast this case with the following. A cannon-ball struck another soldier on the left flank; no outward wound or bruise was to be seen; and his comrades thought that he shammed, in order to quit the field. But on examining the part which had been struck, the surgeon at once recognized that it was fluctuating, and disorganised to a great depth; and after death, the abdominal muscles, the sacro-lumbalis, and the left kidney were found to have been reduced to a jelly, the lumbar nerves torn across, the transverse processes of the lumbar vertebræ, and the last ribs crumbling, and there was a large quantity of black blood within the abdomen and chest;—the skin alone appeared to have escaped, probably from its great elasticity.

I saw a case in the hospital at Antwerp of a soldier whose arm had been struck by a spent-ball—the skin and soft parts were uninjured, but the humerus was dislocated.

Many cases occurred of contusions of the chest, in which frightful hæmoptysis occurred; venesection and other depletory measures were used, with the happiest results, provided the internal injury had not been very serious. When the spine is the seat of the wound, paraplegia is a frequent consequence.

Larrey mentions a curious case of incomplete palsy of the pneumogastric nerve, from the ball striking the region of the stomach; the man lost his voice and taste completely, suffered from dyspnœa and dyspepsia, and the stomach had become insensible to emetics. Ventral hernia is another effect, which is often found to follow these injuries; but a far more disastrous consequence is rupture of the intestines, liver, spleen, or bladder. It is well known to hospital surgeons, that these accidents may be produced by the passage of a wheel over the belly, or the kick of a horse.—*Journ. Hebdom.*

Having been at this now famous siege, having witnessed the cases in the military hospital of Antwerp, to many of which attention will be drawn, no doubt, by M. Paillard, having seen and conversed with that gentleman himself, and being one of those "young English surgeons" to whom M. Paillard alludes, we may venture to offer a remark or two on what we witnessed.

Arriving too late for the siege itself, on the very day in fact upon which it was terminated, we had no opportunity of seeing the wounded immediately on the reception of their wounds. When we reached Antwerp the ambulances were broken up; the temporary hospital at Berchem, the head quarters of Marshal Gerard and of the army, distant about a mile and a half from Antwerp, and in the immediate vicinity of the trenches, was broken up also; and the wounded, French and Dutch, were lodged in the military hospital at Antwerp, then under the charge of M. Seutin, a Belgian surgeon-in-chief. We were told that in this hospital, which formerly had been a convent, there were upwards of a thousand, indeed fourteen hundred, wounded. Whether this computation was strictly accurate we cannot say, but certainly the number of the patients was considerable. The arrangements of the hospital were extremely good; it was clean, airy, well ventilated, and well warmed. The wards were in general large, holding from twenty to sixty patients or more. M. Seutin had under his direction hospital assistants, one or two of whom were intelligent, the rest but so so. Of M. Seutin himself we must speak in very high terms. He appears to have been long accustomed to the precision and dispatch of a military hospital, yet cautious and careful where the case demanded it. Possessed of extreme manual dexterity and precision, he had the head to direct as well as the hand to execute, and was altogether a good specimen of a continental surgeon. He treated the English visitors with urbanity, and shewed them more attention than foreigners would probably have received in this Country. We gladly take this opportunity of expressing our thanks to M. Seutin, and we shall feel gratified if this expression of the high estimation in which we hold his talents should chance to meet his eye.

The cases in the hospital were for the most part severe, but perhaps the worst had been disposed of before our visit. A very long ward was filled with the cases in which amputation had been performed. If our memory serves us, union by the first intention had only taken place in one instance, and in that the amputation had been performed, and the dressings finally adjusted, in the ambulance or upon the field. We must say, however, that the mode in which we saw a stump dressed in the hospital immediately after the performance of amputation, was by no means calculated to ensure primary union. The soft parts were very imperfectly drawn down and supported, a quantity of loose charpie was placed at the extremity of the stump, (not *in* it,) and as no efficient precautions were taken to prevent retraction, we did not expect that union by the first intention would occur. When to this vicious mode of dressing we add the circumstance of the kind of diet usually taken by the French soldiery, and the very low regimen on which patients are usually kept after operations, we cannot be surprised at the want of success in obtaining union.

If, however, we find reason to condemn the system of dressing adopted in reference to stumps, we must exempt from this censure the treatment of compound fractures. So far as we could judge by the cases which we saw, this was very good. M. Seutin dressed two or three compound fractures of the thigh, and the manner in which he did so was highly creditable to himself, and to the school in which he had been formed. We certainly never saw in this country anything approaching to so masterly an exhibition of surgical manipulation. The compound fractures of the thigh were treated by the extended position of the limb, with the long splints of Dessault, a mode of practice which was found so advantageous in our own army during the Peninsular war, that a general order was issued for its general adoption.

A fracture-bed was employed in this and in other Belgian hospitals, which might very advantageously be introduced into this country. The patient lay upon a frame, which admitted of elevation or depression by pullies, attached to the top of the bed-posts. Proper apertures for the natural evacuations were made in the frame, and it could be opened, if necessary, at other places. By this means, wounds in the inferior surface of the body, or of the lower extremities, might be dressed from below, without disturbance to the patient or inconvenience to the surgeon.

We fear our limited space must compel us to stop here. We had intended to offer some further remarks, but we must defer them till our next, when we will notice some particular facts.

---

### LXXXIX.

### Mr. T. M'Carty's Miniature Bust of Mr. Brooks.

This is a very excellent likeness of a man well known and highly esteemed by a large circle of the profession. The artist is a young man of merit, the son of a half-pay officer, who is laudably turning his talents to the support of himself and family. The price is only a guinea, and the bust makes a handsome ornament for the mantlepiece of the medical practitioner's library. We have placed it in our own library, as a mark of respect for the dead, and of encouragement for the living. It may be seen at Mr. Churchill's, (late Callow and Wilson's,) Princes Street, Soho.

---

### XC.

### MEDICAL REFORM. COLLEGE OF PHYSICIANS.

RUMOUR, with a hundred tongues, has lately been busy in circulating reports of meditated changes in the temple of Esculapius, in Pall Mall East. It is confidently asserted that mighty matters are agitated in the councils there, and that measures are in progress which will give general satisfaction, not only to the now alienated licentiates, but to the graduates of all respectable universities, who are now practising throughout the kingdom. We shall be highly gratified, but somewhat surprized if this prove true. We confess we are much less sanguine in our expectations than some of our friends and contemporaries, on this point. Kings and corporations have so seldom surrendered rights or privileges, voluntarily, and extended them to those beyond the pale of power, that such an event is not merely an exception to the general rule, but an exception to exceptions.

It is possible, however, that the spirit of monopoly may abate, when the advantages originally attached to it begin to diminish. We cannot believe that the most strenuous advocate for the present invidious distinction between Fellow and Licentiate, is so dull of apprehension as not to perceive that the day is fast approaching when the said distinction will be an actual disadvantage to him who is now supposed to profit by it. The *medical* education of the Fellow and the Licentiate, must now be the same; and the classical education, under existing circumstances, will differ very little. The professional success of the parties must depend on talent, and that talent is as likely to be on one side as on the other. Indeed the *aggregate* of this article is sure to be on the part of the Licentiates, from their numerical superiority, which is daily increasing. See, then, what is likely to be the result. The College, by degrading, as far as in its power lies, the great body of Licentiates, is perpetually recruiting a corps that must, while human nature is the same, be in active hostility to the oppressive corporation. It must be a very sorry consolation to that corps, that its members will, as far as possible, oppose hostility to hostility, and thus perpetuate the warfare, "even to the knife," between the contending factions! The College advocate, who can calmly contemplate this scene of feud and strife in a profession that ought to be liberal and united, is possessed of a mind which we do not envy, and which in the end will not, perhaps, prove very advantageous to its owner. Both parties are now too extensive to act, in any thing like *union*, against each other; but the aggrieved party, being also the most numerous, will certainly bear in mind their wrongs, and act individually with hostile feelings towards the other. This enmity must be increased, too, by that most Machiavelian policy, the annual transplantation of a member or members from among the most distinguished of the Licentiates, to grace the tail of the Fellows! It is a piece of policy, founded on a thorough knowledge of the worst principles in human nature—vanity and self-interest! It is worthy of the darkest days of the Vatican and the Inquisition. The cruelty of the injury is rendered ten-fold more poignant and irritating by the mask under which it is perpetrated—that of LIBERALITY!!

The real merits of the case are now obtaining light among the community at large—and that to the disadvantage of the College monopolist. Medical men well know indeed that the *professional* education of the Oxonian or Cantab, is as good as that of the Edinburgh graduate; but the public are beginning to entertain an opinion that the Fellow of the College, who prides himself on a degree from the banks of the Cam and the Isis, rests his distinction on university honours, of which they naturally entertain some distrust, when their health and lives are subjected to the trial of purely medical knowledge. The FELLOW may hug himself in the pleasing conviction that his academic education in one of the English Universities, must secure him the confidence of the sick man; and he will not pay the slightest attention to the hint which we now throw out, respecting his self-deception. Yet scarcely a day passes in which we do not hear the observation made by patients, that Oxford and Cambridge degrees will not now do in physic; and the recent example of a Collegiate Fellow openly espousing the cause of an ignorant quack, has not done much to prejudice the public in favour of the College.

Our contemporary, of the Medical Gazette, having hazarded an expression that the Licentiate was *degraded*, one of that order indignantly replies in the negative. Let us see. A man having laboured hard at Edinburgh, Paris, or elsewhere for three or four years, and taken his degree with honour, comes to London to practise. What does the College? It tramples the gradus of his alma mater under foot, and subjects him to three monthly examinations. In the first, he is ordered to describe the os frontis, or some other bone—in the second, he is asked what is the difference between a quotidian and a tertian fever —and, in the third, how much opium there is in a scruple of Dover's powder? If he answers these or such like questions tolerably well, he is placed on his bended knees to take a long Latin oath of fealty to that College which instantly expels him from its portals, and prohibits him from re-entering it, except by the express invitation of its president—nay, he is not even allowed to designate himself as a *member*—not a foot or even toe of that institution which he has sworn to reverence and aggrandize by all means in his power!! Now, if this be not DEGRADATION in the very letter as well as the spirit of the word, we are equally ignorant of language and of facts. Much do we fear that too many of the licentiates are not only degraded but *perjured* by the treatment which they receive, and the oath which they take. They must, indeed, be more or less than mortals, who can honour and promote the interests of an institution by which they are degraded, and turned adrift as outcasts!

Is it imagined that three or four monthly invitations to tea and coffee, in Pall-Mall East, where they are exhibited, in their helot condition, to the gaze of bishops, judges, and officers of the crown, can reconcile the licentiates to their abject state of vassalage? No verily! The red granite pillars (or their imitations) in the library, blush for the graduates of Edinburgh and Paris, when they go in with a ticket of permission from the president and fellows! We do not mince the matter, or disguise the feelings of the licentiates, because we sincerely wish to see union, peace, and friendship between all the members of the medical profession. We are quite sure that the bond of good *fellowship* will never be cemented between the College and its *permissi* till the invidious distinction in question be obliterated. This obliteration, as we said before, is more to be wished than expected; because corporations will do things *collectively*, which the *individuals*, of which they are composed, would not sanction, in their private capacity.—Much, indeed, may be hoped from the temper of the times, the clear perceptions of the talented president, and the liberal disposition of *many* of the fellows of the College. Still, we fear that these combined influences will be insufficient, unless the licentiates, and the regular physicians throughout the kingdom throw off their supineness, and put their hands to the plough. The procedure is simple, constitutional, and must be successful in the end. An association (not a faction) should be formed for the redress of grievances. A committee should be appointed to draw up a calm, dispassionate, but firm address to the College, in the first instance, and to the Legislature, if necessary, stating the existing condition of the medical body politic in this country, and portraying the evils which flow from the present state of things. This petition should be circulated by tens of thousands through the community at large, in order that the grievance may be fully known and understood by the public. Of what use are declamations in medical journals, unless they stimulate to a united remonstrance from the regular graduates of England, Ireland, and Scotland, to the Legislature? This is the only procedure that offers the slightest chance of success, unless the College itself should anticipate such an appeal to Parliament and the public, by a liberal and effectual measure of reformation and concession.

Should such a piece of timely wisdom be evinced by this institution, we are confident that the College of Physicians will flourish as one of the most distinguished and enlightened bodies of professional men in Europe. Every regular physician in the three kingdoms would hasten to enrol his name as a member, and such a library and museum would gradually and rapidly arise, as might compete with any of the kind in the world. The College would then present lectures, publish transactions, and exhibit at its monthly meetings a re-union of the elite of the profession in this country.

---

